# Diels–Alder Cycloaddition with CO, CO_2_, SO_2_, or N_2_ Extrusion: A Powerful Tool for Material Chemistry

**DOI:** 10.3390/ma15010172

**Published:** 2021-12-27

**Authors:** Stanisław Krompiec, Aneta Kurpanik-Wójcik, Marek Matussek, Bogumiła Gołek, Angelika Mieszczanin, Aleksandra Fijołek

**Affiliations:** Institute of Chemistry, Faculty of Science and Technology, University of Silesia, Bankowa 14, 40-007 Katowice, Poland; stanislaw.krompiec@us.edu.pl (S.K.); bj.golek@gmail.com (B.G.); angelika.mieszczanin@gmail.com (A.M.); fijolek.aleksandra@wp.pl (A.F.)

**Keywords:** Diels–Alder cycloaddition, extrusion, APEX strategy, functionalized nanomaterials, nanographenes, organic electronics, scholl reaction, polycyclic aromatic hydrocarbons, polyarenes

## Abstract

Phenyl, naphthyl, polyarylphenyl, coronene, and other aromatic and polyaromatic moieties primarily influence the final materials’ properties. One of the synthetic tools used to implement (hetero)aromatic moieties into final structures is Diels–Alder cycloaddition (DAC), typically combined with Scholl dehydrocondensation. Substituted 2-pyranones, 1,1-dioxothiophenes, and, especially, 1,3-cyclopentadienones are valuable substrates for [4 + 2] cycloaddition, leading to multisubstituted derivatives of benzene, naphthalene, and other aromatics. Cycloadditions of dienes can be carried out with extrusion of carbon dioxide, carbon oxide, or sulphur dioxide. When pyranones, dioxothiophenes, or cyclopentadienones and DA cycloaddition are aided with acetylenes including masked ones, conjugated or isolated diynes, or polyynes and arynes, aromatic systems are obtained. This review covers the development and the current state of knowledge regarding thermal DA cycloaddition of dienes mentioned above and dienophiles leading to (hetero)aromatics via CO, CO_2_, or SO_2_ extrusion. Particular attention was paid to the role that introduced aromatic moieties play in designing molecular structures with expected properties. Undoubtedly, the DAC variants described in this review, combined with other modern synthetic tools, constitute a convenient and efficient way of obtaining functionalized nanomaterials, continually showing the potential to impact materials sciences and new technologies in the nearest future.

## 1. Introduction

**Scheme 1:**
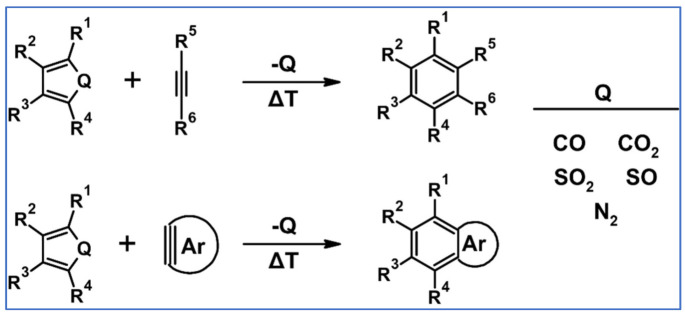
General scheme of reactions presented in this review. R^1^–R^4^ = H, F, Cl, Br, alkyl, Oalkyl, aryl, hetaryl, Nalkyl_2_, Naryl_2_, and others (substituents identical—except for H—or differentiated); R^5^–R^6^ = H, I, alkyl, aryl, hetaryl, arylethynyl, and others (substituents identical—except for I—or differentiated); arynes = benzyne, substituted benzyne, naphthynes, and other arynes.

Undoubtedly, Diels–Alder [4 + 2] cycloaddition is one of the most crucial reactions for modern organic synthesis. Therefore, those reactions’ benefits and almost unlimited possibilities are well-acknowledged and will not be mentioned further. Nevertheless, it is hard to imagine modern technologies, especially organic electronics and photovoltaics industries—dyes and pigments, pharmaceuticals, agrochemicals, and materials chemistry in general—without such a cycloaddition. This review comprises a state of knowledge and development perspectives regarding a chosen class of DA cycloaddition leading to aromatic products. Reagents of discussed reactions include cyclopentadienones, 2-pyranones, 1,1-dioxothiophenes, 1,2-diazines, and 1,2,4,5-tetrazines as dienes and acetylenes (including masked ones), and diynes and arynes as dienophiles. Respectively, those cycloadditions of compounds mentioned above are followed by CO (for cyclopentadienones), CO_2_ (pyranones), SO_2_ (1,1-dioxothiophenes), and N_2_ (diazines and tetrazines) extrusion from DA-cycloadduct (Scheme 1). This work focuses on the thermal and non-catalytic (except for one example) reaction; it does not discuss reagents other than those necessary for in situ aryne generation. Special attention has been paid to materials chemistry, indicating that the discussed cycloadditions, especially combined with another novel synthetic tool, constitute a powerful and versatile tool for materials chemistry. Of particular importance in the aforementioned combination of different reactions is Scholl dehydrocondensation allowing π-expansion from DA cycloaddition (and other types of cycloaddition and coupling reactions) products [1,2,3,4,5,6,7,8,9,10,11,12,13].

Such a tandem approach suits various applications as it allows access to functionalized nanomaterials, for instance, nanographenes and polymers of desired properties from relatively simple and readily available substrates. Due to their unique optical properties, including nonlinear, electrochemical, and photophysical features, nanomaterials obtained in this way are considered promising biosensors, materials for OLED technology and solar cells technology, conductive polymers, nanoreactors or ligands for catalysts, as well as semiconductors, detectors of explosive substances, and others. The application possibilities listed above have been discussed in this review. Particular attention is paid to nanographenes, including functionalized and heteroatoms-doped ones, which preparation and new applications constitute a “hot topic” in science and technology [6,11,12,13,14,15,16,17,18,19,20,21,22,23,24,25,26,27,28,29,30,31,32,33,34,35,36,37,38,39,40,41,42,43,44,45,46,47,48,49,50,51,52,53]. Currently, only bottom-up methods, where nanographene is constructed from smaller entities by stepwise organic synthesis, allow the preparation of nanographenes, including functionalized and doped ones, with perfect control of shapes and sizes and, therefore, perfectly defined properties [2,5,6,11,12,14,15,16,17,20,21,22,23,24,25,26,27,28,29,30,31,32,33,34,35,36,37,38,39,40,43,47,48,51,52,53]. The reactions presented in this review fit perfectly into the previously mentioned synthetic strategy. It should be added that nanographenes (graphenoids), obtained via reactions discussed in this review, can be planar or otherwise distorted, that is, non-planar, one- (1D), two- (2D), and three-dimensional (3D) [6,11,12,17,23,24,25,26,36,38,40,43,46,47,49,52]. Graphenoids with planar sheets of varying size, shape, and periphery have made great progress in practical applications of electronics, energy storage, catalysis, and biosensing [6,11,12,14,15,16,17,20,21,22,23,24,25,26,27,28,29,30,31,32,33,34,35,36,37,38,39,40,42,44,47,50,52,53]. In recent years, a growing number of graphenoid studies have focused on unusual curved and warped structures such as bowls, helices, and saddles, on account of their structural variation and unique physical and chemical properties [6,11,12,14,15,16,17,20,22,23,24,25,26,27,32,33,35,36,37,38,40,44,47,49,50,51]. The discussed reactions were grouped according to the type of diene and the aromatic system resulting from cycloaddition, i.e., polysubstituted benzene or polysubstituted naphthalene. In the formation of the benzene ring, the role of dienophiles played acetylenes, including masked ones and diynes. In turn, naphthalenic systems were created when arynes acted as dienophiles. Cycloaddition products were frequently subjected to further transformations (e.g., functionalization) and, in particular, dehydrocondensation, which ultimately resulted in π-extended polyaromatic structures, notably nanographenes.

Scientific papers devoted to our review were discussed chronologically, from the oldest to the newest. Such an approach allows us to track the development of the presented transformations variants, starting with DA cycloaddition, CO, CO_2_, N_2_, or SO_2_ extrusion, and finally aromatization. Notably, the chronological representation provides insight into how these reactions’ role in the field of material chemistry increases over time. This applies in particular to functionalized nanagraphenes. Thanks to the combination of DA cycloaddition with Q extrusion (Q = CO, CO_2_, and others) with Sonogashira coupling, Buchwald–Hartwig amination, especially with Scholl dehydrocondensation, it became possible to obtain nanographenes of various shapes and sizes.

It should be emphasized that the mechanism of the first stage of this at least two-stage transformation, i.e., the DA-cycloadduct stage, is well known. On the other hand, the mechanism of the extrusion step, which is of key importance for forming the aromatic product—has not been described to date. The complete mechanism of this complex reaction leading to aromatic structures remains a challenge for further research, as we write in conclusion. The reference system for the reactions described in this review can be thermal cycloaddition of alkynes and arynes into perylene bay region—acting as a conjugated diene, with H_2_ extrusion. We described known reactions of this type, including mechanistic considerations, in our 2020 [54] review and our work from 2020 [55].

## 2. Cyclopentadienones for the Multisubstituted Benzene Ring and Naphthalene System Formation

Regarding DA cycloadditions with extrusion of gaseous CO, CO_2_, SO_2_, or N_2_, cyclopentadienones represent the most commonly used group of dienes. This is as the synthesis of these compounds, especially tetraarylsubstituted ones, has been tackled effectively and successfully. It could be boldly said that the synthesis of any designed cyclopentadienone is achievable. Moreover, such structures, especially the most important ones, i.e., tetraarylsubstituted derivatives, are thermally stable; therefore, it is possible to perform cycloaddition reactions even at high temperatures. The great importance of tetraarylsubstituted cyclopentadienones is due to the fact that DA cycloaddition with CO extrusion is commonly realized as a sequence followed by Scholl cyclodehydrocondensation. This sequence allows the formation of coronene or even more extended nanographene systems. Partners for cyclopentadienones in the reactions with CO extrusion are, e.g., acetylenes (including masked ones), diynes or arynes. This review does not include any research on the formation of benzene rings via nucleophilic cycloaddition. The mechanism of such reactions does not meet the criteria of the reaction sequence (DA cycloaddition followed by aromatization via CO, CO_2_, SO_2_, or N_2_ extrusion).

### 2.1. Cyclopentadienones for the Multisubstituted Benzene Ring Formation

This section discusses DA cycloaddition with CO extrusion reactions, the products of which were substituted benzenes up to hexasubstituted ones. Cyclopentadienones played the role of dienes, while mono- and disubstituted acetylenes, masked acetylenes, conjugated and isolated diynes were applied as dienophiles.

#### 2.1.1. Cycloaddition of Cyclopentadienones and Monoynes

**Scheme 2:**
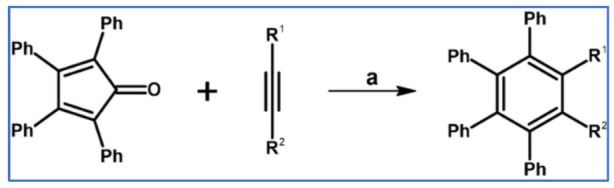
Synthesis of 3,4,5,6-tetraphenyl-1,2-benzenedicarboxylates and phenyl-pentaphenyl(phenyl) ketone via DA cycloaddition with CO extrusion [56]. Reagents and conditions: (a) a = heating over a free flame, Y = 31% (R^1^ = R^2^ = COOMe); (b) a = heating up to 310 °C, Y = 22% (for R^1^ = R^2^ = COOEt); (c) a = heating up to 195 °C, Y = 93% (for R^1^ = Ph, R^2^ = COPh).

In 1934, DA cycloaddition between tetraphenylcyclopentadienone and selected acetylenic dienophiles had already been described (Scheme 2) [56].

**Scheme 3:**
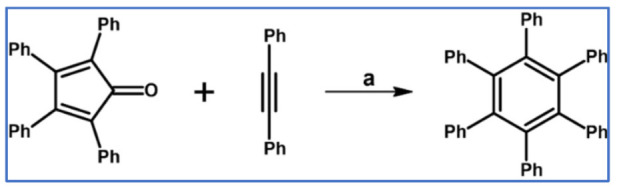
Model reaction for the DA cycloaddition with CO extrusion: synthesis of hexaphenylbenzene via DA cycloaddition of tetraphenylcyclopentadienone and 1,2-diphenylacetylene. Reagents and conditions: a = benzophenone, rfx, approx. 45 min, Y = 84%.

DA cycloaddition of tetraphenylcyclopentadienone and diphenylacetylene leading to hexaphenylbenzene undoubtedly can be the model reaction of cycloaddition of acetylenic dienophiles to cyclopentadienones, with CO extrusion (Scheme 3) [57,58,59].

The above-mentioned reaction was realized for the first time in 1934, but later was modified and improved [57,59]. It should be added that alternatively, hexaphenylbenzene can be obtained via catalytic cyclotrimerization of diphenylacetylene [58].

**Scheme 4:**
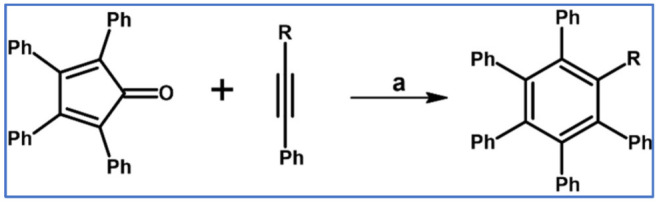
Tetraphenylcyclopentadiene and acetylenic dienophiles Ph–C≡C–R type as partners in the DA cycloaddition with CO extrusion: R influence on the reaction rate [60]. Reagents and conditions: R = H, CH_3_, CH_2_OH, COOH, COOCH_3_, CHO; a = *p*-cymene or toluene, rfx, up to 80 h, up to 89% yield.

In 1952 Dutkowski and Becker examined the electronic influence of R substituent in the R–C≡C–Ph type dienophiles on the DA cycloaddition (with CO extrusion) tetraphenylcyclopentadienone played the role of diene (Scheme 4) [60].

It was stated that the cycloaddition rate increased in the following order: CH_3_ < CH_2_OH < COOH < COOCH_3_ < CHO. According to current knowledge, it is the result of gradually decreasing LUMO energy of the dienophiles [58]. However, in our opinion, for deeper analysis, further DFT calculations and steric effect considerations concerning the size of the R substituent would be required.

**Scheme 5:**
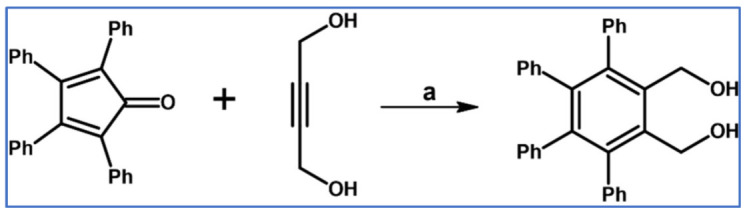
DA cycloaddition with CO extrusion in the synthesis of 3,4,5,6-tetraphenyl-1,2-bis(hydroxymethyl)benzene [61]. Reagents and conditions: a = *p*-cymene, rfx, 11 h, Y = 58%.

In 1953, Bonner et al., described the DA cycloaddition between tetraphenylcyclopentadienone and 2-butyne-1,4-diol in boiling *p*-cymene (Scheme 5) [61].

**Scheme 6:**
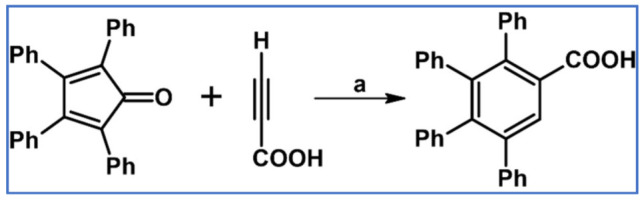
DA cycloaddition with CO extrusion: synthesis of tetraphenylbenzenecarboxylic acid from tetraphenylcyclopentadienone and propynoic acid [62]. Reagents and conditions: a = bromobenzene, rfx, 8 h, Y = 62%.

As reported by Doering et al., tetraphenylbenzoic acid was obtained via DA cycloaddition between propynoic acid and tetraphenylcyclopentadienone, with high yield (Scheme 6) [62].

**Scheme 7:**
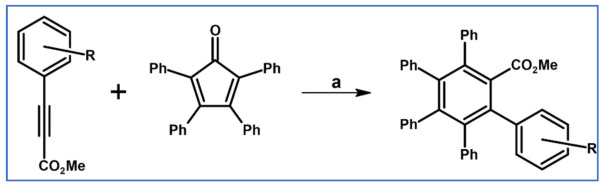
DA cycloaddition with CO extrusion. a = kinetic studies.

In 1958, a kinetic study of DA cycloaddition with CO extrusion using substituted methyl phenylpropiolate and tetraphenylcyclopentadienone as dienophile and diene, respectively, were presented (Scheme 7) [63].

It has been shown that it is a second order reaction, that electron withdrawing substituents increase the reaction rate, the activation energy for R = *o*-Cl and *p*-Cl are almost identical, and activation entropy for aforementioned substituents is strongly negative and nearly the same.

**Scheme 8:**
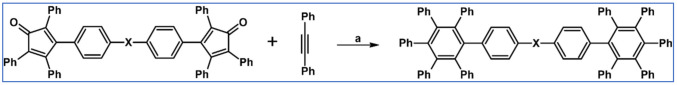
DA cycloaddition of bis-cyclopentadienones and diphenylacetylene: synthesis of bis[(pentaphenyl)benzenes] [64]. Reagents and conditions: X = nil, CH_2_, *O* or *S*; a = heating up to 260 °C, up to 1 h (it depends on the linker structure), up to 81% yield.

Ogliaruso and co-workers described DA cycloaddition between appropriate bis-cyclopentadienones and diphenylacetylene leading to bis(pentaphenyl)benzenes, in which two mirror fragments are connected with –CH_2_–, –*O*– or –*S*– linkers (Scheme 8) [64].

**Scheme 9:**
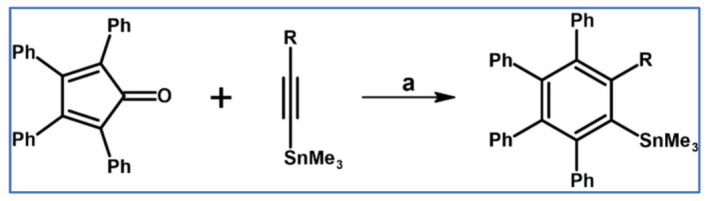
Ethynylthin derivatives in the DA cycloaddition with CO extrusion [65]. Reagents and conditions: R = Me, Ph, SnMe_3_; a = xylene, rfx, up to 147 h, up to 90% isolated yield.

Ethynyltin derivatives were also applied by Seyferth et al., as dienophiles in the syntheses of aryl-trimethyltin derivatives via DA cycloaddition with tetraphenylcyclopentadienone (Scheme 9) [65].

**Scheme 10:**
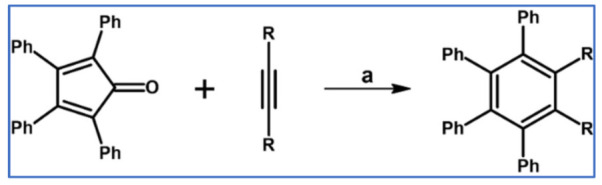
Synthesis of hexaphenylbenzene: DA cycloaddition and diphenylacetylene and tetraphenylcyclopentadienone as partners [59]. Reagents and conditions: a = benzophenone, rfx, 1 h, Y = 85% (for R = Ph); a = 1,2-dichlorobenzene, rfx, ~5 min, Y = 89% (for R = COOMe).

A report from Fieser and Haddadin showed that high yields and short reaction times were reached when cycloaddition leading to hexaphenylbenzene or tetraphenylbenzenedicarboxylate was realized in the boiling 1,2-dichlorobenzene or benzophenone (Scheme 10) [59].

It is worth noting that acetylenedicarboxylate is much more reactive than diphenylacetylene, which in our opinion is the result of electronic and steric effects. Namely, LUMO for COOMe is located lower, but steric hindrance is higher for Ph than COOMe. Analogically, we observed that the influence of electron and steric factors are quite similar to the DA cycloaddition to the perylene bay region [54,55].

**Scheme 11:**
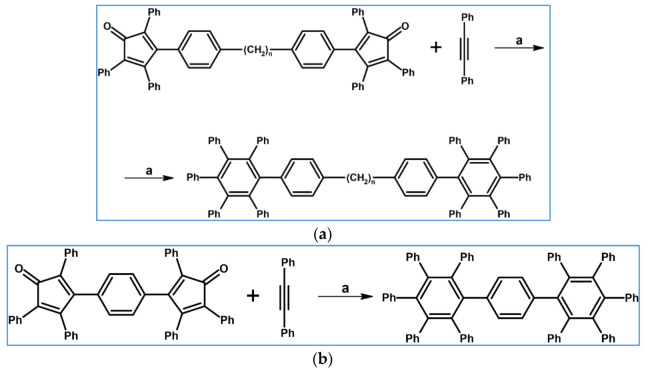
DA cycloaddition with CO extrusion for the synthesis of HPBs—examples [66]. Reagents and conditions: (**a**,**b**) n = 1–6; a = 280 °C (acetylene) or 315 °C (diacetylene), 1 h (acetylene) or 2 h (diacetylene).

Several derivatives of HPB (hexaphenylbenzene) were obtained via DA cycloaddition of 1,2-diphenylacetylene, 1,4-diphenybuta-1,3-diyne, and various cyclopentadienones (Scheme 11) as presented in a publication by Ogliaruso and Becker [66].

**Scheme 12:**
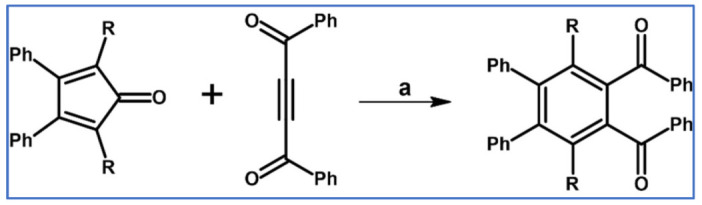
Synthesis of 3,6-dialkyl-4,5-diphenyl-1,2-diacetylbenzenes via DA cycloaddition with CO extrusion using cyclopentadienones and diacetylacetylene as diene and dienophile, respectively [67]. Reagents and conditions: a = *o*-dichlorobenzene, rfx, Y = 93% (for R = Me), Y = 90% (for R = Et) and Y = 79% (for R = *n*-Pr).

In 1966, Miller reported obtaining 1,2-di(benzoyl)benzene derivatives via DA cycloaddition of appropriately substituted cyclopentadienones and 1,2-di(benzoyl)acetylene (Scheme 12) [67].

**Scheme 13:**
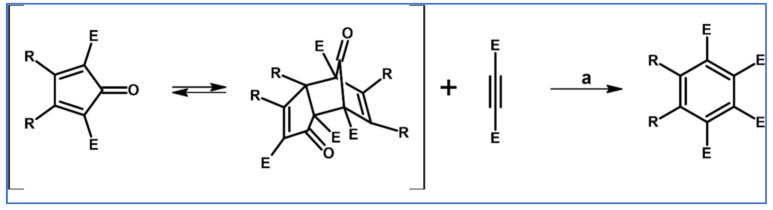
Dimer-monomer equilibrium and DA cycloaddition between monomer and dienophile [68]. Reagents and conditions: R = Me, Ph or COOMe; E = COOMe; a = PhMe, rfx, yields were not given.

The article by Cookson et al., from 1967 points that some cyclopentadienones undergo dimerization. However, due to dimer–monomer equilibrium, the reaction with dienophile (MeOOCC≡CCOOMe) present in the reaction mixture is possible and proceeds. Finally, the expected DA cycloaddition product is created (Scheme 13) [68].

**Scheme 14:**
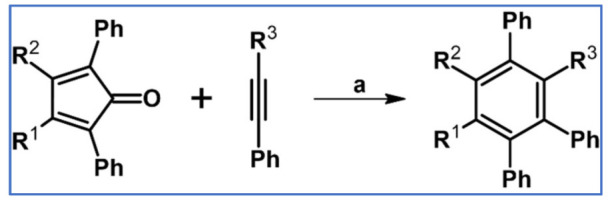
Syntheses of poly aryl-substituted benzenes, diphenyl ethers, triphenylenes, and acenaphthenes via DA cycloaddition with CO extrusion [69]. Reagents and conditions: (a) R^1^, R^2^, R^3^ = 2-Py, 2-Py, Ph; (b) R^1^, R^2^, R^3^ = Ph, 2-Py, Ph; (c) R^1^, R^2^, R^3^ = 2-Py, 2-Py, 2-Py; and others; a = benzene or mixture of benzene and EtOH, up to 200 °C, up to 82% yield.

Several poly-substituted benzenes, diphenyl ethers, triphenylenes, and acenaphthene were synthesized by Bergamnn and Paul in 1967. The synthesis involving DA cycloaddition and corresponding dienes and dienophiles is presented in Scheme 14 [69]. The role of dienes was played by derivatives of cyclopentadienes, including the ones containing one or two dienone motifs. Moreover, dienophiles possessed one or two triple bonds.

**Scheme 15:**
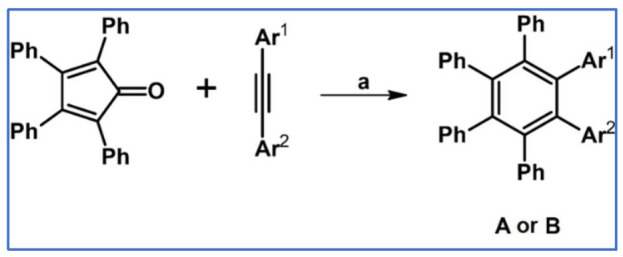
Synthesis of hexaarylbenzenes: DA cycloaddition involving tetraphenylcyclopentanedione and disubstituted acetylenes [70]. Reagents and conditions: **A**: Ar^1^ = Ar^2^ = C_6_F_5_; **B**: Ar^1^ = Ph, Ar^2^ = C_6_F_5_; a = heating, Y = 60% (both for **A** and **B**).

The same year, Filler and Heffern developed the synthesis of two derivatives of diphenylacetylene, namely C_6_H_5_C≡CC_6_F_5_ and C_6_F_5_C≡CC_6_F_5_ [70]. Next, the acetylene derivatives were used to synthesize hexaarylbenzenes via DA cycloaddition (with CO extrusion) with tetraphenylcyclopentadienone as diene (Scheme 15) [70].

**Scheme 16:**
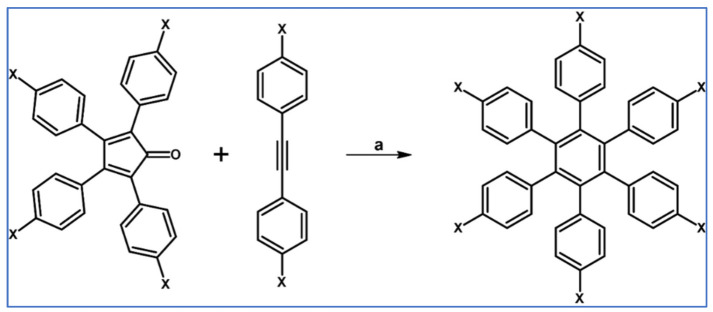
DA cycloaddition with CO extrusion involved in the synthetic route leading to symmetrically substituted hexaarylbenzenes [71]. Reagents and conditions: a = neat, heating; (a) Y = 80% (for X = Br); (b) Y= 75% (Me); (c) Y = 77% (for X = OMe).

The following year, symmetrically substituted hexaarylbenzenes were obtained via DA cycloaddition of tetraarylcyclopentadienones and disubstituted acetylenes bearing the same aryl groups (Scheme 16) [71].

**Scheme 17:**
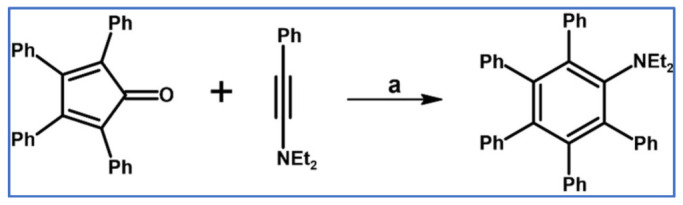
DA cycloaddition with CO extrusion: synthesis of *N*,*N*-diethylaminopetaphenylbenzene [72]. Reagents and conditions: a = diglyme, 180 °C, 12 h, Y = 7%.

Additionally, in 1968, Kuehne and Sheeran, in the article devoted to ynamines reactivity, described DA cycloaddition between tetraphenylcyclopentadienone and *N*,*N*-diethyl(2-phenylethynyl)amine leading to the aniline derivative, but with low yield [72] (Scheme 17).

**Scheme 18:**
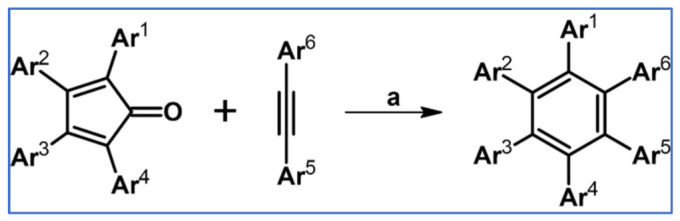
Preparation of hexaarylbenzenes via DA cycloaddition with CO extrusion [73]. Reagents and conditions: (a) Ar^1^, Ar^2^, Ar^3^, Ar^4^, Ar^5^, Ar^6^ = Ph, Ph, Ph, Ph, *p*-tol, *p*-tol; (b) Ar^1^, Ar^2^, Ar^3^, Ar^4^, Ar^5^, Ar^6^ = Ph, *p*-tol, Ph, Ph, Ph, *p*-tol; (c) Ar^1^, Ar^2^, Ar^3^, Ar^4^, Ar^5^, Ar^6^ = Ph, Ph, Ph, *p*-Me_2_NPh, *p*-Me_2_NPh, Ph; and others; a = neat, heating, up to 84% yield.

As reported in a paper from 1969, cyclopentadienones and disubstituted acetylenes, bearing aryl groups in various positions, were used in the syntheses of several hexasubstituted benzenes (Scheme 18) [73]. The syntheses (18 reactions) presented in the scheme below were conducted with yield ranging from 72 to 84%.

**Scheme 19:**
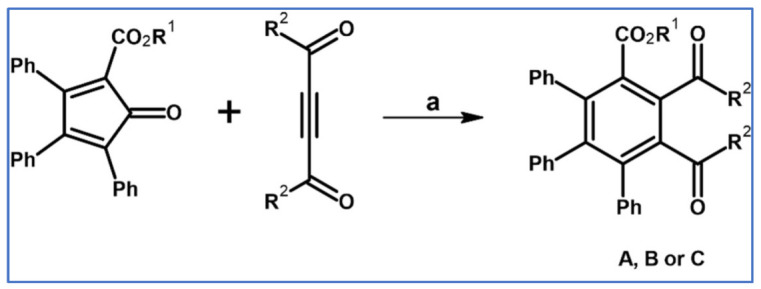
DA cycloaddition with CO extrusion for the synthesis of fully substituted benzenecarboxylate and benzene tricarboxylate [74]. Reagents and conditions: **A**: R^1^ = Me, R^2^ = OMe; **B**: R^1^ = Me, R^2^ = Ph; **C**: R^1^ = Et, R^2^ = OMe a = xylene, rfx, 2 h, Y = 70% (for **A**), Y = 45% (for **B**), and Y = 77% (for **C**).

Ried and Wagner described the syntheses of triphenyl(carboxyalkyl)cyclopentadienones and their application for the DA cycloaddition with acetylene dicarboxylate or 1,4-diphenyl-2-butyn-1,3-dione (dicarboxymethyl- or dibenzoyl acetylene) as dienophiles [74] (Scheme 19).

It is worth noting that the reaction temperature is relatively low, which is, in our opinion, the result of EWG group presence in both diene and dienophiles.

**Scheme 20:**
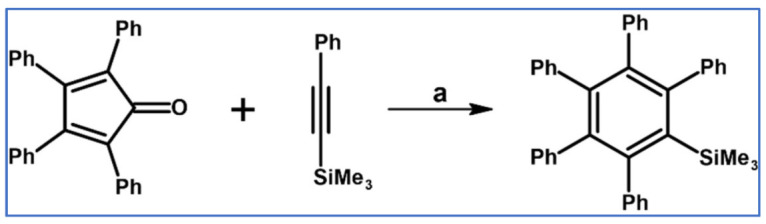
Synthesis of pentaphenyl(trimethylsilyl)benzene via DA cycloaddition with CO extrusion [75]. Reagents and conditions: a = PhMe, 225 °C, 48 h, Y = 93%.

According to Freeburger and Spialter, pentaphenyl(trimethylsilyl)benzene was obtained with high yield via DA cycloaddition of appropriate silane and tetraphenylcyclopentadienone (Scheme 20) [75].

It should be emphasized that DA reactions of vinyl-, allyl-, and phenylethynylsilanes with tetraphenylcyclopentadienone were slower than that of the carbon analogues. Moreover, only phenylethynyltrimethylsilane underwent cycloaddition of all the examined phenylethynylsilanes.

**Scheme 21:**
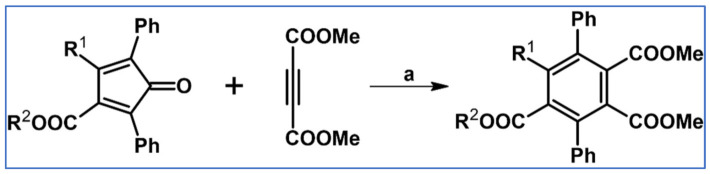
Benzenetricarboxylates: preparation via DA cycloaddition [76]. Reagents and conditions: a = 150 °C, Y = 74% (for R^1^, R^2^ = Ph, Me), Y = 84% (for R^1^, R^2^ = Bn, Et), Y = 78% (for R^1^, R^2^ = Ph, CH_2_CH_2_OC(O)Me).

In 1970, Ried and Olszewski published a paper about 1,2,4-benzenetricarboxylates synthesized via DA cycloaddition with CO extrusion, corresponding cyclopentadienones and dimethyl acetylenedicarboxylate (Scheme 21) [76].

**Scheme 22:**
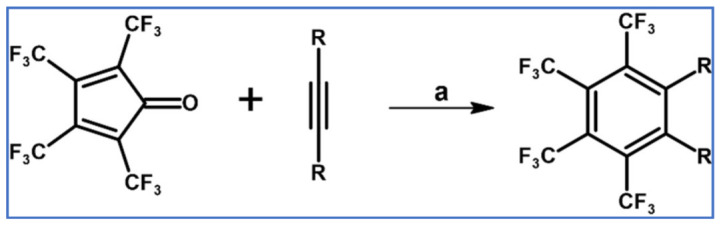
Tetra(trifluoromethyl)cyclopentadienone: very reactive diene in DA cycloaddition with CO extrusion [77]. Reagents and conditions: R = H, Me, COOMe; a = from rt to 120 °C (conditions were not precisely given); yields were not given.

In 1975, Szilagyi et al., applied tetra(trifluoromethyl)cyclopentadienone, which turned out to be thermally very stable and highly reactive, as a diene in various cycloadditions. DA cycloaddition with acetylenes as dienophiles was one of the reactions under examination (Scheme 22) [77].

It is worth stressing that the reaction with gaseous acetylene proceeded effectively at room temperature and with acetylenedicarboxylate at 120 °C. Moreover, cyclopentadienones, especially tetra(trifluoromethyl)- one, owe their significant reactivity to a low-lying LUMO and the consequently narrow gap between FMOs.

**Scheme 23:**
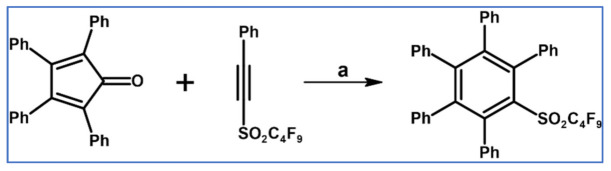
DA cycloaddition with CO extrusion: synthesis of nonafluorobutyl-pentaphenyl(phenyl) sulfone [78]. Reagents and conditions: a = PhMe, rfx, Y = 18%.

Perfluorobutyl-pentaphenyl(phenyl) sulfone was obtained by Hanack and Massa using DA cycloaddition with CO extrusion, involving ethynylsulfone and tetraphenyldcyclopentadienone as dienophile and diene, respectively (Scheme 23) [78].

**Scheme 24:**
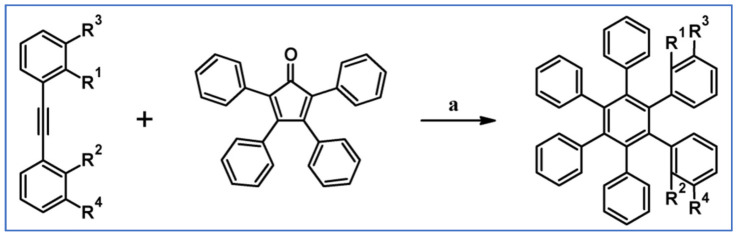
Synthesis of xexasubstituted benzenes via DA cycloaddition of tetraphenylcyclopentadienone and phenyl-substituted diphenylacetylenes [79]. Reagents and conditions: (a) R^1^ = OMe. R^2^ = Me, R^3^ = R^4^ = H; (b) R^1^ = R^2^ = Me, R^3^ = R^4^ = H; (c) R^1^ = R^2^ = H; R^3^ = R^4^ = Me; a = Ph_2_CO, rfx, 3 h; up to 79% yields.

In 1977, Gust published a paper about DA cycloaddition of various acetylenes and tetraphenylcyclopentadienones applied in the synthesis of tetraarylbenzenes (Scheme 24) [79].

It was observed that ortho- and meta-substituted HAB are characterized by relatively high rotational barriers (around the single bond between central and peripheral aryl rings). This rotation limitation causes complex stereoisomerism and stereoisomerization.

**Scheme 25:**
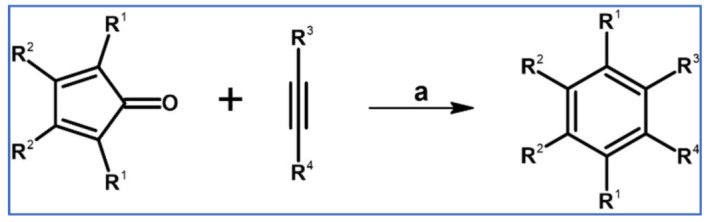
Synthesis of penta- and hexaarylsubstituted benzenes via DA cycloaddition with CO extrusion: cyclopentadienones and acetylenes as partners [80]. Reagents and conditions: (a) R^1^ = Ph, R^2^ = 3-methylphenyl, R^3^ = Ph, R^4^ = H, a = excess of dienophile, rfx, Y = 33%; (b) R^1^ = Ph, R^2^ = 3-methylphenyl, R^3^ = 3-methoxyphenyl, R^4^ = H, a = excess of dienophile, rfx, Y = 67%; (c) R^1^ = Ph, R^2^ = 3-methoxyphenyl, R^3^ = R^4^ = Ph, a = excess of dienophile, rfx, Y = 75%; (d) R^1^ = 3-methylphenyl, R^2^ = Ph, R^3^ = R^4^ = 2-methoxyphenyl, a = benzophenone, rfx, Y = 68%; (e) R^1^ = Ph, R^2^ = 3-methoxyphenyl, R^3^ = R^4^ = 3-methylphenyl, a = excess of dienophile, rfx, Y = 22%.

In the article addressing diastereoismerization mechanism of hexaarylbenzenes, Gust and Patton described the syntheses of selected penta- and hexaarylbenzenes via DA cycloaddition (Scheme 25) [80].

It was measured that free energies of activation for isomerization vary from about 33 kcal/mol for rotations of rings with an ortho-methoxy group to about 17 kcal/mol for rotations of rings having meta- substituents.

**Scheme 26:**
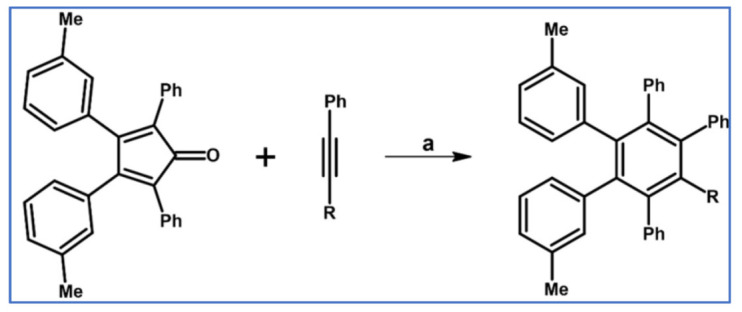
Syntheses of hexasubstituted benzenes: DA cycloaddition in the presence of appropriately substituted cyclopentadienone and Ph–C≡C–R type dienophiles [81]. Reagents and conditions: a = excess of dienophile, rfx, Y = 39% (for R = Me); a = excess of dienophile, rfx, triglyme, 2 h, Y = 22% (for R = Et); a = triglyme, rfx, 9 h, Y = 47% (for R = *i*-Pr); a = excess of dienophile, rfx, Y = 25% (for R = –CH(OH)Me); a = excess of dienophile, rfx, 56 h, Y = 31% (for R = *t*-Bu).

According to a report from Patton, a series of pentaaryl-alkyl-benzenes were obtained via DA cycloaddition with CO extrusion, and the free energies of activation (ranging from 15.5 to 18.7 kcal/mol) for their rotational stereoisomerization were measured (Scheme 26) [81].

Moreover, steric repulsion coming from rotating substituents attached to the central ring are transmitted remotely.

**Scheme 27:**
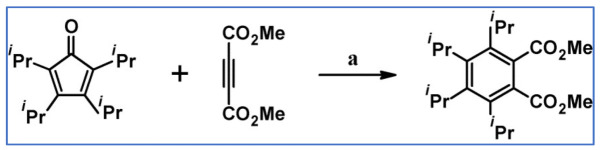
Syntheses of dimethyl 3,4,5,6-tetraisopropylbenzene-1,2- dicarboxylate via DA cycloaddition of appropriate diene and acetylene [82]. Reagents and conditions: a = benzene, rfx, 24 h, Y = 67%.

Dimethyl 3,4,5,6-tetraisopropylbenzene-1,2-dicarboxylate was prepared via DA cycloaddition of tetraisopropylcyclopentadienone and acetylene dicarboxylate (Scheme 27), as Weissensteiner et al., stated in their paper from 1985 [82].

One should pay attention to the low temperature of this cycloaddition, whereas, in standard protocol, refluxing toluene, xylene, or most often diphenyl ether are typically used.

**Scheme 28:**
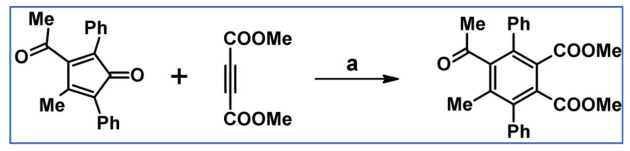
DA cycloaddition with CO extrusion: synthesis of hexasubstituted benzene-1,2-dicarboxylate [83]. Reagents and conditions: a = benzene, rt, 20 h, Y = 28%.

In 1988, Yoshida and colleagues synthesized hexasubstituted 1,2-benzene dicarboxylate via DA cycloaddition involving dimethyl acetylene-1,2-dicarboxylate and 3-acetyl-4-methyl-2,5-diphenylcyclopenta-2,4-dienone (Scheme 28) [83].

**Scheme 29:**
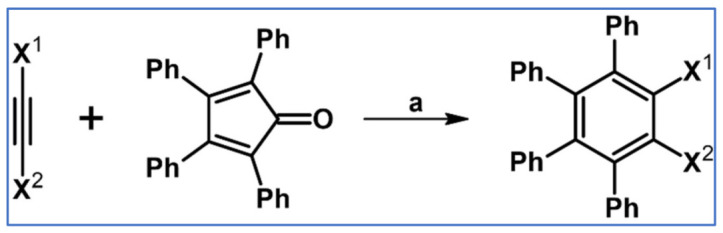
DA cycloaddition involving acetylenes possessing SCF_3_ motifs [84]. Reagents and conditions: a = *o*-dichlorobenzene, rfx, Y = 79% (for X^1^ = X^2^ = SCF_3_), Y = 83% (for X^1^ = H, X^2^ = CH_2_SCF_3_).

The same year, Haas and Krachter described the atypical dienophiles containing SCF_3_ motifs that underwent DA cycloaddition with tetraphenylcyclopentadienone. That reaction resulted in hexasubstituted benzenes, including derivative possessing two SCF_3_ motifs (Scheme 29) [84].

**Scheme 30:**
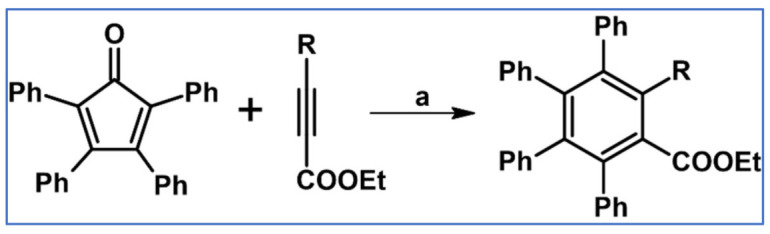
DA cycloaddition with CO extrusion involved in the syntheses of ethyl 2-(perfluoroalkyl)-3,4,5,6-tetraphenylbenzoate [85]. Reagents and conditions: R = CF_3_, C_5_F_11_, C_6_H_13_, C_7_F_15_; a = xylene, up to 150 °C, up to 4 d, up to 95% yield.

A series of ethyl perfluoroalkyl-tetraphenylbenzoate was obtained via DA cycloaddition between tetraphenylcyclopentadienone and ethyl propiolate bearing perfluoroalkyl groups (Scheme 30) as Nezis and co-workers stated in their paper from 1992 [85].

**Scheme 31:**
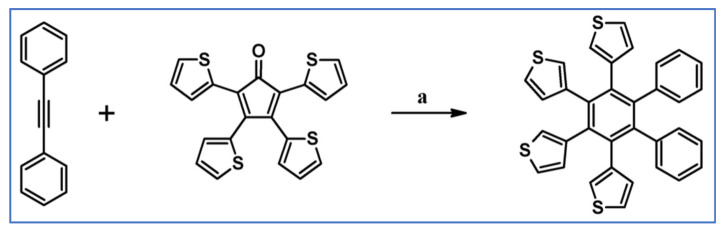
Synthesis of tetra(2-thienyl)-di-phenylbenzene via DA cycloaddition with CO extrusion [86]. Reagents and conditions: a = 190 °C, 1 h, Y = 73%.

Preparation of tetra(2-thienyl)-di-phenylbenzene via cycloaddition of tetra(2-thienyl)cyclopentadienone and diphenylacetylene was described by Kawase et al., (Scheme 31) [86].

**Scheme 32:**
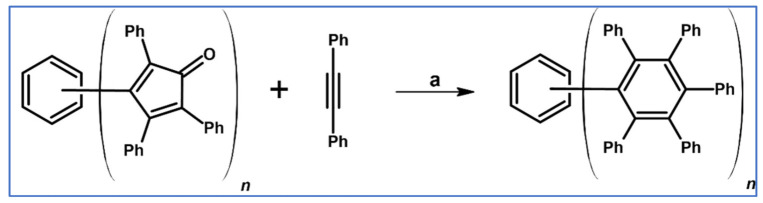
Syntheses of “Albatrossenes”—an example [87]. Reagents and conditions: n = 2 or 3; a = 300 °C, 2 h, Y = 86% (for n = 2), Y = 35% (for n = 3).

C_2_-symmetric polyphenylenes, dubbed ‘Albatrossenes’ by Tong et al., due to their shape, were obtained via DA cycloaddition between dienes possessing di- or tri-cyclopentadienone core and acetylenes (Scheme 32) [87]. Such kinds of PAHs were also obtained via cycloaddition with benzyne as a dienophile (see Section 2.2).

For all the obtained PAHs, their molecular structures were thoroughly analyzed.

**Scheme 33:**
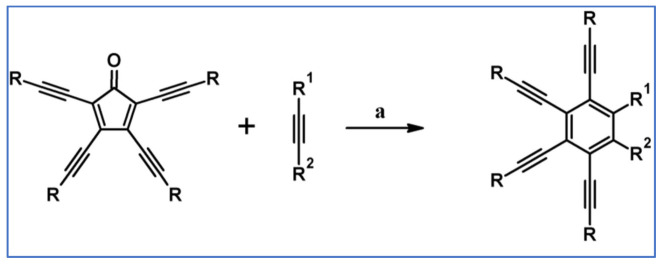
Cycloaddition of tetra(R–C≡C–)substituted cyclopentadienones and symmetrically or non-symmetrically substituted acetylenes [88]. Reagents and conditions: (a) R = TIPS, R^1^ = R^2^ = Et, Ph, COOMe, –C≡C–TMS or other R^1^, R^2^; (b) R = *t*-Bu, R^1^ = R^2^ = –C≡C–TMS or other R^1^, R^2^; (c) R=C≡C–TIPS, R^1^ = R^2^ = –C≡C–TMS; a = PhMe or xylene, rfx, 2–24 h, up to 92% yield.

Preparation of a series of hexasubstituted benzene bearing from four to six –C≡C–R (R—see Scheme 33) moieties via cycloaddition of appropriately substituted cyclopentadienones and acetylenes was reported by Tovar et al., the same year [88].

**Scheme 34:**
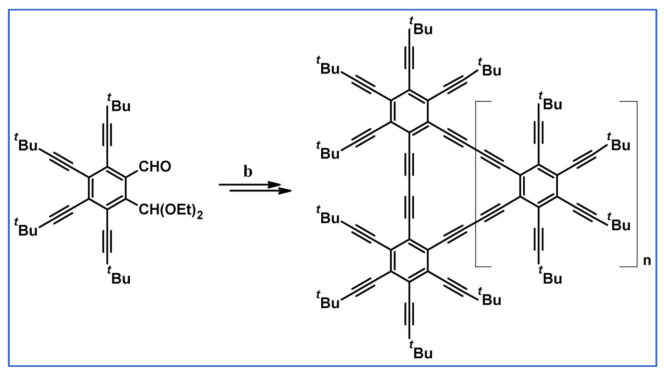
Synthesis of an octaalkynyldibenzooctadehydro[12]-annulene [88]. Reagents and conditions: b = three steps; n = 0, 1, 2, and 3.

One of the products in the scheme visible above was applied to the synthesis of octaalkynyldibenzooctadehydro[12]-annulene (Scheme 34) [88].

One should emphasize that the products of the reactions mentioned above can be treated as nanographenes.

**Scheme 35:**
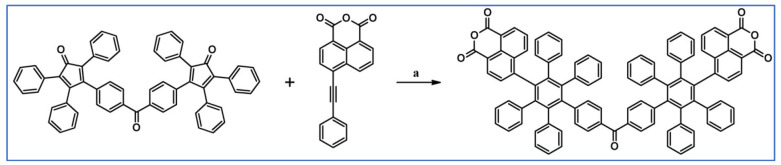
Synthesis of polyphenylene containing naphthalene anhydride motifs and –C(O)– linker via DA cycloaddition of appropriate diene and acetylene [89]. Reagents and conditions: a = 1,2,4-trichlorobenzene, rfx, 10 h, Y = 91%.

It was shown by Keshtov et al., that DA cycloaddition between acetylene containing naphthalene anhydride motif and bis-cyclopentadienone (two tetraphenylcyclopentadienone motifs are connected by –C(O)– linker) is possible and effective (Scheme 35) [89].

According to us, such compounds can play the role of attractive precursors of A–D–A systems, but after Scholl reaction.

**Scheme 36:**
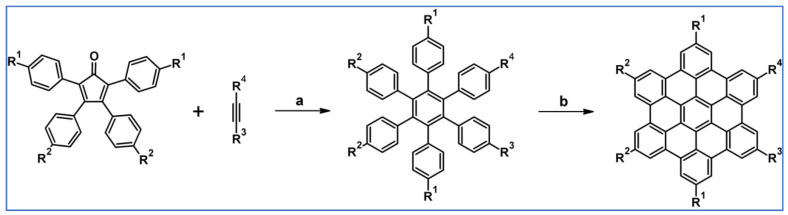
Syntheses of HBCs equipped with solubilizing, donor and acceptor groups via DA cycloaddition with CO extrusion and further transformation [90]. Reagents and conditions: (a) R^1^ = R^2^ = R^4^ = *n*-C_12_H_25_; R^3^ = Br; (b) R^1^ = R^2^ = *n*-C_12_H_25_; R^3^ = R^4^ = Br; (c) R^1^ = Br; R^2^ = R^3^ = R^4^ = *n*-C_12_H_25;_ a = Ph_2_O, rfx, yield up to 76%; b = Scholl reaction conditions, yield up to 93%.

The next year Ito et al., prepared functionalized derivatives of HBC via tandem DA cycloaddition-Scholl dehydrocondensation, using acetylenes and cyclopentadienones containing bromine atoms and solubilizing groups (Scheme 36) [90].

Ultimately, bromine atoms present in the HBC structure were exchanged for acceptor or donor groups, namely alkoxy, amino and ester, and cyano, respectively. While studying the self-assembly of obtained HBC derivatives, the existence of columnar mesophases was revealed.

**Scheme 37:**
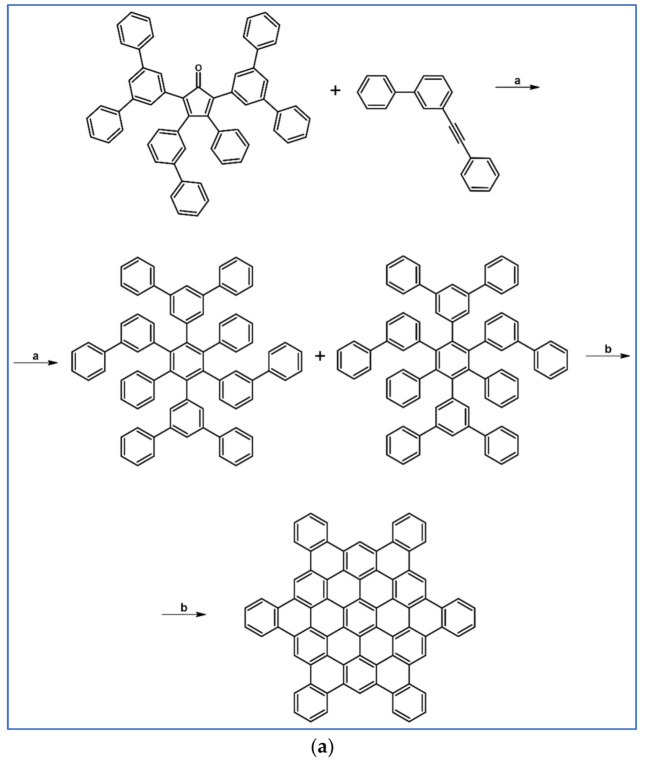

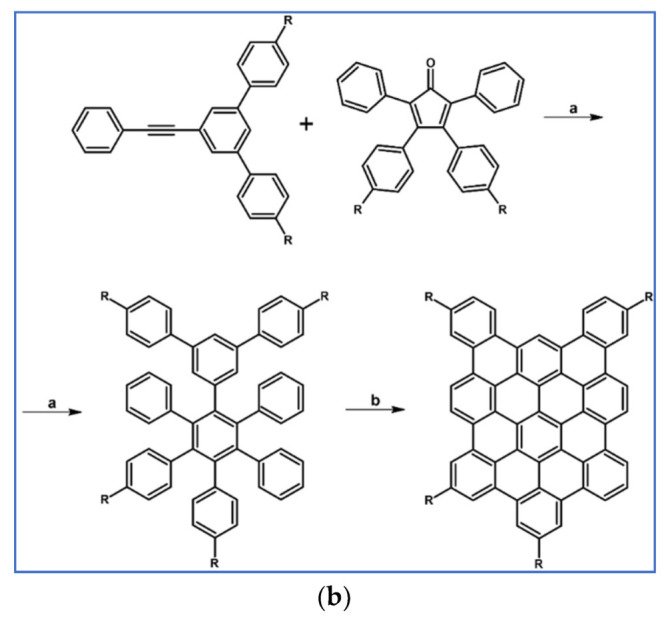
DA cycloaddition with CO extrusion for the synthesis of HPBs and nanographenes—examples [91]. Reagents and conditions: (**a**) a = Ph_2_O, rfx, 2 h; Y = 68%; b = next step (Scholl reaction); (**b**) R = H or C_12_H_25_; a = Ph_2_O, rfx, 16 h (R = H), 18 h (R = C_12_H_25_); Y = 77% (for R = H) or 92% (for R = C_12_H_25_); b = next step (Scholl reaction).

Several derivatives of HPB (hexaphenylbenzene) were obtained by Doltz’s research group via *Co*-catalyzed disubstituted acetylenes cyclotrimerization or DA cycloaddition of 1,2-diarylacetylene, and various cyclopentadienones [91]. The syntheses involving DA cycloaddition with CO extrusion are shown in Scheme 37 [91]. In the scheme, final structures, namely nanographenes containing up to 78 aromatic carbon atoms, obtained from precursors via Scholl reaction are also presented.

Importantly, due to the insufficient solubility of extended PAHs, characterization was achieved by laser desorption/ionization time-of-flight mass spectrometry and UV/visible spectroscopy of thin films [91].

**Scheme 38:**
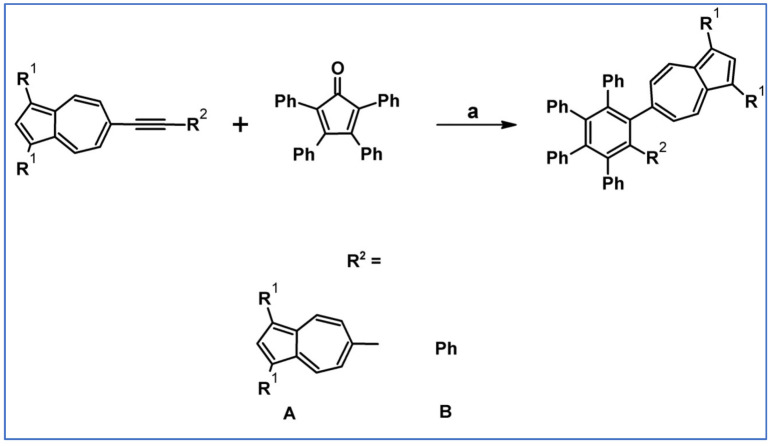
DA cycloaddition with CO extrusion involved in the syntheses of teraphenyl- and pentaphenylbenzenes bearing one or two azulenyl-moieties [92]. Reagents and conditions: a = Ph_2_O, 160 °C, 24–48 h, under Ar atmosphere; compounds **A**: R^2^ = A, for R^1^ = H: Y = 13% and for R^1^ = COOMe, Y= 90%; compounds **B**: R^2^ = B, for R^1^ = H: Y= 32% and for R^1^ = COOMe, Y= 93%.

1,2-Di(6-azulenyl)tetraphenylbenzenes and (6-azulenyl)pentaphenylbenzenes were synthesized by DA reactions of di(6-azulenyl)acetylenes and 6-(phenylethynyl)azulenes with tetraphenylcyclopentadienone (Scheme 38) as described by Ito and colleagues [92].

What is drawing the attention, in this case, is the significantly higher reaction yield for COOMe derivatives than for those with Ph substituent. As mentioned before, in our opinion, this is due to a noticeable decrease in LUMO activation energy of the dienophile (influence of COOMe as EWG). Moreover, in the commented work, the electrochemical properties of synthesized compounds were examined by cyclic voltammetry [92].

**Scheme 39:**
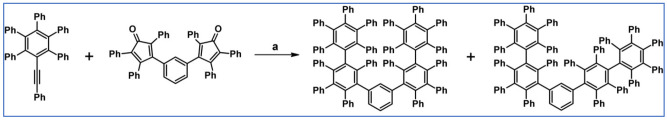
Synthesis of 1,3-bis(nonaphenyl-3-biphenyl)benzene using DA cycloaddition with CO extrusion [93]. Reagents and conditions: a = phenanthrene, 315 °C, 2 h, Y = 6% (both isomers in total).

Pascal et al., reported in 2001 DA cycloaddition between appropriate diene and dienophile. This attempt allowed them to obtain, however, with low yield (despite the drastic conditions), two isomers of 1,3-bis(nonaphenyl-3-biphenyl)benzene (Scheme 39) [93].

**Scheme 40:**
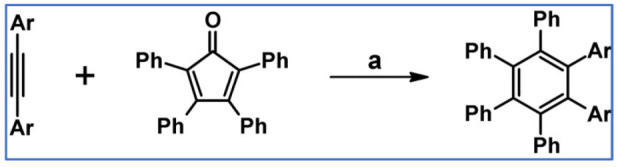
Preparation of 1,2-bis(2-methoxy-1-naphthyl)-3,4,5,6-tetraphenylbenzene via DA cycloaddition with CO extrusion [94]. Reagents and conditions: Ar = 2-methoxynaphtalen-1yl; a = Ph_2_O, rfx, 7 h, Y= 25%.

DA cycloaddition was involved in the synthetic procedure leading to hexaaryl substituted benzene (Scheme 40) [94].

**Scheme 41:**
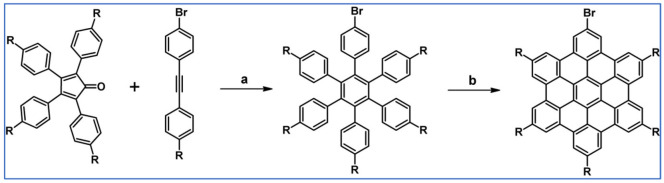
Synthesis of the HBC derivative with the aid of DA cycloaddition [95]. Reagents and conditions: R = 3,7-dimethyloctan-1-yl; a = Ph_2_O, rfx, 5 h, under Ar atmosphere; Y = 53%; b = next step (Scholl reaction); Y = 80%.

Thermotropic liquid crystalline derivatives of HBC substituted at the periphery by up to six chiral or racemic branched groups, i.e., 3,7-dimethyloctan-1-yl, were obtained via DA cycloaddition or catalytic cyclotrimerization-cyclodehydrocondensation. The synthetic way developed by Fechtenkötter and co-workers engaging cycloaddition is presented on the scheme below (Scheme 41) [95].

The presence of bromine atom and high solubility in typical organic solvents make this compound an attractive building block for further functionalization.

**Scheme 42:**
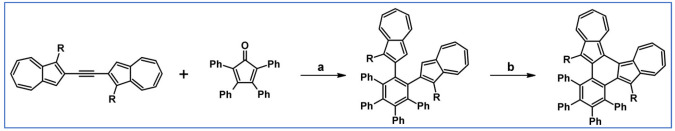
DA cycloaddition followed by dehydrocondensation as crucial steps in the syntheses of diazuleno[2,1-*a*:1,2-*c*]naphthalenes [96]. Reagents and conditions: R = H, COOMe, a = Ph_2_O, heating, up to 48 h, up to 51% yield; b = next step.

The DA cycloaddition with CO extrusion of 1,2-bis(2-azulenyl)acetylenes with tetraphenylcyclopentadienone afforded 7,8,9,10-tetraphenyldiazuleno[2,1-*a*:1,2-*c*]naphthalene via dehydrocondensation of the presumed 1,2-bis(2-azulenyl)benzene derivative. Ito and colleagues stated in their paper that this synthesis can be either carried out in a one-pot attempt or two-step procedure. In the first instance, oxygen acts as the oxidizer, while in the second—FeCl_3_ (Scheme 42) [96].

The redox behaviour of these diazuleno[2,1-*a*:1,2-*c*]naphthalenes was examined by cyclic voltammetry (CV).

**Scheme 43:**
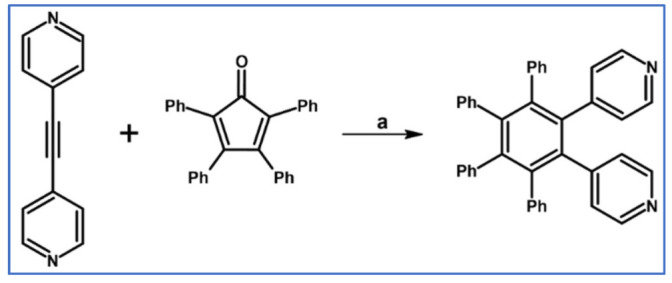
Synthesis of 1,2,3,4-tetraphenyl-5,6-bis(4-pyridyl)benzene via DA cycloaddition with CO extrusion [97]. Reagents and conditions: a = heating.

A bridging ligand equipped with two 4-pyridyl motifs in the 1,2-position was obtained by Steel and Webb using tetraphenylcyclopentadienone, 1,2-bis(4-pyridyl)acetylene and DA cycloaddition with CO extrusion (Scheme 43) [97].

The above-presented ligand was used for the obtaining of a polymeric complex with AgCOOMe.

**Scheme 44:**
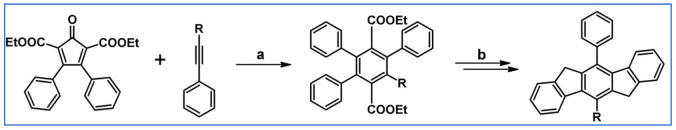
Mono- and diphenylindenofluorenes: DA cycloaddition as a crucial, synthetic step [98]. Reagents and conditions: R = H, Ph; a = dichlorobenzene, rfx, 12 h, Y = 90%; b = next steps.

According to a report from Merlet, Birau, and Wang from 2002, DA cycloaddition with CO extrusion was crucial in the synthetic route leading to indenofluorenes as building blocks for electronic and optoelectronic materials (Scheme 44) [98]. The unsubstituted system was also synthesized, but in other ways than through DA cycloaddition.

These indenofluorenes, including unsubstituted ones, are large band-gap compounds with high photoluminescence yield (73–78%) in solution and, thus, exhibit great potential as optoelectronic materials.

**Scheme 45:**
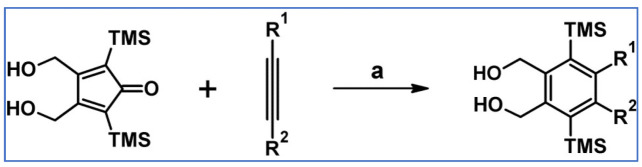
Synthesis of highly functionalized biaryl involving DA cycloaddition with CO extrusion [99]. Reagents and conditions: (a) R^1^ = R^2^ = COOMe, a = PhMe, 110 °C, 10 h, Y = 95%; (b) R^1^ = Ph, R^2^ = COOMe, a = decaline, 210 °C, 8 h, Y = 25%; (c) R^1^ = Ph, R^2^ = CN, a = mesitylene, 140 °C, 8 h, Y = 40%; (d) R^1^ = Ph, R^2^ = SO_2_tol-*p*, a = mesitylene, 140 °C, 8 h, Y = 75%; (e) R^1^ = *p*-MeO_2_CPh, R^2^ = CN, a = mesitylene, 140 °C, 8 h, Y = 42%; (f) R^1^ = *o*-MeO_2_CPh, R^2^ = CN, a = mesitylene, 140 °C, 8 h, Y = 45%.

Highly functionalized biaryls were obtained via DA cycloaddition involving appropriately substituted cyclopentadienones and acetylenes (Scheme 45) [99].

Again, the highest reactivity of acetylene with two COOMe groups is worth noting. This is undoubtedly the effect of LUMO energy decrease [54,55].

**Scheme 46:**
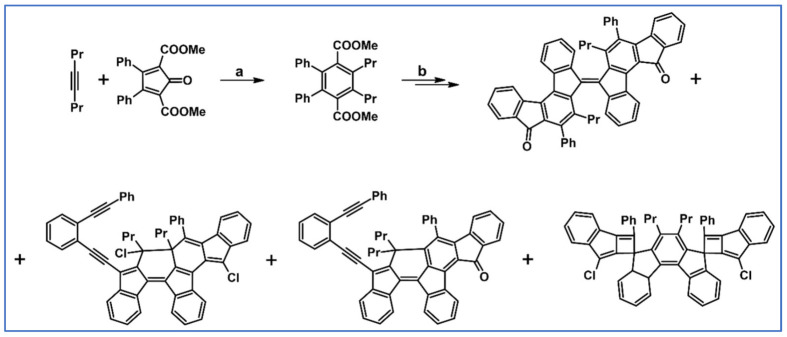
Synthesis of highly twisted compounds starting from DA cycloaddition of internal alkyne and appropriately substituted cyclopentadienones [100]. Reagents and conditions: a = 190 °C, 12 h, sealed tube; Y = 50%; b = next steps.

DA cycloaddition of internal alkyne and substituted cyclopentadienones were used by Yang, Petersen, and Wang for the synthesis of highly twisted 1,1-dialkyl-9,9-bifluorenylidenes (Scheme 46) [100].

**Scheme 47:**
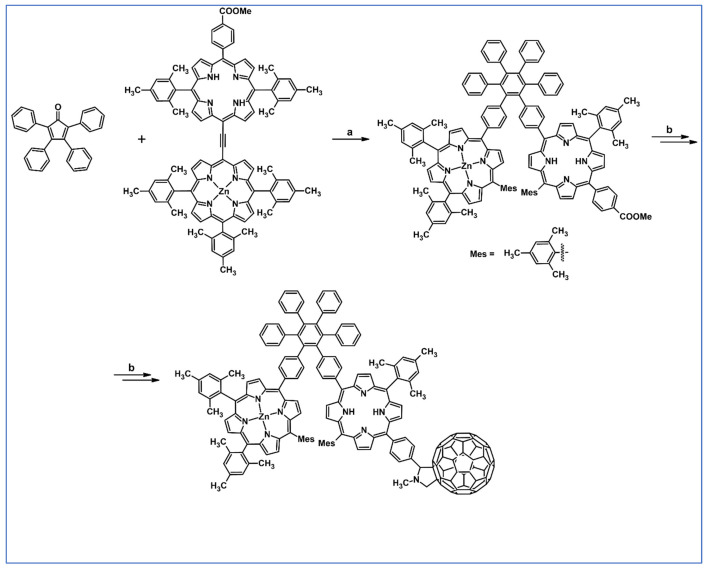
DA cycloaddition of tetraphenylcyclopentadienone and porphyrin-moieties bearing acetylene in the synthesis of zinc porphyrin (PZn)-free base porphyrin (P2H)-fullerene (C60) molecular triad [101]. Reagents and conditions: a = Ph_2_O, rfx, 5 h, Y = 74%; b = next steps.

As described by Liddell and co-workers in 2004, DA cycloaddition with CO extrusion was a crucial step in the multi-step synthetic route leading to the zinc porphyrin (PZn)-free base porphyrin (P2H)-fullerene (C60) molecular triad (Scheme 47) [101]. Spectroscopic investigations showed that the obtained nanomaterial can be attractive for designing supermolecular systems for energy and electron transfer.

**Scheme 48:**
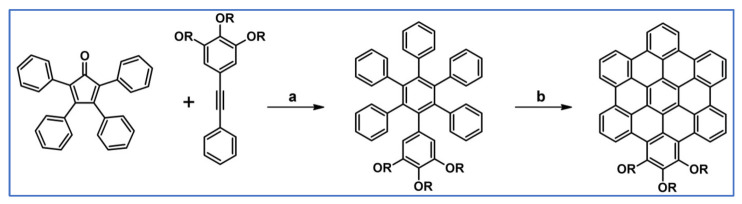
Unwrapped trialkoxy-HBC: syntheses via DA cycloaddition with CO extrusion followed by dehydrocondensation [102]. Reagents and conditions: R = *n*-C_12_H_25_; a = Ph_2_O, 6 h, rfx, Y = 90%; b = next step (Scholl reaction).

Wang et al., described the preparation of unwrapped hexa-peri-hexabenzocoronene derivative via DA cycloaddition followed by Scholl dehydrocondensation (Scheme 48) [102].

The obtained HBC derivative forms a stable columnar liquid crystalline mesophase with a practically accessible isotropization temperature. Moreover, it is characterized by good solution processability and long-range order, making this a kind of unwrapped columnar material a potentially active component in organic electronic devices.

**Scheme 49:**
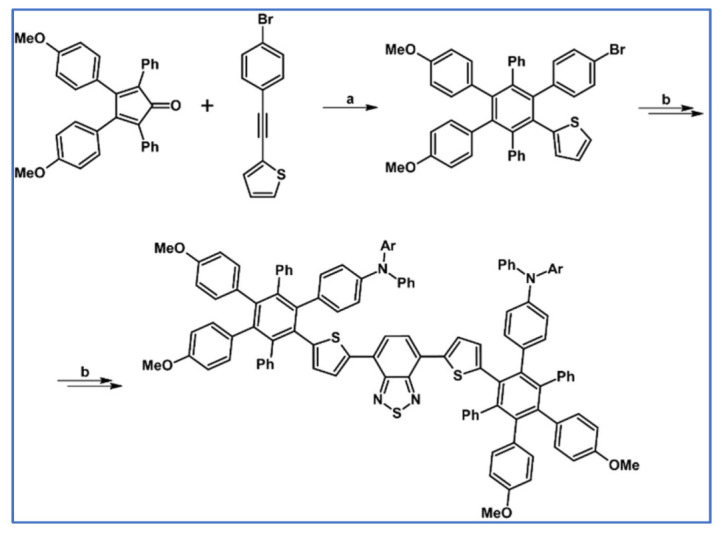
Syntheses of Ar_5_Ph-L-PhAr_5_ type nanometerials starting from DA cycloaddition with CO extrusion [103]. Reagents and conditions: Ar = 1-naphthy, 9-phenantryl, 1-pyrenyl; a = Ph_2_O, 250 °C, 12 h, Y = 88%; b = next steps.

DA cycloaddition with CO extrusion was applied by Thomas’ research group in the synthetic procedures leading to Ar_5_Ph-L-PhAr_5_ type and star-shaped hexarylbenzene derivatives (Scheme 49) [103].

The obtained compounds belonging to donor-acceptor-donor type nanomaterials are characterized by antenna effect, high glass transition temperature, and efficient electroluminescence.

**Scheme 50:**
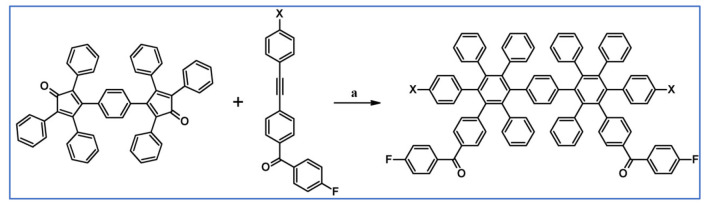
DA cycloaddition of acetylene and cyclopentadienone derivatives involved in the synthesis of polysubstituted hexaarylbenzenes [104]. Reagents and conditions: X = H or F; a = 1,2,4-trichlorobenzene, rfx, 40 h, Y = 89%.

According to a report from Keshtov and co-workers from 2005, hexaarylbenzene containing di- and tetrafluoroarenenyl-motifs were prepared by the DA reactions of appropriately substituted acetylene and cyclopentadienone, with CO extrusion (Scheme 50) [104].

**Scheme 51:**
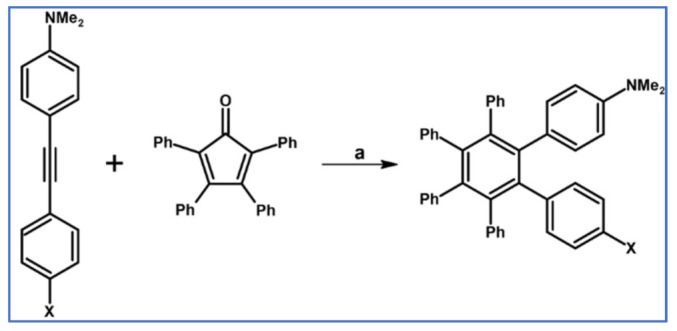
Syntheses of HBC derivatives equipped with one or two donor-groups via DA cycloaddition [105]. Reagents and conditions: a = 200 °C, 24 h, Y = 76% (for X = H) or 25% (for X = Nme_2_).

HPB bearing six, two, or one *p*-NMe_2_ substituents in the phenyl rings were obtained via Co-mediated appropriate acetylene cyclotrimerization or DA cycloaddition with CO extrusion, respectively (Scheme 51) as stated in the paper by Sun et al., [105].

Thoroidal through-space π-delocalization in the radical-cations electrochemically obtained from HPB possessing increasing number of donor groups was thoroughly analyzed involving spectroscopic, electrochemical, and theoretical methods.

**Scheme 52:**
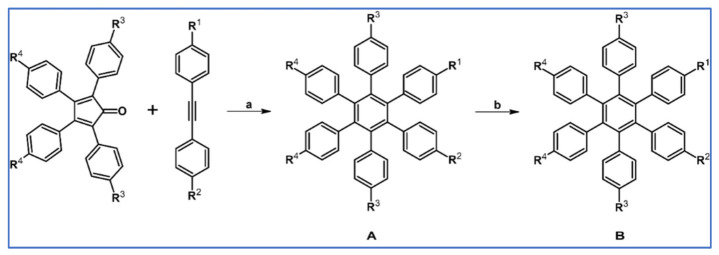
Preparation of HPB derivatives DA cycloaddition with CO extrusion followed by amination [106]. Reagents and conditions: R^1^, R^2^, R^3^, R^4^ (in substrates) = H, Br, *t*-Bu or OMe in various number and positions; in products bromine-atoms are replaced by *N*Ph(pyren-2-yl) groups. a = Ph_2_O, 200 °C; b = Pd-catalyzed amination.

Derivatives of HPB bearing *t*-Bu, OMe, and particularly *N*Ph(pyren-2-yl) substituents were synthesized by Thomas and colleagues via DA cycloaddition followed by Pd-catalyzed amination (Scheme 52) [106].

Due to the presence of *N*Ph(pyren-2-yl) motifs conjugated with HPB-core, the obtained nanomaterials were characterized with good thermal stability, reversible electrochemical red/ox properties, good hole-transporting capability, and efficient electroluminescence. Due to the advantages mentioned above, the commented nanomaterials can be applied in blue-emitting OLEDs.

**Scheme 53:**
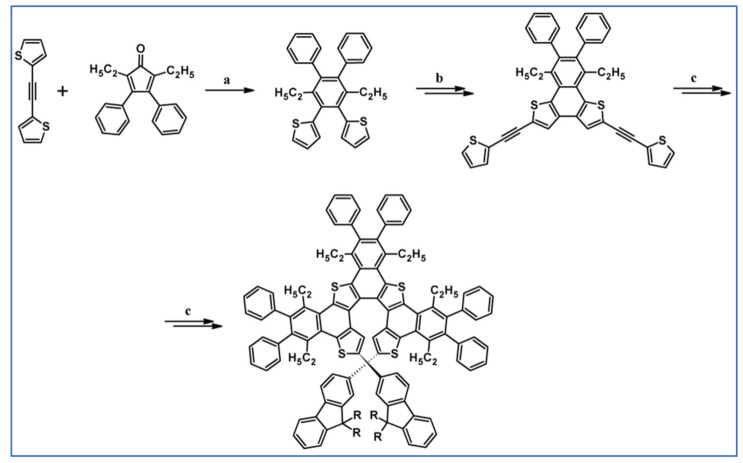
DA cycloaddition of 2-thienylethynyl containing precursor and tetrasubstituted cyclopentadienone in the synthesis of non-planar (helical) thiophene-based polycyclic aromatics [107]. Reagents and conditions: a = mesitylene, rfx, overnight, Y = 92%; b, c = next steps (decarbonylation reactions and FeCl_3_ oxidative cyclization).

In 2006 Pei’s research team reported synthesis of a series of planar and non-planar (helical) thiophene-based polycyclic aromatics. Described compound has been synthesized through the Diels–Alder reaction of acetylenes and cyclopentadienone derivatives followed by decarbonylation reactions and FeCl_3_ oxidative cyclization (Scheme 53) [107].

**Scheme 54:**
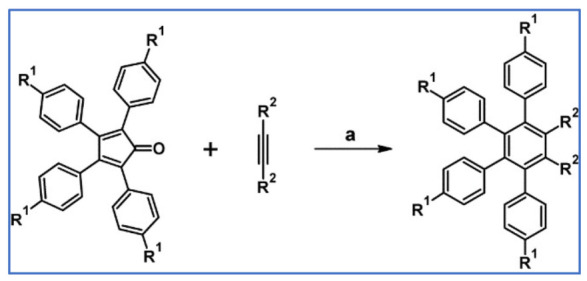
Syntheses of hexarylbenzenes possessing two heteroaryl motifs via DA cycloaddition with CO extrusion [108]. Reagents and conditions: (a) R^1^ = Me, OMe, *t*-Bu, Br, –C≡C–Si(*i*-Pr)_3_, R^2^ = 3,5-diazene-1-yl; a = benzophenone, dipirimidyl acetylene; a = rfx, 1 h; up to 85% yield; (b) for R^1^ = *t*-Bu, R^2^ = 2-thienyl; a = benzophenone, rfx, 1 h; Y = 75%.

Several hexaarylbenzenes bearing four *p*-substituted phenyl and two heteroaryl motifs were obtained involving DA cycloaddition with appropriately substituted cyclopentadienones and di-heteroaryl-acetylenes as substrates (Scheme 54) as reported by Gregg et al., in 2006 [108].

One of the products, namely the one with *t*-Bu (R^1^) and 3,5-diazene-1-ylic (R^2^) groups, underwent partial and complete dehydrocondesation. The latter dehydrocondesation lead to tetraaza-HBC.

**Scheme 55:**
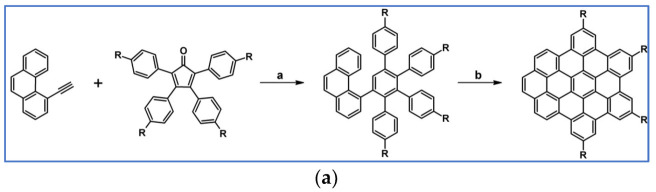

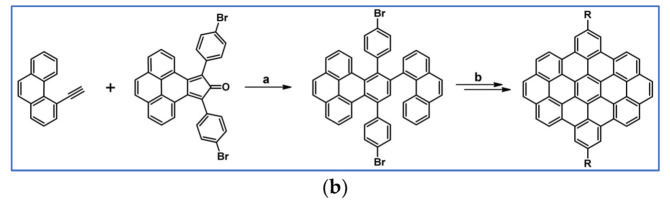
Syntheses of ovalene derivatives starting from DA cycloaddition with CO extrusion followed by next transformation [109]. Reagents and conditions: R = C_12_H_25_; (**a**) a = *o*-xylene, 160 °C, 3 h, Y = 95%; b = next step (Scholl reaction); (**b**) a = *o*-xylene, 160 °C, 5 h, Y = 75%, b = next steps (Suzuki–Miyaura coupling and Scholl reaction).

In work devoted to the syntheses of different sizes and symmetry PAHs’ molecules, two model compounds were prepared via DA cycloaddition with CO extrusion, accompanied by Suzuki–Miyaura coupling and finally Scholl reaction (Scheme 55) [109].

The influence of size, symmetry, shape, as well as peripheral groups on the electronic and other properties of all obtained PAHs were thoroughly discussed.

**Scheme 56:**
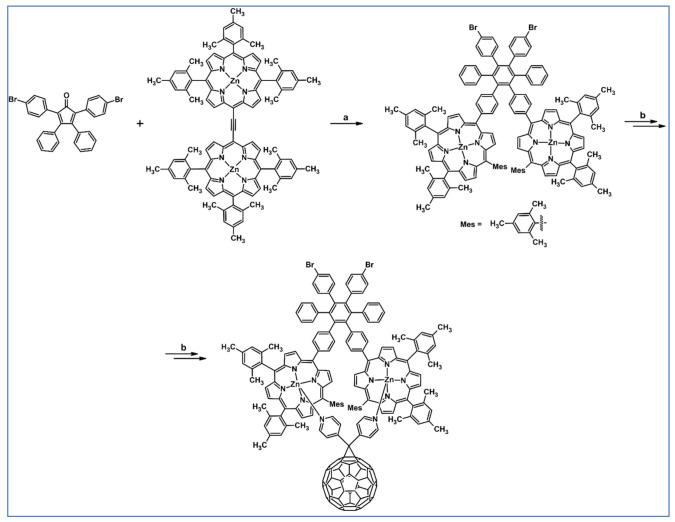
DA cycloaddition for the synthesis of the hexaphenylbenzene-based zinc porphyrin dyad followed by 1:1 complex creation with a fullerene bearing two pyridyl groups (the pyridyl nitrogens coordinate with the zinc atoms) [110]. Reagents and conditions: a = Ph_2_O, rfx, 4 h, Y= 67%; b = next steps.

The following year, Terazono described DA cycloaddition of acetylene bearing a porphyrin–zinc moiety and tetrapnenylcyclopentadienone. It was applied in the synthetic route of hexaphenylbenzene-based zinc porphyrin dyad complex (1:1) with a fullerene bearing two pyridyl groups (via coordination of the pyridyl nitrogens with the zinc atoms) (Scheme 56) [110].

The complex between two zinc porphyrins and fullerene, which is symmetrically located between Zn-atoms is characterized by the binding constant equal 7.3·10^4^ M^−1^ (in 1,2-difluorobenzene). Photophysical properties of this complex (extremely rapid electron transfer from a porphyrin first excited singlet state to the fullerene and the resulting charge-separated state characterized by a relatively slow lifetime) will be helpful in the construction of a photosynthetic antenna-reaction centre.

**Scheme 57:**
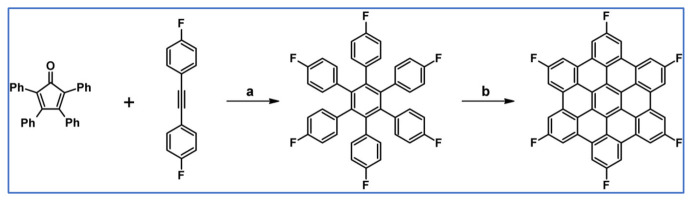
DA leading to F_6_-substituted HPB followed by F_6_-substituted HBC [111]. Reagents and conditions: a = Ph_2_O, 260 °C, 9 h, Y= 72%; b = next step (Scholl reaction).

As reported in 2007 by Kikuzawa and co-workers, fluorine-substituted hexa-peri-hexabenzocoronene was synthesized via tandem: DA cycloaddition with CO extrusion and Scholl reaction (Scheme 57) [111].

It should be added that attempts of catalytic cyclotrimerization 1,2-bis(*p*-fluorophenyl)acetylene to expected F_6_-HPB, via well-established method, notably in the presence of [Co_2_(CO)_8_], were not successful at all. Obtained F_6_-HBC can be used as a thermostable active material for *n*-type semiconductors. Additionally, the F_6_-HBC LUMO and HOMO energy levels were lower by 0.5 eV than those of HPB. Interestingly, the electron-withdrawing effect of the fluorine substituents changed the polarity from *p*-type to *n*-type (a field-effect transistor was fabricated).

**Scheme 58:**
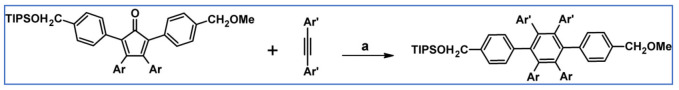
DA cycloaddition with CO extrusion as a practical tool for the syntheses of HAB with a unique group at the 1,4-position [112]. Reagents and conditions: a = Ph_2_O, rfx, 19 h, Y= 86% (for Ar = Ph; Ar’= Ph) or Y = 80% (for Ar = Ph; Ar’ = *p*-MeOPh) or Y = 72% (for Ar = *p*-BrPh; Ar’ = *p*-MeOPh) or Y = 62% (for Ar = *p*-BrPh; Ar’ = *p*-BrPh).

The same year, peculiarly substituted tetraarylcyclopentadienones were obtained via asymmetric carbonylative couplings of benzyl halides to give heterosubstituted 1,3-diarylacetones in the initial step. In the next step, the synthesized acetone derivatives were converted in Knoevenagel condensation to desired cyclopentadienones. Finally, the latest diarylsubstituted acetylenes underwent further conversion via dehydro-DA cycloadditions to give highly heterofunctionalized hexaarylbenzenes with uniquely functionalized aryl groups at the para positions of the central benzene ring (Scheme 58) [112].

It is worth mentioning that such a method allows for the substituents’ control on each of four unique pendant aryl group positions, initiating substitution patterns.

**Scheme 59:**
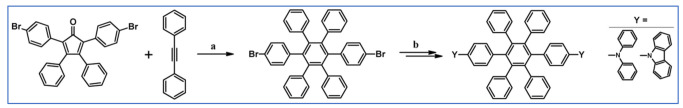
DA cycloaddition with CO extrusion for the synthesis of hexaphenylbenzene derivatives bearing diphenylamine or *N*-carbazole moieties [113]. Reagents and conditions: a = PhCOPh, 300 °C, 0.33 h, Y = 77%; b = next steps.

DA cycloaddition between diphenylacetylene and appropriately substituted cyclopentadienone was used by Watanabe and Kido to synthesize hexaphenylbenzene derivatives having high triplet energy levels (Scheme 59) [113].

The obtained compounds were used in blue organic electrophosphorescent devices, i.e., a blue OLED. The device with a blue phosphorescent emitter, iridium(III) bis[(4,6-difluorophenyl)pyridinato-*N*,C^2′^]-picolinate, exhibited a high external quantum efficiency of 11% (24 cd/A) and a high power efficiency of 12 lm/W at 100 cd/m^2^.

**Scheme 60:**
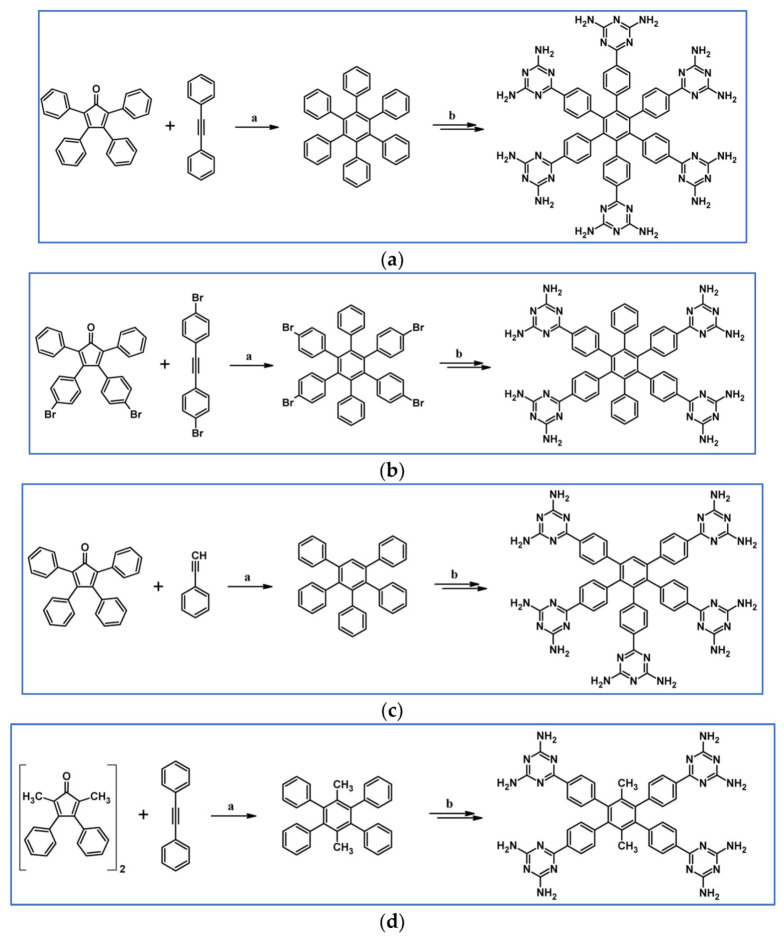
DA cycloaddition with CO extrusion involved in the synthesis of polysubstituted benzene containing peripheral 2,4-diamino-1,3,5-triazin-6-yl moieties—examples [114]. Reagents and conditions: (**a**–**d**) a = Ph_2_O, rfx, 0.5–48 h, up to 93% yield; b = next steps, overall yields up to 47%.

In 2007, Kenneth and colleagues presented DA cycloadditions with CO extrusion, which were the initial steps leading to the polysubstituted benzenes containing four, five, or six peripheral 2,4-diamino-1,3,5-triazin-6-yl (DAT) moieties including hexakis[4-(2,4-diamino-1,3,5-triazin-6-yl)phenyl]benzene (Scheme 60) [114].

The peripheral diaminotriazine groups were engaged in the creation of hydrogen bonds networks. The results demonstrate that significant structural alterations of the polysubstituted benzene core can appreciably change the resulting hydrogen bond network [114].

**Scheme 61:**
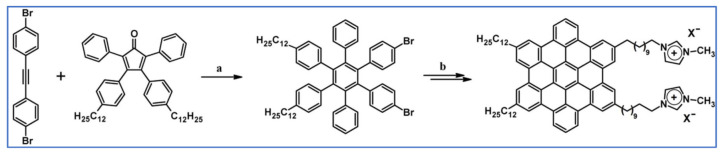
DA cycloaddition of appropriately substituted cyclopentadienone and acetylene in the synthetic route of imidazolium-based amphiphilic hexa-peri-hexabenzocoronenes [115]. Reagents and conditions: a = Ph_2_O, rfx, overnight; Y = 68%; b = next steps.

Another example of applying DA cycloaddition with CO extrusion as an initial step in synthesis was reported by Hamaoui and co-workers. Obtained imidazolium-based amphiphilic hexa-peri-hexabenzocoronenes are presented in Scheme 61. The final product was characterized by ordered columnar self-assembly in solid and solution, leading to defined nanofibers [115].

Significantly, molecular organization and packing, both in solid state and solution, differ majorly. Layer-like columnar molecule aggregation is evident in solid state, while as a solution, HBC discs are organized into well-defined nanofibers due to self-assembly behaviour, numerous weak hydrophilic and hydrophobic interactions, and π-stacking effect. Due to the above-mentioned phenomenons, one should expect that those nanofibers will exhibit satisfying ion conductivity and charge mobility.

**Scheme 62:**
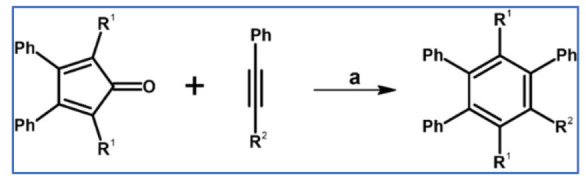
Solventless variant of DA cycloaddition with CO extrusion [116]. Reagents and conditions: (a) R^1^ = Ph, R^2^ = H (a = 135 °C, 10 min; Y = 97%); (b) R^1^ = 4-biphenyl, R^2^ = H (a = 135 °C, 10 min; Y = 95%); (c) R^1^ = R^2^ = Ph (a = 175 °C, 5 h; Y = 93%); (d) R^1^ = 4-biphenyl, R^2^ = Ph (a = 175 °C, 6 h; Y = 90%).

Penta- and hexasubstituted benzenes were obtained via DA cycloaddition with CO extrusion (Scheme 62) according to report from Thiemann et al. [116].

**Scheme 63:**
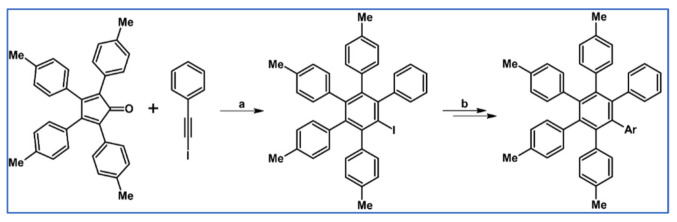
DA cycloaddition as an initial step in the reaction procedure leading to the functionalized pentaarylbenzenes [117]. Reagents and conditions: sources of Ar (electrophiles) = 1,2-dibromobenzene, PhCHO, Ac_2_O, Bu_3_SnCl, CO_2_, B(OMe)_3_ and others; a = *o*-xylene, rfx, 11 h; Y = 71%; b = next steps (lithiation followed by reaction with electrophiles).

DA cycloaddition with CO extrusion was also applied in the synthesis of iodo-pentaarylbenzene, which was the crucial substrate in the convenient preparation of functionalized pentaarylbenzenes through a quick lithiation (Scheme 63) [117].

**Scheme 64:**
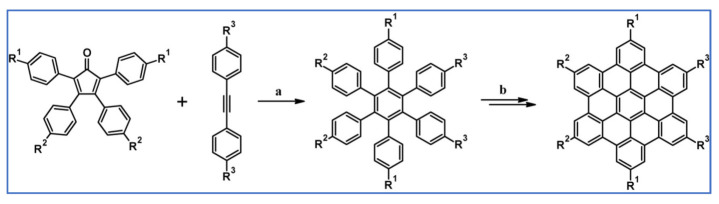
DA cycloaddition of appropriately functionalized cyclopentadienone and acetylene in the synthetic route leading to three classes of classes hexa-peri-hexabenzocoronenes (HBCs). Reagents and conditions: (a) R^1^ = H, R^2^ = OC_12_H_25_, R^3^ = –C_6_H_4_–(CH_2_CH_2_O)_3_Me; (b) R^1^ = Br, R^2^ = C_12_H_25_, R^3^ = –C_6_H_4_–(CH_2_CH_2_O)_3_Me; (c) R^1^ = R^2^ = H, R^3^ = –C_6_H_4_–(CH_2_CH_2_O)_3_Me; (d) R^1^ = H, R^2^ = C_12_H_25_, R^3^ = –C_6_H_4_–OC_12_H_25_; (e) R^1^ = H, R^2^ = C_12_H_25_, R^3^ = H; and others; a = Ph_2_O, rfx, 20–72 h; b = next steps (Scholl reaction), up to 57% yield (final HBCs).

In 2008, thirteen different hexa-peri-hexabenzocoronenes (HBCs) belonging to the three classes were synthesized via DA cycloaddition of appropriately functionalized cyclopentadienones and acetylenes, which was essential for the creation of hexaaryl substituted benzene core (Scheme 64) [118].

Self-assembling behaviours studies revealed that some structural parameters of HBCs are pivotal for their tubular assembly. It was proven that the role of two phenyl groups present at one side of the HBC scaffold is vital. Moreover, long paraffinic chains play a crucial function as far as assembly properties are concerned. Surprisingly, the presence of hydrophilic moiety is not necessary.

**Scheme 65:**
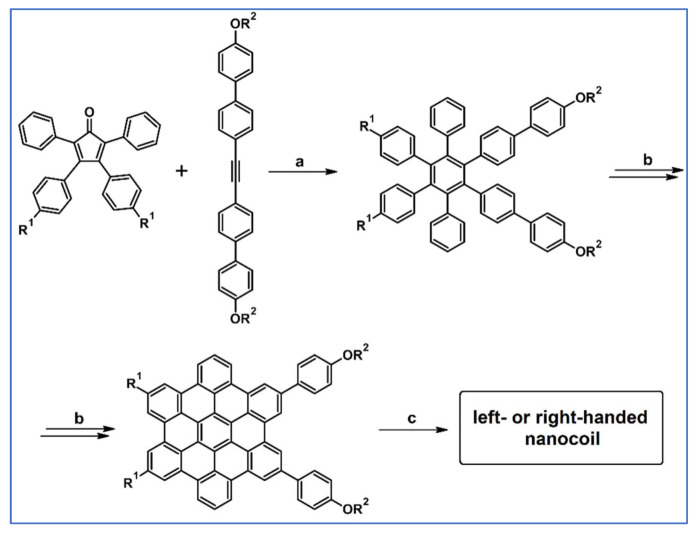
DA cycloaddition with CO extrusion and Scholl dehydrocondensation engaged in the synthetic route leading to helically chiral one-handed nanocoil [119]. Reagents and conditions: R = achiral or *C*-chiral terminal –OCO– norbornenyl containing chains; a = Ph_2_O, rfx, Y = up to 47%; b, c = next steps (Scholl reaction and co-assembly of HBC).

Using DA cycloaddition followed by Scholl reaction and finally co-assembly of HBC (equipped with *C*-chiral norbornene motif), covalently stabilized, electroconductive, and one-handed nanocoils were obtained by Yamamoto’s research team (Scheme 65) [119].

**Scheme 66:**
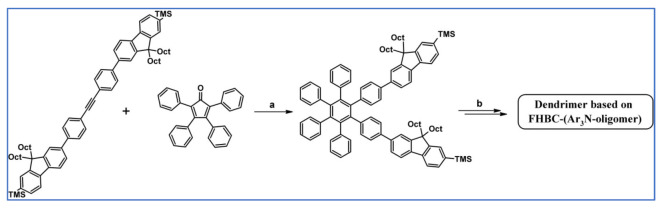
Synthesis of dendrimers based on HBC equipped with fluorene and triarylamine-oligomers motifs involving DA cycloaddition with CO extrusion—an example [120]. Reagents and conditions: a = Ph_2_O, 260 °C, 24 h, Y = 99%; b = next steps (i.a. Buchwald–Hartwig amination).

DA cycloaddition with CO extrusion combined with Buchwald–Hartwig coupling were essential steps in the synthetic route leading to the fluorene-containing hexabenzocoronenes (FHBC), which additionally possessed triarylamine oligomers moieties (Scheme 66) as reported by Wong et al. [120].

The obtained series of dendritic fluorenylhexabenzocoronene (FHBC) compounds equipped with triarylamine oligomers (responsible for fluorescence quenching) belongs to the electron-donating materials. Combined with fullerene derivatives as electron acceptor components, these materials were tested to be effective in bulk heterojunction solar cells (>5% EQE).

**Scheme 67:**
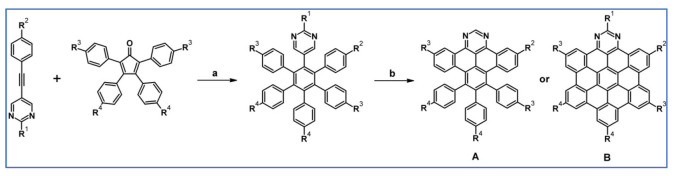
DA cycloaddition involved in the syntheses of *N*-doped hexaarylbeznenes and HBC [121]. Reagents and conditions: R^1^-R^4^ = H or *t*-Bu in various position (for **A**); R^1^-R^4^ = *t*-Bu (for **B**); a = Ph_2_O, 260 °C, 45 min, up to 62%; b = next step (Scholl reaction).

The same year, pyrimidyl-penta-phenylbenzenes were synthesized by DA cycloaddition of substituted phenylethynylpyrimidines and tetraphenylcyclopentadienones under MW irradiation (Scheme 67) [121]. Scholl reactions of these compounds led to the hetero-polyaromatic hydrocarbons including diaza-hexa-peribenzocoronene (which require the presence of tert-butyl group in the pyrimidine ring).

**Scheme 68:**
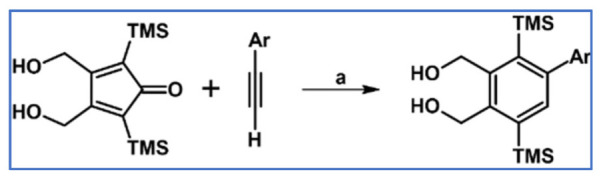
Syntheses of aryl–aryl and aryl–hetaryl type derivatives via DA cycloaddition with CO extrusion [122]. Reagents and conditions: Ar = Ph, 2-O2NPh, 4-MeO2Ph, 2-MeOPh, 1-naphthyl, 2-pyridyl and others (11 examples); a = PhMe, 110 °C, 48 h, Y = up to 88%.

Biaryls and aryl–heteroaryl derivatives were obtained via DA cycloaddition involving various aryl(hetaryl)acetylenes and 3,4-di(hydroksymethyl)-2,5-(trimethylsilyl)cyclopentadienone (Scheme 68) according to a paper from Pearson et al. [122].

Regarding the roles of the dienophiles substituents, it has been observed that the cycloaddition product yield is higher for both electron-withdrawing ones and those showing a weaker steric effect.

**Scheme 69:**
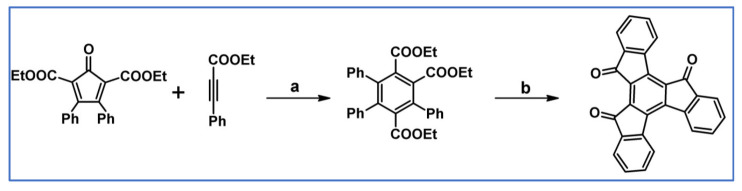
DA cycloaddition with CO extrusion involved in the syntheses of isotruxenone precursor [123]. Reagents and conditions: a = 180 °C, 24 h, Y = 76%; b = next step.

In 2009, Yang’s research team described preparation of isotruxenone core involving DA cycloaddition of suitable cyclopentadienones and acetylenes (Scheme 69) [123]. The authors also showed the possibility of obtaining this compound starting from Co-catalyzed cyclotrimerization of 1-phenyl-1-propyne.

We share the authors’ opinion that above-presented method should facilitate the development of isotruxene-based materials.

**Scheme 70:**
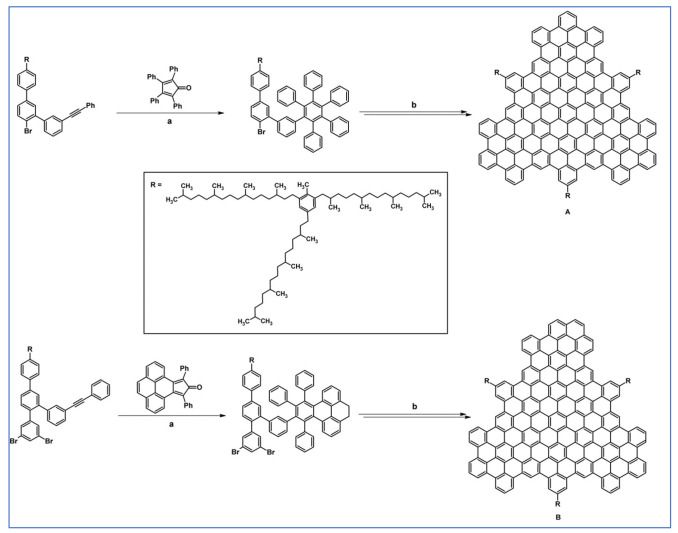
DA cycloaddition involved in the synthesis of graphene quantum dots [124]. Reagents and conditions: a = Ph_2_O, rfx; Y = 61% (for **A**), Y= 40% (for **B**).

DA cycloaddition with CO extrusion and Scholl dehydrocondensation were crucial synthetic tools engaged in synthesizing sizeable colloidal graphene quantum dots with a uniform and tunable size, through solution chemistry (Scheme 70) as stated by Yan and colleagues [124].

Quantum dots presented in this work have significant extinction coefficients in a wide spectral range from UV to near-infrared and, thus, can serve as a new type of light-harvesting media for photovoltaics.

**Scheme 71:**
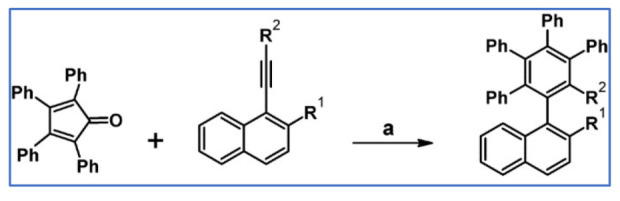
The convenient synthesis of atropisomeric biaryls via DA cycloaddition with CO extrusion [125]. Reagents and conditions: R^1^, R^2^ = H, P(O)Ph_2_; OMe, P(O)Ph_2_; OMe, COPh and others; a = Ph_2_O, 260 °C, 8 h, yield up to 93%.

The DA cycloaddition with CO extrusion between tetraphenycyclopentadienone and functionalized, internal aryl acetylenes gave access to a vast range of bulky atropisomeric biaryls with good to excellent yield (Scheme 71) as described by Hapke and co-workers [125].

Notably, the selected atropisomers have been resolved via the diastereomers quickly and efficiently to yield the pure enantiomers.

**Scheme 72:**
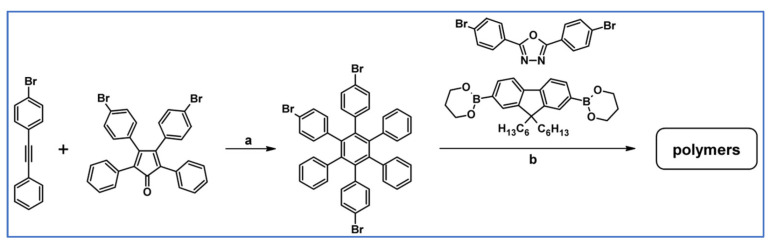
DA cycloaddition involved in the synthetic way leading to the hyperbranched conjugated polymers containing hexaphenylbenzene as the core [126]. Reagents and conditions: a = Ph_2_O, rfx, Y = 86%; b = next step.

In 2010, Li et al., described DA cycloaddition with CO extrusion as an initial step in synthesizing hyperbranched conjugated polymers containing hexaphenylbenzene as the core through a one-pot Suzuki polymerization reaction (Scheme 72) [126].

Interestingly, all obtained hyperbranched polymers were characterized by good solubility in organic liquids, film-forming ability and thermal stability. Moreover, the discussed materials emitted pure deep-blue light in a stable manner in air and at increased temperatures. Finally, two-layer PLED devices fabricated from the polymer mentioned above were promising as far as their electroluminescence, luminance efficiency, and brightness are concerned.

**Scheme 73:**
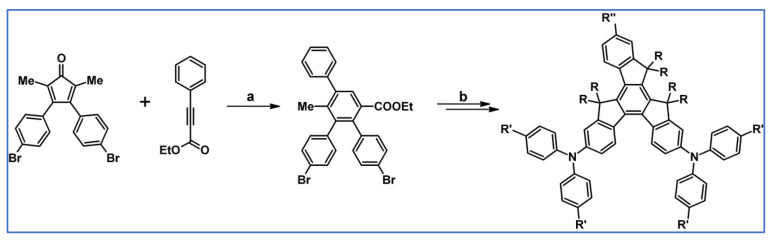
DA cycloaddition as a crucial step in the cone-shaped organic dye core [127]. Reagents and conditions: R = Et, *n*-C_6_H_13_, R’ = H or OMe, R’’ = CHO, 2-thienyl and others; a = PhMe, rfx, 24 h, Y = 91%; b = next steps.

As Lin’s research team stated in their paper, another example of such a reaction type was involved in the multiple-step synthetic route of cone-shaped organic dyes containing the isotruxene π-scaffold (Scheme 73) [127].

Obtained isotruxene dyes display reversible anodic waves in cyclic voltammograms with both HOMO and LUMO potentials suitable for application in dye-sensitized solar cells (DSSCs). The DSSCs fabricated with these organic dyes exhibited high open-circuit voltages (0.67–0.76 V) and fill factors (0.67–0.72) with a power conversion efficiency (η) up to 5.45%, which is 80% of the ruthenium dye N719-based standard solar cell.

**Scheme 74:**
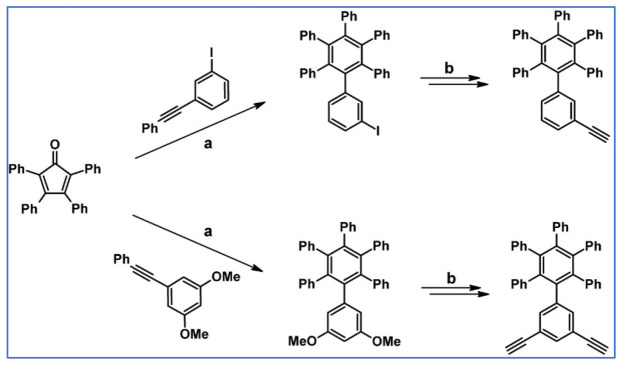
DA cycloaddition involved in the synthetic route leading to the derivatives of HPB [128]. Reagents and conditions: a = Ph_2_O, rfx, 20 h, up to 86% yield; b = next steps.

Hexaarylbenzenes were synthesized via DA cycloaddition involving tetraphenylcyclopentadienone and appropriately substituted acetylene as diene and dienophiles, respectively (Scheme 74) [128].

HBC and its derivatives shown in the scheme above were considered a potential acetylene sponge as hexaphenylbenzenes have a particularly strong tendency to form C-H⋯π interactions with alkynes [128].

**Scheme 75:**
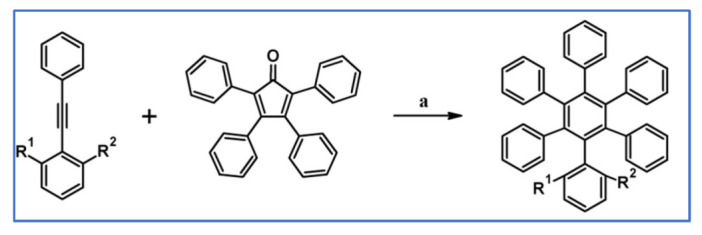
DA cycloaddition of tetraphenylcyclopentadienone and substituted acetylenes leading to diversified hexphenylbenzenes [129]. Reagents and conditions: a = Ph_2_O, rfx, 24–96 h; R and R’ = H, Me, Et or *i*-Pr, up to 77% yield.

Gagnon and colleagues in 2010 described synthesis of a series of hexaphenylbenzenes (HPBs), in which one phenyl motif contained substituents in positions 2 and 6, were obtained via DA cycloaddition of tetraphenylcyclopentadienone and appropriately substituted acetylenes (Scheme 75) [129].

To compare HPB and its ortho-substituted derivatives, one should mainly take into consideration the influence of changing H···H, C···H, C···C and C-H⋯π-interactions on their final properties. It was proven that such interactions could be restricted by adding simple ortho-alkyl substituents to the peripheral phenyl groups. It should be emphasized that the properties of hexaphenylbenzene (HPB) and analogous compounds hold broad applicability in material chemistry and technology. These properties include conformationally well-defined molecular structures, high thermal stability, high HOMO–LUMO gaps, little self-association, inefficient packing, and high solubilities.

**Scheme 76:**
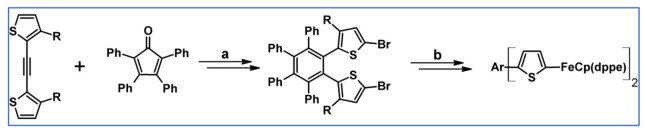
Syntheses of hexaarylbenzenes bearing two thienyliron substituents: DA cycloaddition involved in the substituted-HPB core creation [130]. Reagents and conditions: (a) R = H; a = NBS, THF, next Ph_2_O, rfx, 24 h, Y = 63%; (b) R = Me; a = Ph_2_O, rfx, 24 h, followed by NBS, THF, Y = 96%.

An alternative series of hexaarylbenzenes having two peripheral thienyliron substituents has been prepared involving DA cycloaddition with CO extrusion (Scheme 76) [130].

It was revealed that the two metal centres communicate with each other through toroidal delocalization among the peripheral aromatic groups—see scheme above.

**Scheme 77:**
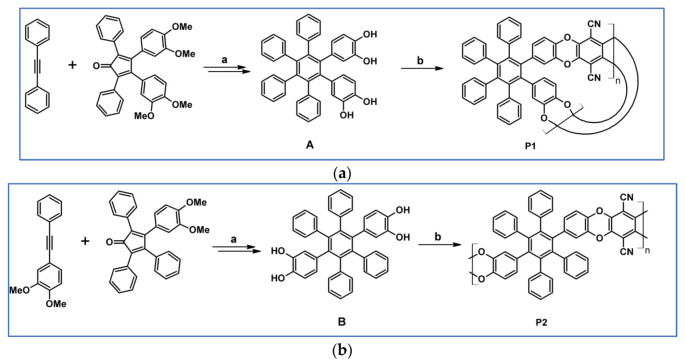
DA cycloaddition as a tool allowing to obtain monomers for polymerization [131]. Reagents and conditions: (**a**,**b**) a = Ph_2_O, 250 °C, 15 h, next BBr_3_, CH_2_Cl_2_, 20 °C, 1 h, Y = 89% (for **A**) and 91% (for **B**); b = next step (polimerization reaction).

Another report from 2011 by Short et al., described DA cycloaddition as an initial step in synthesising microporous polymers derived from the 1,2- and 1,4-regioisomers of di(3′,4′-dihydroxyphenyl)tetraphenylbenzene (Scheme 77) [131].

Permeability data referring to **P1** presents an excellent example of a compromise in the trade-off between the desirable properties of high permeability and good selectivity required for efficient gas separation membranes. **P2** showed significantly lower permeabilities than **P1** but enhanced selectivities (e.g., selectivity for CO_2_ vs. N_2_ = 26 for **P2** and 18 for **P1**).

**Scheme 78:**
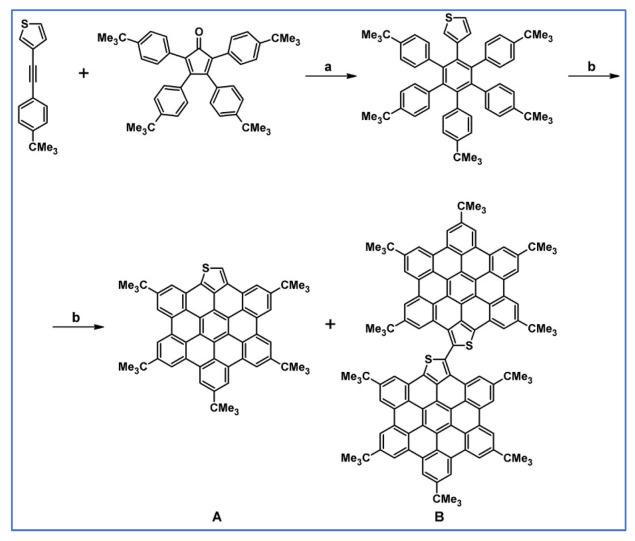
DA cycloaddition and Scholl reaction involved in the syntheses of S-doped HBC [132]. Reagents and conditions: a = Ph_2_CO, 300 °C, 45 min, Y = 87%; b = next step (Scholl reaction).

In 2011, Martin and colleagues reported the synthesis of S-doped HBC due to the combination of the desirable electronic and photochemical properties of hexabenzocoronene (HBC) and bearing in mind the ease of C–C bond forming of thiophenes. Hence, 1-(3-thienyl)-2,3,4,5,6-penta(4-tert-butyl-phenyl)benzene was obtained via DA cycloaddition using dienophile containing thiophene motif, then it was followed by oxidation with FeCl_3_ (Scheme 78) [132].

Interestingly, dimeric species **B** has concentration-dependent optical properties due to the orientation of the two HBC platforms around the newly formed dimer bond.

**Scheme 79:**
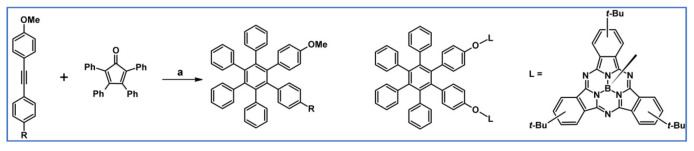
Syntheses of HPB substituted with one or two methoxy group [133]. Chemical structure of bis-[*B*(SubPc)]. Reagents and conditions: R = H, OMe; a = Ph_2_O, rfx, 12 h, Y = 46% (for R = OMe) or a = sulfolane, 260 °C, 7.5 h, Y = 88%.

Mono- to highly congested hexameric subphthalocyanines were synthesized by axial chlorine-to-phenoxy substitution of mono-, two-, and hexakis(4-hydroxyphenyl)benzenes. The latest were prepared via acetylene cyclotrimerization, but mono- and di-substituted (4-hydroxyphenyl)benzenes were obtained by DA cycloaddition. Syntheses engaging DA reactions with CO extrusion were presented on the scheme below (Scheme 79) [133].

**Scheme 80:**
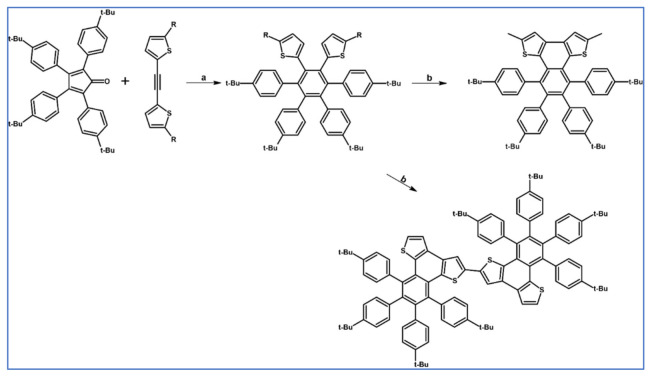
DA cycloaddition between appropriately substituted cyclopentadienone and acetylene as an initial step in the syntheses of the polyphenylenes bearing dithienyl moieties [134]. Reagents and conditions: R = Me, H; a = Ph_2_CO, 300 °C, 1.5 h; Y = 41% (for R = Me), Y = 66% (for R = H); b = next step (Scholl reaction).

Once again, DA cycloaddition with CO extrusion was an initial step in the synthetic procedures leading to the set of dithienyl polyphenylenes (Scheme 80) [134].

**Scheme 81:**
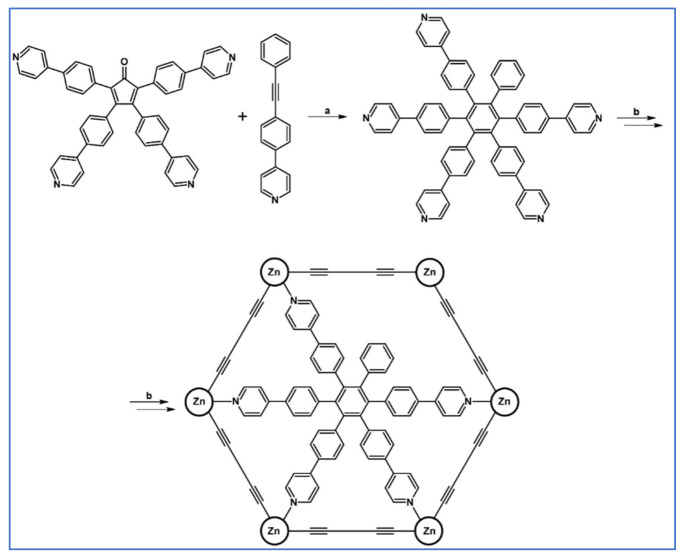
The example of supramolecular structure: hexamer of zinc porphyrin-hexa(pyridylphenyl)benzene [135]. Reagents and conditions: a = 280 °C, 4 d; b = multistep reaction.

In 2011, Hogben and her team applied DA cycloaddition of appropriate cyclopentadienones and acetylenes for the syntheses of ligands belonging to the derivatives of hexaphenylbenzene bearing from one to six 4-pyridyl moieties (Scheme 81) [135].

The ligands shown in Scheme 81 were employed in the research devoted to cooperation between zinc porphyrin oligomers and multivalent ligands, namely hexaphenylbenzene bearing from two to six pyrudyl-coordination centres. Importantly, this work demonstrates that rigid preorganized shape-complementary multivalent host-guest systems can exhibit high effective molarities (up to 10^3^ M) and very high association constants (up to 10^36^ M^−1^).

**Scheme 82:**
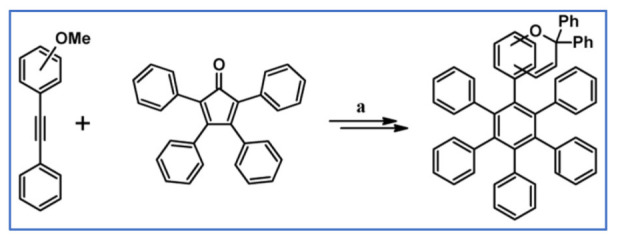
DA cycloaddition in the synthesis of photochromic chromenes [136]. Reagents and conditions: a = Ph_2_O, 280 °C, 10 h, Y = 82–88%, then next step.

DA cycloaddition of appropriate acetylene and cyclopentadienone was engaged in synthesizing regioisomeric photochromic chromenes substituted with the (2,3,4,5,6-pentamethyl/phenyl)phenyl scaffold (Scheme 82) [136].

**Scheme 83:**
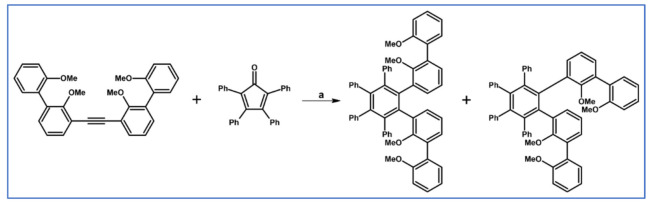
3D ligand precursors synthesis via DA cycloaddition involving tetraphenylcyclopentadienone and acetylenes possessing two bisphenol moieties [137]. Reagents and conditions: a = Ph_2_O, 250 °C, 24 h, Y= 63%; diastrereoselective reaction (cis/trans = 35:65).

As Amaya et al., stated in 2011, ligand precursors for titanium complexes (catalysing pinacol cross-coupling) were obtained via DA cycloaddition with CO extrusion (Scheme 83) [137].

**Scheme 84:**
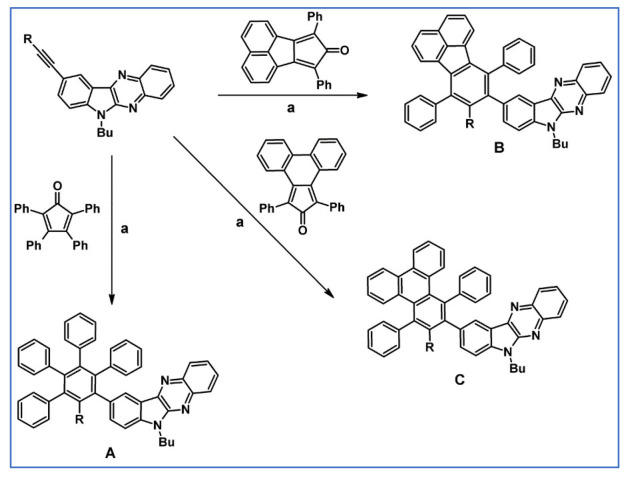
DA cycloaddition as a crucial step in the syntheses of indoloquinoxaline derivatives containing bulky polyaromatic hydrocarbons moieties [138]. Reagents and conditions: a = Ph_2_O, 200 °C, 24 h; R = H, Ph; **A**: Y = 92% (for R = H), Y = 91% (for R = Ph); **B**: Y = 95% (for R = H), Y = 92% (for R = Ph); **C**: Y = 72% (for R = H), Y = 60% (for R = Ph).

Hybrid materials featuring the dipolar fragment 6*H*-indolo[2,3-*b*]quinoxaline attached to the bulkier polyaromatic hydrocarbons (such as fluoranthene, triphenylene, or polyphenylated benzene) have been successfully synthesized in 2011 involving DA cycloaddition (Scheme 84) [138].

The electronic properties of the compounds are dominated by the 6*H*-indolo[2,3-*b*]quinoxaline chromophore, but the polyaromatic moiety reduces the chances of nonradiative deactivation processes and improves the emission properties. Moreover, the presence of rigid 6*H*-indolo[2,3-*b*]quinoxaline and bulkier polyaromatic segments enhances the thermal stability and glass transition temperature significantly. Optical, electrochemical, and other properties suggest that obtained compounds are promising electron-transporting and emitting materials, suitable for double layer organic light-emitting diodes.

**Scheme 85:**
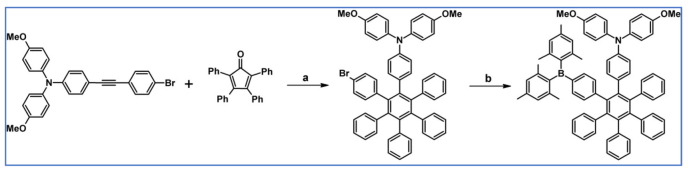
Synthesis of hexarylbenzene possessing *p*-[di(mezytyl)boryl]phenyl]- and *p*-[di(*p*-methoxyphenyl)amino]-groups in the 1,2-position [139]. Reagents and conditions: a = Ph_2_O, rfx, 72 h, Y = 55%; b = next step, Y = 29%.

DA with CO extrusion was also used in 2012 by Steeger and Lambert to synthesize a model compound necessary in the research on charge–transfer interactions in tris-donor-tris-acceptor hexaarylbenzene redox chromophores (Scheme 85) [139].

The examined HAB were synthesized via non-regioselective Co-catalysed cyclotrimerization of appropriate acetylene. The compounds under scrutiny belong to symmetrical and non-symmetrical HAB possessing three *p*-[di(mezytyl)boryl]phenyl]-groups and three *p*-[di(*p*-methoxyphenyl)amino]-groups.

**Scheme 86:**
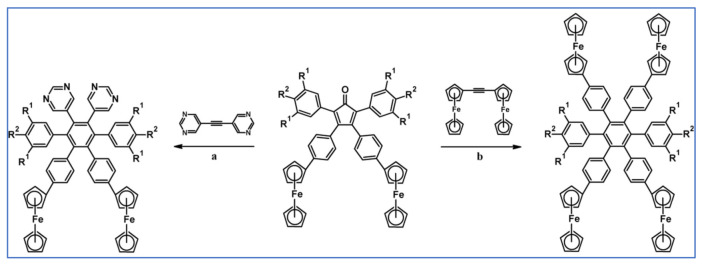
DA cycloaddition between appropriately substituted acetylenes and cyclopentadienones in the synthetic route leading to polyphenylenes bearing ferrocenyls moieties [140]. R^1^ = H or OMe, R^2^ = H or ferrocenyl; Reagents and conditions: a = PhCOPh, 200 °C, 18 h, up to 58% yield; b = PhCOPh, 190 °C, 18 h, Y = 18% (for R^1^).

The syntheses and characterisation of a series of polyphenylenes with up to four ferrocenyl moieties via [2 + 4] Diels–Alder cycloadditions with appropriately substituted acetylenes and cyclopentadienonees were described the same year by Roberts and colleagues (Scheme 86) [140].

The electrochemical, absorption, and NLO properties of the ferrocenyl-polyphenylenes are presented and discussed in the cited work.

**Scheme 87:**
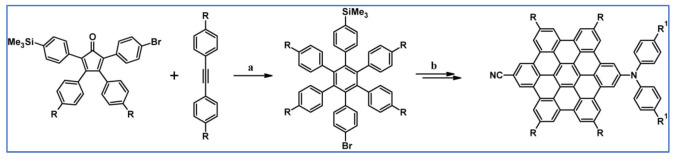
DA cycloaddition with CO extrusion in the synthetic route leading to hexa-peri-hexabenzocoronene derivatives [141]. Reagents and conditions: R = 2-ethylhexyl, R^1^ = Me or MeO; a = Ph_2_O, 185 °C, 12 h, Y = 49%; b = next steps (i.a. Scholl reaction).

DA cycloaddition with CO extrusion was involved in the syntheses of two hexa-peri-hexabenzocoronene (HBC) derivatives possessing strong molecular dipole moments (MDM) (Scheme 87) [141]. As Chen et al., claimed in their paper, both the push–pull structure of the obtained HBCs, and the presence of one EDG (Nar_2_) and one EWG (NC) substituent in their structures are responsible for such a high value of dipole moment.

Additionally, the high value of MDM is also responsible for strong intermolecular dipole–dipole interactions and self-association of both HBCs in solution and the liquid crystalline mesophase.

**Scheme 88:**
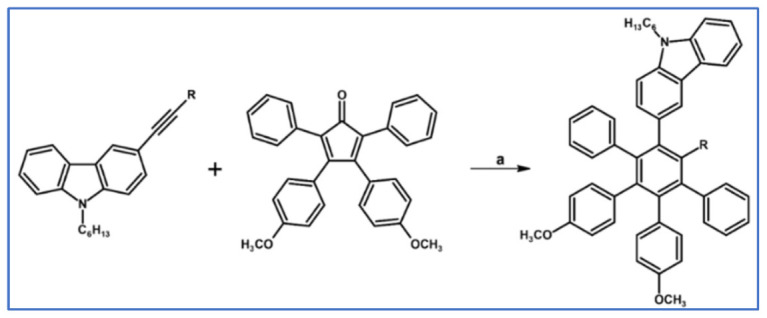
Syntheses of hexaarylbenzenes bearing one carbazoyl and two dimethoxyphenyl moieties via DA cycloaddition with CO extrusion [142]. Reagents and conditions: R = H, Ph; a = Ph_2_O, rfx, 18 h, Y = 82% (for R = H) and Y = 81% (for R = Ph).

Kumar, Vij, and Bhalla reported in 2012 that AIE-exhibiting penta- and hexaarylbenzenes equipped with specific chromophores were prepared using classical DA cycloaddition with CO extrusion, appropriately substituted cyclopentadienone and acetylenes (Scheme 88) [142].

Both compounds were envisioned as perfect selective sensors for TNT in solution, solid, and vapor state (at the picogram level).

**Scheme 89:**
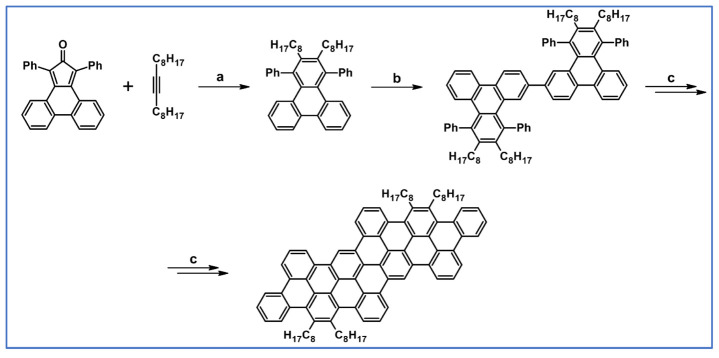
Synthesis of PAH via DA cycloaddition-Scholl dehydrocondensation [143]. Reagents and conditions: a = Ph_2_O, rfx, Y = 61%; b = dimerization; c = Scholl reaction.

In work dedicated to the syntheses of penthacene derivatives from 2012, one of potential precursors, namely 1,2-diheptyl-3,4,5,6-tetraphenylbenzene, was obtained via DA cycloaddition involving 8-hexadecyne and tetraphenylcyclopentadienone as substrates (Scheme 89) [143].

Interestingly, this precursor was unreactive in the Scholl reaction. Namely, all attempts to obtain the expected pentaphene failed and intermolucular dimerization product was obtained. However, with the use of other cyclopentadienone, a fully aromatic structure was prepared (Scheme 89).

**Scheme 90:**
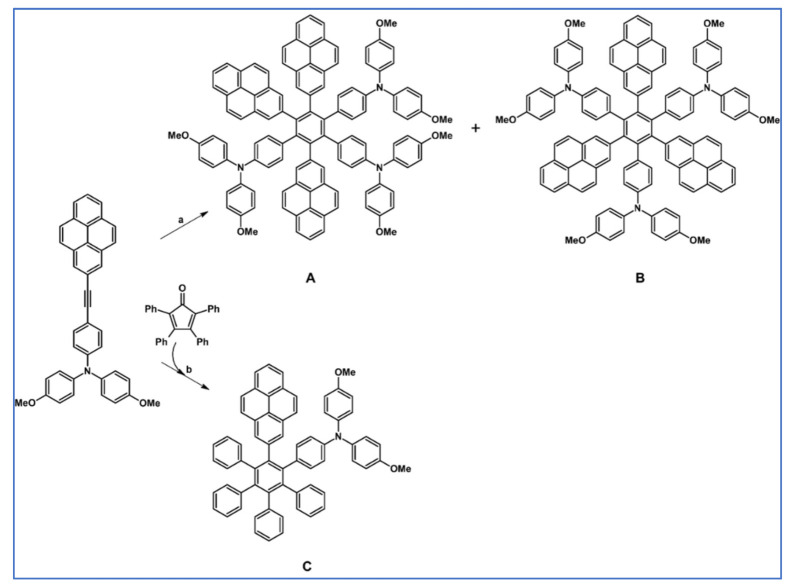
DA cycloaddition with CO extrusion and Co-catalysed acetylene cyclotrimerization involved as a main synthetic tools for multichromophoric systems preparation [144]. Reagents and conditions: a = Co_2_(CO)_8_, dioxane, 7 d, 101 °C, Y = 18% (for **A**) or 2% (for **B**); b = Ph_2_O, rfx, 7 d, Y = 57% (for **C**).

In 2012, Lambert and colleagues described the synthetic route for preparing hexaarylbenzenes substituted with pyrene and triarylamine units. Products were obtained via DA reaction with carbon monoxide extrusion and Co-catalyzed acetylene cyclotrimerization (Scheme 90) [144].

In the resulting extended aromatic systems, pyrenes act as acceptors, while triarylamines are strong donors of electrons. Considering the fluorescent properties of the obtained chromophores, they do not differ remarkably; all exhibit similar luminescent charge-transfer states.

**Scheme 91:**
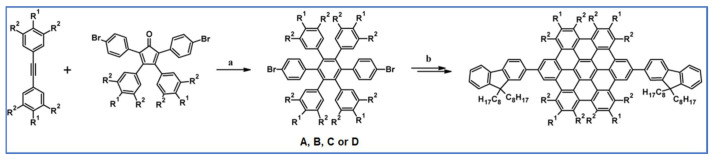
DA cycloaddition in the synthesis of hexa-peri-hexabenzocoronenes with electron withdrawing groups [145]. Reagents and conditions: **A**: R^1^ = R^2^ = H; **B**: R^1^ = F, R^2^ = H; **C**: R^1^ = CF_3_, R^2^ = H; **D**: R^1^ = H, R^2^ = F; a = Ph_2_O, 250 °C, 2 h, Y = not given (for **A**), Y = 65% (for **B**), Y = 82% (for **C**) and Y = 75% (for **D**); b = next steps.

Application of DA cycloaddition, followed by the oxidative cyclodehydrogenation of electron-poor arenes, resulted in hexa-peri-hexabenzocoronenes with electron withdrawing Br, F, and CF_3_ groups (Scheme 91) [145]. Such synthesized compounds represent a new family of materials for electron transport. One should emphasise that a new set of reaction conditions for the oxidative cyclodehydrogenation of electron-poor arenes has been developed.

It was stated that the presence of electron withdrawing groups, for example F or CF_3_, has a remarkable impact on the energies of frontier orbitals of HBC unit and facilitate the application of products as electron transport materials. Moreover, the utilization of the hexa-peri-HBC in organic electronics was also considered.

**Scheme 92:**
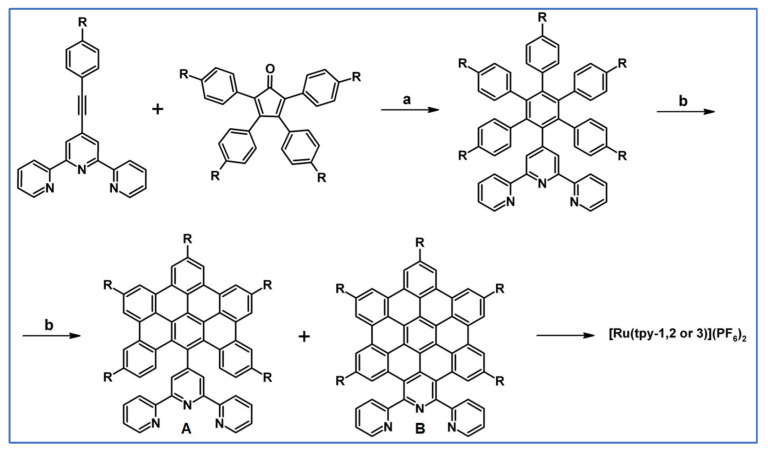
Tpy ligands syntheses using DA cycloaddition and Scholl reaction [146]. Reagents and conditions: R = *t*-Bu; a = Ph_2_CO, 280 °C, 24 h; Y = 56%; R = H or *t*-Bu; b = next step (Scholl reaction), Y = 34% (for **A**) and 5% (for **B**).

Graczyk and colleagues described in 2012 classical versions of DA cycloaddition with CO extrusion and Scholl dehydrocondensation which were engaged in the synthetic way, supplying 2,2′:6′,2′′-terpyridine (tpy) ligands (Scheme 92) [146].

Using tpy presented in the scheme above, ruthenium(II) complexes were also prepared via a typical manner. Unfortunately, it turned out that the obtained complexes have similar spectroscopic properties to the simple [Ry(tpy)_2_]^2+^.

**Scheme 93:**
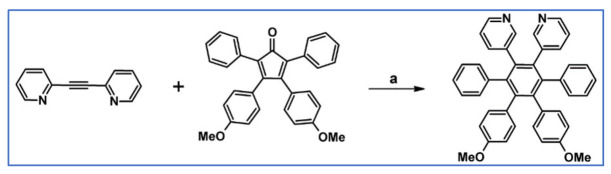
Synthesis of double-N-doped HPB via DA cycloaddition with CO extrusion [147]. Reagents and conditions: a = Ph_2_O, 240 °C, 18 h, Y = 70%.

Bhalla et al., reported in 2012 synthesizing N-doped, hetero-oligophenylene via DA cycloaddition with CO extrusion involving 1,2-bis(3-pyridyl)acetylene and suitable cyclopentadienone derivative (Scheme 93) [147].

Interestingly, it was shown that the discussed N-doped HPB easily create aggregates, which in H_2_O + THF solution exhibits AIEE phenomenon. Moreover, these aggregates can be used as a selective biological probe for particular proteins and the indication of some metal ions. Namely, Pb^2+^ and Pd^2+^ ions can be selectively determined, despite the fact that almost 30 metal ions were tested.

**Scheme 94:**
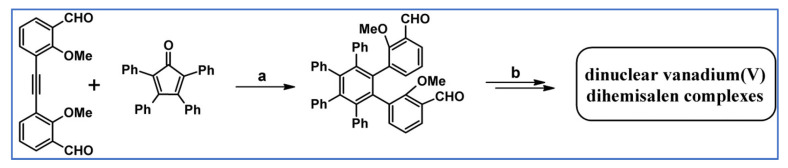
Synthesis of ligand precursor for vanadium(V) dihemisalen complexes starting from DA cycloaddition of appropriate cyclopentadienone and acetylene [148,149]. Reagents and conditions: a = Ph_2_O, rfx, 8 h (the mixture of cis and trans isomers were formed); Y = not given (products were not isolated); b = next steps.

As Miyasaka, Amaya, and Hirao reported in 2012 and continued in 2014, the hexaarylbenzene scaffold for the dinuclear vanadium(V) dihemisalen complexes were designed and synthesized involving DA cycloaddition (Scheme 94) [148,149].

These complexes were examined as promotors for the reductive coupling reaction between aliphatic and aromatic aldehydes (in the presence of Me_3_SiCl and Zn), which provided the corresponding cross-coupled 1,2-diols in good yields and with high cross-selectivity.

**Scheme 95:**
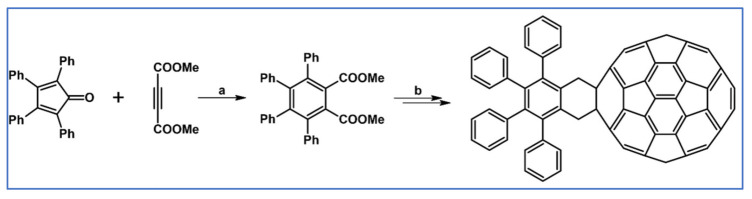
DA cycloaddition as an initial step in synthesizing [60]fullerene derivative bearing tetra(phenyl)phenyl motif [150]. Reagents and conditions: a = PhMe, heating.

In another paper from 2012, one of the synthesized *o*-quinodimethane adducts with [60]fullerene was obtained starting from DA cycloaddition of tetraphenylcyclopentadienone and dimethyl acetylene dicarboxylate (Scheme 95) [150].

Obtained adducts were examined as far as their intramolecular π-interactions between aryl and CH-fullerene motifs were concerned.

**Scheme 96:**
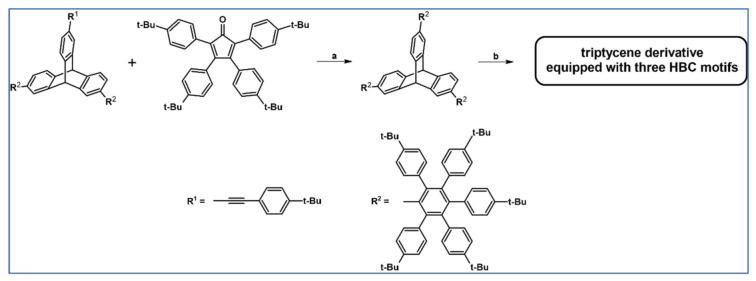
Synthesis of three dimensional tripticene derivative equipped with HBC moieties via DA cycloaddition with CO extrusion-Scholl reaction tandem [151]. Reagents and conditions: a = Ph_2_O, rfx, 48 h, Y = 43%; b = Scholl dehydrocondensation.

In 2012, Zhang and colleagues reported an example of DA cycloaddition followed by Scholl dehydrocyclocondensation that was engaged in the synthetic procedure providing tripticene derivative possessing three HBC motifs (Scheme 96) [151].

The obtained 3D nanographene showed intrinsic fluorescence and, after appropriate functionalization, can be used as in vivo and in vitro bioimaging agent characterized by low toxicity and good anti-photobleaching properties.

**Scheme 97:**
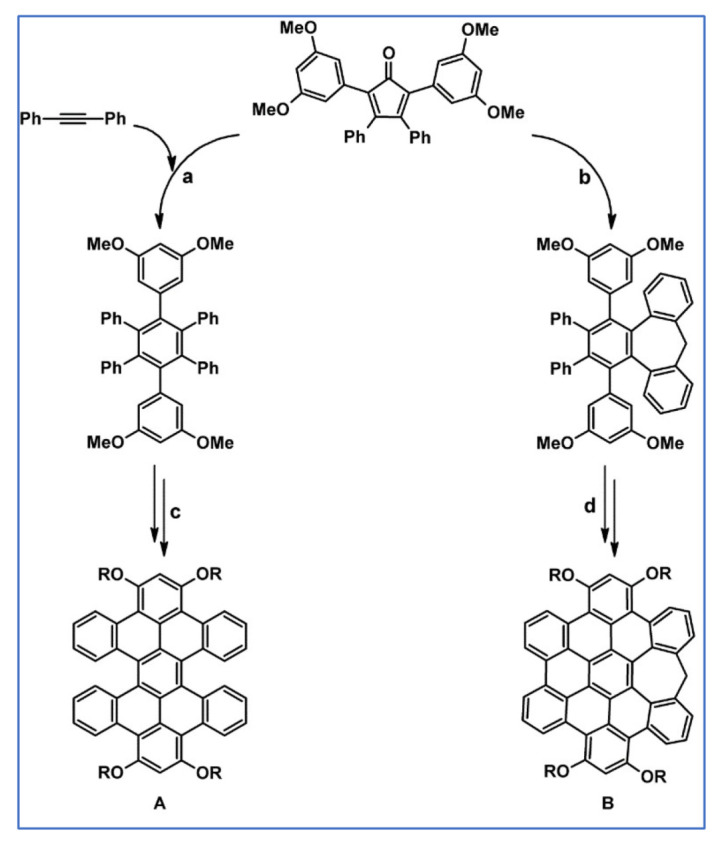
Syntheses of curved HBP and HEHBC using [4 + 2] cycloaddition and Scholl-type dehydrocondensation [152]. Reagents and conditions: a = Ph_2_O, rfx, Y = 83%; b = *t*-BuOK, Et_2_O, rt, Y = 32%; c, d = next steps (Scholl reaction).

Two curved functionalised PAHs, namely hexabenzoperylene (HBP) and heptagon-embedded hexabenzocoronene (HEHBC), were obtained by Luo et al., in 2012 using DA cycloaddition with CO extrusion, followed by partial or complete Scholl dehydrocondensation (Scheme 97) [152].

In the synthesis of (B), dienophile, i.e., alkyne, was generated in situ from 10-bromo-5*H*-dibenzo[*a,d*]cycloheptene. Interestingly, synthetical success was possible due to the presence of alkoxy group in the appropriate position in the HPB core. The curvature of the commented nanostructures is forced either by a fused seven-membered ring or by steric repulsion in the bay region. The influence of the curvature on the properties of the synthesized compounds was thoroughly examined. Chirality and isomerization of twisted isomers, π–π interactions, semiconducting and insulating properties, photophysical and electrochemical properties, and influence of curvature on frontier molecular orbitals were analyzed. The reference structures for crowded compounds were their π-isoelectronic planar analogues.

**Scheme 98:**
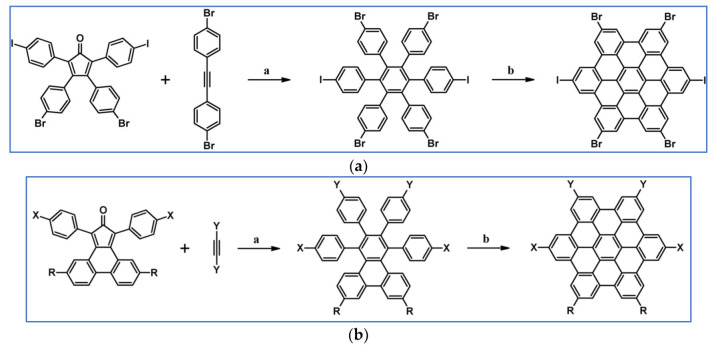
DA cycloaddition of cyclopentadienones and diarylacetylenes for the synthesis of nanographenes’ precursors [153]. Reagents and conditions: (**a**) a = Ph_2_O, rfx,14 h, Y = 59%; b = next step (Scholl reaction), Y = 86%; (**b**) (a) Y = R = H, X = Br; (b) Y = R = H, X = I; (c) Y = H, R = I, X = Br; a = Ph_2_O, rfx, 1–20 h, up to 91% yield; b = next step (Scholl reaction).

Another notable reaction is DA cycloaddition with CO extrusion used in the synthetic route, leading to monomers for graphene nanoribbons, wires, and graphene-type networks with predefined graphene structure to minimize the number of structural defects (Scheme 98) [153].

**Scheme 99:**
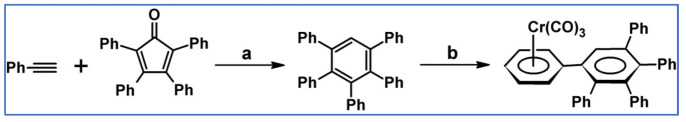
Ph_5_Ph obtained via DA cycloaddition as a ligand in the [Cr(CO)_3_L] complex; L = η^6^–P–(PhPh_4_) [154]. Reagents and conditions: a = Ph_2_O, 210 °C; b = next step.

Pentaphenylbenzene obtained via DA cycloaddition with CO extrusion and used as a ligand for synthesising the (CO)_3_chromium(0)-η^6^-arene complex (Scheme 99) was described by Brydges and colleagues in 2013 [154].

**Scheme 100:**
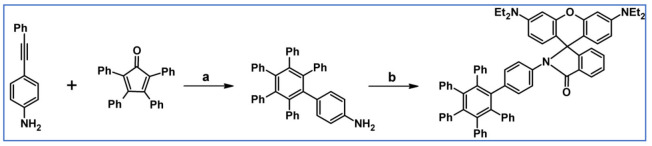
DA cycloaddition involved in the synthesis of HPB derivative possessing one rhodamine B motifs [155]. Reagents and conditions: a = Ph_2_O, rfx, overnight, Y = 60%; b = next steps.

In 2013, Bhalla et al., reported applying DA cycloaddition and Co-catalyzed cyclotrimerization in the syntheses of HPB derivatives possessing one, two (DA cycloaddition) or six (cyclotrimerization) rhodamine B moieties (Scheme 100) [155].

Interestingly, the authors claimed that HPB equipped with two or six rhodamine B substituents demonstrate fluorescence when Hg^2+^ ions are present in the solution. On the contrary, the presence of other metal ions does not cause energy transfer. Moreover, the energy transfer occurs through space or via bonds in aprotic or protic solvents, respectively.

**Scheme 101:**
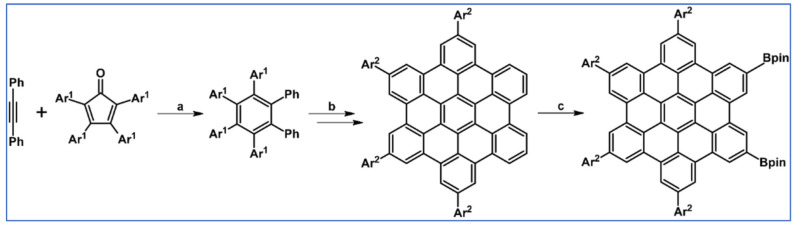
HBC functionalization via cycloaddition-Scholl reaction-direct borylation [156]. Reagents and conditions: Ar^1^ = 4-bromophenyl, Ar^2^ = mesityl; a = Ph_2_O, 200 °C, 36 h; Y = 93%; b = next steps; c = Ir-catalyzed borylation.

In the case described by Yamaguchi and his team in 2013, DA cycloaddition with CO extrusion was an initial step in the synthetic procedure leading to functionalized HBC (Scheme 101) [156]. It is worth adding that regioselective direct borylation catalysed by iridium complexes is especially interesting. This is due to the fact that HBC containing Bpin motifs can be widely utilized for further transformations.

**Scheme 102:**
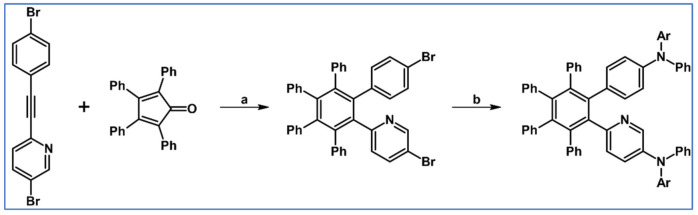
Preparation of HPB-derivatives bearing triaryl- and 2-pyridyl moieties via DA cycloaddition [157]. Reagents and conditions: Ar = Ph, 1-naphthyl, 2-naphthyl; a = Ph_2_O, rfx, 48 h, Y = 71%; b = next step, yield up to 24%.

The same year, Kim’s research team reported another case of DA cycloaddition with CO extrusion that were involved in the syntheses of HPB-derivatives dedicated to hole-transporting in OLEDs (Scheme 102) [157].

Properties of tested devices constructed using the synthesized materials were as follows: luminance: up to 3.72 cd/A; external quantum efficiency: up to 1.29% (10 mA/cm^2^); and glass transition temperature: up to 133 °C.

**Scheme 103:**
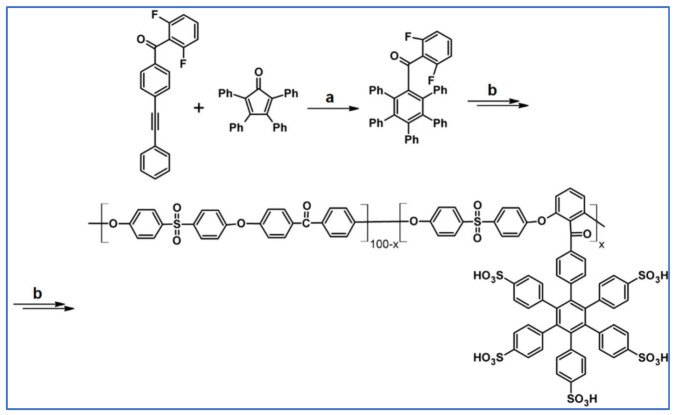
DA cycloaddition with CO extrusion involved in the synthetic way leading to polymeric ionomers bearing pentasulfonated-HPB moiety [158]. Reagents and conditions: a = sulfolane, 250 °C, 3 h, Y = 90%.

Another example of the use of DA cycloaddition with CO extrusion is the creation of HPB-core of polymeric ionomers based on a wholly aromatic poly(arylene-ether) backbone with locally pentasulfonated pendent groups has been described by Pang et al., (Scheme 103) [158].

Membranes made from the obtained polymers have many advantages compared to the generally known ones prepared with Nafion-17. Namely, they are hard, transparent, and flexible.

**Scheme 104:**
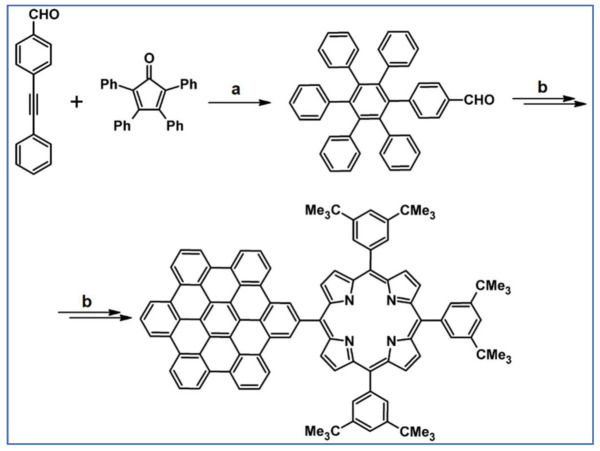
DA cycloaddition followed by Scholl reaction in the synthesis of porphyrin bearing HBC motif [159]. Reagents and conditions: a = MW, neat, 240 °C, 0.5 h, Y = 60%; b = next steps.

In 2013 Englert and colleagues presented DA cycloaddition combined with Scholl reaction as pivotal steps in syntheses leading to photo/redox active porphyrin directly connected with the HBC unit (Scheme 104) [159].

The obtained HBC-porphyrin can play the role of a prototype carbon-rich material with two photo/redox active and electronically interrelating groups.

**Scheme 105:**
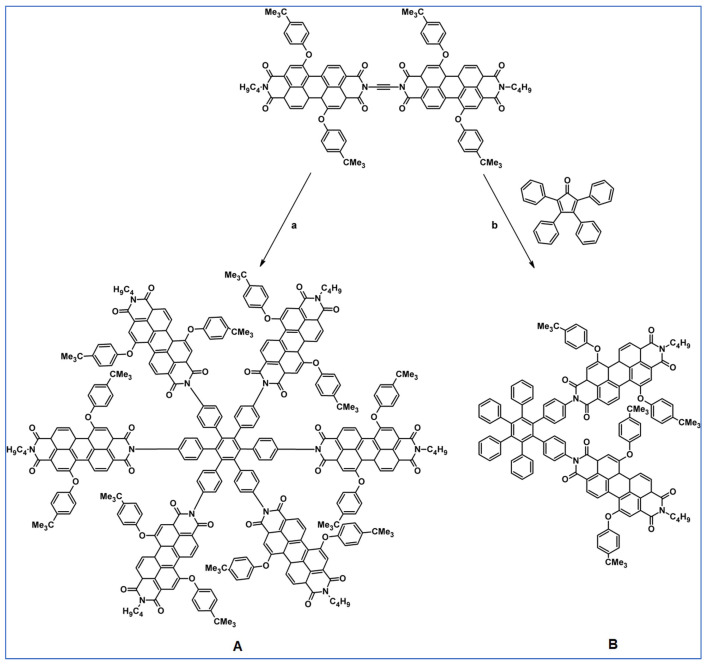
Syntheses of HPB derivatives bearing two or six PBI-moieties via DA cycloaddition or Co-catalyzed cyclotrimerization, respectively [160]. Reagents and conditions: a = dioxane, [Co_2_(CO)_8_], rfx, 12 h; b = Ph_2_O, rfx, 12 h; Y = 5% (for **A**) or 4% (for **B**).

The same year, Xue and colleagues described DA cycloaddition and Co-catalyzed cyclotrimerization used in the syntheses of perylenetetracarboxylic diimide hexamer (**A**) and dimer (**B**) linked with the same hexaphenylbenzene core (Scheme 105) [160].

Interactions between the PBI subunits in A are stronger than in B, as there is a greater steric hindrance in A and, consequently, rotation of PBI motifs along the molecular long axis is blocked. For comparison, these motifs in product B can freely rotate. For this reason, the photophysical and electrochemical properties of **A** and **B** vary considerably.

**Scheme 106:**
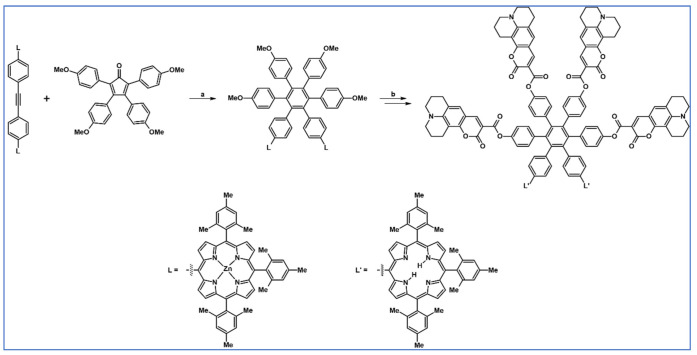
Synthetic route, involving DA cycloaddition, for the preparation of hexad and heptad [161]. Reagents and conditions: a = Ph_2_O, rfx, 5 h, Y = 86%; b = next steps.

DA cycloaddition between acetylene bearing two ZnPhC motifs and tetraphenylcyclopentadienones, leading to molecular hexad and heptad with two porphyrins scaffolds and four coumarin-based antenna systems attached to hexaphenylbenzene core, was presented by Garg et al., (Scheme 106) [161].

Notably, the efficiency of the energy transfer from coumarins motifs to porphyrin centre is quantitative. Moreover, creating a heptad antenna-reaction centre complex was observed due to the self-assembling of pyridyl-equipped fullerene motifs.

**Scheme 107:**
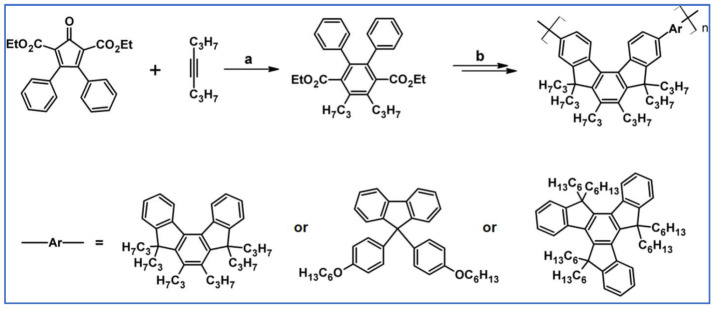
Synthesis of twisted fluorene-containing polymers starting from DA cycloaddition with CO extrusion [162]. Reagents and conditions: a = mesitylene, 180 °C, 24 h, Y = 50%; b = next steps.

As reported by Du and his team in 2013, DA cycloaddition with CO extrusion could also be involved in the multi-step synthetic procedure giving indeno[2,1-*c*]fluorene-based blue fluorescent oligomers and polymers (Scheme 107) [162].

Using single-crystal analysis, the authors showed that the structure of the indeno[2,1-*c*]fluorene is twisted. Such geometry is prominent as close H-H contacts occur in the crowded area of the molecule. Studies have shown that in dilute solution and thin film, all obtained polymers emit a strong blue fluorescence with narrow full width at half maximum (FWHM)—about 50 nm. Photophysical and electroluminescence investigations of obtained materials revealed that indeno[2,1-*c*]fluorene motif could be a potential building block for blue and UV light-emitting devices.

**Scheme 108:**
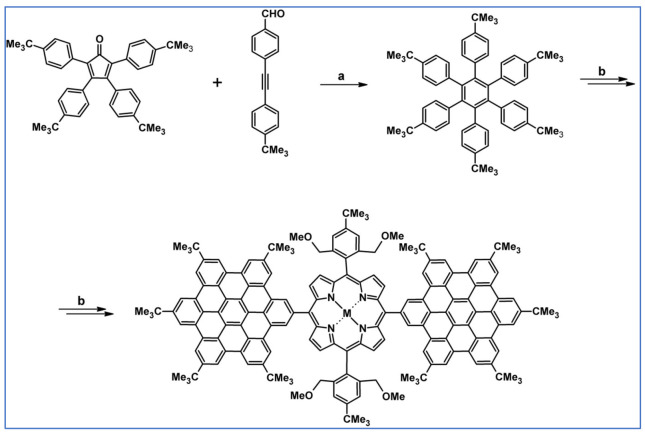
DA cycloaddition between tetraphenylcyclopentadienone and adequately substituted acetylene as a starting step in the procedure leading to HBC-porphyrin and its Zn-complex [163]. Reagents and conditions: M = 2H, Zn; a = Ph_2_O, rfx, 20 h; Y = 75%; b = next steps.

In 2014 Lungerich et al., proposed the synthesis pathway of free-base porphyrin with two HBC moieties and its Zn^2+^ derivative, starting from DA cycloaddition between appropriately substituted cyclopentadienone and acetylene (Scheme 108) [163].

Importantly, due to the strong electron transfer between the two chromophores, HBC-porphyrin conjugate exhibits extraordinary absorption and emission, making them potential building blocks for carbon-rich supramolecular functional architectures in the field of organic electronics.

**Scheme 109:**
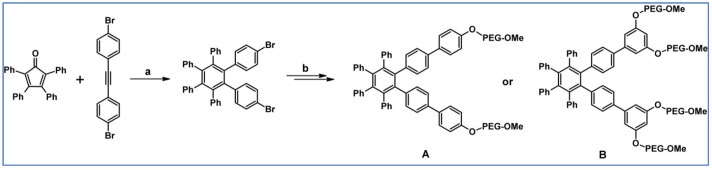
HPG-PEG syntheses via DA cycloaddition-pegylation strategy [164]. Reagents and conditions: a = Ph_2_O, 220 °C, MW (300 W), 12 h, Y = 45%; b = next steps. A = PEG-750; B = PEG-380.

DA cycloaddition with CO extrusion was applied by Wunderlich et al., as an initial step in the synthetic route leading to the HPB-PEG derivatives (Scheme 109) [164].

Importantly, in dilute aqueous solutions, the final compounds are capable of forming hydrogel fiber bundles. Interestingly, the water content of these fibers does not depend on the number of hydrophilic groups in molecule, but on the length of PEG chains and the precursor’s substitution.

**Scheme 110:**
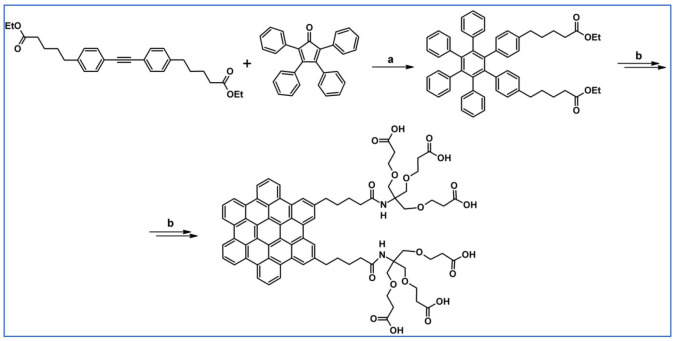
DA cycloaddition engaged in the synthesis of amphiphilic hexa-peri-hexabenzocoronene derivative [165]. Reagents and conditions: a = Ph_2_O, 230 °C, 24 h, Y = 61%, b = next steps (i.a. Scholl reaction).

As reported by Carta and colleagues in 2014, DA cycloaddition with CO extrusion was involved in the multistep synthetic procedure leading to a water-soluble surfactant consisting of hexa-peri-hexabenzocoronene (HBC) hydrophobic aromatic core and hydrophilic carboxy substituents (Scheme 110) [165].

The obtained surfactant exhibited a self-assembled nanofiber structure in the solid state. Profiting from the π interactions between the sizeable aromatic core of HBC and graphene, the surfactant mediated the exfoliation of graphite into graphene in polar solvents, which the bulky hydrophilic carboxylic groups further stabilized.

**Scheme 111:**
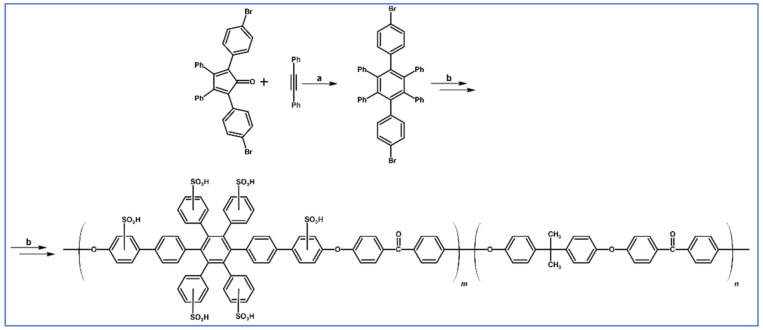
DA involved in the synthesis of monomer for sulfonated, multi-phenylated polyarylene ethers; structure of copolymer with 4,4′-difluorobenzophenone and bisphenol A [166]. Reagents and conditions: a = Ph_2_O, 220 °C, 3 days; b = multistep reaction.

Lee et al., described DA cycloaddition with CO extrusion as initial step in the synthesis of sterically encumbered sulfonated, poly(arylene ether) copolymers (Scheme 111) [166].

As for the parameters of membranes obtained from the described copolymers, they are as follows: (a) ion exchange capacities: 1.9 to 2.7 mmol/g; (b) proton conductivities: 0.24 to 16 mS/cm at 80 °C/60% RH, and 3 to 167 mS/cm at 80 °C/95% RH. In addition, the membranes are characterised by excellent mechanical strength.

**Scheme 112:**
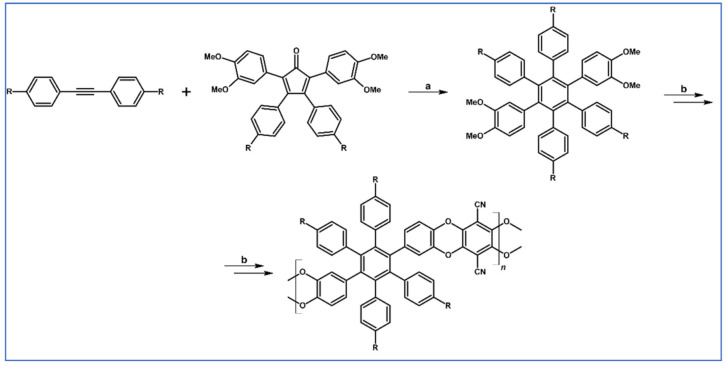
DA cycloaddition as a crucial step in the synthetic procedure reaching of hexaphenylbenzene based polymers of intrinsic microporosity [167]. Reagents and conditions: R = H, Br, Me; a = Ph_2_O, 270 °C, MW; b = next steps.

In 2014, Carta’s research team reported the synthesis of HPB-based polymers of intrinsic microporosity with methyl, bromine, and nitrile substituents, obtained via DA cycloaddition with CO extrusion (Scheme 112) [167].

Notably, the modification of polymer structures by introducing the polar groups allows for effective tuning of membranes properties. In particular, it increases the selectivity towards specific gases, especially carbon dioxide.

**Scheme 113:**
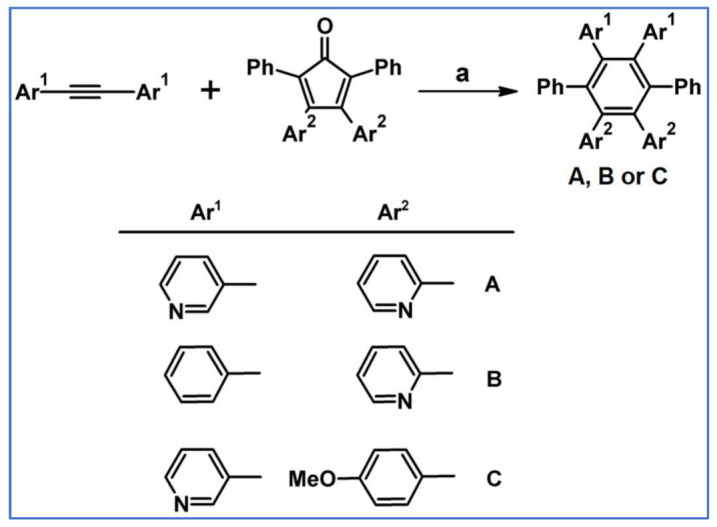
DA cycloaddition as a synthetic tool for the syntheses of N-doped HPBs [168]. Reagents and conditions: a = Ph_2_O, rfx, 18 h, Y = 70% (for **A**), 65% (for **B**), and 71% (for **C**).

As reported by Kaur in 2014, N-doped hetero-oligophenylene derivatives were obtained via DA cycloaddition with CO extrusion, engaging vaired derivatives of acetylene and cyclopentadienone (Scheme 113) [168].

In an aqueous solution, oligothiophene **C** forms fluorescent aggregates that can be used in a selective sensor for picric acid disclosing, both in solid state and in solution.

**Scheme 114:**
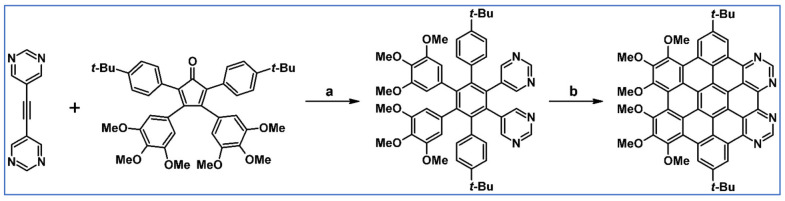
Synthesis of methoxy-functionalized N-containing heterosuperbenzenes via DA cycloaddition–Scholl reaction—an example [169]. Reagents and conditions: a = Ph_2_CO, rfx, 1 h, Y = 82%; b = next step (Scholl reaction).

Another notable reaction tandem, described by Wijesinghe’s research team, is a synthesis of a series of methoxy-functionalized N-containing heterosuperbenzenes via DA cycloaddition, Scholl reaction, and appropriately substituted dienes and dienophiles, as illustrated in Scheme 114 [169].

It was discovered that the role of methoxy substituents is critical in the adjustment of electronic properties, cyclodehydrogenation promotion, and maintenance of π–π stacked supramolecular order of the products. Moreover, the presence of MeO group destabilizes the HOMO orbital, while nitrogen atoms stabilize the LUMO; in consequence, the HOMO–LUMO gap is reduced.

**Scheme 115:**
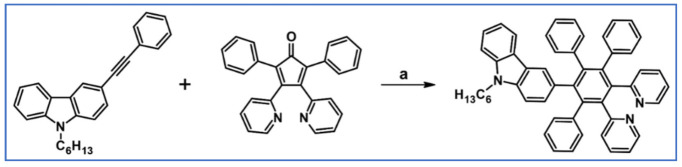
Synthesis of N-doped HPB derivative via DA cycloaddition involving appropriately substituted acetylene and cyclopentadienone [170]. Reagents and conditions: a = Ph_2_O, 240 °C, 24 h, Y = 72%.

N-doped heterooligophenylene possessing carbazole and 2-pirydyl moieties can be obtained via DA cycloaddition with CO extrusion, as described by Kaur et al., in 2014 and depicted in Scheme 115 [170].

Interestingly, the obtained hexaarylbenzene equipped with two pyridine and one carbazole moieties can play the super-effective threonine detectors in aqueous solution and the presence of Zn^2+^ ions.

**Scheme 116:**
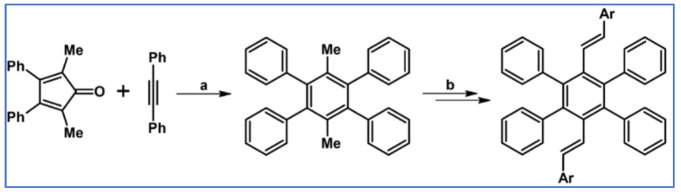
DA cycloaddition of cyclopentadienene and diphenylacetylene as an initial step in the syntheses of 1,4-distyryl-2,3,5,6-diphenylbenzenes [171]. Reagents and conditions: Ar = Ph, 3,4-(CF_3_)_2_Ph, *p*-CH(OEt)_2_Ph, *p*-(CHO)Ph, *p*-Nbu_2_Ph; a = xylene, 200 °C. MW, Y = 85%; b = next steps.

Freudenberg, Rominger, and Bunz reported obtaining a series of novel tetraphenyl-1,4-distyrylbenzenes via DA cycloaddition of diphenyldimetylcyclopentadienone and diphenylacetylene (Scheme 116) [171].

Notably, the selected obtained compounds show attractive aggregation-induced emission. Moreover, the attachment of electronically active functional groups, such as aldehyde or dialkylamino units, changes the character of the AIE fluorophore. Namely, in these species, emission from the dilute solution is also possible, while the electronically unperturbed, that is, only hydrocarbon AIEs, emit in the agglomerated state.

**Scheme 117:**
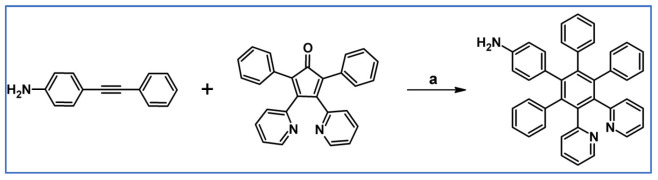
Synthesis of N-doped HBC using DA cycloaddition with CO extrusion [172]. Reagents and conditions: a = Ph_2_O, rfx, 24 h; Y = 78%.

DA cycloaddition was used by Walia et al., as a synthetic tool to prepare an N-doped oligophenylene derivative, whose aggregates can act as reactors and stabilizers for the generation of palladium nanoparticles in the Sonogashira coupling under aerial conditions, at room temperature (Scheme 117) [172].

**Scheme 118:**
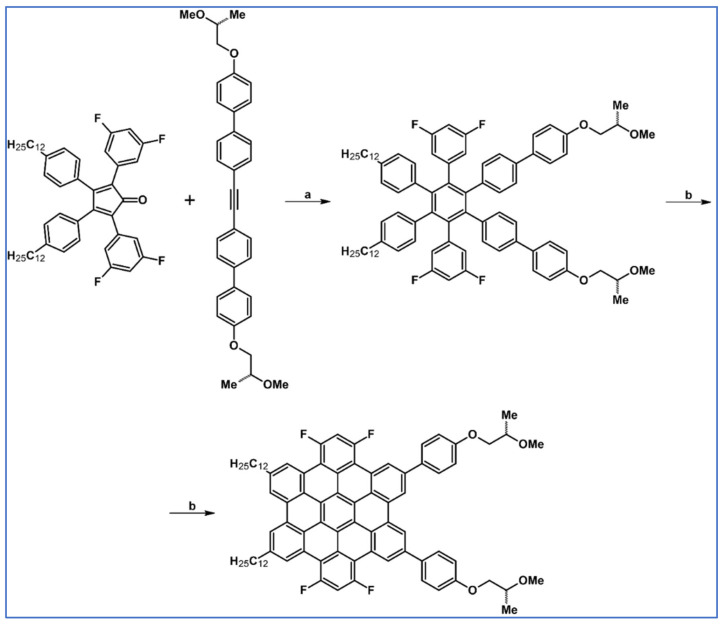
Racemic and chiral HBCs syntheses via DA cycloaddition-Sholl reaction tandem (an example) [173]. Reagents and conditions: a = Ph_2_O, rfx, 18 h, Y = 79% (for I-isomer), Y = 72% (for (S)-isomer); b = next step (Scholl reaction).

As reported by Zhang’s research group in 2016, racemic and chiral HBC derivatives were obtained via DA cycloaddition with CO extrusion followed by Scholl reaction and then were used as monomers for supramolecular polymerization (Scheme 118) [173].

The obtained HBCs, including the chiral version possessing stereogenic C-atom in the oligoether fragment (instead of the OR fragment presented in the scheme), were applied as monomers, and nanotubes were created via assembly or co-assembly.

**Scheme 119:**
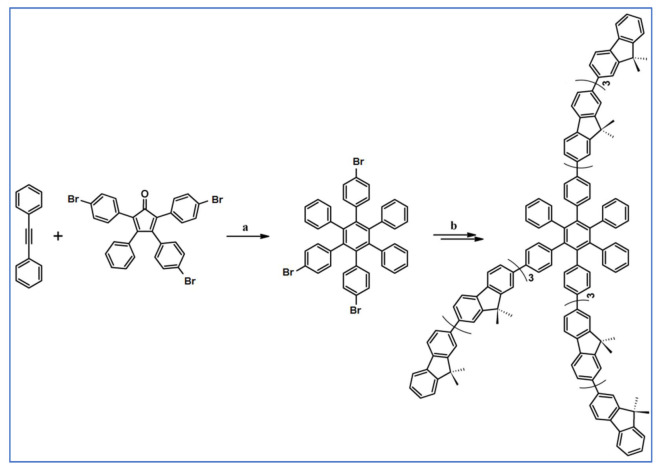
DA cycloaddition for the synthesis Br_3_HPB—an example [174]. Forming nanostructures from Br_3_HBC and DBTF on Au(111). Reagents and conditions: a = Ph_2_O, 260 °C, Y = 43%; b = next steps.

Well-defined molecular wires-type oligomeric structures organized around the polyphenyl node were presented by Nacci et al. The DA cycloaddition of appropriately substituted cyclopentadienones and acetylenes was involved in those multi-step procedures (Scheme 119) [174].

By depositing Br_3_HBC and DBTF molecules onto Au-(111) and subsequently heating the sample to 523 K, the authors succeeded in forming nanostructures from the obtained molecules.

**Scheme 120:**
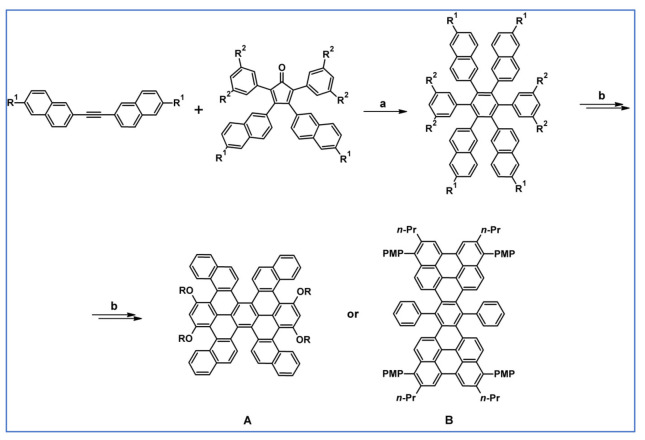
DA cycloaddition and Scholl reaction as crucial steps leading to twisted polycyclic arenes [175]. Reagents and conditions: a = Ph_2_O, rfx, 48–72 h; Y = 21% (for R^1^, R^2^ = *n*-Pr, H) or 68% (for R^1^, R^2^ = H, OMe); b = next steps (i.a. Scholl reaction); **A**: R = *n*-Pr (Y = 8%) or *n*-hexyl (Y = 13%); **B**: PMP—*p*-methoxyphenyl (Y = 60%).

Twisted polycyclic arenes with constitutionally isomeric π-backbones were reported by Yang and colleagues in paper form 2016. Compounds mentioned above were synthesized involving DA cycloaddition with CO extrusion followed by controlled Scholl reaction of 1,2,4,5-tetaphthahth-2-yl)-3,6-diphenylbenzene (cycloaddition product) with properly positioned electron-donating substituents (Scheme 120) [175].

Especially important is that the thin films of product B are insulating, which is probably caused by limited π-π-stacking, while the twisted and anti-**A** containing the *n*-hexyl substituent processed TFT behave in solution like *p*-type semiconductors.

**Scheme 121:**
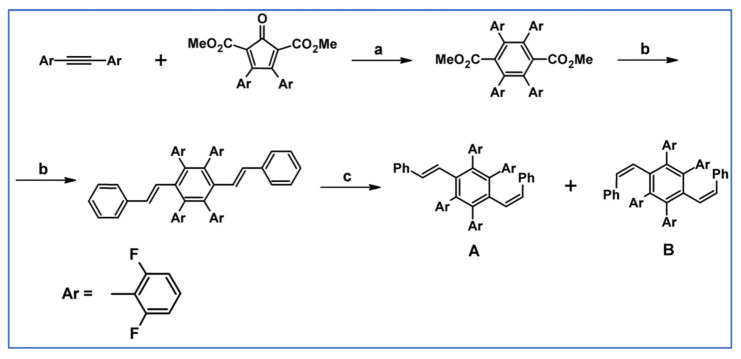
DA cycloaddition as a crucial step in the synthetic procedure leading to 1,4-di-styrylbenzene [176]. Reagents and conditions: a = MW, 250 °C, 1 h, Y = 86%; b, c = next steps.

Again, in 2016, Freudenberg, Rominger, and Bunz presented DA cycloaddition with CO extrusion as a crucial step in the construction of 2,3,5,6-tetrakis(2,6-difluorophenyl)-1,4-di(styryl)benzene core (Scheme 121) [176].

In-depth photophysical studies revealed that the obtained compound does not undergo photo-induced electrocyclization due to the steric effect of fluorine substituents. Furthermore, AIE and photostability make it attractive emitter and building block for manufacturing materials dedicated to organic electronics.

**Scheme 122:**
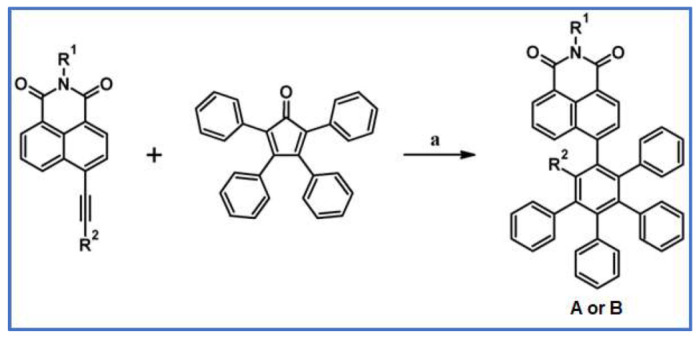
DA cycloaddition for the synthesis of new type of dyes [177]. Reagents and conditions: **A**: R^1^ = *n*-Bu, R^2^ = Ph; **B**: R^1^ = *n*-Bu, R^2^ = SiMe_3_; a = Ph_2_O, rfx, 10 h, Y = 60% (for **A**) or a = Ph_2_O, rfx, 30 h, Y = 62% (for **B**).

Wang et al., obtained two novel dyes derived from 1,8-naphthalimide using DA cycloaddition of appropriate derivatives of naphthalimide and tetrapenylcyclopentadienone (Scheme 122) [177].

The two dyes showed unique AIEE characteristics, namely displayed excellent optical properties, such as solvent-induced emission changes from deep blue to light green, and the sensitive fluorescence response to nitroaromatic explosives. Interestingly, an unexpected SiMe_3_ effect was found: the SiMe_3_-containing compound **B** exhibit remarkably enhanced optical properties compared with the non-SiMe_3_ compound **A**.

**Scheme 123:**
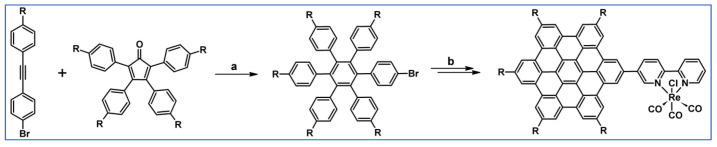
DA cycloaddition as an initial step in the synthetic way leading to the chloro-tri(carbonyl)-*Re*(I) complex bearing HBC-bpy ligand [178]. Reagents and conditions: a = Ph_2_O, rfx, 18 h; R = *t*-Bu (Y = 62%) or C_12_H_25_.

In 2016, Elliot et al., published results concerning the metallographene hybrids Re(I) complexes bearing alkyl-substituted HBC-bpy ligand, obtained via DA cycloaddition with CO extrusion followed by Scholl reaction, Suzuki–Miyaura coupling, and finally complexation using [ReCl(CO)_5_] as a *Re*(I) source (Scheme 123) [178].

Significantly, photophysical properties of transition metal complexes with HBC with bpy motifs can vary in a wide range, incomparably wider compared to HBC without a complexing fragment.

**Scheme 124:**
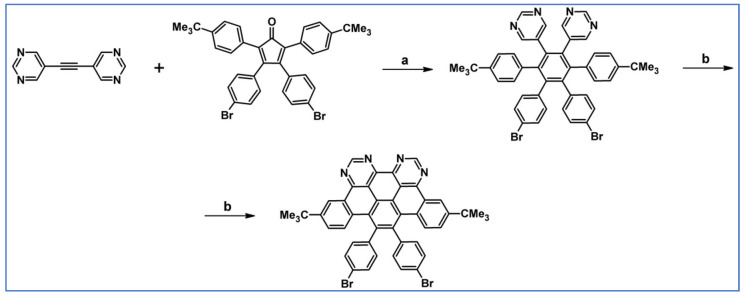
DA cycloaddition with CO extrusion and partial cyclodehydrocondensation as crucial steps in the synthesis of N-doped nanographene [179]. Reagents and conditions: a = 280 °C, 3 h, Y = 85%; b = next step.

As stated in another paper from 2016, DA cycloaddition with CO extrusion and intramolecular oxidative cyclodehydrogenation were initial steps in the synthetic route, giving rise to a family of partially fused, selectively brominated N-doped pyrimidine graphenes (Scheme 124) (selected synthesis is presented) [179].

The N-doped nanographene shown in the scheme above is essential for obtaining ortho-metalated or N-coordinated transition metal complexes with interesting photophysical applications.

**Scheme 125:**
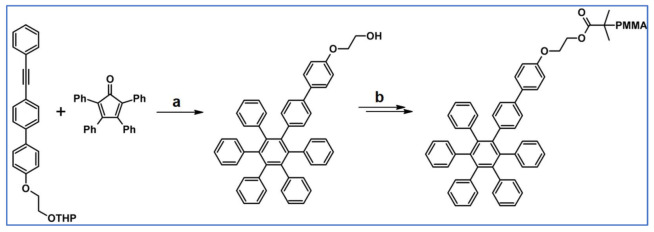
DA cycloaddition and Scholl reaction used for creation of functionalised HBC-motif in the synthesis of poly(methyl methacrylate) end-functionalized with HBC [180]. Reagents and conditions: a = 150–210 °C, 1 h, Y = 33%.

In 2016, Jeong et al., published an article devoted to synthesizing poly(methyl methacrylate) end-functionalized with HBC unit. The final product was obtained by applying the tandem of DA cycloaddition with CO extrusion and Scholl reaction (Scheme 125) [180].

The additive concentration based on hexabenzocoronene subunit required to disperse the multi-walled carbon nanotubes was significantly lower than the concentration needed for existing additives.

**Scheme 126:**
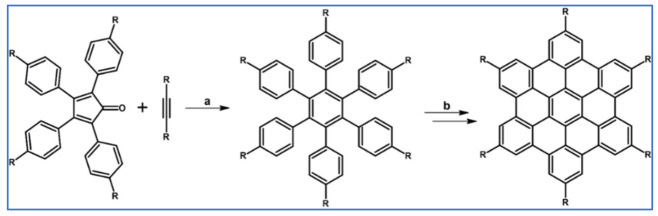
Oxidative coupling of hexaphenyl benzenes under electrochemical conditions [181]. Reagents and conditions: R = H, OMe, Me, *t*-Bu; a = heating; b = next step (Scholl reaction).

As claimed by Rçse and colleagues, hexaaryl benzene can be obtained by DA cycloaddition (with CO extrusion) or diarylacetylene catalytic cyclotrimerization. It should be emphasized that the second method can be used only for symmetric derivatives. Hexaaryl benzenes obtained via the presented above method can be transformed into coronene derivatives using Scholl reaction. In 2017, electrochemical variant of the above-mentioned intramolecular dehydrogenative carbon–carbon bond formation was described (Scheme 126) [181].

Interestingly, the Scholl reaction was realized in the electrochemical version, namely via indirect anodic oxidation with DDQ as a redox mediator turned out to be very effective. It is worth adding that in the electrochemical method, the required amount of DDQ was 1000 times less than the amount that was used in a classical stoichiometric variant.

**Scheme 127:**
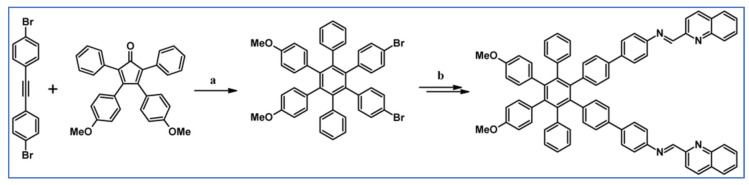
DA cycloaddition as a crucial step in the synthesis of HPB derivative wearing two imine-quinoline motifs [182]. Reagents and conditions: a = Ph_2_O, 240 °C, 24 h, Y = 70%.

S. Pramanik et al., obtained the derivative of HPB bearing two imine-quinoline motifs able to selectively complex Zn^2+^ ions [182]. The crucial step of the multistep synthesis was the Diels–Alder reaction of 1,2-bis(4-bromophenyl)ethyne with 3,4-bis(4-methoxyphenyl)-2,5 diphenylcyclopenta-2,4-dienone (Scheme 127).

**Scheme 128:**
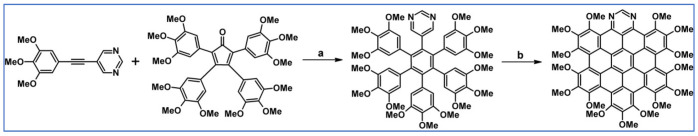
Synthesis of N-doped PAH using DA cycloaddition and Scholl cyclodehydrocondensation [183]. Reagents and conditions: a = Ph_2_O, 220 °C, 9 h, Y = 47%; b = next step (Scholl reaction).

It is worth mentioning that the ligand shown in Scheme 128 exhibits aggregation induced emission enhancement (AIEE) characteristics and undergoes fluorescence enhancement in the presence of only Zn^2+^ ions in aqueous media (different metal ions were tested). Importantly, the in situ synthesized Ligand–Zn^2+^ combination was employed as a valuable tool for selective detection of pyrophosphate ions in aqueous media.

Shi et al., synthesized N-doped specifically curved hetero-HBC via DA cycloaddition with CO extrusion followed by Scholl reaction (Scheme 128) [183].

X-ray analysis has shown that the double-concave conformation of the above-mentioned N-doped nanographene is due to peripheral methoxy groups and pyrimidine ring.

**Scheme 129:**
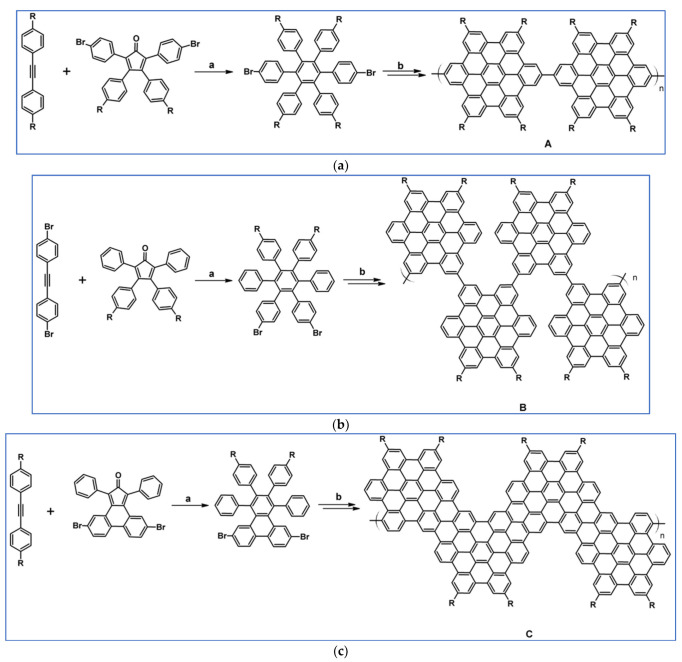
Polymeric-PAHs syntheses using APEX strategy DA cycloaddition with CO extrusion, aryl bromide coupling and Scholl reaction [184]. Reagents and conditions: R = C_4_H_9_; (**a**–**c**): a = 240–280 °C, 1 h, Y = 81% (for **A**) and 88% (for **B**) and 82% (for **C**); b = next steps.

In 2017, Chen’s research group reported synthesizing three types of polymeric-PAHs possessing *t*-butyl peripheral groups, starting from DA cycloaddition, followed by Ni(0)-catalyzed aryl bromide coupling and Scholl reaction (Scheme 129) [184].

The obtained polymeric PAHs (before Scholl reaction) were characterized by an increasing degree of planarity and decreasing bandgaps from P1 (**A** precursor) to P3 (**C** precursor), varied weight-average molecular weights, namely 9000 (P1), 5500 (P2), and 69,000 (P3), respectively, and intermolecular π–π stacking. As far as polymers after Scholl dehydrocondensation are concerned, the increase in the differences between bandgaps and oxidation onset from **A** to **C** results from the variability of planarity degree. All properties of obtained polymeric-PAHs suggested their usefulness for OFETs.

**Scheme 130:**
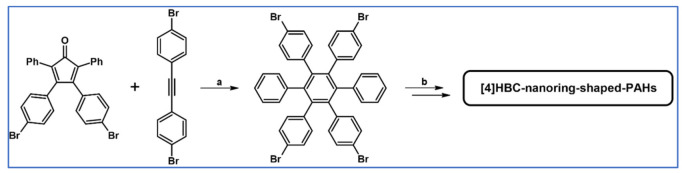
Synthesis of [4]HBC-nanoring-shaped-PAHs involving DA cycloaddition with CO extrusion as an initial step [185]. Reagents and conditions: a = Ph_2_O, rfx, 3 d, Y = not given; b = coupling and Scholl reaction.

The APEX-bottom-up strategy was employed by Lu et al., in 2017 in the synthetic route leading to a carbon nanoring-shaped-nanographene containing four HBC moieties equipped with mesityl-peripheral groups (Scheme 130) [185].

The obtained large π-expanded [4]HBC was characterized by unique photophysical properties and, especially, by forming 1:1 host-guest complex with fullerene C_70_.

**Scheme 131:**
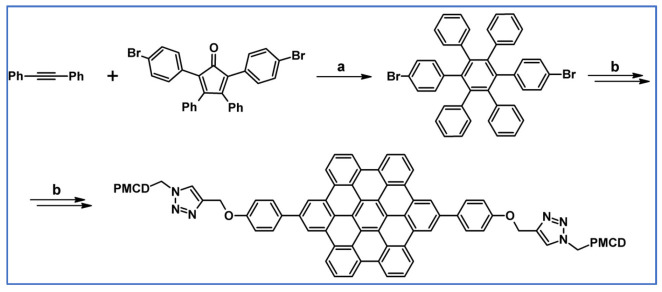
DA cycloaddition with CO extrusion, other transformations and Scholl reaction engaged in the synthesis of PMCD–L–HBC–L–PMCD [186]. Reagents and conditions: a = Ph_2_O, rfx, 24 h, Y = 88%.

The same year, Yu’s research group obtained HBC with two permethylated-β-cyclodextrin (PMCD) moieties and colleagues using DA cycloaddition with CO extrusion, further functionalization and Scholl reaction (Scheme 131) [186].

It is of particular importance that self-assembly of commented PMCD–L–HBC–L–PMCD molecule is characterized by specific fluorescence response as far as exploders are concerned. Precisely, detection of 2,4,6-trinitrophenol can be reached at 306 and 2 ppb in solution and on the test paper, respectively, what seems to meet practical requirements of exploder detectors.

**Scheme 132:**
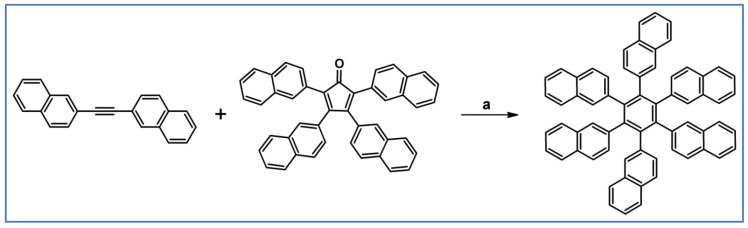
DA cycloaddition with CO extrusion: synthesis of Ph(2-naphthyl)_6_ [187]. Reagents and conditions: a = Ph_2_O, heating, 30–35 min, Y = 60%.

In another paper from 2017 Harrington and colleagues presented the synthesis of a molecular propeller, i.e., hexa(2-naphthyl)benzene via DA cycloaddition involving acetylene and cyclopentadienone bearing two and four 2-naphthyl moieties, respectively (Scheme 132) [187].

Similarly to *m*-substituted Ph-groups, the rotation barrier of 2-naphthyl substituents equals approximately 17 kcal/mol.

**Scheme 133:**
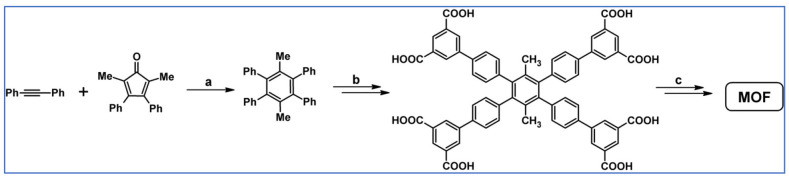
DA cycloaddition as an initial step in the synthetic procedure leading to MOF thin films [188]. Reagents and conditions: a = Ph_2_O, rfx, 2 h, Y = 81%.

Chenikova et al., described obtaining MOF-ligand starting from DA cycloaddition with CO extrusion followed by further functionalisation (Scheme 133) [188].

The presence of COOH groups in the ligand was decisive for successful and precise control of epitaxial growth, MOF-on-MOF oriented thin films characterized by highly crystalline form hierarchical porosity.

**Scheme 134:**
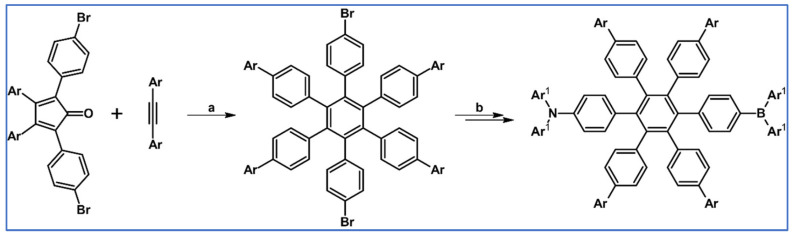
DA cycloaddition–Scholl reaction-amination and eventual borylation as crucial steps in the synthesis of D-π-A HBC derivative [189]. Reagents and conditions: Ar = 4-mesitylphenyl; a = Ph_2_O, rfx, 17 h, Y = 70%; b = next steps.

Luminescent HBC derivatives equipped with both donor (Ar_2_*N*) and acceptor (Ar_2_*B*) motifs were successfully synthesized by Kurata’s research team using DA cycloaddition with CO extrusion followed by Scholl reaction and further functionalization (Scheme 134) [189].

The obtained functionalized nanographene is characterized by significant dipole moment, remarkable solvatochromic emission and high fluorescence quantum yield untypical as far as another HBCs is concerned. Due to the properties mentioned above, the obtained compound can be attractive for optoelectronics.

**Scheme 135:**
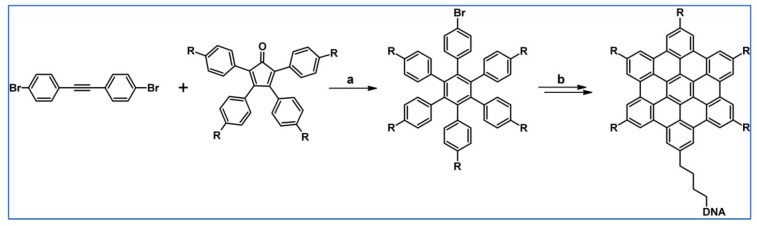
DA cycloaddition with CO extrusion followed by Scholl reaction as initial steps in the synthetic path providing HBC equipped with the DNA fragments [190]. Reagents and conditions: R = 3,7-dimethyloct-1-yl; a = Ph_2_O, 250 °C, 20 h, Y = 58%; b = next steps (i.a. Scholl reaction).

As reported in 2017 by Albert and colleagues, DA cycloaddition with CO extrusion was an initial step in the multistep synthetic path leading to HBCs bearing DNA fragment attached through *C_4_*-alkyl chain (Scheme 135) [190].

The presence of both hydrophobic (HBC moiety with alkyl chains) and amphiphilic fragments (DNA moiety) in the HBC-DNA structures results in a strong ability to self-assembly and finally leads to the creation of 2D micrometer-size sheets. Amphiphilic properties of DNA fragment mainly drive sheet creation, but π-stacking of HBC fragments and van der Waals interaction between alkyl chains are also important. In the presence of information-rich DNA fragments on the sheet surface, the considered structures can be attractive in catalysis, optoelectronics, and molecular recognition.

**Scheme 136:**
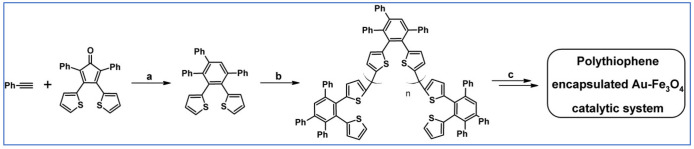
DA cycloaddition with CO extrusion as an initial step in the synthetic route leading to bimetallic Au-Fe_3_O_4_ nanoparticles closed in polythiophene aggregates. The latter plays the role of a shape- and morphology-directed template for assembling the above-mentioned bimetallic system in a flower-like arrangement [191]. Reagents and conditions: a = Ph_2_O, 240 °C, 24 h, Y = 70%; b, c = next steps.

The same year, Kaur et al., synthesised polythiophene coated bimetallic Au-Fe_3_O_4_ nano-catalyst starting from DA cycloaddition with CO extrusion (Scheme 136) [191].

Notably, the obtained polythiophene-encapsulated Au-Fe_3_O_4_ aggregates turned out to be efficient and recyclable catalytic systems for obtaining quinoline carboxylates starting from ortho-C-H activation in aniline-substrate, under mild and eco-friendly conditions.

**Scheme 137:**
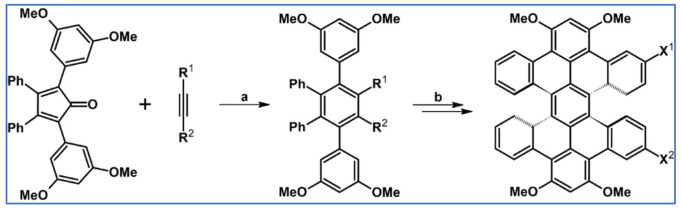
Functionalised hexabenzoperylenes: DA cycloaddition with CO extrusion and partial Scholl reaction as crucial steps in the synthetic path [192]. Reagents and conditions: X, Y = –CH_2_CH_2_CH_2_C(O)OMe, –CH_2_CH_2_CH_2_C(O)OMe; –CH_2_CH_2_CH_2_CH_2_OH, –CH_2_CH_2_CH_2_CH_2_OH; –CH_2_CH_2_CH_2_CH_3_, –CH_2_CH_2_CH_2_CH_2_OSiMe_3_; –(CH_2_)_5_CH_3_, –(CH_2_)_4_O(CO)(CH_2_)_4_-biotine motif; –(CH_2_)_11_CH_3_, –(CH_2_)_11_CH_3_; a = Ph_2_O, 240 °C, Y = 82% (for R^1^ = n-C_6_H_12_, R^2^ = –CH_2_CH_2_CH_2_C(O)OMe), Y = 84% (for R^1^ = –CH_2_CH_2_CH_2_C(O)OMe, R^2^ = –CH_2_CH_2_CH_2_C(O)OMe, Y = 51% (for R^1^ = n-C_4_H_9_, R^1^ = –CH_2_CH_2_CH_2_C(O)OMe).

In 2018, Li and colleagues reported obtaining hexabenzoperylenes possessing special peripheral groups using DA cycloaddition with CO extrusion and Scholl cyclodehydrocondensation (Scheme 137) [192].

Despite the peripheral X and Y groups, the obtained semiconducting HBPs maintain the ability of π-stacking and are attractive as a supramolecular platform for sensing applications. Such HBPs used in devices integrating an OFET channel and a microfluidic channel enable the detection of diverse chemical and biological objects.

**Scheme 138:**
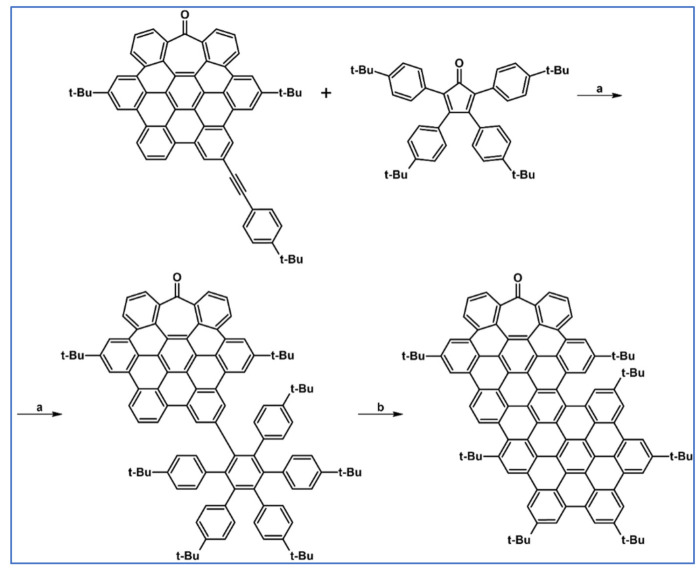
Synthesis of nanographene (NG) via DA cycloaddition and Scholl reaction [193]. Reagents and conditions: a = Ph_2_O, rfx, 8 h; Y = 34%; b = next step (Scholl reaction); Y = 46%.

As described in a paper by Cruz et al., from 2018, DA cycloaddition with CO extrusion was involved in synthesizing a distorted carbonyl group and *t*-butyl end-groups containing ribbon-shaped nanographene (Scheme 138) [193].

Notably, a [5]carbohelicene and aromatic saddle-shaped ketone moieties are responsible for the distortion of the whole structure. It is worth adding that the nanomaterial (size: ca. 2 nm length and 1 nm width) is characterized by extraordinary properties, namely: on two-photon absorption-based up-conversion, circularly polarized luminescence and long emission lifetime (21.5 ns). Indeed, that is an unprecedented yet sought-after combination of properties as far as graphene-related nanomaterials are concerned. [5]Helicene motif and extensive aromatic carbon atoms networks are of particular significance when it comes to hiroptical activity, including nonlinear absorption and pull–push structure, respectively [193].

**Scheme 139:**
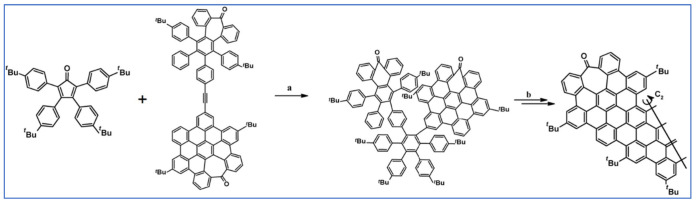
DA cycloaddition with CO extrusion followed by Scholl dehydrocondensation as crucial steps leading to enantiopure superhelicene nanographene [194]. Reagents and conditions: a = Ph_2_O, rfx, 10 h, Y = 75%; b = Scholl reaction.

The synthesis and characterization of an enantiopure superhelicene nanographene in which two saddle-shaped and one planar hexabenzocoronene (HBC) units are arranged in a helicoidal shape to form an undecabenzo[7]carbohelicene was also presented by Cruz et al., (Scheme 139) [194].

It is worth noting that the obtained product was the first completely π-conjugated [7]helicene. Moreover, after resolution using chiral-HPLC, the obtained enantiopure isomers were deeply characterized as far as their chiroptical, photophysical, and especially non-linear properties are concerned.

**Scheme 140:**
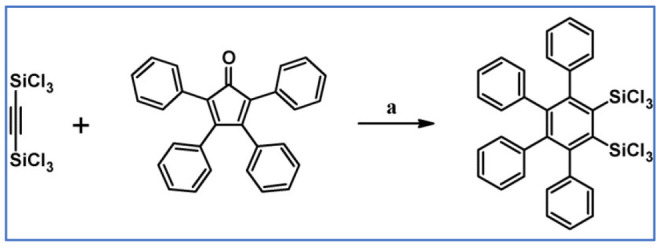
The synthesis of 1,2-bis(trichlorosilyl)-3,4,5,6-tetraphenylbenzene via cycloaddition of appropriate diene and dienophile [195]. Reagents and conditions: a = CH_2_Cl_2_, 180 °C, 4 d, Y = 83%.

Georg et al., have developed convenient protocols for the synthesis of multiply SiCl_3_-substituted organic scaffolds, starting from Cl_2_C=CCl_2_. One of the products was used for the synthesis of 1,2-bis(trichlorosilyl)-3,4,5,6-tetraphenylbenzene (with 83% yield) using DA cycloaddition of 1,2-bis(trichlorosilyl)ethyne and 2,3,4,5-tetraphenyl-2,4-cyclopentadien-1-one with CO extrusion (Scheme 140) [195].

**Scheme 141:**
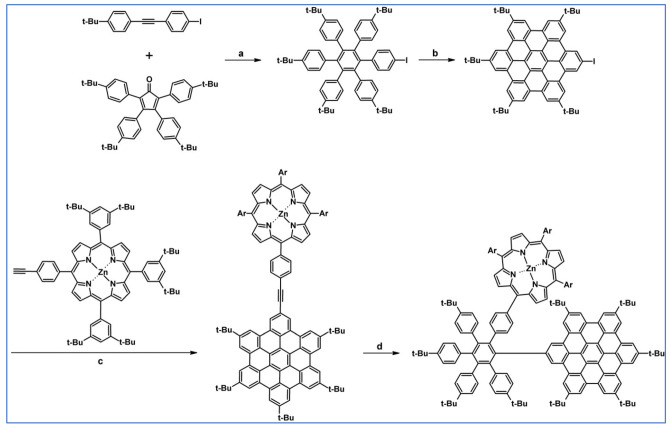
DA cycloaddition with CO extrusion as crucial steps in the synthesis of Zn-porphyrin-HAB-HBC triad [196]. Reagents and conditions: R = 3,5-di-tert-butylphenyl; a = Ph_2_O, 260 °C, MW, 12 h, Y = 78%; b = Scholl reaction; c = Sonogashira coupling; d = Ph_2_O, 260 °C, MW, 12 h, Y = 50%.

Additionally, in 2018, Zinc-porphyrin bearing hexaarylbenzene-hexabenzocoronene (HAB-HBC) motif was obtained involving DA cycloaddition with CO extrusion (two times), Scholl reaction and Sonogashira coupling (Scheme 141) [196].

Theoretical calculations revealed that frontier orbitals and also HOMO-1 and LUMO+1 are located entirely on the porphyrin fragment. In our opinion, the prospect of using this complex as a nanomaterial for organic electronics is not promising.

**Scheme 142:**
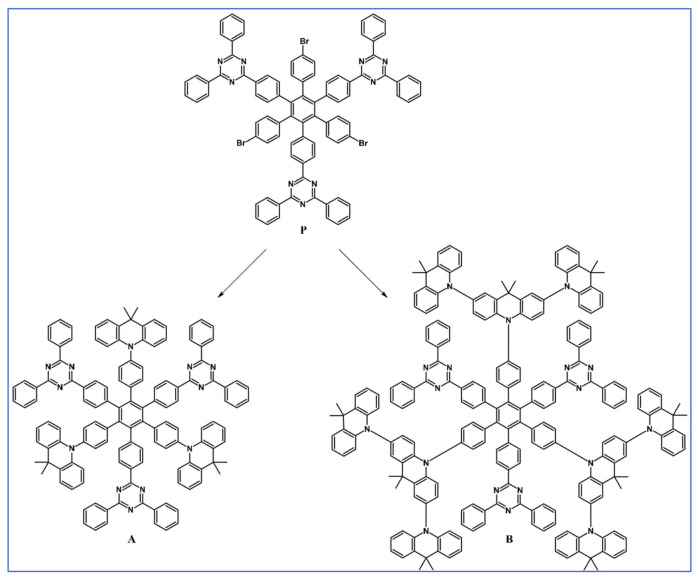
Precursor (**P**) of the target structures (**A**,**B**) [197].

**Scheme 143:**
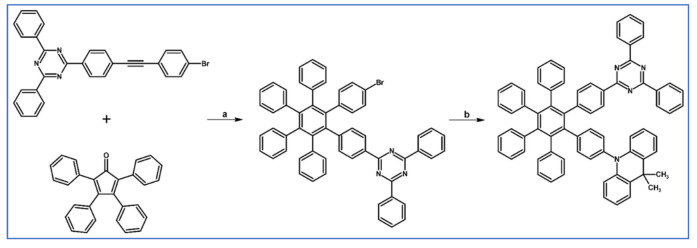
DA cycloaddition with CO extrusion involved in the syntheses one of the control compounds [197]. Reagents and conditions: a = Ph_2_O, 260 °C, 48 h; Y = 50%; b = next step.

In 2019, Wang et al., reported the design and synthesis of hexaarylbenzene derivatives equipped with circularly arranged around HAB-node electron donors (ED) and acceptors (EA). The role of ED and EA play acridan/dendritic triacridan and triazine motifs, respectively. Notably, the discussed nanomaterials are characterized by very effective, through-space charge transfer and TADF and AIE effects. The precursor of both target compounds (Scheme 142) was obtained via Co-catalyzed cyclotrimerization of appropriate acetylene, but one of the control compounds was provided by DA cycloaddition (Scheme 143) [197].

Importantly, when finishing this paper, OLEDs fabricated from described TSCT-HABs belong to the most effective TADF OLEDs (with external quantum efficiency up to 14.2%). Moreover, it was shown that in the case of fully symmetrical derivatives, HPB catalytic cyclotrimerization of appropriately substituted acetylenes could be an alternative to DA cycloaddition with CO extrusion.

**Scheme 144:**
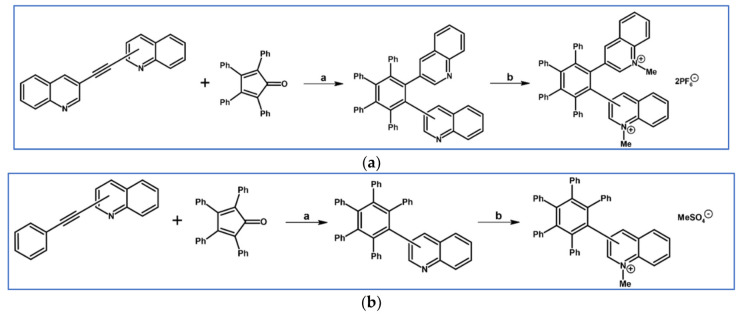
DA cycloaddition in the syntheses of hexaaryl substituted benzene bearing two or three quinoline motifs [198]. Reagents and conditions: (**a**) a = benzophenone, rfx; up to 90% yield; b = next step; (**b**) a = benzophenone, rfx, 0.5 h; up to 81% yield; b = next step.

In 2019, a series of propeller-shaped quinolinium-substituted benzenes and their electrostatically neutral precursors were prepared under variation of the substitution site of the quinoline ring (2-yl, 3-yl, and 4-yl), covering the range from the monocationic quinolinium-2,3,4,5,6-pentaphenylbenzene to the hexacationic hexakis(1-methylquinolinium-3-yl)benzene. Neutral precursors were obtained via cyclotrimerization of appropriately substituted acetylenes (for instance hexa-substituted one) or using DA cycloaddition among adequately substituted acetylenes and tetraphenylacetylene. The syntheses involving DA cycloaddition are shown in Scheme 144 [198].

Notably, the hexacationic hexakis(1-methylquinolinium-3-yl)benzene can exist in eight different rotamers, and due to the propeller-shape geometry which suppresses conjugation throughout the entire π-electron system, the frontier orbitals are located in the individual quinolinium rings.

**Scheme 145:**
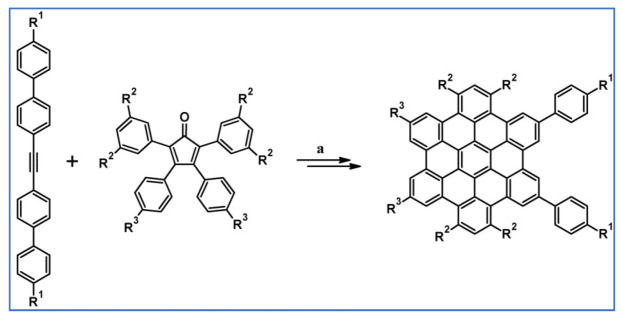
DA cycloaddition of acetylene and cyclopentadienone in the multi-step syntheses of Gemini-shaped hexa-peri-hexabenzocoronenes (HBCs) carrying pyridyl-appended side chains [199]. Reagents and conditions: R^1^ = –(OCH_2_CH_2_)_n_OX. n = 3 or 4, X = Ac or H; R^2^ = (CH_2_CH_2_)_n_OCH_2_Py, n = 3 or 4; R^2^ = F; R^3^ = *n*-C_12_H_25_; a = Ph_2_O, rfx and following reactions.

The DA cycloaddition of acetylene and cyclopentadienone provided four different Gemini-shaped hexa-peri-hexabenzocoronenes (HBCs) carrying pyridyl-appended side chains, which was reported in 2019 by Liu and co-workers (Scheme 145) [199].

It was stated that self-assembling behaviours (in different conditions) of obtained HBCs strongly depend on the substituents at the periphery of the HBC core and the length of hydrophilic chains. For instance, HBCs not containing fluorine atoms self-assembled into nanotubes, whereas HBC with four *F*-substituent (R^2^ = F) self-assembled into nanofibers.

**Scheme 146:**
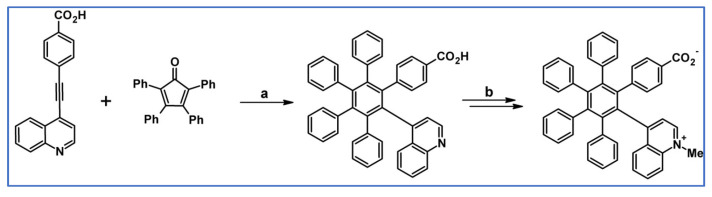
Synthesis of heterocyclic mesomeric betaine starting from DA cycloaddition with CO extrusion [200]. Reagents and conditions: a = Ph_2_O, rfx, 4 h, Y = 80%; b = next steps.

As Batsyts and colleagues reported in 2019, pseudo-cross-conjugated betaine possessing quinolinium moiety was successfully obtained using DA cycloaddition with CO extrusion, tetraphenylcyclopentadienone, and appropriately disubstituted acetylene (Scheme 146) [200].

As far as frontier orbitals of this propeller shaped compound are concerned, HOMO is located on the COOH group but LUMO on the quinolinium moiety. Moreover, propeller shape of considering betaine prevents coupling, the charge is focused on the specific part of this molecule and causes that benzene fragments have no influence on the electronic spectrum.

**Scheme 147:**
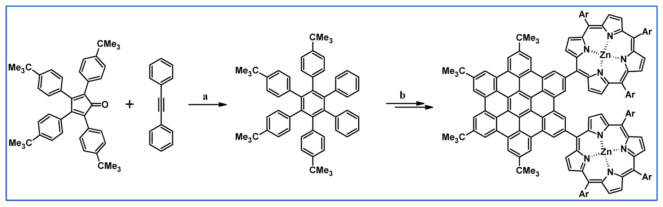
DA cycloaddition between acetylene and cyclopentadienone in the syntheses of ortho-bis-porphyrinatozinc(II) [201]. Reagents and conditions: a = Ph_2_O, 260 °C, MW, 12 h, Y = 75%.

In 2019, Martin et al., described the successful preparation and characterization of a series of ortho-, meta- and para-bis-porphyrin with functionalized hexabenzocoronenes and their Zn-complexes [201]. Ortho- and para-isomers were synthesized starting from DA cycloaddition between appropriate acetylene and cyclopentadienone (the synthesis of ortho-isomer is depicted in Scheme 147) [201].

It was observed that the intensity of the electronic interaction between the two optical electrodes (two Zn-porphyrin moieties) depends on the substitution type (ortho-, meta-, or para-).

**Scheme 148:**
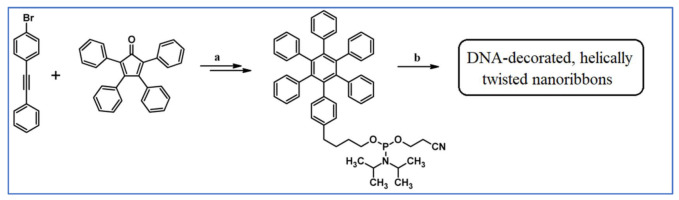
DA cycloaddition in the synthetic procedure of DNA decorating with HPB [202]. Reagents and conditions: a = Ph_2_O, 280 °C, 12 h and next steps.

Golla et al., reported a new DNA-amphiphiles equipped with HPB motifs. Due to the self-assembling properties of the above-mentioned modified DNA-containing molecules, driven by amphiphilicity, helically twisted nanoribbons were created. HPB-motif was introduced into discussed structures via DA cycloaddition involving appropriate acetylene and tetraphenylcyclopentadienone (Scheme 148) [202].

This was the first report demonstrating the design and syntheses of a helically twisted ribbon with one handedness from the self-assembly of a DNA-based amphiphile that can act as a universal template for the fabrication of 1D chiral plasmonic nanomaterials.

**Scheme 149:**
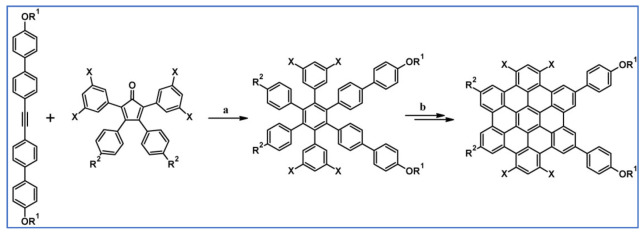
DA cycloaddion of acetylene and cyclopentadienones in the syntheses of hexa-peri-hexabenzocoronenes [203]. Reagents and conditions: R^1^ = hydrophilic chiral chains, for instance (*R*)- or (*S*)-CH_2_CH(Me)CH_2_CH_2_OMe; R^2^ = C_12_H_25_, X = H or F; a = NaH, THF, rfx; b = next steps.

The same year, two groups of chiral gemini-shaped, amphiphilic hexa-peri-hexabenzocoronenes, carrying two hydrophilic chiral chains with different lengths, were synthesized using DA cycloaddition of acetylene and cyclopentadienones as a crucial step (Scheme 149) [203].

Importantly, synthesized nanographenes not containing fluorine atoms (X = H, see Scheme 149), self-assembled into nanotubes under certain conditions and the molecular chirality were successfully transferred to the supramolecular system during such a process. By contrast, nanographenes substituted with fluorine atoms (X = F, see Scheme 149) alone could not assemble into nanotubes.

**Scheme 150:**
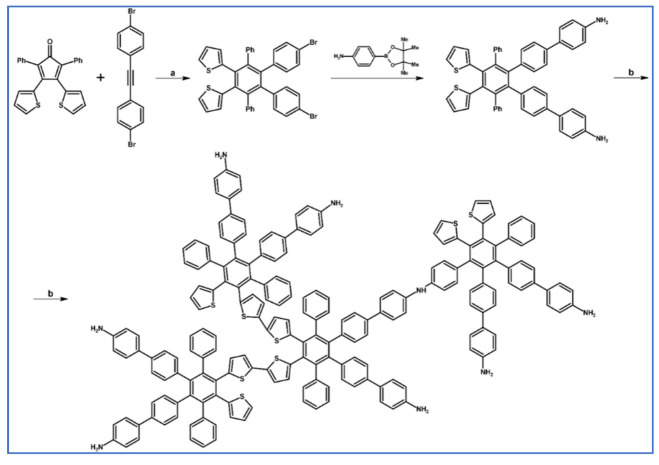
Synthetic procedure starting from DA cycloaddition with CO extrusion leading to Au-Fe_3_O_4_ nanocomposites encapsulated inside AIE-active, supramolecular, porous hetero-oligophenylene nanomaterial [204]. Reagents and conditions: a = Ph_2_O, 240 °C; b = Au^3+^ + Fe^3+^.

Hexaarylbenzene bearing two 2-thienyl and two 4-bromophenyl groups was obtained via DA cycloaddition with CO extrusion (Scheme 150) [204]. This precursor was utilized for developing photo-active, easily separated, and reusable catalyst (thanks to its magnetic properties). The above-mentioned catalytic systems were applied for aniline reactions with propiolate, leading to quinoline or aminoacrylate derivatives in mild and eco-friendly conditions.

It should be emphasized that AIE activity of supporting material (for Au-Fe_3_O_4_) plays a crucial role in the significant increase in the efficiency of the analyzed photocatalytic system.

**Scheme 151:**
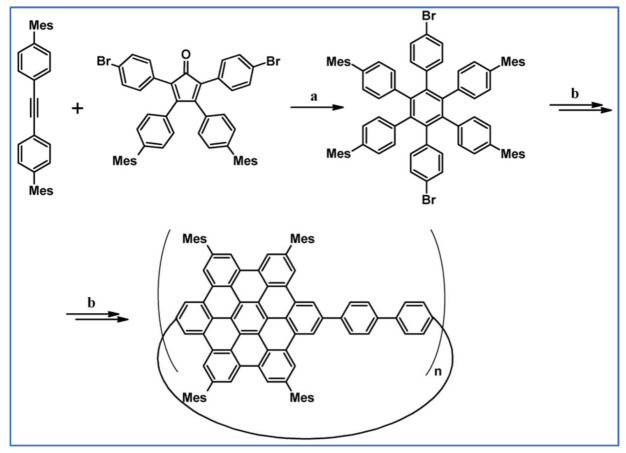
DA cycloaddition in the multistep synthesis of large sized carbon nanohoops [205]. Reagents and conditions: n = 3 or 4; a = Ph_2_O, 200–220 °C, 7 h, Y = 91%; b = next steps.

In 2019, large-sized highly flexible carbon nanohoops were successfully prepared using DA cycloaddition of acetylene to cyclopentadienone, followed by Scholl reaction, borylation and Pt-mediated macrocyclization of the diborylated hexa-peri-hexabenzocoronene (HBC) derivative containing four mesityl substituents at the periphery (Scheme 151) [205].

**Scheme 152:**
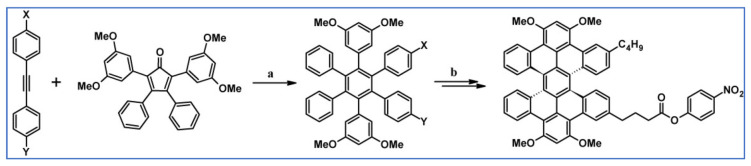
Synthesis of hexabenzoperylene’s functionalized active ester using DA cycloaddition of acetylene and cyclopentadienone [206]. Reagents and conditions: X = Y = COOMe; or X = (CH_2_)_3_Me, Y = (CH_2_)_3_COOMe; a = Ph_2_O, 240 °C; b = next steps.

In 2019, Li et al., it stated that methyl acetate functionalized HBP (hexabenzoperylene), similar to other functionalized HBPs, in single crystals self-assembles into a π-stacked supramolecular nanosheet [206]. The perylene motif was created via DA cycloaddition of appropriate acetylenes and cyclopentadienones, followed by selective Scholl dehydrocondensation (Scheme 152).

Moreover, it was confirmed that active ester-functionalized HBP (b—see Scheme 152) could be used for manufacturing analytical devices for effective differentiation between primary, secondary, and tertiary amines in water solutions.

**Scheme 153:**
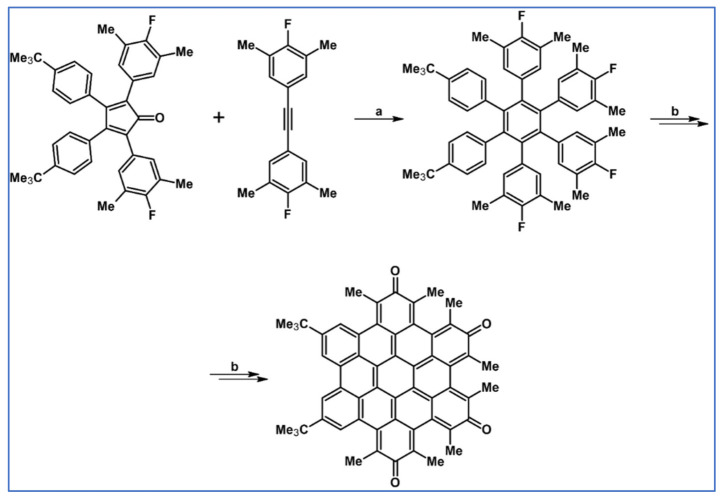
Syntheses of SBQs starting from DA cycloaddition-Scholl reaction tandem—an example [207]. Reagents and conditions: a = Ph_2_O, 250 °C, 24 h, Y = 97%; b = next steps (i.a. Scholl reaction).

A series of superbenzoquinones (SBQs) with up to six C=O groups was obtained by Li and co-workers in 2019. The synthesis involved DA cycloaddition with CO extrusion or Co-catalysed cyclotrimerization and finally step-by-step dearomatization of HBC precursors. An example of such a reaction cycle, leading to the SBQ mentioned above equipped with four carbonyl groups, is shown in Scheme 153 below [207].

Importantly, similar to bisanthenedione, which is our remark, and contrary to classical quinone, such SBQs presented multiradical and open-shell character. Moreover, electrochemical, magnetic, and optical properties of considered SBQs are strictly related to the structure and symmetry of such nanomolecules.

**Scheme 154:**
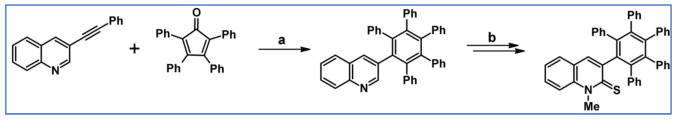
DA cycloaddition with CO extrusion: synthesis of 3-(Ph_5_Ph)-quinoline [208]. Reagents and conditions: a = Ph_2_CO, rfx, 0.5 h, Y = 51%; b = next steps.

Batsyts and colleagues reported that DA cycloaddition between pentaphenylcyclopentadienone and 3-(phenylethynyl)quinoline was involved in the synthetic route, leading to 3-(Ph_5_Ph)-quinoline (Scheme 154) [208].

The obtained compound and other 3-arylquinolines (prepared via arylation of 3-bromoquinoline) were transformed into *N*-methylquinolinium salts in the next step. The obtained salts were used to generate *N*-heterocyclic carbene, which finally underwent trapping by sulphur or selenium.

**Scheme 155:**
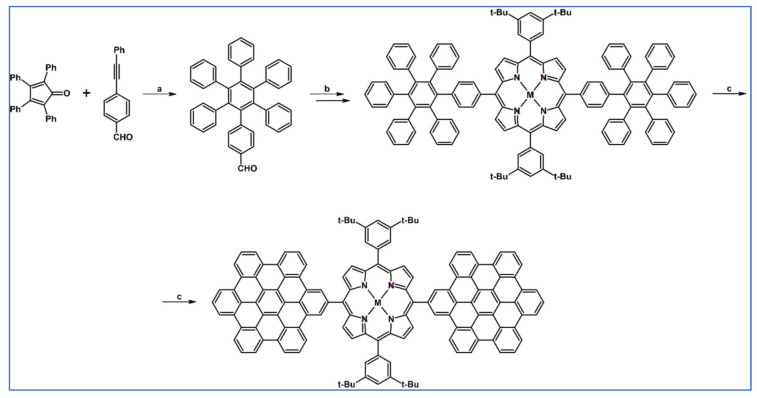
DA cycloaddition with CO extrusion and Scholl reaction engaged in synthetic route leading to the porphyrins equipped with the Ph_5_Ph or HBC-motifs [209]. Reagents and conditions: a = Ph_2_O, 260 °C; b = next steps; and c = Scholl reaction.

Nisa et al., employed DA cycloaddition with CO extrusion and cyclodehydrocondensation in synthesizing porphyrin bearing two Ph_5_Ph or two HBC motifs (Scheme 155) [209]. Moreover, Zn-complexes of the ligands mentioned above were synthesized.

DA cycloaddition was realized typically, but Scholl oxidative dehydrogenation was conducted using eco-friendly NOBF_4_ instead of typically used FeCl_3_. Importantly, in porphyrins possessing peripheral HBC moieties, intense and long-distant electronic communication was confirmed. Additionally, a small HOMO–LUMO gap suggests that such porphyrins are attractive for light-harvesting and charge transport in optoelectronic devices.

**Scheme 156:**
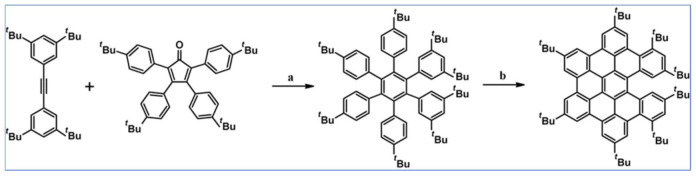
Synthesis of HBC-based [5]helicene via Diels–Alder cycloaddition with CO extrusion and finally Scholl reaction [210]. Reagents and conditions: a = Ph_2_O, 260 °C, MW, 12 h, Y = 81%; b = next step (Scholl reaction).

A synthetic route involving DA cycloaddition step and leading to hexabenzocoronene-based helical nanographene motif was developed by Martin and colleagues in 2020 (Scheme 156) [210].

**Scheme 157:**
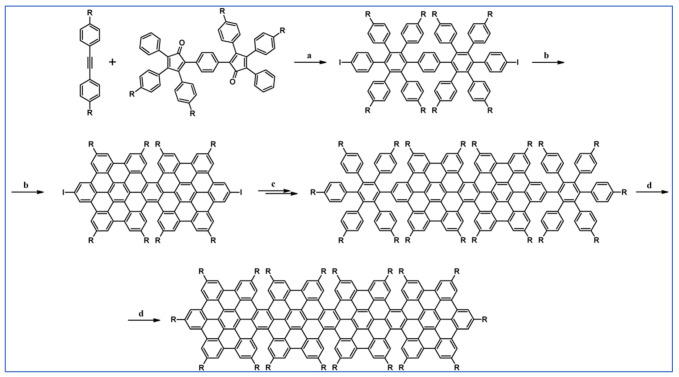
DA of acetylenes and cyclopentadienones for the synthesis of supertwistacene [211]. Reagents and conditions: R = *t*-Bu; a = Ph_2_O, 260 °C, 48 h, Y = 76%; b = Scholl reaction; c = 2,3,4,5-tetrakis(4-(tert-butyl)phenyl)cyclopenta-2,4-dien-1-on; Ph_2_O, 260 °C, 48 h, Y = 36%; d = Scholl reaction.

In 2020, Ma et al., reported the syntheses and theoretical, (chir)optical, and electronic characterization of a helical graphene nanoribbon, named supertwistacene, which has four superbenzene (HBC) units linearly fused in a helical manner [211]. DA cycloaddition of acetylenes and cyclopentadienes were two key steps in this multi-step procedure (Scheme 157).

It is noteworthy that the obtained supertwistacene exhibits significantly higher confirational stability than other known twistacenes. Moreover, racemic product resolution via chiral HPLC led to enantiopured isomer. The structure of final compound was confirmed using single-crystal X-ray diffraction.

**Scheme 158:**
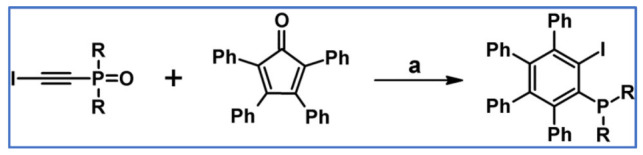
Syntheses of 1-iodo-2-PR_2_-3,4,5,6-tetraphenylbenzenes via cycloaddition involving new iodo-PR_2_- substituted acetylene dienophiles [212]. Reagents and conditions: a = 125 °C, 4–10 d, Y = 75% (for R = OMe) and Y = 83% (for R = Ph).

In 2020, Marian and Maas reported that new disubstituted acetylene dienophiles bearing iodo- and PR_2_ substituents were synthesized and used for cycloadditions, including DA cycloaddition with CO extrusion (Scheme 158) [212].

**Scheme 159:**
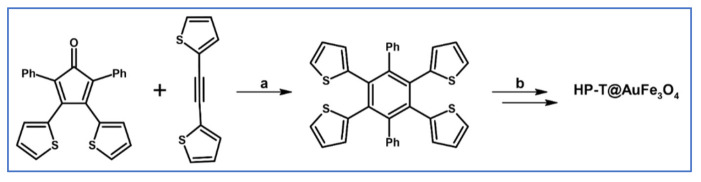
Syntheses of supramolecular catalytic system (HP-T@Au-Fe_3_O_4_) starting from DA cycloaddition with CO extrusion [213]. Reagents and conditions: a = Ph_2_O, 240 °C, 24 h, Y = 75%; b = next steps.

The same year Kaur, Kumar, and Bhalla described reusable, supramolecular, visible light activated and magnetic catalytic systems HP-T@Au-Fe_3_O_4_ that was easily fabricated starting from DA cycloaddition with CO extrusion (Scheme 159) [213].

The above-mentioned catalytic systems were highly active in the Kumada cross-coupling and Heck reaction using mild, aerial conditions and visible light irradiation. Moreover, thanks to the magnetic properties of Fe_3_O_4_, they were easily and effectively recoverable.

**Scheme 160:**
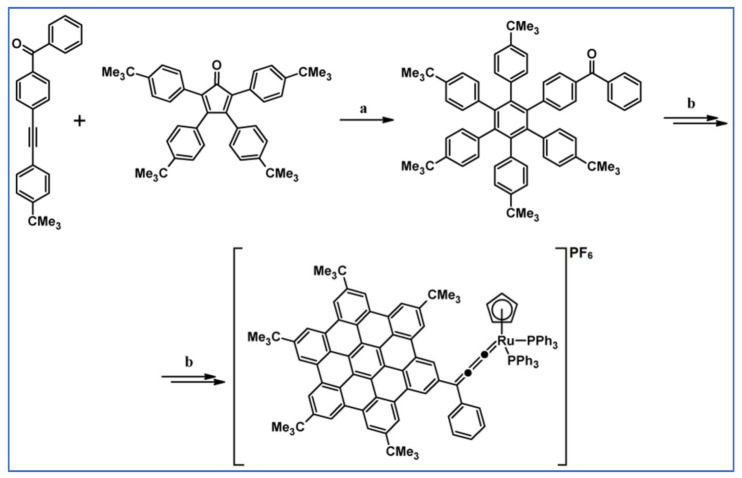
Introducing of HBC-motif into Ru-allenylidene complex with the aid of DA cycloaddition of appropriate acetylene and cyclopentadienone [214]. Reagents and conditions: a = PhMe, 220 °C, 60 h, Y = 71%.

Carbon-rich ruthenium allenylidene complexes containing a hexaarylbenzene (HAB) or a hexaperihexabenzocoronene (HBC) substituent were synthesized and published in 2020 (Scheme 160) [214]. The HBC-motif was created via Diels–Alder cycloaddition–Scholl dehydrocondensation sequence.

The complexes exhibit interesting π–π-stacking interaction in the solid state as well as aggregation in solution. The authors claim that the HBC-motif-containing complex showed in Scheme 160 is an allenylidene complex with one of the most extended conjugated systems.

#### 2.1.2. Cycloaddition of Cyclopentadienones and Diynes

**Scheme 161:**
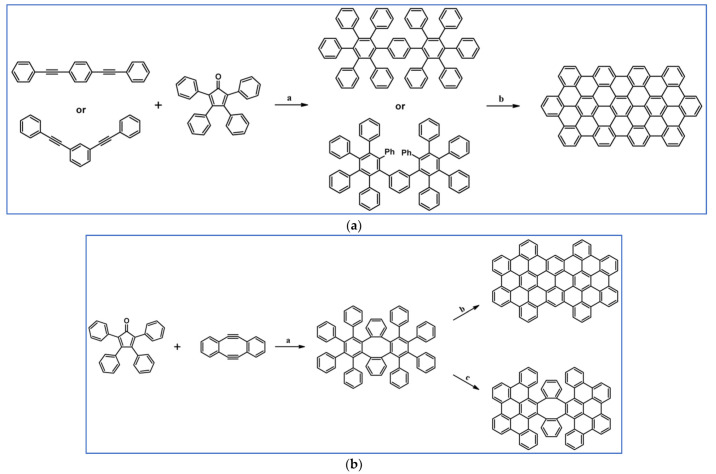
DA cycloaddition in the synthesis of polyphenylenes as nanographenes precursors [215]. Reagents and conditions: (**a**) a = Ph_2_O, 220 °C, 48 h, up to 97% yield; b = next step (Scholl dehydrocondensation); (**b**) a = Ph_2_O, 150 °C, 12 h, Y = 95%; b, c = next steps (Scholl dehydrocondensation-skeletal rearrangement).

Different variants of nanographenes obtained in 1997 by Müller and colleagues via DA cycloaddition–Scholl cyclodehydrocondensation and skeletal rearrangement sequence are presented in Scheme 161 [215].

Interestingly, intramolecular cyclodehydrogenation is accompanied by surprisingly smooth skeletal rearrangements, leading to planar PAHs from “nonplanarizable” oligophenylenes by removing steric strain.

**Scheme 162:**
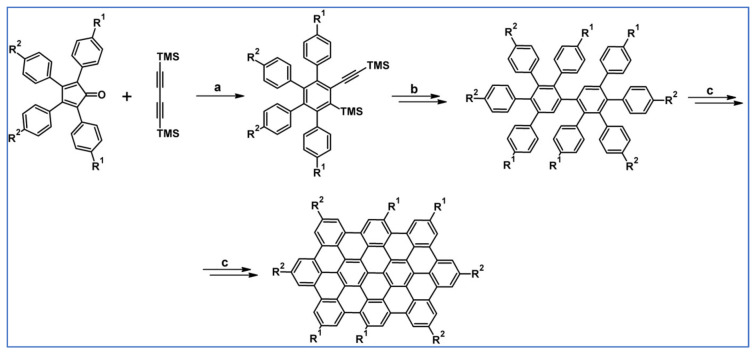
Syntheses of soluble C_60_ graphite segments via DA cycloaddition-Scholl reaction [216]. Reagents and conditions: (a) R^1^ = R^2^ = H; (b) R^1^ = H, R^2^ = *n*-C_12_H_25_; (c) R^1^ = R^2^ = *n*-C_12_H_25_; a = Ph_2_O, 250 °C, 3–6 h, yield up to 100%; b, c = next steps (c = Scholl reaction).

As work from Iyer’s research team suggested in 1998, DA cycloaddition with CO extrusion followed by cyclodehydrocondensation were decisive steps in the synthetic procedure leading to C_60_H_22_ PAH (Scheme 162) [216]. Notably, the synthesis of C_60_H_22_ graphite segment in gram or even decagram quantities is surprisingly straightforward. More importantly, soluble tetra- and octa-*n*-alkyl derivatives of C_60_H_22_ can be prepared. This way, the formation of monolayers from the solution is possible.

**Scheme 163:**
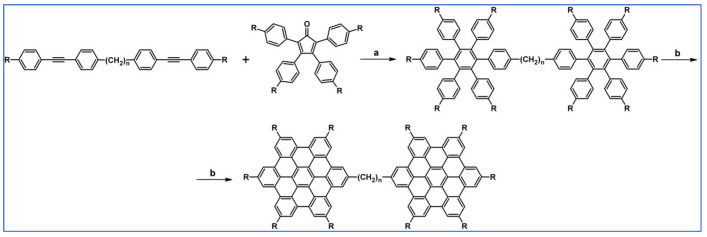
Syntheses of alkyl-substituted bis-hexa-peri-hexabenzocoronenyls connected directly or via –(CH_2_)_12_– bridge using DA cycloaddition-Scholl reaction sequences [217]. Reagents and conditions: (a) n = 0, R = *t*-Bu; (b) n = 12, R = *n*-C_12_H_25_; a = Ph_2_O, rfx.; b = next step (Scholl reaction).

As Ito and co-workers reported in 2000, DA cycloaddition and Scholl dehydrocyclocondensation were crucial steps in the synthetic procedure leading to alkyl-substituted bis-hexa-peri-hexabenzocoronenyls connected directly or via –(CH_2_)_12_– bridge (Scheme 163) [217].

Both obtained bis-HBCs are characterized by good solubility, and both form liquid crystalline phases, while only in the case of reaction **b** product a highly ordered columnar structure with a hexagonal superstructure was observed.

**Scheme 164:**
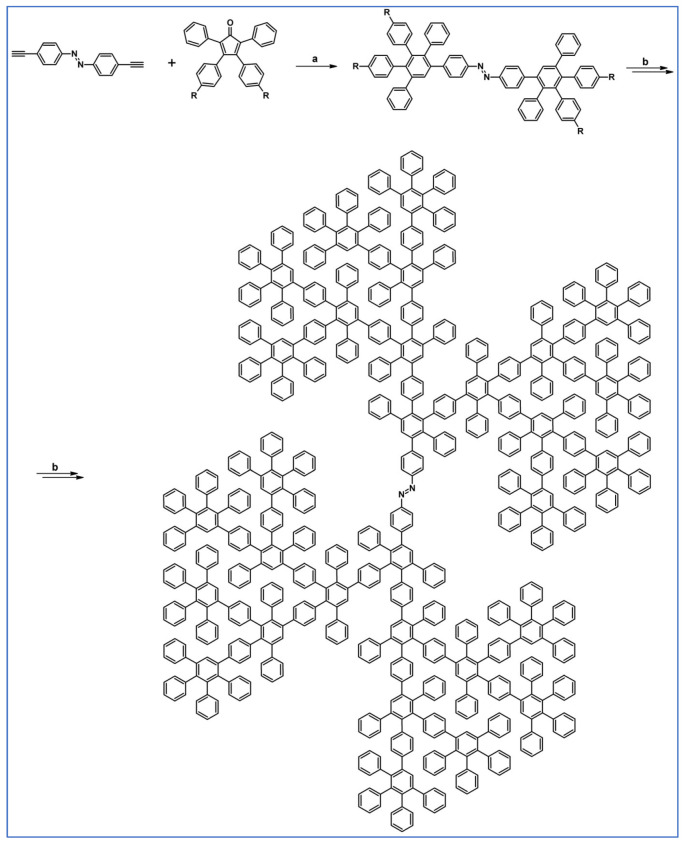
DA cycloaddition with CO extrusion as a crucial tool for the preparation of polyphenylene denrimers posessing azobenzene core [218]. Reagents and conditions: a = *o*-xylene, 142 °C, R= C≡C–Si(*i*-Pr)_3_, H; generation G1: Y = 92% (for R = H), Y = 89% (for R = C≡C–Si(*i*-Pr)_3_); generation G2: Y = 91% (for R = H), Y = 86% (for R = C≡C–Si(*i*-Pr)_3_); generation G3: Y = 93% (for R = H); generation G4: Y = 74% (for R = H); b = next steps.

In 2003 the synthesis, structure, and photochemistry of polyphenylene dendrimers (from the first to fourth generation) with an azobenzene core were described [218]. The synthesis leading up to the fourth-generation dendrimer, starting from DA cycloaddition, is presented in the scheme below (Scheme 164) [218].

These azobenzene-cored polyphenylene dendrimers exhibit effective, reversible, and reproducible photoresponsive properties upon UV and visible irradiation. The photoresponsive behaviour of above-mentioned dendrimers strictly depends on their well-defined and rigid structure. Due to this fact, the greatest photomodulation of dendrimer molecular size has been observed by changing a single central unit.

**Scheme 165:**
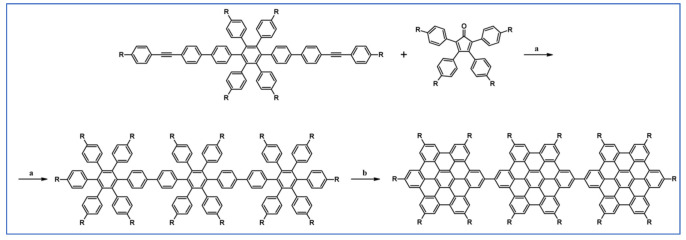
DA cycloaddition and Scholl reaction for the synthesis of super-oligophenylene linear trimer [219]. Reagents and conditions: R = 3,7-dimethyloctanyl; a = Ph_2_O, rfx, 19 h, Y = 78%; b = next step (Scholl reaction).

**Scheme 166:**
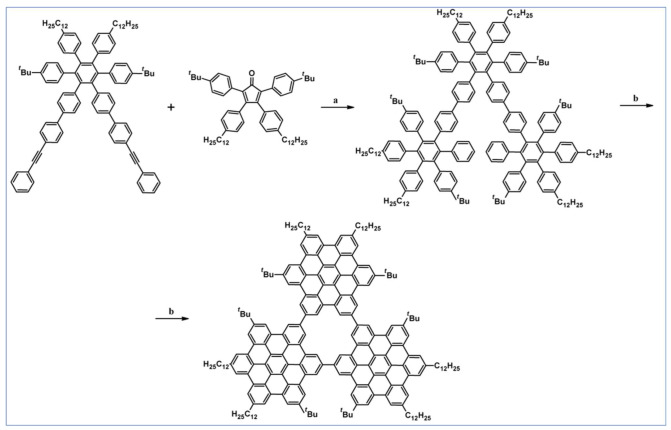
DA cycloaddition and Scholl reaction for the synthesis of super-oligophenylene triangular trimer [219]. Reagents and conditions: a = Ph_2_O, rfx, 4 h, Y = 72%; b = next step (Scholl reaction).

**Scheme 167:**
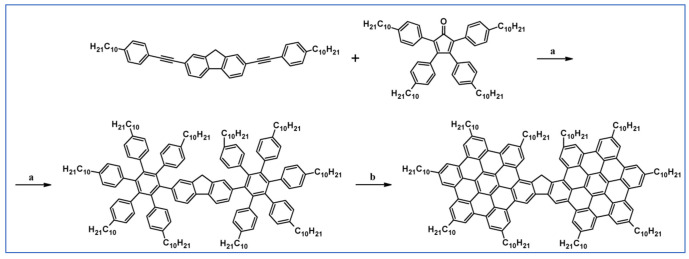
DA cycloaddition and Scholl reaction for the synthesis of Super-oligophenylene dimer with fluorene linker (“Superfluorene”) [219]. Reagents and conditions: a = Ph_2_O, rfx, 19 h, Y = 27%; b = next step (Scholl reaction).

The next year DA cycloaddition with CO extrusion and Scholl reaction were engaged in the multi-step synthesis of oligomers (up to trimer) of “superbenzene” (HBC) with different geometries, linear trimer, triangular trimer, and “superfluorene” are shown in Scheme 165, Scheme 166 and Scheme 167 [219].

These oligomers represent a kind of electronically decoupled oligoarylenes and serve as model compounds of corresponding poly-*p*-(*o*-) “superphenylenes”. Reducing these oligomers by an active metal such as potassium offers the possibility of high-spin oligoradical anion formation. The strong aggregation tendency of HBC leads to a columnar stacking of the HBC dimer, trimer, and “superfluorene” in the bulk state, allowing us to study the charge carrier transport along the HBC multi-columns.

**Scheme 168:**
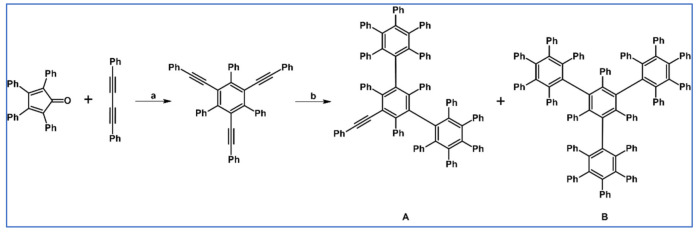
Co-catalysed cyclotrimerization and thermal DA cycloaddition with CO extrusion involved in the syntheses of “cubic graphite” subunit [220]. Reagents and conditions: a = [Co_2_(CO)_8_], dioxane, 8 h, rfx; b = tetraphenylcyclopentadienone, Ph_2_O, 265 °C, 72 h, Y = 34% (for **A**) and 2% (for **B**).

Additionally, in 2006, Wasserfallen and colleagues described Co-catalysed cyclotrimerization, followed by thermal DA cycloaddition with CO extrusion. This way, it was possible to synthesize oligophenylene with an extremely dense packing of the benzene rings from diphenylbutadiyne and tetraphenylcyclopentadienone (Scheme 168) [220].

Unfortunately, time reaction prolongation and increasing the reaction temperature did not reach a higher product yield (**B**). Crystallographic data reveals that the molecule (**B**) retained its theoretical threefold symmetry with only minor deviations. Due to its structure and dense packing of interlocked benzene rings, this oligophenylene could be furthermore used as a suitable precursor for constructing a subunit of ‘cubic graphite’.

**Scheme 169:**
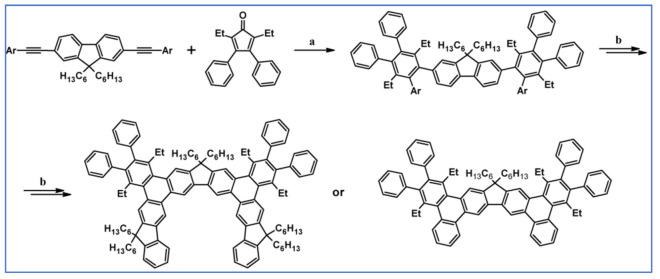
DA cycloaddition with CO extrusion as a crucial step in synthesizing polycyclic aromatic hydrocarbons based on the 10*H*-indeno[1,2-*b*]-triphenylene skeleton [221]. Reagents and conditions: a = mesitylene, rfx, 24 h, Y = 76% (for Ar = 2-(9,9-dihexylfluorenyl)) and Y = 68% (for Ar = Ph); b = next steps (decarbonylation and oxidative cyclization/Scholl reaction).

A family of polycyclic aromatic hydrocarbons of various shapes based on the 10*H*-indeno[1,2-*b*]-triphenylene skeleton has been synthesized by Zhou et al., via a reaction sequence including Diels–Alder cycloaddition, decarbonylation, and oxidative cyclization/Scholl reaction [221]. The synthesis and two structures of the obtained compounds are shown in Scheme 169 [221].

The investigation of photophysical properties demonstrates that the derivatives presented in the scheme above might be suitable for organic field effect transistor (OFET) applications.

**Scheme 170:**
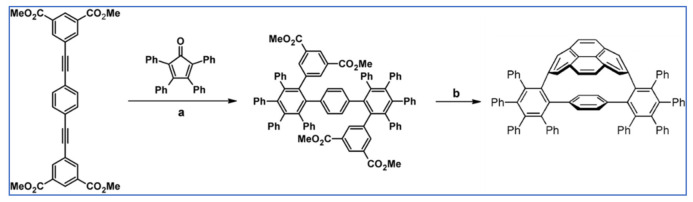
DA cycloaddition with CO extrusion involved in the synthetic route of severely bent pyrene derivative [222]. Reagents and conditions: a = Ph_2_O, rfx, Y =65%; b = next step.

Zhang and colleagues reported synthesis of the pyrenophane octaphenyl derivative using DA cycloaddition with CO extrusion, involving appropriate diene and dienophile (Scheme 170) [222].

DFT calculations revealed that, despite the distorted pyrene motif, the aromaticity of the system remains at a level up to 98% in comparison to planar unsubstituted pyrene.

**Scheme 171:**
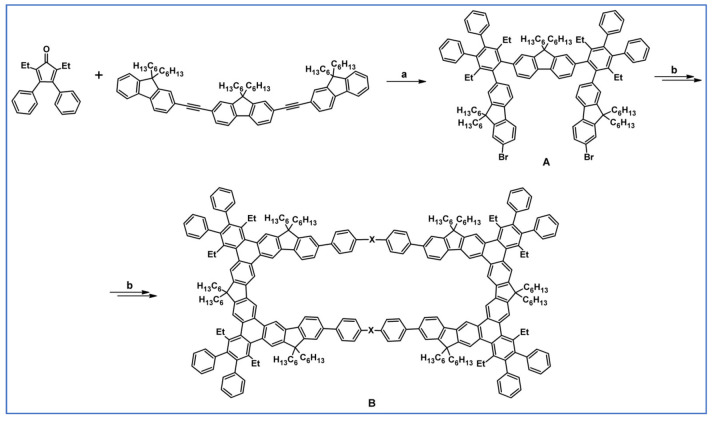
DA cycloaddition with CO extrusion as a crucial step in the synthetic route leading to shape-persistent elliptic macrocycles [223]. Reagents and conditions: X = (E)–CH=CH, C≡C; a = 1,2,4-trimethylbenzene, rfx, 24 h, Y = 88%; b = next steps.

Bimolecular coupling/unimolecular cyclization strategies, including DA cycloaddition, McMurry-, and Glaser-type homocoupling reactions, were utilized in 2008 by Liu et al., to synthesize two shape-persistent elliptic macrocycles, which consist of polycyclic aromatic hydrocarbon units (Scheme 171) [223].

The fluorescence quantum yields (ΦF) of compounds A and B in dilute THF solution were 0.94 and 0.82, respectively, standardized to 9,10-diphenylanthracene.

**Scheme 172:**
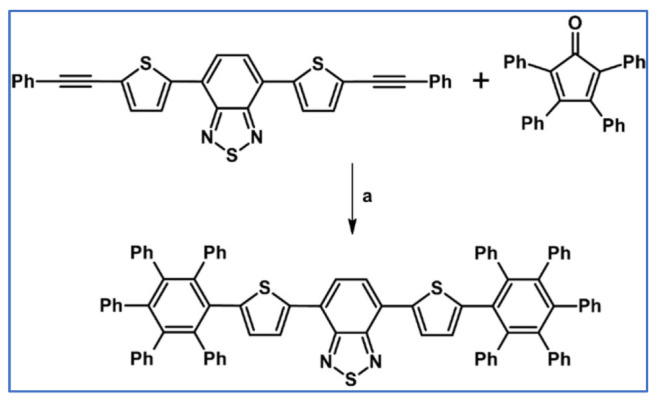
DA cycloaddition with CO extrusion in the synthesis of nonplanar Ph_5_Ph–L–PhPh_5_ derivative [224]. Reagents and conditions: a = Ph_2_O, rfx, 12 h.

DA cycloaddition was applied by Sun and colleagues in the synthetic route leading to a molecule consisting of two pentaphenylbenzene motifs connected through thiazole-benzo[2,1,3]thiadiazole-thiazole bridge (Scheme 172) [224].

The obtained compound is thermally stable, soluble in organic solvents, has excellent film-forming, and exhibits a low tendency to aggregation and stable photoluminescence. This material is dedicated to OLEDs as a red-light molecular emitter.

**Scheme 173:**
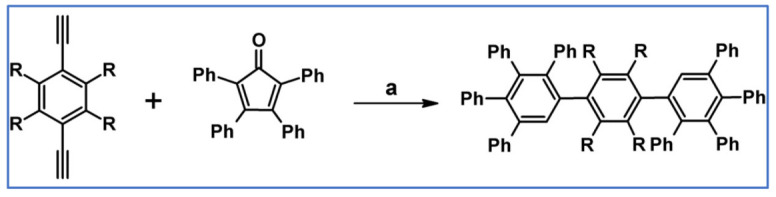
Syntheses of terphenyl derivatives via DA cycloaddition with CO extrusion [225]. Reagents and conditions: a = Ph_2_O, 150 °C, 24 h, Y = 78% (for R = H) or 84% (for R = F).

Two terphenyl derivatives were obtained via DA cycloaddition involving 1,4-diethynylbenze or 1,4-diethynyl-23,5,6-tetrafluorobenzene and tetraphenylcyclopentadienone (Scheme 173) [225].

Notably, replacing four H atoms with four F atoms completely changes the conformation and spatial structure of molecules as there are new, strong intermolecular C-H⋯F interactions.

**Scheme 174:**
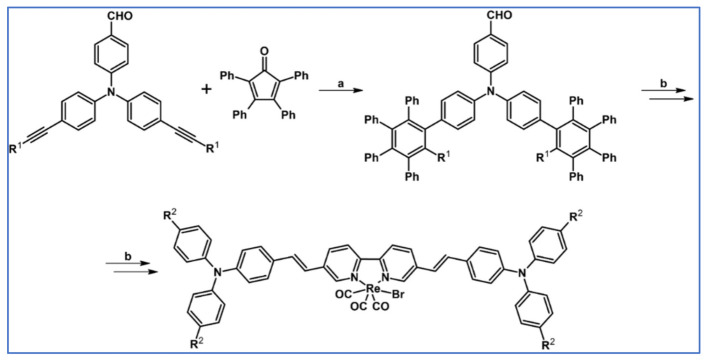
Obtaining of Re(I) complexes with functionalised bpy ligand involving DA cycloaddition with CO extrusion [226]. Reagents and conditions: a = xylene, rfx, 12 h, Y = 46 % (for R^1^ = H) or a = Ph_2_O, rfx, 12 h, Y = 52% (for R^1^ = Ph); b = multistep reaction, R^2^ = Ph_4_Ph or Ph_5_Ph.

Bipyridine ligands equipped with Ph_4_Ph or Ph_5_Ph motifs for rhenium(I) complexes were obtained by Yu and colleagues using DA cycloaddition with CO extrusion. For this measure, they applied tetraphenylcyclopentadienone and appropriately substituted triarylamine (Scheme 174) [226]. Moreover, for comparative purposes, complexes with R^1^ = H, carbazol-1-yl, phenyethynyl were obtained without applying DA cycloaddition.

The much greater reactivity of dienophile possessing mono-substituted triple bond is noticeable, namely the required reaction temperature for R = Ph is over 100 °C higher than for R = H. Photophysical investigations of the obtained complexes revealed that the presence of donor and bulky Ph_4_Ph or Ph_5_Ph motifs in the NAr_2_ chromophores results in red shifting of its emission.

**Scheme 175:**
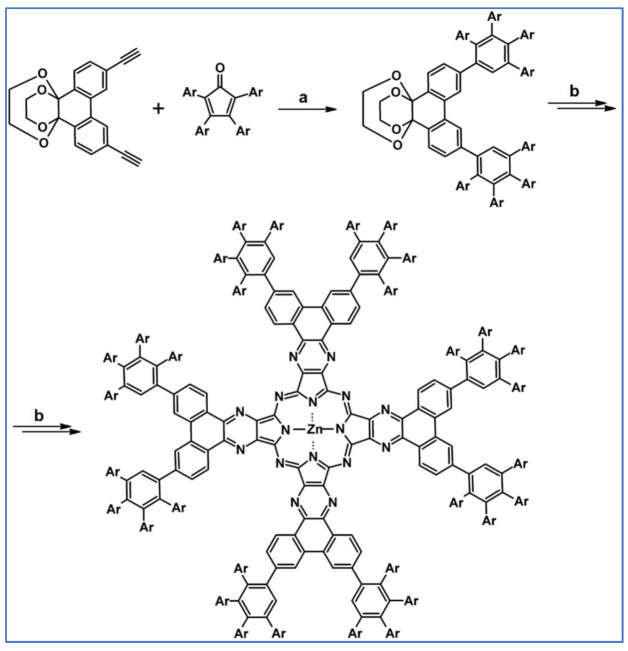
Synthesis of fluorinated Zn–Pc complex starting from DA cycloaddition [227]. Reagents and conditions: Ar = C_6_F_5_; a = MW, *o*-dichlorobenzene, 210 °C, 5 h, Y = 91%.

DA cycloaddition with CO extrusion was a key step in the synthetic way leading to the phthalocyanine ligand and, finally, Zn-complexes, bearing two perfluorinated periphery (tetraphenyl))phenyl motifs (Scheme 175), as stated by Löser, Winzenburg, and Faust in a paper from 2013 [227].

Moreover, due to the increased solubility of the dendritically firmly fixed chromophores in apolar organic solvents, easy handling of the obtained materials in different applications is possible.

**Scheme 176:**
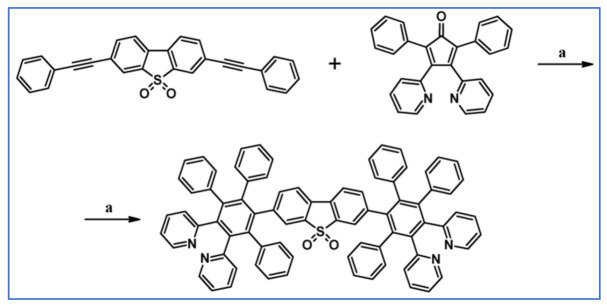
Synthesis of Hexarylbenzene derivative: the Diels–Alder reaction of 3,7-bis(phenylethynyl)dibenzo[*b*,*d*]thiophene-5,5-dioxide with 2,5-diphenyl-3,4-di(pyridin-2-yl)cyclopenta-2,4-dienone [228]. Reagents and conditions: a = Ph_2_O, rfx, 24 h, Y = 74%.

Again in 2015, Kaur et al., presented DA cycloaddition with CO extrusion involved in the synthesis of AIEE active hexarylbenzene derivative, which forms fluorescent aggregates in aqueous media (Scheme 176) [228].

Intriguingly, the obtained aggregates are applied as reactors and stabilizers to prepare in situ generated copper nanoparticles (CuNPs). The CuNPs served as effective catalysts for the alkyl–azide ‘click’ reactions to synthesize 1,2,3-triazoles could be recycled and reused five times without substantial loss of their catalytic activity.

**Scheme 177:**
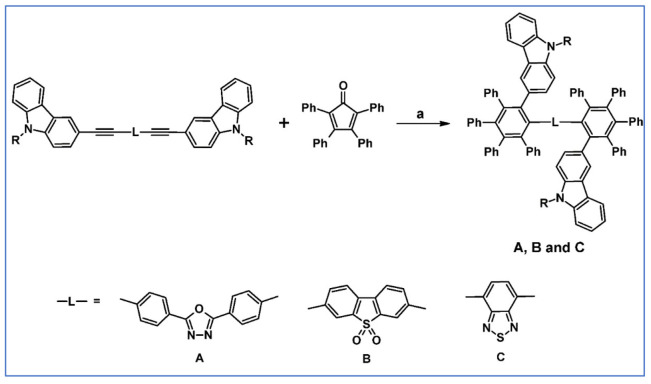
Syntheses of D–A–D molecules via DA cycloaddition of appropriate acetylenic dienophiles and tetraphenylcyclopentadienone [229]. Reagents and conditions: R = *n*-hexyl; a = Ph_2_O, rfx, 48 h, Y = 25% (for **A**) or 37% (for **B**) or 39% (for **C**).

Another example of DA cycloaddition followed by CO extrusion was also an essential part of the syntheses of D–A–D compounds described by Singh et al., in 2015 (Scheme 177) [229]. In the synthesized compounds, the donor was carbazole motif, and the acceptors were 2,5-diaryl-1,3,4-oxadiazole, dibenzothiophene-*S*,*S*-dioxide, or benzo[1,2,5]thiadiazole moieties.

Embedding polyphenylene dendrons into the structure blocks the face-to-face π–π stacking in aggregates. Furthermore, the high solid-state luminescence without quenching of fluorescence and no red shift are observed in the discussed molecules. Additionally, the size of the steric repulsion is decisive when it comes to donor–acceptor interaction, aggregates forming via self-assembling and, finally, emission intensity. Namely, if the steric repulsion prevents the complete molecule planarization, an emission in aggregates is observed.

**Scheme 178:**
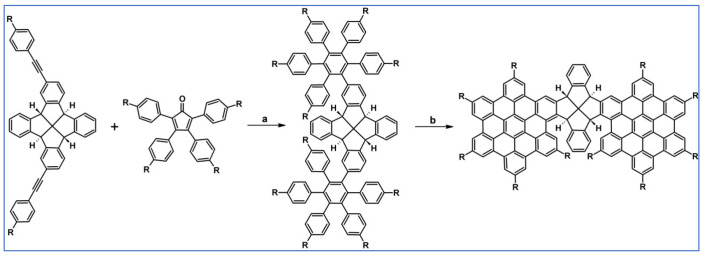
DA cycloaddition with CO extrusion and Scholl reaction involved in the synthetic path leading to HBC fenestrindine bearing two HBC moieties [230]. Reagents and conditions: R = *t*-Bu; a = Ph_2_O, 250 °C, 12 h, Y = 21%; b = next step (Scholl reaction).

An and colleagues described DA cycloaddition with CO extrusion engaging di(*p*-tert-butylphenylethynyl)fenestrindine and tetrakis(*p*-tert-butylphenyl)- cyclopentadienone as substrate. This reaction, followed by multiple Scholl dehydrocondensation, took part in the fusion of two HBC moieties with an all-cis-[5.5.5.5]fenestrane core (Scheme 178) [230].

**Scheme 179:**
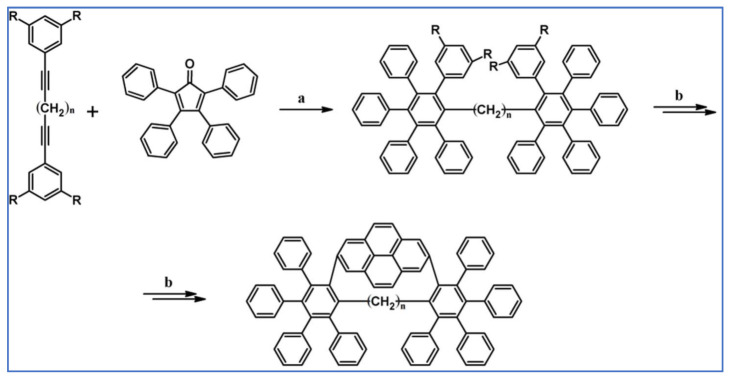
DA cycloaddition of diynes and tetraphenylcyclopentadienone for the synthesis of bent pyrenophanes [231]. Reagents and conditions: R = n = 2–6; a = Ph_2_O, 260 °C, 1–6 d, up to 40% yield; b = next steps.

A series of twisted pyrenophanes, including highly bent ones, were prepared by Zhang and co-workers starting from DA cycloaddition of appropriate substituted diynes and tetraphenylcyclopentadienone (Scheme 179) [231].

**Scheme 180:**
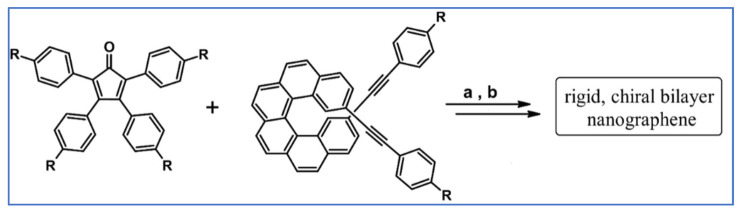
DA cycloaddition of appropriately substituted diyne (rac or chiral) and cyclopentadienone in the synthesis of racemic and enantiopure helical bilayer nanographene [232]. Reagents and conditions: R = *t*-Bu; a = MW, 280 °C, 0.25–0.5 h, 48 and 30% yield (rac and chiral); b = Scholl dehydrocondensation (82%—rac and 92% chiral, 93% ee).

According to Evans’ research group, DA cycloaddition between appropriately substituted diethynylpentacene derivatives (rac or chiral) and tetraarylcyclopentadienones plays a crucial role in the synthetic route leading to a rigid, inherently chiral bilayer nanographene (Scheme 180) [232]. Crucially, both the racemate and enantioenriched isomers (with 93% yield ee) were obtained.

This folded nanographene bilayer system, containing 30 fused benzene rings, comprises two hexa-peri-hexabenzocoronene layers fused to [10]helicene. It should be emphasized that thanks to helicene linker’s rigidity, the obtained system possesses rarely observed conformation, is chiral and soluble in organic solvents. We share the authors’ opinion that the obtained nanographene can be attractive for many applications in modern technologies due to the properties mentioned above.

**Scheme 181:**
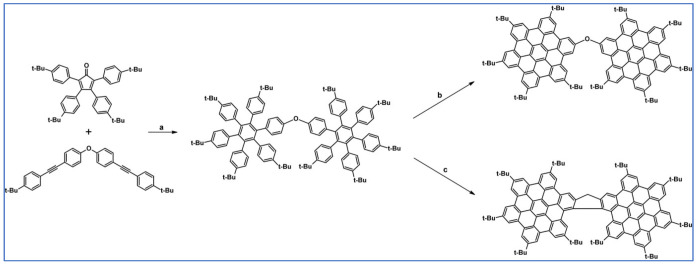
Syntheses of Ph_5_Ph–*O*–PhPh_5_, HBC–*O*–HBC and oxa[7]superhelicene [233]. Reagents and conditions: a = PhMe, 220 °C, 23 h, Y = 86%; b, c = next steps (i.a. Scholl reaction).

The same year Roger et al., described DA cycloaddition with CO extrusion that initiated the synthetic path leading to HBC-*O*-HBC and especially to π-expanded helical chromophore, i.e., oxa[7]superhelicene (Scheme 181) [233].

It is worth emphasizing that the fluorescence quantum yield of the obtained nonplanar, oxa[7]superhelicene is exceptional, namely higher than 80%.

**Scheme 182:**
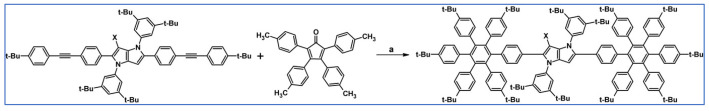
Syntheses of HAB-L-HAB type molecules via DA cycloaddition with CO extrusion [234]. Reagents and conditions: a = Ph_2_O, 250 °C, 42–46 h, Y = 80% (for X = H) or 39% (for X = Cl).

Stężycki and co-workers applied DA with CO extrusion in the synthesis of two HPB-L-HBC type molecules equipped with ten *t*-Bu groups in Ph ring and four *t*-Bu groups in bridging L-fragments (Scheme 182) [234].

The prepared hybrid dyes are characterized with violet (X = Cl) or strongly blue (X = H) fluorescence. However, the electronic communication between HPB fragments and pyrrolo[3,2-*b*]pyrrole core is strongly limited. Moreover, HOMO is located on the pyrrolo-pyrrole core, and, probably, for this reason, attempts to convert HPB moieties into HBC ones failed.

**Scheme 183:**
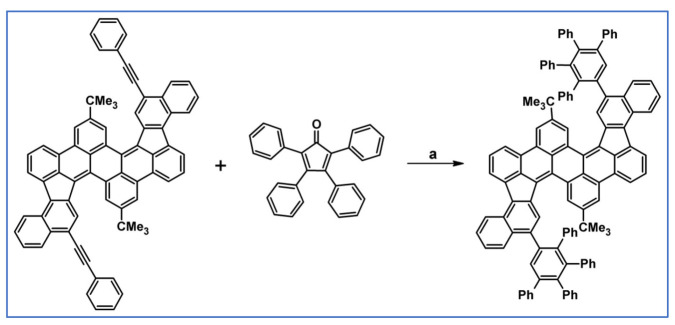
DA cycloaddition with CO extrusion as a final step in the synthesis of contorted PAH [235]. Reagents and conditions: a = Ph_2_O, 200 °C, 72 h, Y = 66%.

In a paper from 2019, Yang et al., described DA cycloaddition with CO extrusion as a final step in the multistep procedure leading to a contorted PAH (Scheme 183) [235].

**Scheme 184:**
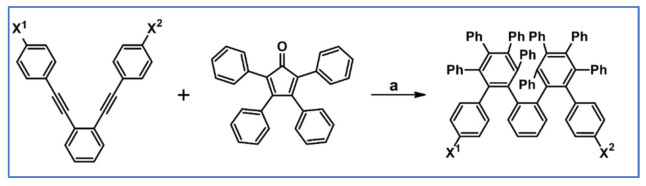
DA cycloaddition for the syntheses of 1,2-bis(pentaarylphenyl)benzenes [236]. Reagents and conditions: a = Ph_2_O, 260 °C, 48–72 h, Y = 70% (for X^1^ = X^2^ = H), Y = 35% (for X^1^ = H, X^2^ = OMe), Y = 15% (for X^1^ = X^2^ = OMe).

In 2019 the syntheses of 1,2-bis(pentaarylphenyl)benzenes via DA cycloaddition were presented by Nguyen’s research group (Scheme 184) [236].

The obtained compounds are characterized by configurational lability as the free energies of activation for the racemization of these compounds are only 20.3 kcal/mol.

**Scheme 185:**
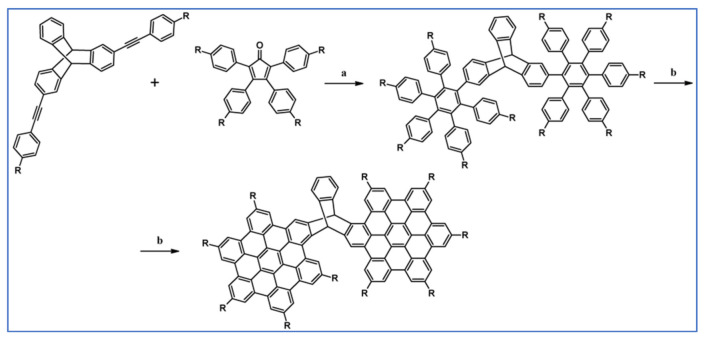
Synthesis of triptycene derivatives ((*R*,*R*)- and (*S*,*S*)-isomers) containing HBC moieties [237]. Reagents and conditions: R = *t*-Bu; a = Ph_2_O, 260 °C, 12 h, Y = 38% ((*R*,*R*)-enantiomer), Y = 37% ((*S*,*S*)-enantiomer); b = next step.

DA cycloaddition is also the important step in the synthesis of optically active triptycene-based molecules (pure (*R*,*R*)- and (*S*,*S*)-enantiomers) containing two hexabenzocoronene moieties (Scheme 185) as stated by Wada, Shinohara, and Ikai in 2019 [237].

One should emphasise that UV irradiation of the product results from the emission of circularly polarized light (with dissymmetry factors ~1.0 × 10^−3^).

**Scheme 186:**
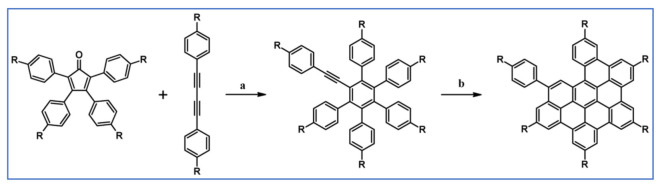
Pentacene derivative synthesis via DA cycloaddition-Scholl dehydrocondensation sequence [238]. Reagents and conditions: R = *t*-Bu; a = Ph_2_O, 250 °C, 24 h; Y = 59%; b = next step.

Rietsch and colleagues applied DA cycloaddition of corresponding butadiyne, namely 1,4-bis(4-(tertbutyl)phenyl)buta-1,3-diyne with cyclopentadienone as an initial step in the synthesis of a novel pentaphene derivative (Scheme 186) [238].

Such pentaphene is closely related to HBCs. However, it has 41 instead of 42 sp^2^-hybridized carbon atoms. Therefore, the synthesized pentaphene is chiral, and in the crystal lattice, there is a pair of enantiomers with the distance between the π-planes c.a. 1 nm. This type of substitution causes the pentaphene to exhibit increased fluorescence in both solution and solid state compared to HBC.

**Scheme 187:**
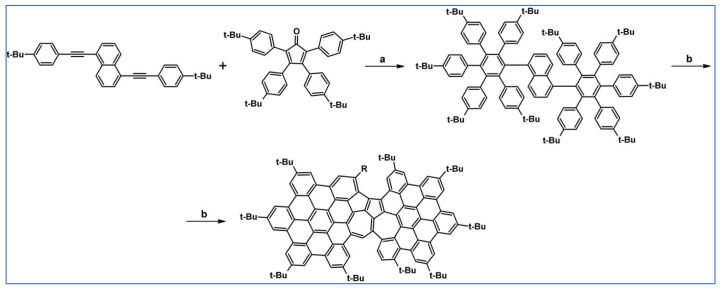
DA cycloaddition engaged in the synthesis of azulene-embedded-nanographene [239]. Reagents and conditions: a = Ph_2_O, rfx, Y = 67%; b = next step (Scholl reaction), up to 22% yield (for R = *t*-Bu); up to 27% yield (for R = OTf).

In 2020 typical DA cycloaddition with CO extrusion followed by Scholl reaction was applied by Han and colleagues in the synthesis of previously designed nanographene. Surprisingly, instead of the expected naphthalene-connected C_7_-ring containing nanographene, azulene-embedded nanographene was obtained as a unique Scholl reaction product (Scheme 187) [239].

The mechanism of the above-mentioned rearrangement of naphthalene into azulene as well as optical and electrochemical properties of the unexpectedly nanographene were thoroughly analyzed. It was also proven that the presence of azulene motif was decisive as far as the properties of the whole structure are concerned.

**Scheme 188:**
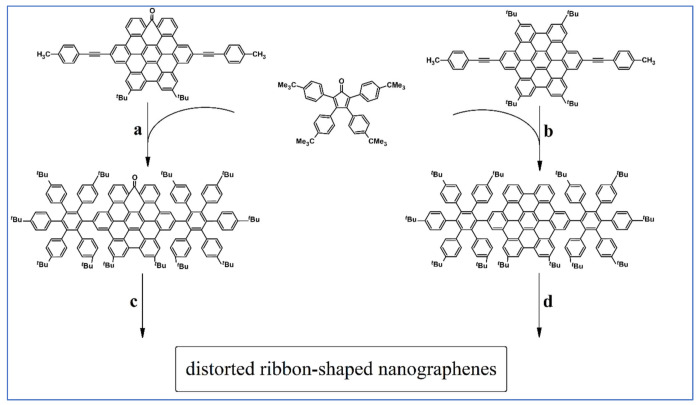
Synthetic routes leading to distorted ribbon-shaped nanographenes ingaging DA cycloaddition with CO extrusion followed by cyclodehysdrocondensation. Reagents and conditions: a = Ph_2_O, rfx, 8 h, Y = 45%; b = Ph_2_O, rfx, 16 h, Y = 29%; c, d = Scholl reaction.

In 2020, a new family of distorted ribbon-shaped nanographenes was designed, synthesized, and their optical and electrochemical properties were evaluated by Castro-Fernández et al. [23]. Namely, three nanographene ribbons were prepared using Diels–Alder cycloaddition of appropriate acetylenes to cyclopentadienones and, finally, Scholl reaction (Scheme 188).

It is worth noting that incorporating additional curvature has a beneficial impact in terms of non-linear optical properties of those ribbons. The idea that introducing distortions on nanographenes in a well-defined manner is a viable molecular engineering strategy to tune their properties for optoelectronic devices.

**Scheme 189:**
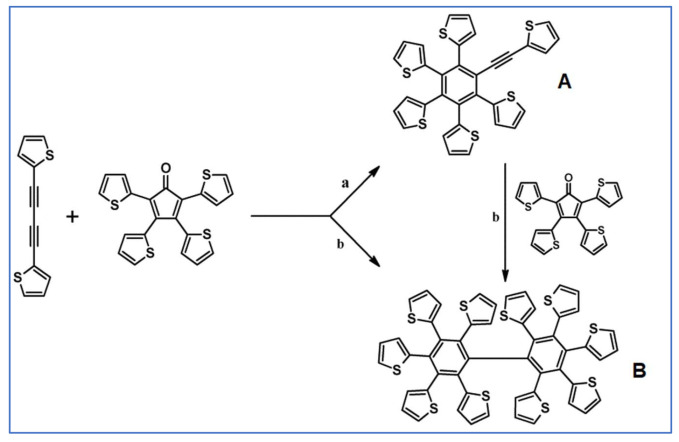
Synthesis of deca(2-thienyl)biphenyl via Diels–Alder cycloaddition (the yield of monoaddition product was equal 90%) [240]. Reagents and conditions: a = 1,2-dichlorobenzene, 200 °C, 18 h, Y = 90% (for **A**); b = 1,2-dichlorobenzene, 200 °C, MW, 4.5 h, Y = 7 or 14%, respectively (for **B**).

Thanks to Diels–Alder cycloaddition of 1,2-bis(2-thienyl)acetylene to tetra(2-thienyl)cyclopentadienone, sterically crowded and twisted thienylene-phenylenes were synthesized and characterized in comparison with analogous polyphenylene nanostructures (Scheme 189) [240]. Spectroscopic, diffraction, and theoretical studies indicated the through-space delocalization of π-electrons of peripheral (hetero)aromatic rings in catenated and toroidal topologies.

For many years, the syntheses of polyphenylene nanostructures have attracted considerable attention due to the improved availability of complicated distinct structures. We share the authors’ opinion that hexaphenylbenzenes (HPB) and similar compounds are strong candidates for application as photochemical and chemical switches, redox materials, liquid crystalline materials, molecular receptors, as well as in organic light-emitting diodes or molecular machines.

**Scheme 190:**
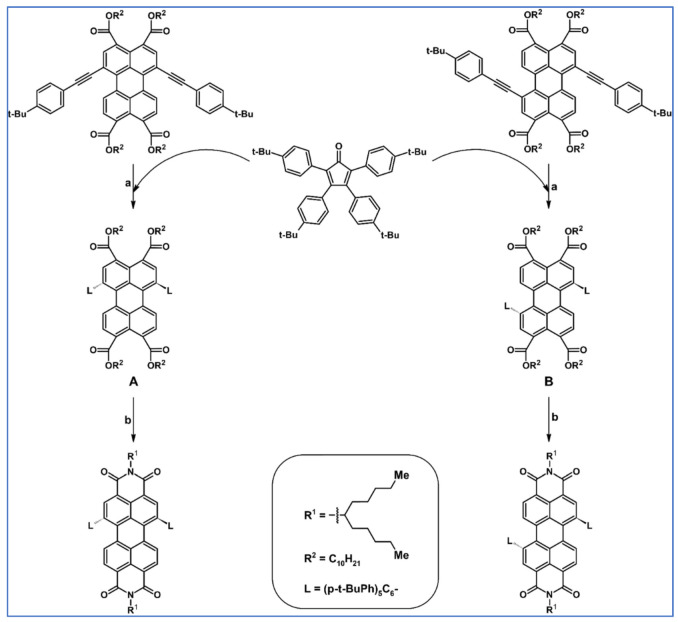
Syntheses of bay-disubstituted PBI: Ph(*p*-*t*-Bu-Ph)_5_ substituents formation via DA cycloaddition [241]. Reagents and conditions: a = toluene, 220 °C, 24 h, Y = 62% (for **A**) or 72% (for **B**); b = next step.

Mono- and, especially, disubstituted in bay region PBI derivatives were obtained by Dusdold and colleagues via multistep synthetic procedures engaging DA cycloaddition with CO extrusion as crucial steps (Scheme 190) [241].

The presence of bulky Ph(*p*-tert-Bu-Ph)_5_ substituents causes high core distortion of the obtained disubstituted derivatives but prevents aggregation domains arising. It is worth emphasizing that the photophysical properties of the PBI derivatives mentioned above are quite different from the peri-substituted PBIs.

#### 2.1.3. Cycloaddition of Cyclopentadienones and Dienophiles with Multiple Triple Bonds

**Scheme 191:**
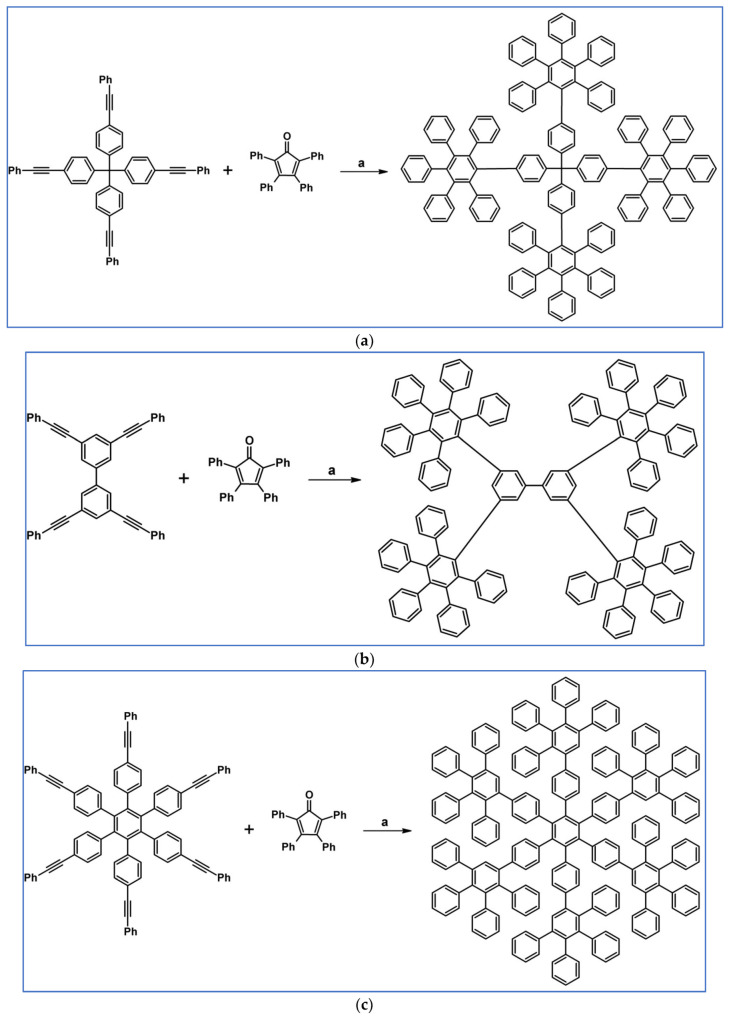
Syntheses of polyphenylene dendrimers via DA cycloaddition between tetraphenylcyclopentadienone and appropriate phenylethynyl derivatives [242]. Reagents and conditions: (**a**–**c**) a = Ph_2_O, rfx, 48 h, yield up to quantitative.

As reported by Bauer et al., in 2002, a series of first-generation polyphenylene dendrimers based on three core variants were prepared by DA cycloaddition between tetraphenylcyclopentadienone and adequately substituted acetylenes, with CO extrusion. The most prominent structure among those obtained is presented in Scheme 191 [242].

X-ray analysis of the obtained dendrimers showed that their channel-like crystal structure could be used to create guest–host type systems with rod-like shape molecules. The structure of dendrimers comes from the complete stop of the rotation around a single bond, which causes stiffening of phenyl moieties positions.

**Scheme 192:**
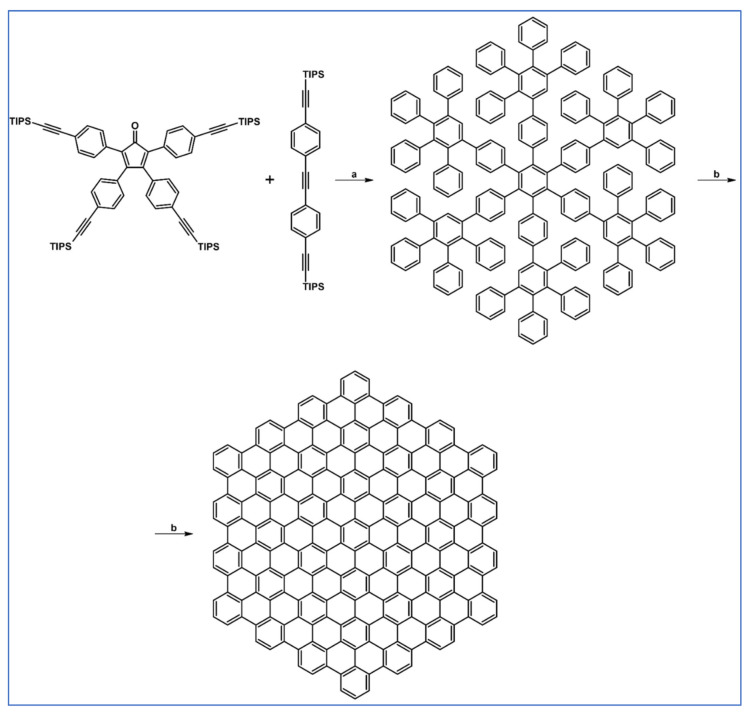
DA cycloaddition and Scholl reaction involved in the synthetic route leading to the graphite sheet [243]. Reagents and conditions: a = Ph_2_O, 200 °C, 11 days, Y = 72%; b = next step (Scholl reaction).

Simpson et al., applied the APEX bottom-up strategy involving DA cycloaddition with CO extrusion and Scholl cyclodehydrocondensation in the synthetic path leading to the PAH containing 222 carbon atoms (Scheme 192) [243]. This nanographene can also be obtained via Co-mediated cyclotrimerization of acetylene depicted in scheme below, followed by Scholl reaction.

**Scheme 193:**
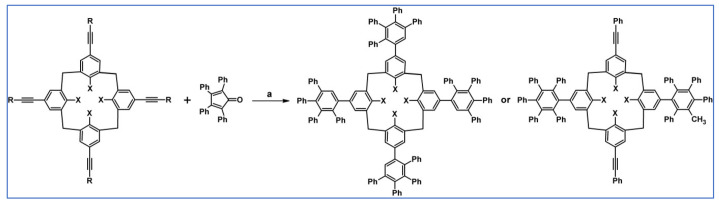
Syntheses of polyphenylenic calix[4]arenes via DA cycloaddition with CO extrusion [244]. Reagents and conditions: a = Ph_2_O, 190 °C, 72 h, Y= 85% (for R = H) and Y= 72% (for R = Ph).

DA cycloaddition with CO extrusion between tetraethynylcalix[4]arene or tetrakis(phenylethynyl)calix[4]arene (as dienophiles) and tetraphenylcyclopentadienone (as diene) was described by Mastalerz et al., (Scheme 193) [244].

The derivatives shown in the scheme above were designed to act as precursors of nonplanar polyaromatic hydrocarbons after dehydrocondensation.

**Scheme 194:**
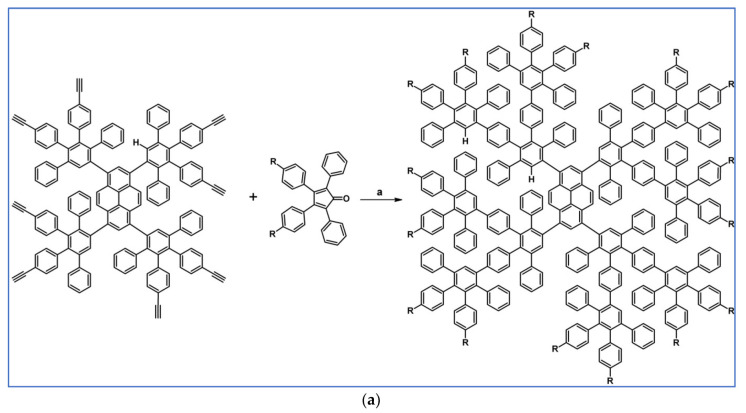

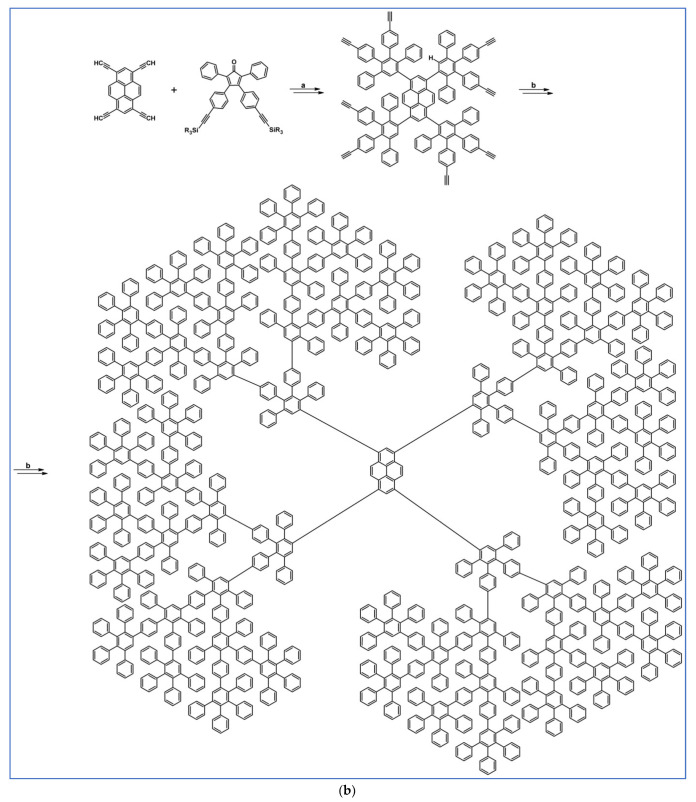
DA cycloaddition as a crucial step in the synthesis of dendrimers: second generation (with solubilizing peripheral groups) and fourth generation (without peripheral alkyl groups) [245]. Reagents and conditions: (**a**) (a) R = H; a = *o*-xylene, 160 °C, 16 h, Y = 73%; (b) R = 3-decyltetradecan-1-yl; a = Ph_2_O, 220 °C, 16 h, Y= 63%; (**b**) R = *i*-Pr; a = o-xylene, 160 °C, 12 h, Y = 71%, then TBAF, THF, Y = 93%; b = next steps.

Involving DA cycloaddition of appropriately substituted acetylene (fourfold ethynyl-substituted 1,3,6,8-tetraethynylpyrene as core) and cyclopentadienone derivatives as dienes, a series of polyphenylene dendrimers (up to fourth generation) was prepared in high yield by combining divergent and convergent growth methods by Bernhardt and co-workers [245]. The scheme below (Scheme 194) presents examples of syntheses leading to second- and fourth-generation dendrimers.

It was confirmed that a second-generation dendrimer layer is needed to shield the encapsulated pyrene chromophore and prevent aggregate formation efficiently. Additionally, macromolecules presented in the above-mentioned article are characterized by combining excellent optical features (fluorescent quantum yield > 0.92) and improved film-forming ability. Such a set of properties, especially as far as the second-generation dendrimer bearing solubilizing group is concerned, makes them attractive for electronic devices, OLEDs especially.

**Scheme 195:**
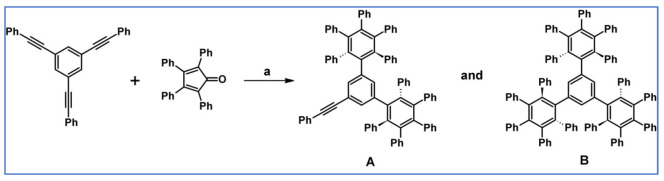
Syntheses of 1-phenylethynyl-3,5-bis(pentaphenylphenyl)benzene (**A**) and 1,3,5-tris(pentaphenylphenyl)benzene (**B**) via DA cycloaddition with CO extrusion [246]. Reagents and conditions: a = Ph_2_O, 230 °C, 120–380 h, Y = not given.

Highly crowded polyphenylenes, namely 1-phenylethynyl-3,5-bis(pentaphenylphenyl)benzene (**A**) and 1,3,5-tris(pentaphenylphenyl)benzene (**B**) were obtained via DA cycloaddition (Scheme 195) as Komber, Stumpe, and Voit reported in 2007 [246].

Using dynamic NMR, it was claimed that both products possess two rotation barriers resulting from the rotation around single bonds. One of them refers to the rotation of Ph_5_Ph groups connected with benzene core, while the other stems from the rotation of terminal Ph-substituents present in Ph_5_Ph units.

**Scheme 196:**
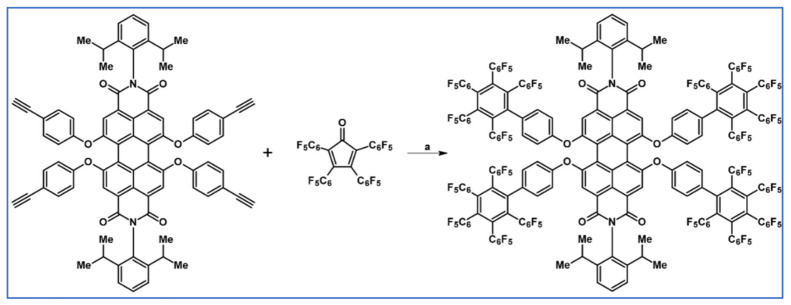
DA cycloaddition as a crucial step in the synthesis of PBI-derivative bearing perfluorinated motifs [247]. Crystal structure of the obtained PBI-derivative. Reagents and conditions: a = *o*-xylene, rfx, 7 d; Y = 81%.

A perylene bisimide derivative (PBI-derivative) with fluorinated shell was prepared by Liu and co-workers via DA cycloaddition in a crucial step (Scheme 196) [247]. Due to its structure, the obtained PBI-derivative possesses remarkably stable amorphous state, further increased electron affinity, efficient and independent red emission.

Three-dimensional non-planar molecular structure of the obtained PDI was achieved by introducing bulky branches. Thanks to their properties, namely, optical (high luminescent quantum yield), thermal (excellent stability), excellent amorphous stability, and electrochemical features, the obtained PDI can be qualified as a potential multifunctional material, i.e., *n*-type red emitter for non-doped OLEDs and electron acceptor for solar cells [145].

**Scheme 197:**
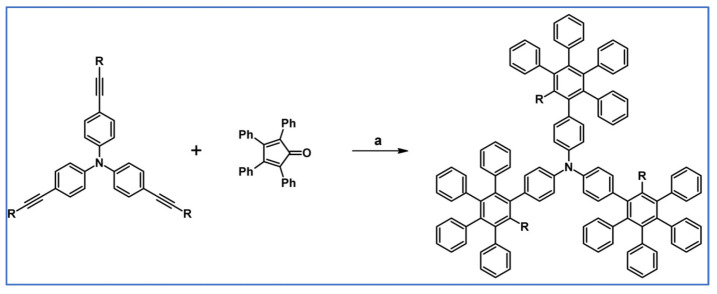
DA cycloaddition between tetraphenylcyclopentadienone and appropriately substituted tris(ethynylphenyl)amine [248]. Reagents and conditions: R = H, Me, Ph; a = Ph_2_O, heating, up to 92% yield.

Gagnon et al., applied DA cycloaddition with CO extrusion in the synthesis of triaryloamines designed to constrain or even prevent crystallization and form long-lived molecular glasses (Scheme 197) [248].

It was suggested that methylpentaphenyl substituents might be particularly effective in inhibiting crystallization, both by enforcing highly nonplanar topologies that pack poorly, and by interfering with normal molecular association induced by *π*⋯*π* and C-H⋯*π* aromatic interactions.

**Scheme 198:**
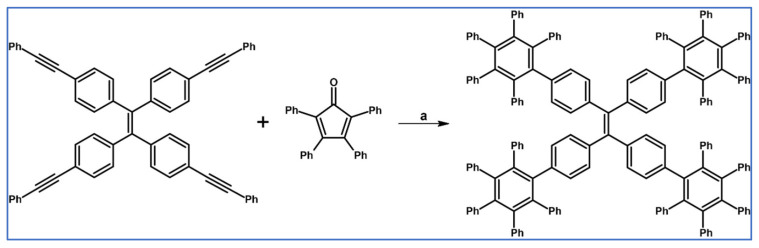
DA cycloaddition involved in the synthesis of dedicated to optoelectronics (Ph_5_Ph)_4_C=C(PhPh_5_)_4_ [249]. Reagents and conditions: a = Ph_2_O, rfx, 24 h, Y = 74%.

Hexaphenylethylene derivative bearing four Ph_5_Ph motifs was obtained via DA cycloaddition with CO extrusion, using pentaphenylcyclopentadienone and tetra(phenylethynyl)ethene as diene and dienophile, respectively (Scheme 198) [249].

Thanks to steric repulsion created by bulk Ph_5_Ph substituents, the obtained derivative is emissive, contrary to non-emissive tetraphenylethylene. The steric repulsion mentioned above causes limited rotation around C-C bonds in (Ph_5_Ph)_4_C=C(PhPh_5_)_4_, which practically eliminates the non-radiative deactivation of the excited state.

**Scheme 199:**
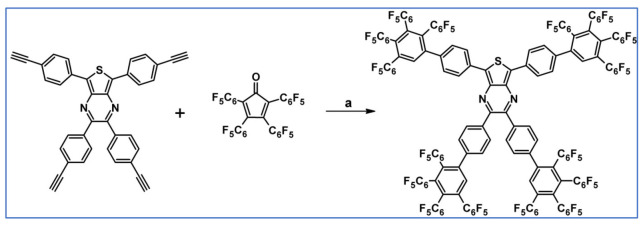
Synthesis of thieno-[3,4-*b*]-pyrazine derivative possessing four –C_6_H_4_–C_6_H(2,3,4,5–C_6_F_5_)_4_ groups via DA cycloaddition [250]. Reagents and conditions: a = *o*-xylene, rfx, 72 h, Y = 41%.

A thieno-[3,4-*b*]-pyrazine derivative bearing four –C_6_H_4_–C_6_H(2,3,4,5–C_6_F_5_)_4_ groups was obtained via DA cycloaddition with CO extrusion involving tetrakis(pentafluorophenyl)cyclopentadienone and tetra(phenylethynyl)thieno-[3,4-*b*]-pyrazine as substrates (Scheme 199) [250].

Moieties, namely –C_6_H_4_–C_6_H(2,3,4,5–C_6_F_5_)_4_, present in the product structure perform several important functions: ensure good thermal stability and large Stokes shift, prevent emission quenching (as they almost eliminate intermolecular stacking), and facilitate electron injection (thanks to lowering LUMO energy). Due to the above-mentioned advantages, the commented compound is an attractive OLED material characterized by red electroluminescence and good brightness and luminance.

**Scheme 200:**
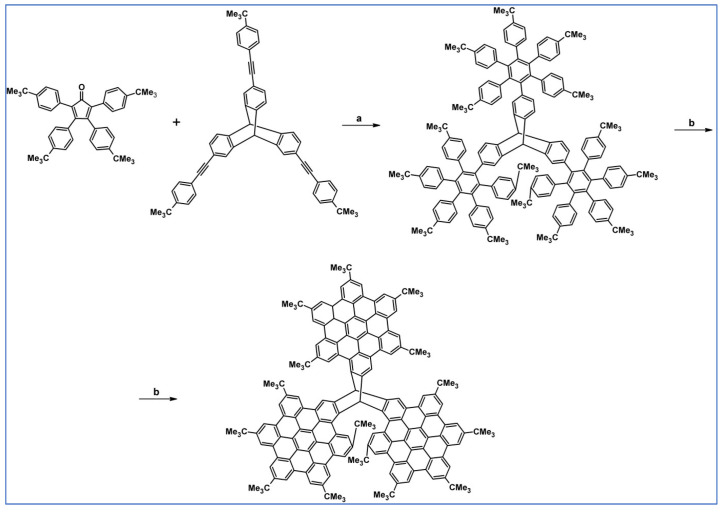
DA cycloaddition with CO extrusion involved in the syntheses of HBC-based triptycenes (the derivative bearings tri-HBC-moieties is shown on the scheme) [251]. Reagents and conditions: a = Ph_2_O, rfx, 48 h, Y = not given; b = next step (Scholl reaction).

A peculiar series of hexa-peri-hexabenzocoronene-based triptycenes bearing mono-, di-, or tri-HBC moieties were synthesized using appropriate ethynyl triptycene derivative, tetraphenylcyclopentadienone through DA cycloaddition with CO extrusion (Scheme 200) [251].

It is worth emphasizing that the HBC are well distributed in the triptycene core, so no fluorescence quenching of each HBC unit is observed.

**Scheme 201:**
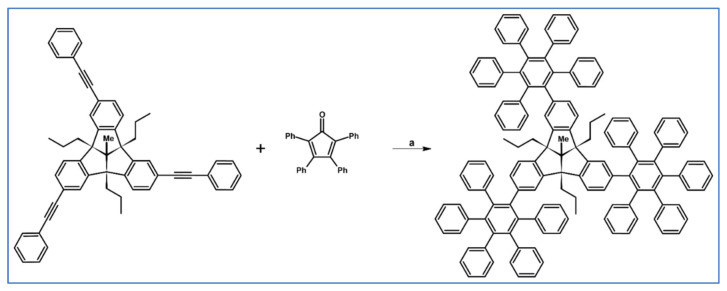
DA cycloaddition with CO extrusion as a useful tool for synthesis of TBTQs bearing tetra(phenyl)phenyl moieties [252]. Reagents and conditions: a = Ph_2_O, 250 °C, 2 d, Y = 76%.

DA cycloaddition was employed by Mughal et al., in the syntheses of tribenzotriquinacenes (TBTQs) substituted with three phenylethynyl moieties at the periphery of bowl-shaped macrocycles (Scheme 201 (an example)) [252].

**Scheme 202:**
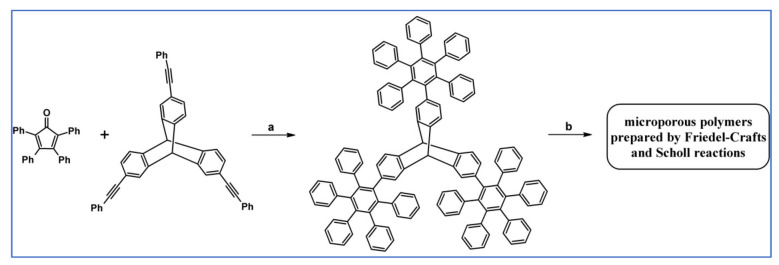
Synthesis of triptycene bearing three HPB-motifs via DA cycloaddition with CO extrusion [253]. Reagents and conditions: a = Ph_2_O, rfx, 72 h, Y = 52%; b = next step.

In 2016, Zhang et al., proposed the synthesis route of hexaphenylbenzene-based triptycene monomer from triethynyltriptycene and tetraphenylcyclopentadienone via DA cycloaddition with CO extrusion (Scheme 202) [253].

Engaging Friedel–Crafts and Scholl reactions, two types of microporous polymers were synthesised from the above-presented monomers. Obtained polymers vary in size of the surface (569 and 914 m^2^/g for products obtained by Friedel–Crafts and Scholl, respectively) and ability to reversible H_2_ and CO_2_ adsorption.

**Scheme 203:**
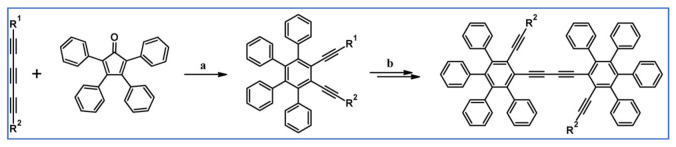
DA cycloaddition of tetraphenylcyclopentadienone and 1,6-disubstituted-1,3,5-hexatriynes as an initial step in the syntheses of 1,4-bis[(tetraphenyl-arylethynyl)phenyl]-1,3-butadiynes [254]. Reagents and conditions: R^1^, R^2^ = Si*i*Pr_3_, Si*i*Pr_3_; Si*i*Pr_3_, SiMe_3_ and others; a = thermal (250 °C in dichlorobenzenes) or MW (210 °C in xylene) conditions; b = next steps.

Sale et al., used the Diels–Alder cycloaddition reactions of 1,3,5-hexatriynes with tetraphenylcyclopentadienone as a crucial step for the synthesis of 1,4-bis[(hexasubstituted)phenyl]-1,3-butadiynes (Scheme 203) [254].

**Scheme 204:**
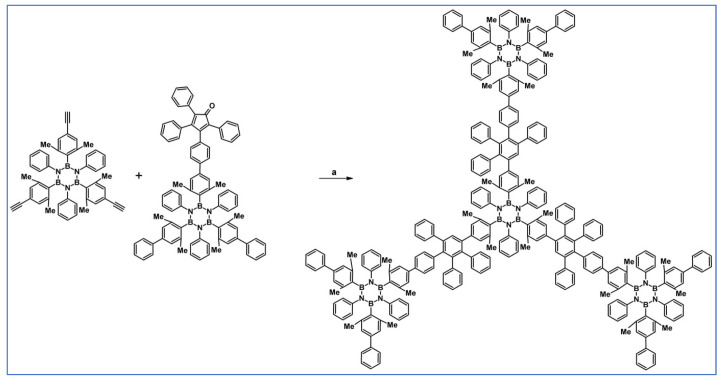
DA cycloaddition as a crucial step in the synthesis of star-shaped BN-doped polyphenylene—the first example [255]. Reagents and conditions: a = Ph_2_O, 190 °C, 18 h, Y = 22%.

**Scheme 205:**
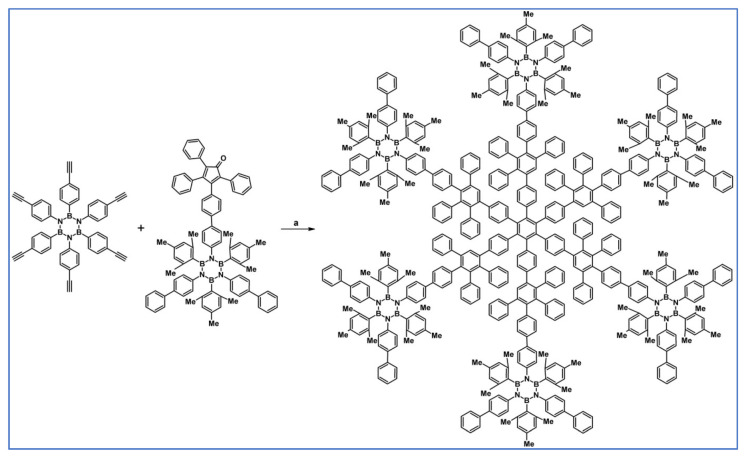
DA cycloaddition as a crucial step in the synthesis of BN-doped polyphenylene—the second example [255]. Reagents and conditions: a = Ph_2_O, 230 °C, 16 h, Y = 19%.

In 2017, Marinelli et al., described the application of DA cycloaddition with CO extrusion for the syntheses of two series of BN-doped polyphenylenes of various shapes, including star-shaped ones. BN-doping was realized by adding one or more borazine moieties to the structure (Scheme 204 and Scheme 205) [255]. It should be added that sterically hindered aryl groups protected all *B*-atoms in the borazine rings to ensure the final structure thermal stability.

The examination of photophysical properties of the obtained BN-doped nanostructures revealed that the increase in doping degree strongly and negatively influences their luminescence. It suggests that the growing number of B_3_N_3_ moieties in the doped-polyphenylenes structure strongly increases the participation of nonradiative deactivation of the excited states. Interestingly, such an effect was observed only in case of low-BN-doped structures. However, the authors add that planarization of obtained BN-doped structures leading to regularly BN-doped nanographenes, e.g., via cyclodehydrocondensation, remains a challenge. Moreover, it is a limitation for the bottom-up strategy used to obtain such structures.

**Scheme 206:**
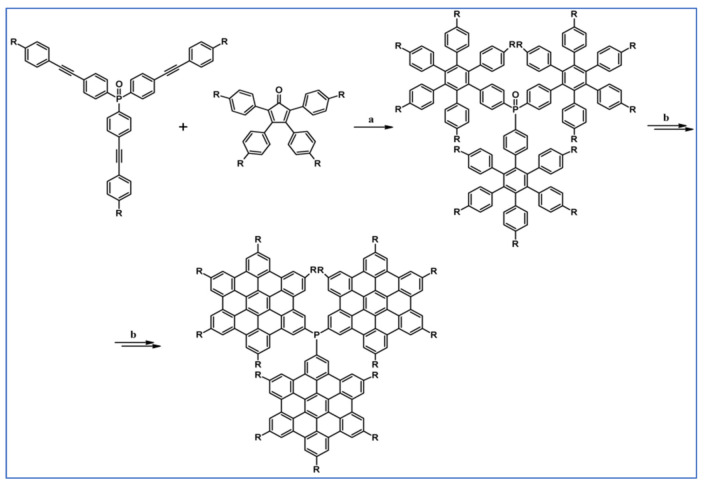
Superphenylphosphines synthesis: Diels–Alder cycloaddition (tetrarylcyclopentadienone as a diene), Scholl dehydrocondensation, reduction of P=O bond—an example [256]. Reagents and conditions: R = *t*-Bu; a = Ph_2_O, 250 °C, Y = 92%; b = next steps (Scholl dehydrocondensation).

Smith et al., described a series of triarylphosphines that bear large PAH substituents able of engaging in strong π-stacked interactions [256]. HBC-motif was introduced via Diels–Alder cycloaddition–Scholl dehydrocondensation sequences (Scheme 206).

**Scheme 207:**
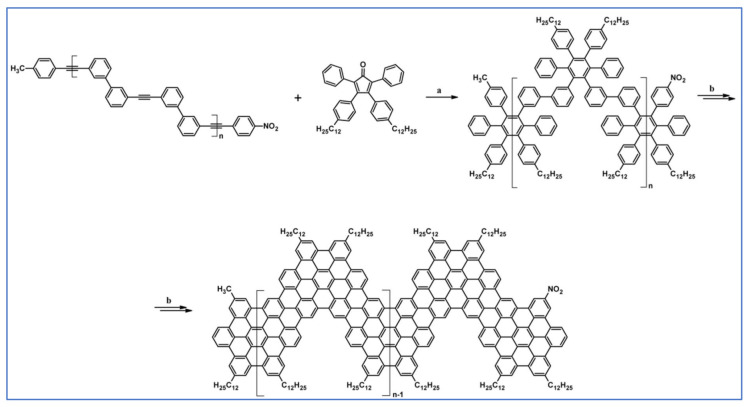
DA cycloaddition of acetylene and cyclopentadienone derivatives in the multi-step syntheses of graphene nanoribbons [257]. Reagents and conditions: a = Ph_2_O, sulfolane, 250 °C, 48 h, MW; Y= 60%; b = next steps.

The same year, Kugelgen et al., presented atomically precise bottom-up synthesized graphene nanoribbons (GNRs) as promising candidates for next-generation electronic materials [257]. DA cycloaddition of cyclopentadiene and polymeric acetylene (provided via ROMP) was involved synthetically (Scheme 207).

It should be emphasized that cutting-edge synthetic tools applied in this work allowed to fully control the implementation of the obtained semiconductors into devices characterized by complex architectures. Controlling or tuning the size, sequence of structural elements, types of end-groups and hierarchical assemblies turned out to be essential. Moreover, using copolymer templating strategy will enable integration leading to well-defined herojunctions and single-graphene nanoribbons transistors.

**Scheme 208:**
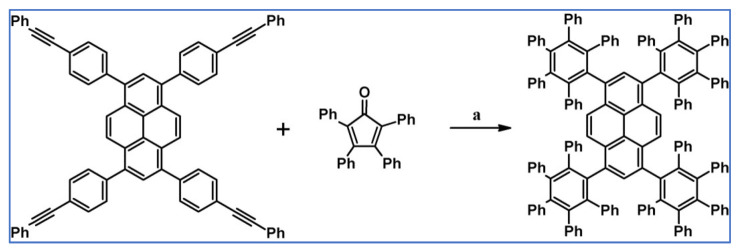
DA cycloaddition for the synthesis of 1,3,6,8-tetra(pentaphenylphenyl)pyrene [258]. Reagents and conditions: a = Ph_2_O, 230 °C, 24 h, Y = 72%.

Vyas et al., described the synthesis of 1,3,6,8-tetraaryl substituted pyrenes, including tetra(pentaphenylphenyl) ones [258]. The latter was obtained via DA cycloaddition of tetra(phenylethynyl)pyrene with tetraphenylcyclopentadiene (Scheme 208).

It was stated that the substituents (substituted phenyl or Ph_5_P) influence the optoelectronic properties of the pyrene core indicating a strong coupling between the pyrene core and the aryl groups. Additionally, the aryl groups attached to the 1-, 3-, 6-, and 8- positions inhibit the π-stacking of the pyrene core in solution.

**Scheme 209:**
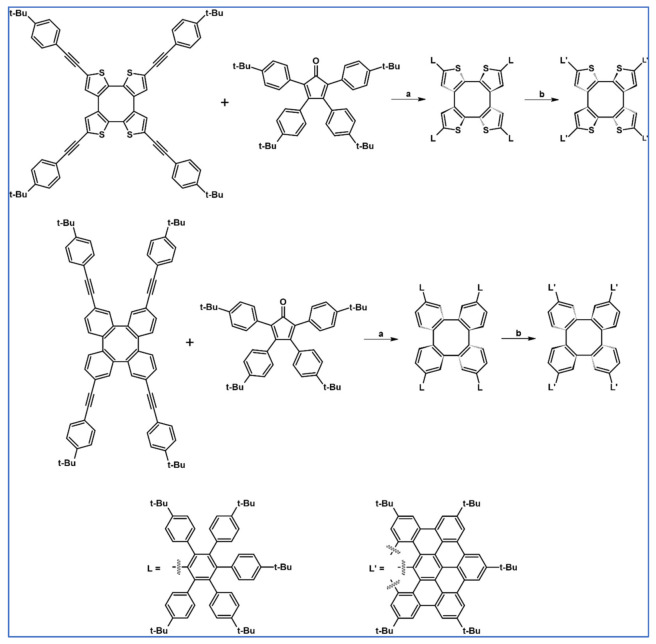
DA cycloaddition and Scholl dehydrocondensation as powerful tools for the synthesis of nonographenes constructed around the cyclooctatetraene core [259]. Reagents and conditions: a = MW, 300 °C, 0.3 h; Y = 32–47%; b = next step (Scholl reaction).

In 2020, Urieta-Mora et al., described two novel homo and hetero three-dimensional nanographenes, featuring a cyclooctatetraene core synthesized with the aid of DA cycloaddition with CO extrusion followed by Scholl dehydrocondensation (Scheme 209) [259].

Notably, both of the above-presented saddle-like nanographenes are well soluble in common organic solvents. Moreover, due to their electron-rich structures, both discussed nanographenes are chemically and electrochemically stable.

#### 2.1.4. Cycloaddition of Cyclopentadienones and Mixed Acetylenes

**Scheme 210:**
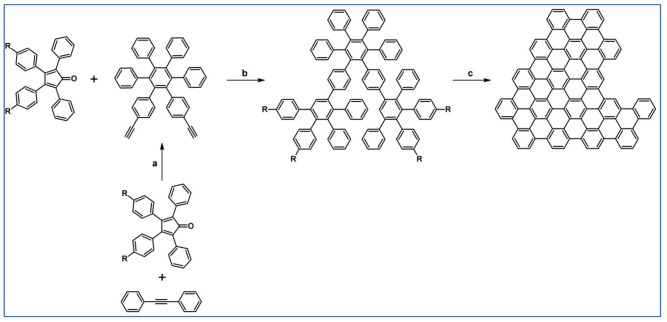
From three-dimensional to two-dimensional structures: DA cycloaddition with CO extrusion followed by flaterization of obtained dendrimer—an example of synthetic procedures [260]. Reagents and conditions: R = C≡C–Si(*i*-Pr)_3_; a = Ph_2_O, rfx, Y = over 90%; b = Ph_2_O + 1-methylnaphthalene, 1.5 h, Y = over 85%; c = next step (Scholl reaction).

The tandem approach of DA cycloaddition followed by cyclodehydrocondesation and using suitable substrates (namely cyclopentadienones and acetylenes) was essential for the synthetic route from highly branched dendrimers to the flat PAHs (Scheme 210) [260,261].

It is worth stressing that Si(*i*-Pr)_3_ groups present in diene structures protect from unexpected DA cycloaddition at the unfavourable stage of the procedure. Only after deprotection does the cycloaddition to the freed from steric hindrance triple bond occur at the preferable stage. The same idea, namely applying DA cycloaddition followed by Scholl dehydrocondensation, was the base for synthesising various polyphenylbenzenes and, eventually, obtaining superacenes [261].

**Scheme 211:**
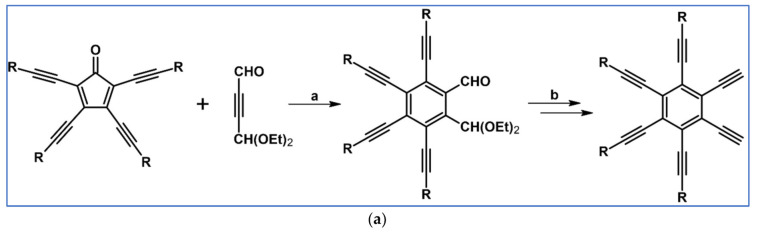

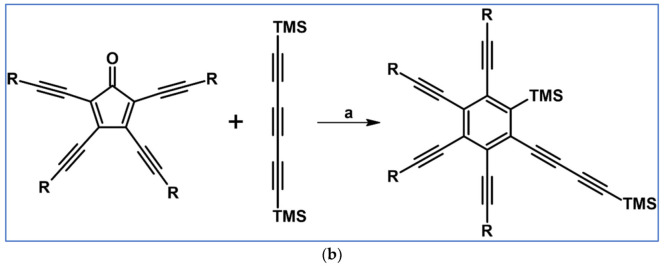
DA cycloaddition with CO extrusion as a crucial step in the synthesis of hexa(ethynyl)benzene and penta(ethynyl)(buta-1,3-dienyl) benzene [262]. Reagents and conditions: (**a**) R = TIPS; a = PhMe, rfx; Y = 90%; b = next steps; (**b**) R = TIPS; a = PhMe, rfx; Y = 73%.

In 1997 Tobe, Kubota, and Naemura described synthesis of hexa(ethynyl)benzene and penta(ethynyl)(buta-1,3-dienyl) benzene via DA cycloaddition of adequately substituted cyclopentadienone and acetylene or 1,3,5-hexatriene (Scheme 211) [262].

It is worth noting that cycloaddition of conjugated triyne was regioselective.

**Scheme 212:**
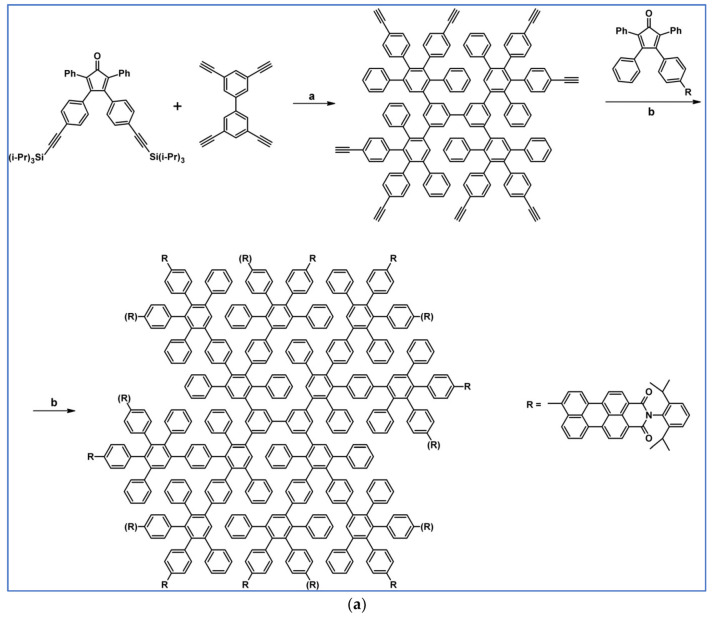

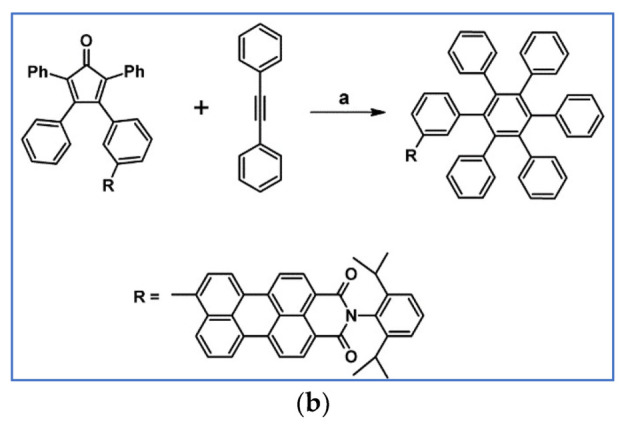
DA cycloaddition with CO extrusion involved in the syntheses of a first and second generation of polyphenylene dendrimers bearing monoperyleneimide motifs as a chromophore [263]. Reagents and conditions: (**a**) (R) = alternative position of substituents R; a = initial step, b = Ph_2_O, 195 °C, Y = 82%; (**b**) a = Ph_2_O, 260 °C, Y = 85%.

In 1999, repetitive and straightforward DA cycloaddition between appropriately substituted acetylenic-dienophile and cyclopentadienones was engaged in the synthetic route leading to dendrimers bearing various perylenemonoimide chromophores (Scheme 212) [263].

According to the authors, taking into consideration fluorescence properties of dendrimers, these studies offer the possibility of closing the gap between single-molecule studies on single chromophores and multichromophoric systems, such as fluorescent polymers, phycoerythrin, and light-harvesting protein complexes from the photosynthetic apparatus.

**Scheme 213:**
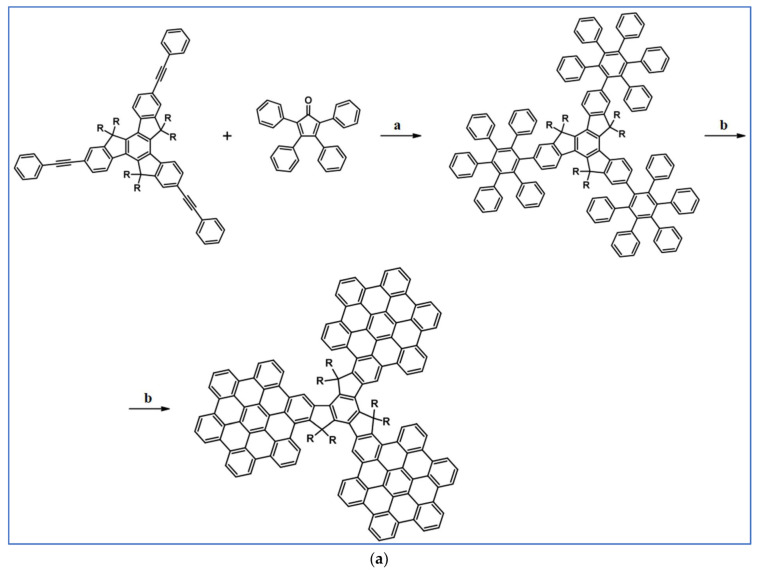

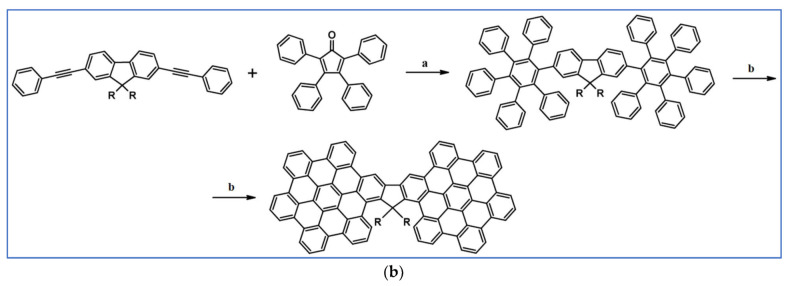
DA Cycloaddition as a crucial step in the synthesis of HBC and fluorene motifs [264]. Reagents and conditions: (**a**) a = Ph_2_O, rfx, overnight, Y = 90%; (**b**) a = Ph_2_O, rfx, overnight, Y = 93%; b = Scholl dehydrocondensation.

The utilization of truxene and fluorene moieties as basic building blocks and DA cycloaddition to construct large star-shaped PAHs family containing HBC chromophores has been presented by Cao and colleagues (Scheme 213) [264].

A high tendency to aggregation characterizes the obtained HBC derivatives and, due to their electronic and photoelectronic properties, they are interesting objects to research for organic electronics.

**Scheme 214:**
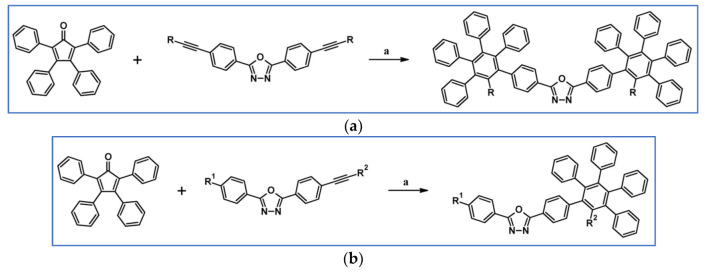
Synthesis of polyphenylenes contained oxadiazole linker: DA cycloaddition as a crucial step [265]. Reagents and conditions: (**a**) R = H or Ph; a = *m*-xylene, rfx, 12 h; (**b**) R^1^ = H or OMe, R^2^ = H or Ph; a = *m*-xylene, rfx, 12 h; up to 95% yield.

A series of polyphenylenes containing an oxadiazole linker were prepared in high yields by a convenient synthetic procedure involving DA cycloaddition with CO extrusion (Scheme 214) according to a report from Chen et al. [265].

The obtained polyphenylene nanostructures are characterized by exciting channel-forming structure and dynamic properties thanks to the presence of wholly interlocked, twisted phenyl rings. The polyphenylene-dendronized material has a sterically crowded structure, efficiently blocking π–π stacking and increasing thermal stability. The photophysical and reversible electrochemical properties result in improved electron transport properties of materials. Blue electrophosphorescent devices fabricated using these materials, and iridium(III)bis[4,6-di-fluorophenylpyridinato-*N*,C^2^′]picolinate (as host materials and the blue phosphorescent dopant emitter, respectively) turned out to be promising.

**Scheme 215:**
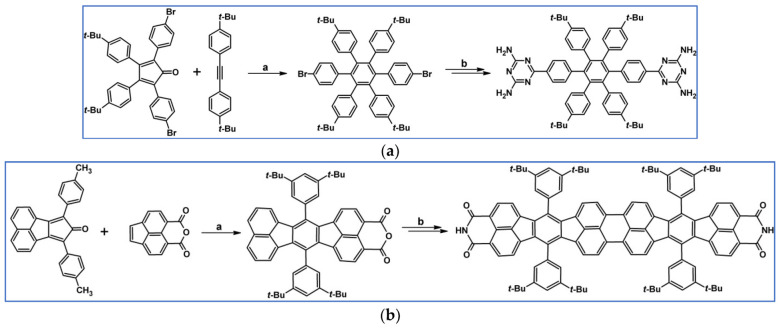
DA cycloaddition with CO or CO and H_2_ extrusion involved in the syntheses of molecular moulds components [266]. Reagents and conditions: (**a**) a = Ph_2_O, MW (300 W), 250 °C, 45 min, Y = 65%; b = next steps; (**b**) a = *o*-xylene, MW (300 W), 210 °C, 40 min, Y = 52%; b = next steps.

Barratin’s research group described DA with CO extrusion as crucial in synthesizing two complementary molecular moulds (Scheme 215) [266]. One should pay attention to the fact that acetylene plays the role of dienophile in the first reaction, while in the second, the alkene is a dienophile, i.e., acenaphthene derivative. Due to steric repulsion, acenaphthene reacts as masked acetylene.

The molecules shown in the scheme comprise a polyaromatic central part and four bulky lateral tert-butylphenyl or di-tert-butylphenyl groups. After the deposition of such molecules onto a surface, a prone to metallic moulding cavity is created.

**Scheme 216:**
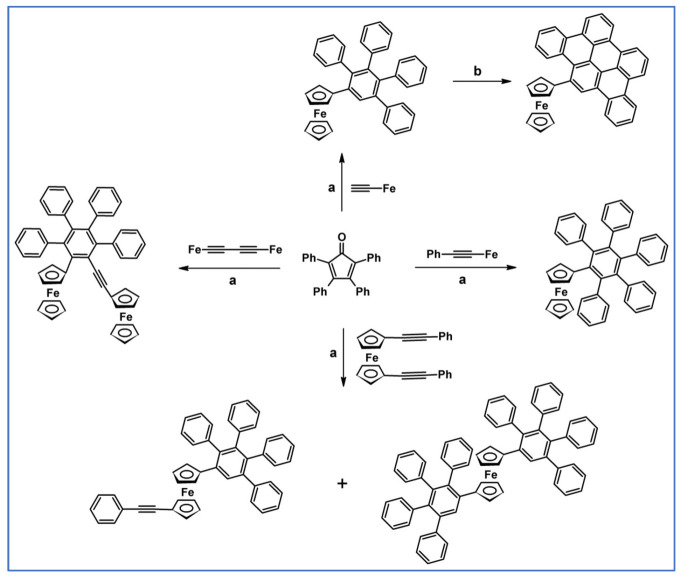
DA cycloaddition with CO extrusion in synthetic route providing ferrocene derivatives bearing tetra- or pentaphenyl or eight-ring fused PAH-moieties [267]. Reagents and conditions: a = Ph_2_CO, 200 °C, 48 h; Y = up to 70%; b = next step (Scholl reaction).

According to a paper from 2010, a series of ferrocenyl-substituted polyphenylenes was obtained involving tetraphenylcyclopentadienone acetylenes bearing ferrocenyl moiety and DA cycloaddition with CO extrusion (Scheme 216) [267]. Moreover, [(tetraphenyl)phenyl]ferrocene, formed via the above-mentioned DA cycloaddition, underwent Lewis acid-catalyzed cyclodehydrogenation, resulting in ferrocene with an eight-ring fused PAHs-substituent [267].

The complete spectroscopic characterization and all the obtained systems’ electronic and redox properties were described and compared.

**Scheme 217:**
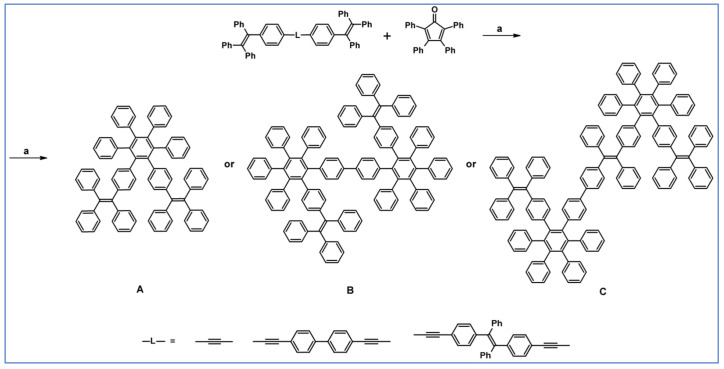
Syntheses of nonplanar tetraphenylethene-hexaphenylbenzene cycloadducts using DA cycloaddition with CO extrusion [268]. Reagents and conditions: a = DA reaction, rfx, 48 h; Y = 32% (for **A**) or 37% (for **B**) or 44% (for **C**).

In 2013, Hu et al., synthesized a series of luminogenic materials via DA cycloaddition using acetylene precursors bearing tetraphenylethene moiety and tetraphenyloctyclopentadienone as a dienophile and diene, respectively (Scheme 217) [268].

All of the obtained compounds showed AIE with high solid-state fluorescence quantum yields up to 87%. The twisting amplitude and steric hindrance of the tetraphenylethene and HPB motifs play an essential role in fluorescence behaviours of the above-mentioned luminogenic materials in their aggregated state.

**Scheme 218:**
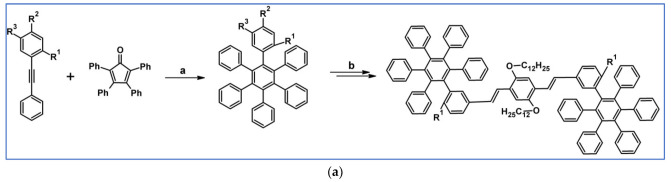

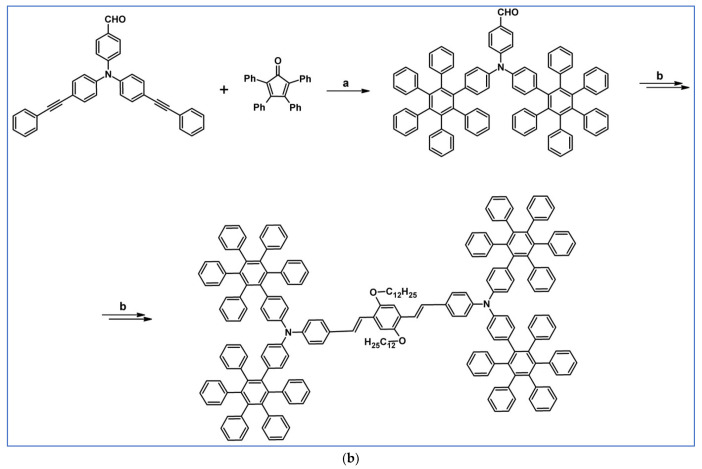
DA cycloaddition between tetraphenylcyclopentadienone and appropriate acetylene and diyne involved in the syntheses of HPB end-capped tri(*p*-phenylenevinylenes) [269]. Reagents and conditions: (**a**) a = Ph_2_O, rfx, 6 h; Y = 74% (for R^1^ = R^3^ = H, R^2^ = CHO) or Y = 65% (for R^1^ = OMe, R^3^ = H, R^2^ = CHO); R^1^ = H or MeO (in final product); b = next steps; (**b**) a = Ph_2_O, rfx, 6 h, Y = 60%; b = next steps.

Jana and Ghora described DA with CO extrusion employed in a synthetic pathway to three HPB-derivatives, i.e., HPB end-capped tri(*p*-phenylenevinylenes) with high overall yield (up to 86%) (Scheme 218) [269].

The synthesized hexaphenylbenzene end-capped tri(*p*-phenylenevinylenes) are bright fluorescent molecules (in the blue to blue-green region) with high emission efficiency in solution. Moreover, these compounds display good electrochemical reversibility as well as high thermal stability and high values of HOMO.

**Scheme 219:**
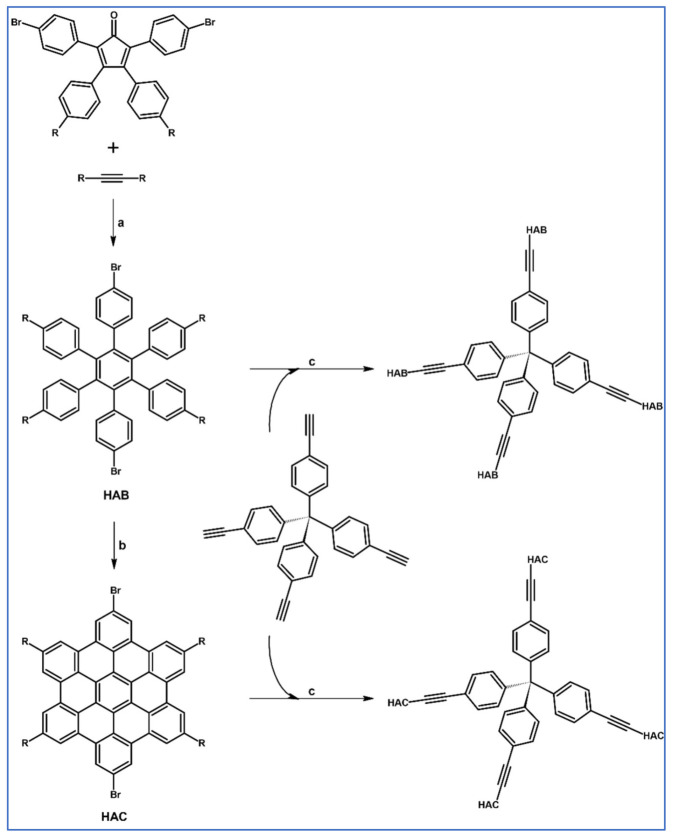
DA cycloaddition–Sonogashira cross-coupling and DA cycloaddition–Scholl reaction–Sonogashira cross-coupling tandems as a synthetic tool for obtaining porous polymers [270]. Reagents and conditions: R = H, Me, *t*-Bu; a = Ph_2_O, 250 °C, MW (300 W); b, c = next steps.

In the paper from 2014, Thompson and colleagues presented DA cycloaddition with CO extrusion as a crucial step in the synthetic route providing porous polymers, in which hexaarylbenzene or hexaarylcoronene motifs are connected through ethynyl-aryl-diarylmethylene-aryl-ethynyl- bridge (Scheme 219) [270].

Porosity and gas-sorption ability of polymers A and B strongly depended on R-substituents, due to the steric repulsion of the peripheral substituent and diverse ability to π-stacking. Interestingly, changing H into Me and finally into *t*-Bu resulted in increased porosity A but decreased porosity B.

**Scheme 220:**
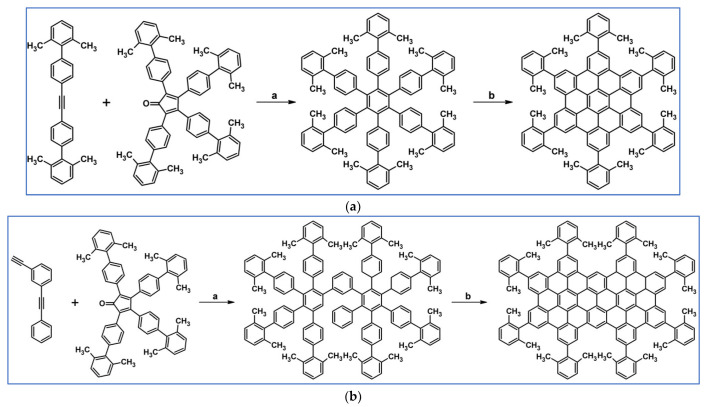
Syntheses of edge-functionalized nanographenes via DA cycloaddition–Scholl reaction [271]. Reagents and conditions: (**a**) a = Ph_2_O, rfx, 48 h, Y = 81%; b = next step (Scholl reaction); (**b**) a = Ph_2_O, rfx, 49 h, Y = not given; b = next step (Scholl reaction).

In 2007, attractive for OLED technology, flat nonographenes equipped with peripheral 2,6-dimethylphenyl groups were obtained by Cho and co-workers using a bottom-up strategy, especially DA cycloaddition with CO extrusion followed by Scholl dehydrocondensation (Scheme 220) [271].

Importantly, both experiments and theoretical calculation displayed that the steric effect of peripheral groups plays a key role in reducing aggregation (intermolecular distance is higher than 12 Ǻ), narrowing EL band, increasing emission in the solid state. HBC possessing such picket-fence-type substituents exhibits almost the same emissive properties both in solid state and solution.

**Figure 1:**
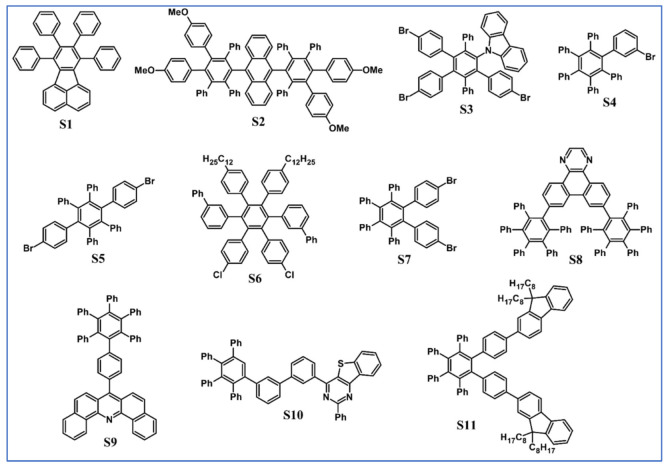
Patented polyarylbenzenes obtained via DA cycloaddition with CO extrusion (appropriate patent in the parentheses) **S1** [272], **S2** [273], **S3** [274], **S4** [275], **S5** [276], **S6** [277], **S7** [278], **S8** [279], **S9** and **S10** [280], **S11** [281].

Many DA cycloadditions with CO extrusion, involving cyclopentadienone derivatives and acetylenic dienophiles (disubstituted acetylenes, diynes, and polyynes), can be found in patents [272,273,274,275,276,277,278,279,280,281]. The obtained polyaryl benzenes were afterwards tested as materials for organic electronics. Sometimes, cycloaddition products underwent further transformations, e.g., Scholl reaction. The final products were tested as materials for organic electronics (OLEDs, photovoltaic cells), as graphene nanoribbon precursors, alkylated graphene (mimicking indigenous asphaltenes), and others. Selected product structures of the above-mentioned DA cycloaddition described in the patents are presented below (Figure 1).

#### 2.1.5. Cycloaddition of Cyclopentadienones and Non-Acetylenic Dienophiles

**Scheme 221:**
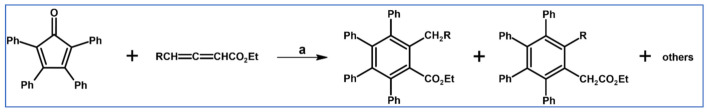
Allenic esters as dienophiles in DA cycloaddition with CO extrusion [282]. Reagents and conditions: R = Me or Et; a = PhH, 150 °C, 48 h, up to 25% yield.

Ishar et al., discovered that the DA cycloaddition with CO extrusion involving tetraphenylcyclopentadienone and allenic esters as diene and dienophiles, respectively, is not regioselective (Scheme 221) [282].

### 2.2. Cyclopentadienones for the Multisubstituted Naphthalene System Formation

This section discusses the DA cycloaddition with CO extrusion reactions and the products of such substituted naphthalenes. Cyclopentadienones played the role of the dienes, and arynes (especially benzyne) acted as the dienophiles.

**Scheme 222:**
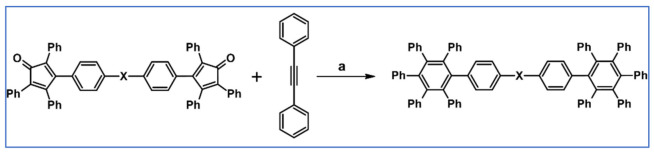
Preparation of bis-hexaphenylbenzene derivatives via DA cycloaddition with CO extrusion [64]. Reagents and conditions: X = nil or CH_2_ or *O* or *S*; a = heating; Y = up to 81%.

Bis-hexaphenylbenzene derivatives were obtained involving DA cycloaddition of diphenylacetylene and bis-tetraphenylcyclopentadienone derivatives (Scheme 222) [64].

**Scheme 223:**
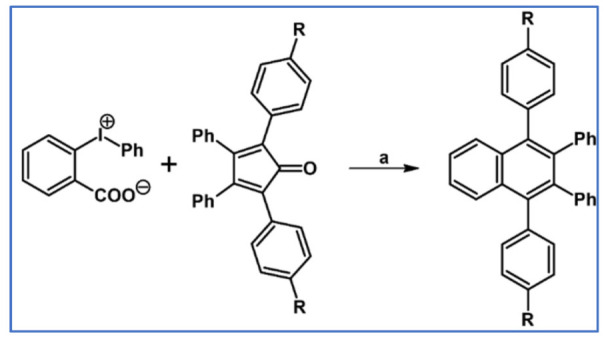
Syntheses of tetraarylnaphthalenes involving tetraarylsubstituted cyclopentadienones, in situ generated benzyne and DA cycloaddition with CO extrusion [283]. Reagents and conditions: R = H or OMe or NMe_2_; a = diglyme or γ-butyrolactone or triglyme, rfx (until the end of the CO evolution), up to 79% yield.

In 1964, Beringer and Huang reported obtaining tetraarylnaphthalenes via DA cycloaddition of benzyne (generated from 2-phenyliodonio benzoate) to tetraarylsubstituted cyclopentadienones (Scheme 223) [283].

In 1965 the only review on chemistry of cyclopentadienones, including their activity as dienes in the DA cycloadditions with CO extrusion, to date was published. As dienophiles in the described reactions, alkenes, nitryles, acetylenes, benzyne and other arynes and alkene derivatives were applied. In the above-mentioned work, several examples of cycloaddition involving cyclopentadienones and benzynes and heteroarynes were listed. Regarding benzynes, there were noted three cycloadditions of benzyne into tetraarylcyclopentadienone and one cycloaddition of 3-chlorobenzyne into tetraarylcyclopentadiene. Benzynes were generated from mercury–organic compounds or 2-halogeno- (or 2,4-dichloro)benzenecarboxylic acids salts. Moreover, Ogliaruso et al., reported the cycloadditions of hetarynes–hiophyne and *n*-methylindolyne (generated from halogeno–mercury–organic thiophene or indole derivatives, respectively), both to the tetraarylcyclopentadienone [284].

To sum up, it will be readily seen that DA cycloaddition reactions involving cyclopentadienones as dienes and arynes as dienophiles is much less than those with acetylenes. In our opinion, this is due to the good commercial availability and relatively simple synthesis of acetylenes of different structures, in contrast to arynes. However, more important is that DA cycloaddition has been combined with subsequent cyclodehydrocondensation, especially in the last two decades. This allows for the synthesis of coronenes, ovalenes, and nanographenes of various structures. Such DA cycloaddition–Scholl reaction tandem only concerns reactions starting from cycloaddition involving cyclopentadienones and acetylenes.

**Scheme 224:**
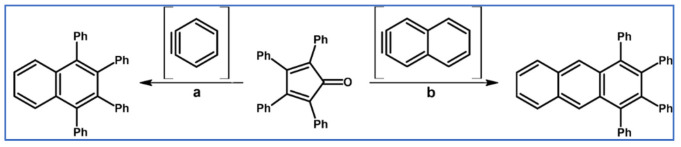
Synthesis of tetraphenyl- naphthalene and anthracene: DA cycloaddition of benzyne or 2,3-naphthyne and tetraphenylcyclopentadienone [59]. Reagents and conditions: a = benzyne source (conditions): diphenylidodonium carboxylate (diethylbenzenes, rfx, Y = 69%) or anthranilic acid (isopentyl nitrite, DME, rfx, Y = 93%); b = 2,3-naphthyne source (conditions): 3-amino-2-naphthalenecarboxylic acid, DME, rfx, Y = 73%).

Naphthalene and anthracene tetraphenyl derivatives were obtained involving DA cycloaddition of appropriate arynes and tetraphenylcyclopentadienones (Scheme 224) [59].

**Scheme 225:**
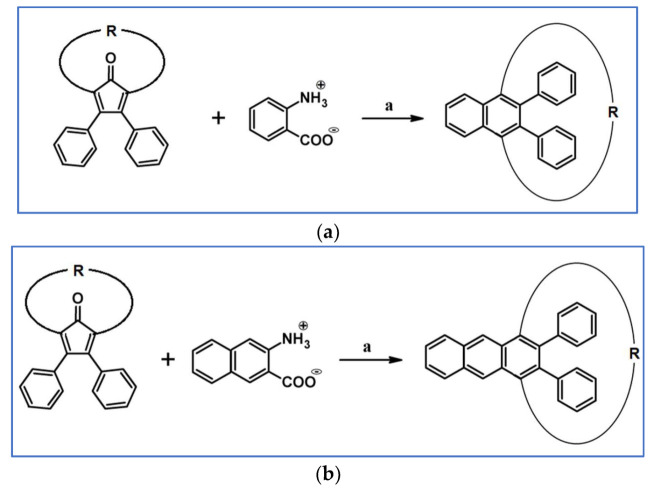
DA cycloaddition with CO-extrusion in the syntheses of [12]paracyclophanes [285]. Reagents and conditions: (**a**) R–R = (CH_2_)_12_ or R = *n*-C_3_H_7_; a = isoamyl nitrite, anthranilic acid, 1,2-dimethoxyethane, rfx, 1.25 h; Y = 63% (for R–R = (CH_2_)_12_); Y = 69% (for R = *n*-C_3_H_7_). (**b**) R–R = (CH_2_)_12_ or R = *n*-C_3_H_7_; a = isoamyl nitrite, anthranilic acid, 1,2-dimethoxyethane, rfx, 2 h; Y = 22% (for R–R = (CH_2_)_12_); Y = 39.5% (for R = *n*-C_3_H_7_).

In 1966, Dignan and Miller described the DA cycloaddition of 3,4-diphenyl-2,5-dodecamethylenecyclopentadienone or 3,4-diphenyl-2,5-dipropylcyclopentadienone with benzyne or 2,3-naphthyne to obtain the appropriate derivatives of [12]paracyclophanes (Scheme 225) [285].

**Scheme 226:**
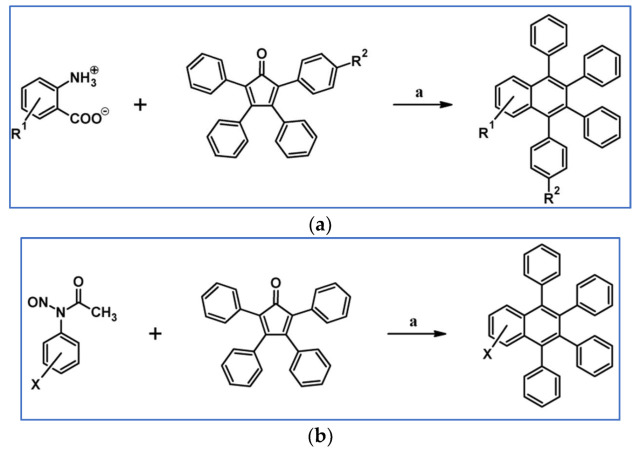
Synthesis of naphthalene derivatives via DA cycloaddition of substituted benzynes (generated from substituted anthranilic acid or nitrosoanilides) into cyclopentadienones [286]. Reagents and conditions: (**a**) R^1^ = 6-*t*-Bu, R^2^ = H; or R^1^ = H, R^2^ = *p*-*t*-BuPh; or R^1^ = 6-Cl, R^2^ = H; and others (substituents in naphthalene core). a = pentyl nitrite, CH_2_Cl_2_, acetone, rfx, up to 70% yield. (**b**) X = 5-, 6- or 7-Cl, 6-*t*-Bu. a = benzene, up to rfx, up to 50% yield.

As stated in 1971 by Brydon et al., decompositions of *N*-nitrosoacetanilides, including substituted and unsubstituted ones, in benzene and other solvents in the presence of 2,3,4,5-tetra-arylcyclopentadienones lead to naphthalene derivatives via DA cycloaddition (Scheme 226) [286]. Anthranilic acid derivatives were also used in this work as aryne sources in the DA cycloaddition to tetraarylcyclopentadienone (aryl = Ph) (Scheme 226) [286].

**Scheme 227:**
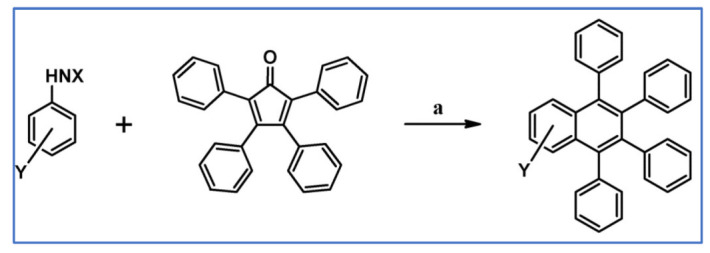
Synthesis of tetraphenylnaphthalene derivatives via cycloaddition of substituted benzynes to tetraphenylcyclopentadienone [287]. Reagents and conditions: X = H or COOMe; Y = H, F, Cl, Br, I, Me, *t*-Bu, and other substituents in various position. a = pentyl nitrite (for anilines) or *p*-chlobenzoyl nitrite (for anilides), up to 78% yield.

Subsequently, report from Baigrie and co-workers claimed that substituted anilines and anilides were smoothly converted into substituted benzynes, followed by the application of the benzynes as dienophiles in DA cycloaddition to tetraphenylcyclopentadienone (or in some cases 2,-diphenyl3,4-*p*-tolylcyclopentadienone) with CO extrusion (Scheme 227) [287].

**Scheme 228:**
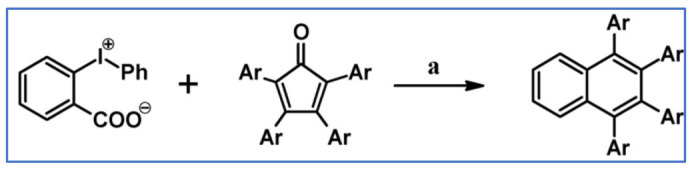
Cycloaddition of benzyne to tetraarylcyclopentadienone—synthesis of tetraarylnaphthalene [288]. Reagents and conditions: Ar = C_6_F_5_; a = triglyme, 200 °C, 10 min; Y = 27%.

Rausch et al., presented in 1979 the synthesis of tetra(pentafluorophenyl)naphthalene. This compound was obtained via cycloaddition of benzyne (in situ generated from diphenyliodonium-2-carboxylate) to tetrakis(pentafluorophenyl)cyclopentanedione (Scheme 228) [288].

**Scheme 229:**
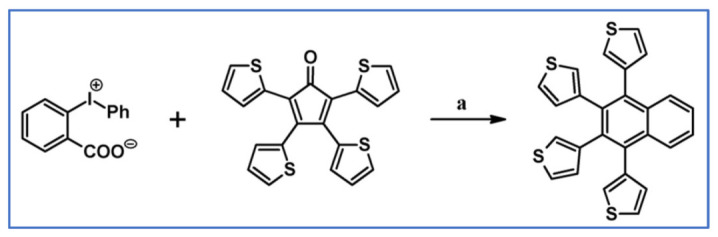
Synthesis of tetra(2-thienyl)naphthalene via DA cycloaddition with CO extrusion [86]. Reagents and conditions: a = glyme, 210 °C, 10 min; Y = 71%.

**Scheme 230:**
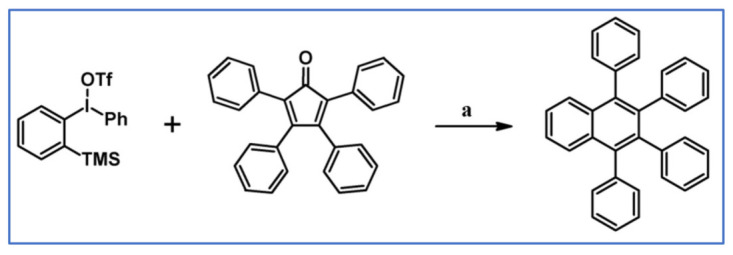
DA cycloaddition of benzyne and tetraphenylcyclopentadienone in the syntheses of tetraphenylnaphthalene [289]. Reagents and conditions: a = NBU_4_F, rt, CH_2_Cl_2_; Y = 100%.

Analogous cycloaddition of benzyne (generated from diphenyliodonium-2-carboxylate), however using tetra(2-thienyl)cyclopentadienone as a diene and leading to tetra(2-thienyl)naphthalene was described by Kawase et al., in 1994 (Scheme 229) [86].

(Phenyl)[*o*-(trimethylsilyl)phenyl]iodonium triflate (readily prepared from *o*-bis(trimethylsilyl)benzene) and the hypervalent iodine(III) reagent, namely [PhI(OAC)_2_*2TfOH, were used as a benzyne source under mild and neutral conditions and efficiently provided DA cycloaddition product, i.e., 1,2,3,4-tetraphenylnaphthalene (Scheme 230) [289].

**Scheme 231:**
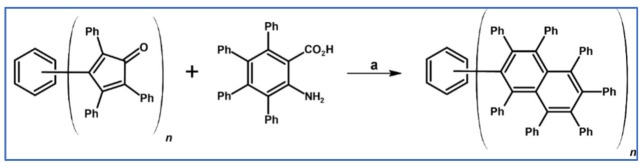
Synthesis of octaphenylnaphthalene via DA cycloaddition with CO extrusion [290]. Reagents and conditions: a = isoamyl nitrite, 1,2-dichloroethane, rfx, 1.5 h, Y = 62%.

Qiao’s research team presented in 1996 the preparation of octaphenylnaphthalene via cycloaddition with CO extrusion involving tetraphenylcyclopentadienone and tetraphenylbenzyne generated from tetraphenylanthranilic acid (Scheme 231) [290].

Slightly undulating molecular structure of the obtained compound was deeply analysed using X-ray and NMR analyses.

**Scheme 232:**
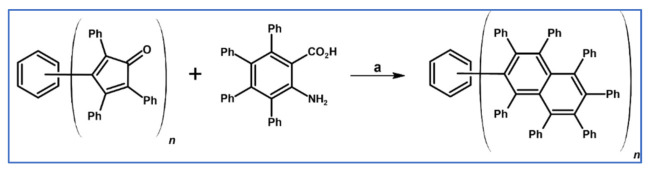
Syntheses of ‘Albatrossenes’—an example [87]. Reagents and conditions: n = 2 or 3; a = isoamyl nitrite, 1,2-dichloroethane, rfx, 4–9 h, Y = 5–15%.

As mentioned in Section 2.1.1., Tong et al., also described a synthesis of uniquely shaped C_2_-symmetric polyphenylenes via DA cycloaddition of benzyne into dienes possessing di- or tri-cyclopentadienone core, followed by CO extrusion (Scheme 232) [87].

**Scheme 233:**
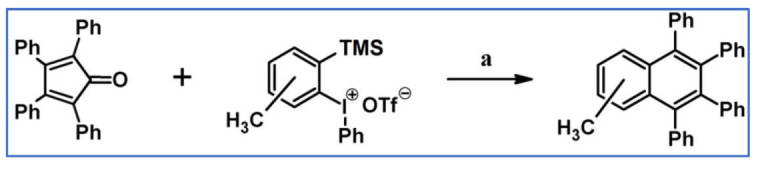
DA cycloaddition with CO extrusion engaging in situ generated methylbenzynes and tetraphenylcyclopentadienone: syntheses of naphthalene derivatives with quant. Yields [291]. Reagents and conditions: a = benzyne-precursor/diene = 1/5, Bu_4_NF, THF + CH_2_Cl_2_, rt, 30 min, quantitative yield.

In 1999, Kitamura et al., described a new and extraordinarily efficient hypervalent iodine-benzyne (or methylbenzynes) precursor, i.e., (phenyl or methyl-substituted)[2-(trimethylsilyl)phenyl]-iodonium triflate [291]. Application of this precursor and tetraphenylcyclopentadienone as a trapping agent allowed to obtain naphthalenes with quantitative yields (Scheme 233).

**Scheme 234:**
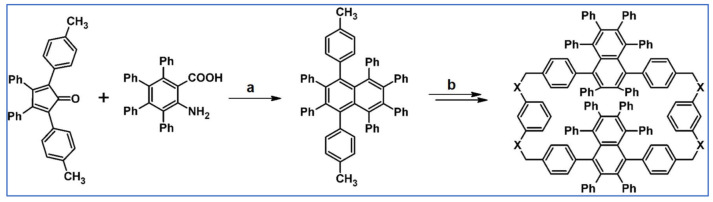
DA cycloaddition with CO extrusion in the syntheses of large cyclophanes [292]. Reagents and conditions: X = *S* or SO_2_; a = isoamyl nitrite, 1,2-dichloroethane, rfx, 2.5 h, Y = 83%.

According to Pascal and colleagues, DA cycloaddition of suitably substituted cyclopentadienone and benzyne was a key step in ynthesizing large cyclophanes (Scheme 234) [292].

**Scheme 235:**
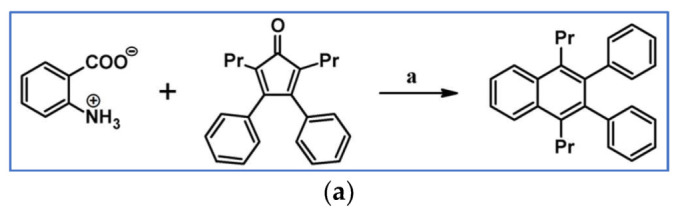

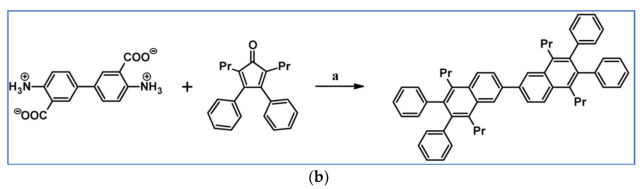
DA cycloaddition of arynes in the synthesis of tetrasubstituted naphthalene and octa-substituted 2,2′-binaphthalene [293]. Reagents and conditions: (**a**) a = ClCH_2_CH_2_Cl, isoamyl nitrite, rfx, 2.8 h; quantitative yield; (**b**) a = 1,2-dimethoxyethane/DMSO/1,1,1-trichloroethane, isoamyl nitrite, 110 °C, 19 h; Y = 50%.

As Mellakpour et al., reported in 2002, tetrasubstituted naphthalene and octa-substituted 2,2′-naphthalene derivatives can be obtained via DA cycloaddition of cyclopentadienone and benzyne or 4,4′-bis-benzyne, respectively (Scheme 235) [293]. Corresponding arynes were generated in situ from diazonium salts.

**Scheme 236:**
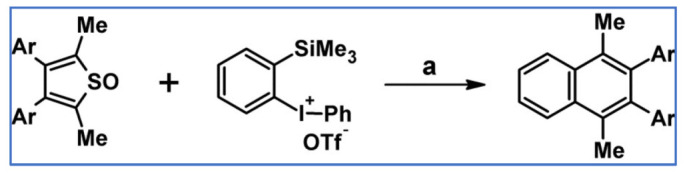
DA cycloaddition with formally SO extrusion involving in situ generated benzyne and thiophene oxide [294]. Reagents and conditions: Ar = *p*-MeOC_6_H_4_; a = Bu_4_NF, THF, rt, 1 h, Y= 55%.

In 2003, Thiemann and co-workers described synthesis of another tetrasubstituted naphthalene. DA cycloaddition*,* in situ generated benzyne and thiophene *S*-oxide were involved in a synthetic route (Scheme 236) [294].

For more comments see Section 4.

**Scheme 237:**
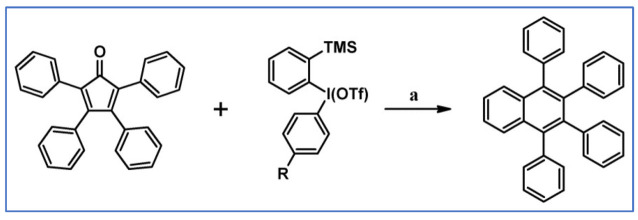
Highly soluble benzyne precursor for DA cycloaddition with tetraphenylcyclopentadienone [295]. Reagents and conditions: R = *n*-C_14_H_29_; a = Bu_4_NF, CH_2_Cl_2_/THF or CH_2_Cl_2_/toluene, rt, 0.5 h, Y = 89% Y = 86% or 89% (depending on the solvent mixture).

The same year, Kitamura et al., published a paper about long-chained hypervalent iodine-benzyne precursors soluble in most organic solvents, including nonpolar solvents such as diethyl ether, THF, CPME, benzene, and toluene. These precursors were synthesized and applied for DA cycloaddition, which led to tetraphenylcyclopentadienone (Scheme 237) [295].

Importantly, the above-mentioned benzyne precursors dissolve even in organic solvents of low polarity such as diethyl ether, THF, cyclopentyl methyl ether, benzene, and toluene.

**Scheme 238:**
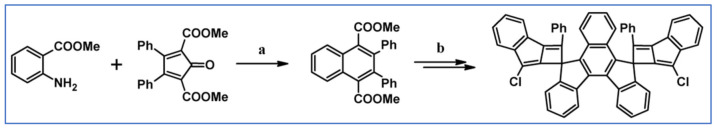
Synthesis of twisted spiro[1*H*-cyclobut[*a*]indene-1,9′-[9*H*]fluorenes] starting from DA cycloaddition of benzyne and appropriately substituted cyclopentadienones [100]. Reagents and conditions: a = isoamyl nitrite, Y = 26%.

Additionally, in 2003, Yang, Petersen, and Wang applied DA cycloaddition of benzyne and appropriately substituted cyclopentadienone for the synthesis of highly twisted 1,1-dialkyl-9,9-bifluorenylidenes—the main product is presented in Scheme 238 [100].

**Scheme 239:**
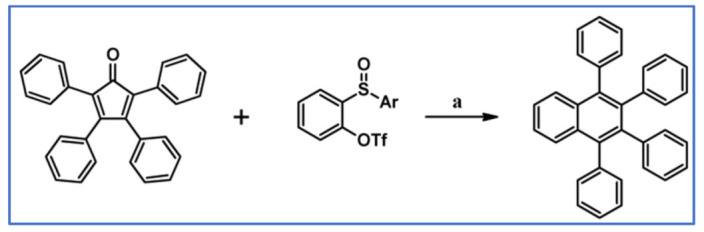
1,2,3,4-Tetraphenylnaphthalene synthesis via benzyne cycloaddition to tetraphenylcyclopentadienone [296]. Reagents and conditions: Ar = *p*-tolyl, a = benzyne precursor/dien = 1/5, PhMgBr, THF, −78 °C, 10 min, Y = 80%.

In 2014, Yoshida et al., showed that arynes could be efficiently generated from readily available ortho-sulfinylaryl triflates, triggered by the Grignard exchange reaction [296]. Benzyne precursor was applied for the synthesis of tetraphenylnaphthalene via trapping of in situ generated benzyne by tetraphenylcyclopentadienone (Scheme 239).

**Scheme 240:**
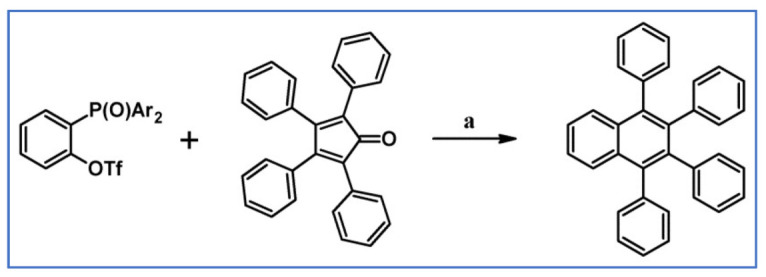
DA cycloaddition for the synthesis of tetraphenylnaphthalene: application of the new benzyne precursor [297]. Reagents and conditions: Ar = 1,3-bis(trifluoromethyl)phenyl; a = PhMgBr, THF, rt, 15 min, Y = 65%.

In 2018, a novel method for generating arynes, including disubstituted benzynes and a dehydrophenoxathiin was reported [297]. Namely, treatment of easily synthesizable *o*-(diarylphosphinyl)aryl triflates having two electron-deficient aromatic groups on the phosphorus atom with a phenyl Grignard reagent initiated the efficient generation of arynes. As far as DA cycloaddition leading to benzene or naphthalene system construction is concerned, this method was only employed in synthesising tetraphenylnaphthalene (Scheme 240) [297].

**Scheme 241:**
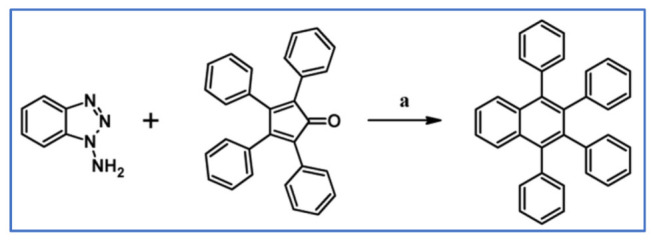
1-Aminotriazole as a benzyne source: synthesis of tetraphenylnapthalene [298]. Reagents and conditions: a = anodic conditions; Y = 78%.

In 2020, Li et al., described the synthesis of tetraphenylnaphthalene involving (for the first time) an electrochemical method for the generation of benzyne (Scheme 241) [298].

## 3. 2-Pyranones for the Multisubstituted Benzene Ring and Naphthalene System Formation/Creation

Undoubtedly, 2-pyranones are less often used than cyclopentadienones as dienes in DA cycloaddition reactions causing multisubstituted benzenes, naphthalenes, or more π-extended aromatic systems creation.

In our opinion, the limitations are insufficiently universal methods of dienes synthesis, i.e., piranones. Comparing 2-pyranones with cyclopentadienones, the synthesis of the latter substituted with very different substituents is more accessible. However, there are known several reactions involving 2-pyranones, especially with benzene, which lead to attractive nanomaterials, e.g., for OLED technology. Advances in the synthesis of substituted 2-pyranones, especially in all positions (including heteroaryls with solubilising groups), will undoubtedly contribute to the increased importance of DA cycloaddition with CO_2_ extrusion. Additionally, of interest are reactions with CO_2_ or both CO_2_ and N_2_ extrusion, with aza- and diaza-pyranones, respectively, which are presented in this section.

### 3.1. 2-Pyranones for the Multisubstituted Benzene Ring Formation

**Scheme 242:**
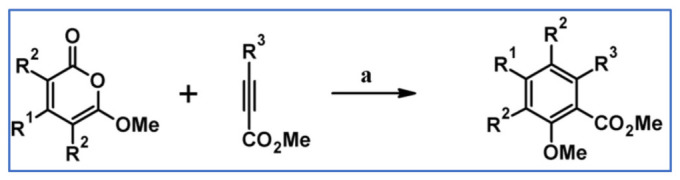
DA cycloaddition with carbon dioxide extrusion for the synthesis of benzenecarboxylates and benzenedicarboxylates [299]. Reagents and conditions: R^1^ = Me, R^2^ = H, R^3^ = COOMe; or R^1^ = *n*-pentyl, R^2^ = H, R^3^ = COOMe; or R^1^ = Me, R^2^ = R^3^ = H; and others, yield varied 35–95%.

In 1988, Ahmed et al., reported obtaining benzenecarboxylates and benzenedicarboxylates applying DA cycloaddition of 2-pyranones and methylpropiolates or methyl acetylenedicarboxylate (Scheme 242) [299].

**Scheme 243:**
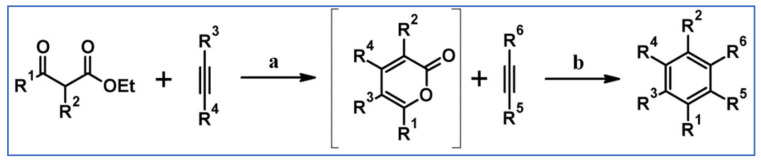
DA cycloaddition of acetylene into 2-pyranone—crucial step in the syntheses of multisubstituted benzenes [300]. Reagents and conditions: a = [ReBr,(CO)_3_(thf)]_2_, MS4A in toluene, 180 °C, 24 h, b = toluene, 150 °C, 24 h, R^1^ = R^2^ = Me, R^3^ = R^4^ = Ph, R^5^ = R^6^ = COOEt, Y = 83%; or R^1^ = R^2^ = Me, R^3^ =Me, R^4^ = Ph, R^5^ = R^6^ = COOEt, Y = 91%; or R^1^ = R^2^ = Me, R^3^ =H, R^4^ = Ph, R^5^ = R^6^ = COOEt, Y = 87%; and others.

In 2008 and then again in 2010, Kuninobu et al., published a reaction between a β-keto ester and an acetylene in the presence of a [ReBr(CO)_3_(thf)]_2_, as a catalyst, provided a 2-pyranone derivative and finally multisubstituted aromatic compounds via DA of acetylenes to pyranones (Scheme 243) [300,301]. Later, in 2010 they referred to the results when comparing manganese- and rhenium-catalysed synthesis of similar aromatic compounds [300]. However, only the reaction route presented in the scheme below involves the DA cycloaddition step (2-pyranone and acetylenes) [300,301].

We share the authors’ opinion that the aforementioned method is a powerful tool to synthesize multisubstituted aromatic compounds.

**Scheme 244:**
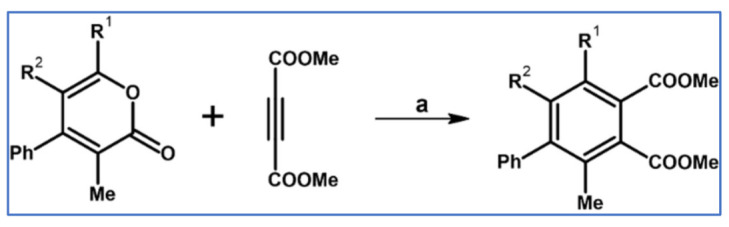
DA cycloaddition with CO extrusion for the synthesis of fully substituted 1,2-benzenedicarboxylates [302]. Reagents and conditions: R^1^, R^2^ = H, Ph; Ph, H; Ph, Ph; (CH_2_)_4_; CH_2_CH_2_CH(Me)CH_2_; and others; a = xylene, 180 °C, 48 h, up to 98% yield.

In 2009, Kim and co-coworkers developed an efficient synthetic protocol of fully substituted 2-pyranones starting from the Baylis–Hillman adducts. Subsequent DA cycloaddition of the obtained dienes and dimethyl acetylenedicarboxylate produced poly-substituted benzene 1,2-dicarboxylates and others polysubstituted aromatic compounds in high yields (Scheme 244) [302].

**Scheme 245:**
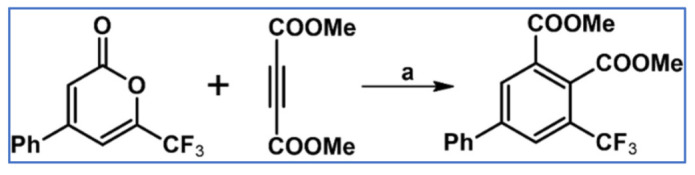
Highly effective synthesis of benzenedicarboxylate derivative via DA cycloaddition with CO_2_ extrusion [303]. Reagents and conditions: a = xylene, 200 °C, Y = 95%.

In the publication from 2014 about a new method of di- and trisubstituted 2-pyranones syntheses, authors mentioned the potential use of such compound in cycloaddition reactions with acetylenedicarboxylate (Scheme 245) [303]. There was also a reaction with ethyl propiolate, but it was not regioselective.

**Scheme 246:**
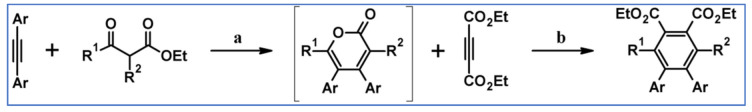
Acetylenedicarboxylate DA cycloaddition to in situ prepared 2-pyranones [304]. Reagents and conditions: R^1^ = Me or *n*-Pr, R^2^ = H, Me or Bn, Ar = Ph or 2,2′-bitiophen-5-yl; a = [ReBr(CO)_3_(thf)]_2_, MS4A, 180 °C, 24 h; b = 150 °C, 24 h, up to 60% yield.

In 2015, Kula with colleagues reported that a small molecule with D-A-D framework was prepared using a [2 + 1 + 2 + 1] cycloaddition (providing 2-pyranones) followed by the [4 + 2] Diels–Alders reaction with CO_2_ extrusion (Scheme 246) [304].

The preliminary tests of application possibility of synthesised compounds in devices for optoelectronics proved the attractiveness of those mentioned earlier for organic electronics.

**Scheme 247:**
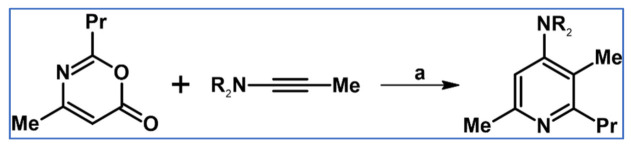
Aza-pyranone and ynamines in the syntheses of aminopyridines via DA cycloaddition with CO_2_ extrusion [305]. Reagents and conditions: a = benzene, 80 °C or toluene, 75 °C. R = Et, Y = 83%; or R = Bn, Y = 69%.

Another notable case is the synthesis of pyridine derivatives via DA cycloaddition involving azapyranone described in 2017 by Duret and his colleagues. It is noteworthy that the reactions occur under mild conditions with good yields (Scheme 247) [305].

**Scheme 248:**
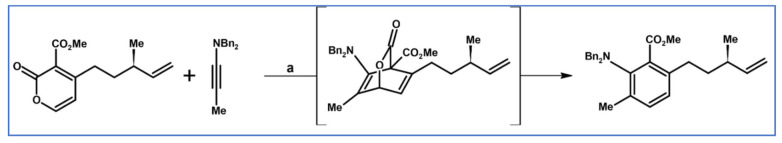
DA cycloaddition with carbon dioxide extrusion engaged in the synthesis of substituted methyl benzoate [305]. Reagents and conditions: a = PhMe, rfx, overnight, Y = 80%.

As authors stated, methyl 2-dibenzylaminobenzenecarboxylate can also be smoothly obtained via DA cycloaddition with CO_2_ extrusion using electron-rich ynamine and electron-poor diene, i.e., 2-pyranone (Scheme 248) [305].

**Scheme 249:**
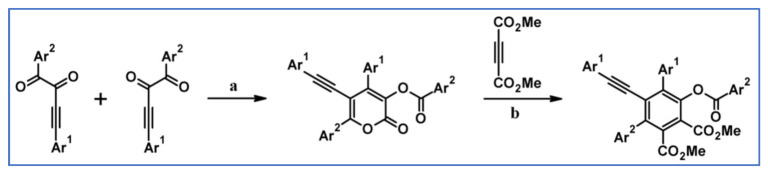
NHC-catalysis for the synthesis of alfa-pyranones. Application of the selected pyranone for the synthesis of hexasubstituted benzene via DA cycloaddition with CO_2_ extrusion [306]. Reagents and conditions: Ar^1^ = Ar^2^ =Ph, a = NHC-carbene source, Cs_2_CO_3_, THF, Y = 99%; b = 1,2-dichlorobenzene, 180 °C, 6 h, Y = 41%.

Kong and colleagues achieved the umpolung of alkynyl 1,2-diketones via *N*-heterocyclic carbene catalysis for the first time in 2017. This attempt allowed rapid access to xmany synthetically and pharmaceutically important α-pyrones under very mild conditions [306]. The possibility of applying the pyranones mentioned above in cycloaddition with acetylenedicarboxylate was also shown (Scheme 249).

**Scheme 250:**
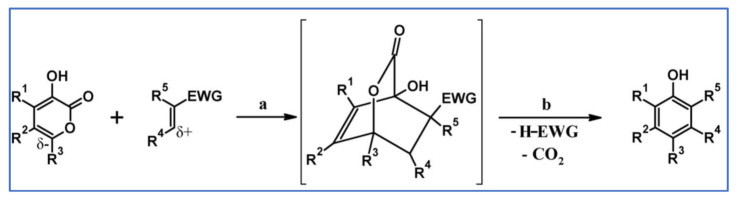
Polysubstituted phenols synthesis via DA cycloaddition with CO extrusion involving hydroxypyranones (as dienes) and nitroalkenes (as masked dienophiles) [307]. Reagents and conditions: a = AlCl_3_, *o*-C_6_H_4_Cl_2_, BHT, 150 °C. R^1^ = R^2^ = R^3^ = R^5^ = H, R^4^ = *t*-Bu, Y = 85%; or R^1^ = *t*-Bu, R^2^ = R^3^ = H, R^4^ = Et, R^5^ = Me, Y = 70%; or R^1^ = *t*-Bu, R^2^ = Me, R^3^ = Ph, R^4^ = COOMe, R^5^ = Et, Y = 70%; and others; b = next step.

In 2020, Zhang and Beaudry reported a direct and regiospecific for the alkene synthesis of substituted phenols from 3-hydroxypyrones and nitroalkenes (as masked alkynes) [307]. This reaction proceeds by a Diels−Alder−retro-Diels−Alder sequence (Scheme 250).

Phenol derivatives are crucial precursors and target compounds in pharmaceutic science, plant protection, and other traditional and modern technologies. On that account, Zhang and Beaudry’s publication is essential. It was confirmed that AlCl_3_ activates pyranone derivatives, while the addition of butylated hydroxytoluene protects the reagents against thermal decomposition.

**Scheme 251:**
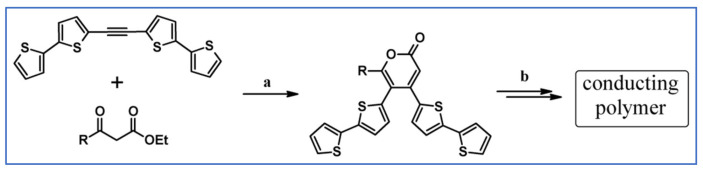
Synthesis and electropolymerisation of 2-pyranone containing bithienyl moieties [308]. Reagents and conditions: a = [ReBr(CO)_3_(thf)]_2_, MS4A, 180 °C, toluene, 24 h, b = next steps. R = Me, Y = 73%; or R = Ph, Y = 60%; or R = 2,3,4,5-tetrafluorophenyl, Y = 57%.

In 2021, Kula et al., described obtaining 2-pyranones, containing bithienyl motifs, which enabled the polimerisation of such 2-pyranones (Scheme 251) [308].

The synthesised monomers of Bt-py-Bt (where Bt = 2,2′-bithiophene-5-yl, py = pyranone moiety) type were transformed into conducting polymers in an electrochemical oxidation process. The influence of the pyranone bridge on the stability, luminescent, and (spectro)electrochemical behaviour of polymers proved to be very important. Namely, the polymers presented extraordinary stability during both multiple n- and p-doping.

### 3.2. 2-Pyranones for the Multisubstituted Naphthalene System Formation/Creation

**Scheme 252:**
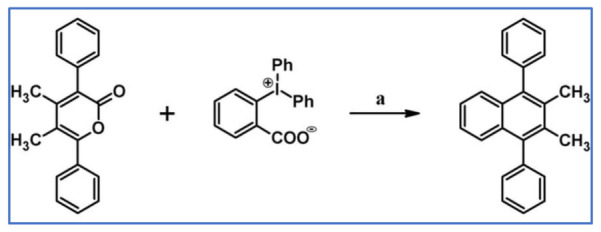
DA cycloaddition with benzyne and 2-pyranone: synthesis of a naphthalene derivative [309]. Reagents and conditions: a = diethylbenzene, heating, Y = 65%.

The first record of pyranones used as dienes in DA cycloaddition dates back to 1973. Ishibe et al., published a paper showing that diphenyliodonium-2-carboxylate (as a benzyne source) and 3,6-diphenyl-4,5-dimethyl-2-pyranone were applied for the synthesis of tetra-substituted naphthalene via DA cycloaddition with CO extrusion (Scheme 252) [309].

In the 1986 Research Report focused on cycloaddition of 1,3,4-oxadiazin-6-ones in organic synthesis, the application of such readily available compounds in the synthesis of PAHs was described [310].

**Scheme 253:**
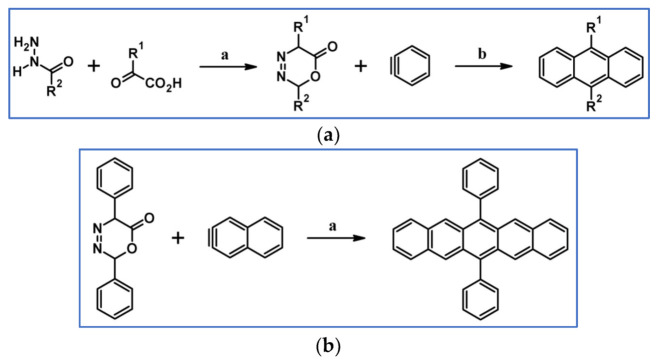
1,3,4-Oxadiazin-6-ones synthesis and application in DA cycloaddition to arynes with both CO_2_ and N_2_ extrusion [310]. Reagents and conditions: (**a**) a = dehydrating agent (not specified); b = dehydrating agent (not specified); R^1^ = R^2^ = Ph, Y = 60%; or R^1^ = Ph, R^2^ = 1-naphhtyl, Y = 52%; or R^1^= 1-naphthyl, R^2^ = Ph, Y = 52%; or R^1^ = 1-naphthyl, R^2^ = 4-ClC_6_H_4_, Y = 62%; (**b**) a = dehydrating agent (not specified), Y = 60%.

**Scheme 254:**
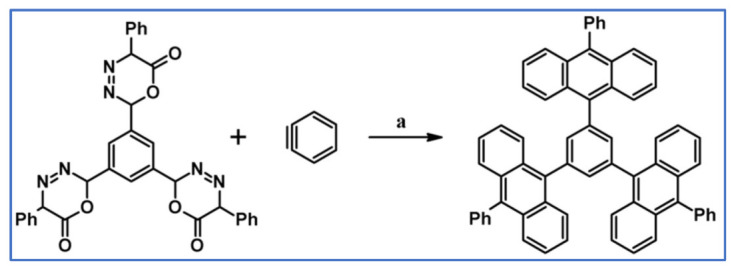
1,3,4-Oxadiazin-6-ones in the synthesis of diphenylpentacene via DA cycloaddition to 2,3-naphthyne with both CO_2_ and N_2_ extrusion [310]. Reagents and conditions: a = dehydrating agent (not specified), Y = 24%.

Importantly, for the scope of this review, the derivatives can be treated as diaza-2-pyranones. In the course of the reaction of 1,3,4-oxadiazin-6-ones with arynes, thermal extrusion of both CO_2_ and N_2_ occurs. The syntheses described in the literature are presented in Scheme 253 and Scheme 254 [310].

**Scheme 255:**
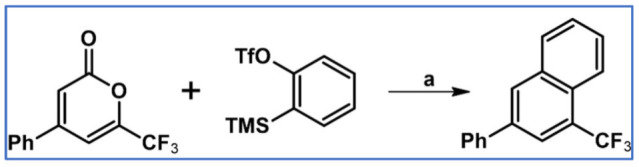
Synthesis of disubstituted naphthalene derivative via DA cycloaddition with CO_2_ extrusion [303]. Reagents and conditions: a = CsF, MeCN, 100 °C, Y = 54%.

As already mentioned in Section 3.1, in 2014 Yeh et al., developed a new method of di- and trisubstituted 2-pyranones’ synthesis. One of the described examples is applicable in cycloaddition reactions (concerning the reaction with benzyne) (Scheme 255) [303].

**Scheme 256:**
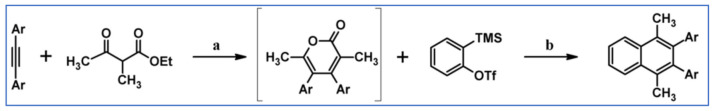
DA cycloaddition of benzyne to (in situ prepared) 2-pyranones: synthesis of tetrasubstituted naphthalene [304]. Reagents and conditions: Ar = Ph or 2,2′-bithiophen-5-yl; a = [ReBr(CO)_3_(thf)]_2_, MS4A, 180 °C, 24 h; b = rt, 24 h, up to 60% yield.

In 2015, Kula et al., reported synthesising tetra-substituted naphthalene derivatives via DA cycloaddition of in situ generated benzyne to 2-pyranones (Scheme 256) [304].

Both obtained compounds exhibit strong 2D-π-conjugation. The influence of this type of interaction on photophysical properties with the aid of DFT calculations was examined. The preliminary tests of the application possibility of synthesised compounds in devices for optoelectronics were optimistic [304].

**Scheme 257:**
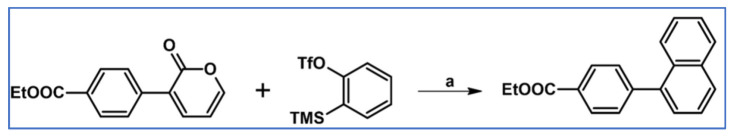
DA cycloaddition with CO_2_ extrusion involving 3-(4-ethoxycarbonylphenyl)-pyran-2-one and in situ generated benzyne [311]. Reagents and conditions: a = CsF, MeCN, 100 °C, Y = 78%.

In 2017, Huang et al., presented in their work a new method of 2-pyranone derivatives’ synthesis, namely 3-aryl substituted 2-pyranones [311]. They also showed the possibility of using one of the obtained pyranones in the aryne DA cycloaddition with CO_2_ extrusion (Scheme 257).

**Scheme 258:**
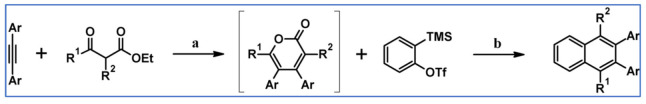
DA of in situ prepared 2-pyranones to in situ generated benzyne: synthesis of napthalene derivative containing two bithiophene or carbazole motifs [312]. Reagents and conditions: R^1^ = Me, R^2^ = H or Me, Ar = *N*-ethylcarbazole-3-yl or 2,2′-bithiophen-5-yl; a = [ReBr(CO)_3_(thf)]_2_, MS4A, 180 °C, 24 h; b = CSF, MeCN, rt, 24 h, up to 65% yield.

Another achievement was described in 2018 by Szłapa-Kula et al. A series of solution-processable tetra-substituted naphthalene derivatives bearing thiophene or carbazole units were synthesised using the tandem cycloaddition [2 + 1 + 2 + 1] and DA cycloaddition with CO extrusion (Scheme 258) [312].

Thermal, electrochemical, absorption, and emission properties of synthesised compounds exhibited that they can be considered molecular glasses with a glass transition temperature ranging from 37 to 122 °C with high thermal stability up to 300 or 400 °C. Moreover, all products were luminescent, electrochemically active, and showed a low energy band gap between 1.64 and 1.85 eV. Pre-application tests confirmed that the analyzed derivatives of naphthalene show the best properties as a material for OLED technology when Ar substituent is *N*-ethylcarbazol-3-yl, but R^1^ = Me, R_2_ = H.

**Scheme 259:**
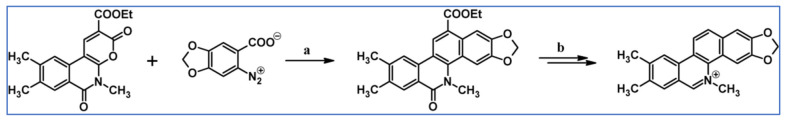
DA cycloaddition of aryne and 2-pyranone in the synthesis of nitidine [313]. Reagents and conditions: a = 80 °C, Y = 88%, b = other steps.

In 2019, Cai published the review devoted to the 2-pyranones applications (as the diene component) in the total syntheses of natural products. One of the syntheses described in this review meets this review’s criteria. Namely, the aromatic product is created via thermal CO, CO_2_, or SO_2_ extrusion without any other reagent, especially dehydrogenating or oxygenating (Scheme 259) [313].

**Scheme 260:**
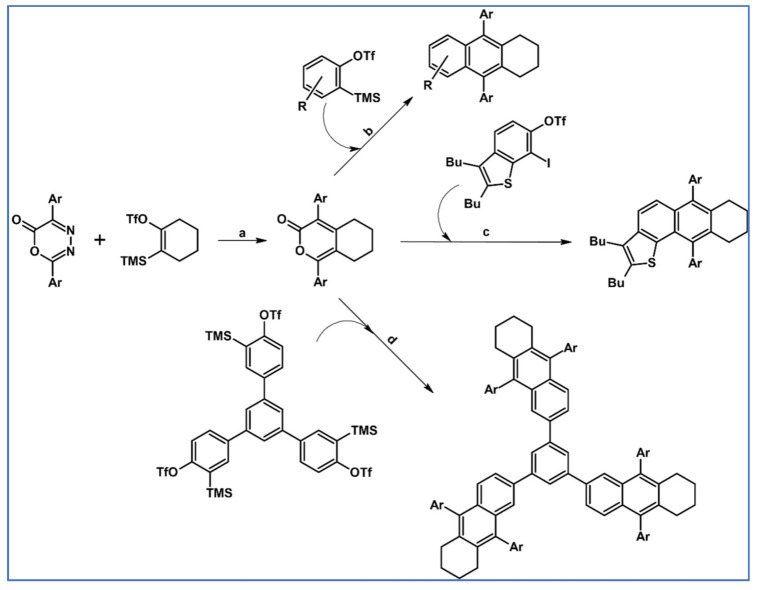
Syntheses of polysubstituted benzenes involving DA cycloaddition with N_2_ followed by CO_2_ extrusion [314]. Reagents and conditions: a = KF, 18-Crown-6, THF, 60 °C, Y = 85% (for Ar = Ph) or a = Bu_4_N[SiPh_2_F_2_], THF, 60 °C, Y = 91% (Ar = 2-thienyl); b = Bu_4_N[SiPh_2_F_2_], THF, 60 °C, Ar = Ph, R = H, Me, (MeO)_2_ and others; Ar = 2-thienyl, R = (MeO)_2_, up to 78% yield; c = Me_3_SiCH_2_MgCl, THF, −78 °C; d = KF, 18-Crown-6, THF, 60 °C, Y = 33%.

Tomohiro Meguro et al., used diazapyranones as valuable substrates for the synthesis of polyaryl- or hetarylsubstituted benzenes containing up to three six-membered saturated and benzene-fused ring (Scheme 260) [314]. In the first step, diazapyrones were transformed into pyranones containing cyclohexane-fused ring via DA cycloaddition with N_2_ extrusion. The obtained pyranones were used as substrates in DA cycloaddition with arynes leading to the final product.

According to the authors, the obtained compounds can be attractive for material and medicinal chemistry, primarily due to their structure of arene-fused saturated six-membered rings, difficult to introduce in any other way. Some of the above-presented reactions 2-pyranones with arynes were briefly enumerated in the review from 2021 [315].

## 4. 1,1-. Dioxothiophene for the Benzene and Naphthalene Ring Formation

There are only a few known applications of the [4 + 2] reaction with SO_2_ extrusion using 1,1-dioxothiophenes as dienes and dienophiles possessing triple bond (alkynes, arynes). The described examples of such reactions are limited to several works in which the dienes are tetrachloro- and tetrabromothiophenes or thiophenes containing other substituents, even adamantyl. In recent years, the progress of this variant of DA cycloaddition has not been observed, although tetrachloro- and tetrabromothiophenes are readily available. This is obviously as further functionalization of the obtained tetrahalogene-substituted benzene derivative via coupling and other transformations is necessary. As for thiophenes having substituents other than F, Cl, or Br, the products of such cycloadditions, i.e., benzene and naphthalene derivatives, can be obtained using more synthetically accessible dienes, namely cyclopentadienones.

**Scheme 261:**
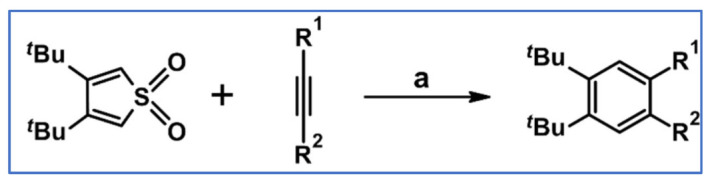
Syntheses of *o*-di-tert-butylbenzene derivatives [316]. Reagents and conditions: a = appropriate solvent, up tu 230 °C, up to 45 h; R^1^,R^2^ = CO_2_Me, CO_2_Me (Y = 100%); H, CO_2_Me (Y = 72%); H, COMe (Y = 84%); H, Ph (Y = 92%); H, *n*-Bu (Y = 32%); PhS, NEt_2_ (Y = 34%), and others.

The first DA cycloaddition with SO_2_ extrusion, 3,4-di-tert-butyl-1,1-dioxothiophene as a diene, mono-, and disubstituted acetylenes involved in the syntheses of 1,2-di-tert-butyl benzene derivatives (Scheme 261) was described by Nakayama in 1988 [316].

Moreover, when in situ generated benzyne or phenyl-vinyl sulfoxide were used as dienophiles giving 2,3-di-tert-butylnaphthalene or 1,2-di-tert-butylbenzene, respectively (with 40 and 77% yield).

**Scheme 262:**
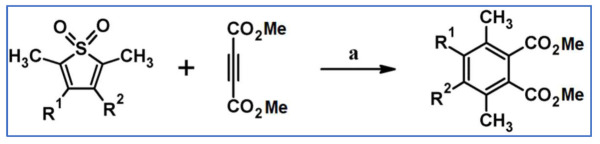
DA cycloaddition with SO_2_ extrusion for the synthesis of benzenedicarboxylates bearing two bulky group in the position 4 and 5 [317]. Reagents and conditions: R^1^ = R^2^ = *t*-Bu, 1-adamantyl or neopentyl; R^1^ = 1-adamantyl, R^2^ = *t*-Bu; a = 1,2-dichlorobenzene, rfx, up to 87% yield.

Highly congested 1,1-dioxothiophenes, namely 3,4-di-tert-butyl-, 3,4-di(1-adamantyl)-, 3,4-dineopentyl-, and 3-(1-adamantyl)-4-tert-butylthiophenes, were prepared in satisfactory overall yields by intramolecular reductive coupling of 3-thiapentane-1,5-diones followed by acid-catalyzed dehydration of the resulting thiolane-3,4-diols and finally, oxidation of the obtained thiophenes with *m*-CPBA gave the corresponding thiophene 1,1-dioxides in good yields. The obtained 1,1-dioxides underwent DA reactions with alkynic dienophile, i.e., acetylene dicarboxylate resulting in the corresponding benzene derivatives carrying two bulky substituents on adjacent positions (Scheme 262) as reported by Nakayama and co-workers in 1998 [317].

**Scheme 263:**
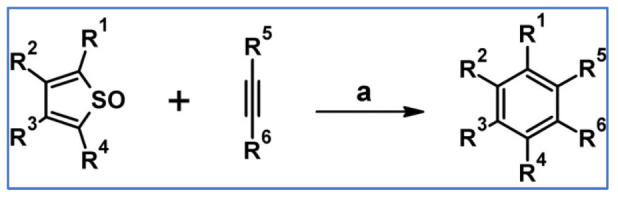
DA cycloaddition with SO extrusion involving substituted cyclopentadienones and acetylenes [294]. Reagents and conditions: for one-pot version: a = *m*-CPBA, DMC, from 0 °C to rfx, up to 85 h: R^1^–R^4^ = Me; R^2^ = R^3^ = H; R^5^ = R^6^ = CO_2_Me (Y = 41%); R^1^ = R^4^ = Me; R^2^ = R^3^ = H; R^5^ = R^6^ = COPh-*p*-However, (Y = 29%); R^1^ = R^4^ = Me; R^2^ = R^3^ = H; R^5^ = COOMe; R^6^ = COPh (Y = 32%); R^1^ = R^4^ = R^2^ = R^3^ = Me; R^5^ = R^6^ = COOMe (Y = 23%). For the two-step version: R^1^ = R^2^ = R^3^ = R^4^ = Ph, R^5^ = R^6^ = COPh; R^1^ = R^2^ = R^3^ = R^4^ = *t*-Bu, R^5^ = R^6^ = COPh and others; a = CHCl_3_ or benzene, up to 80 °C, up to 36 h; yield up to quantitative.

In 2003, Thiemann et al., described DA cycloaddition of in situ generated thiophene *S*-oxides (or prepared in a separate step) and acetylenes involved in synthetic route leading to a wide range of substituted arenes (24 examples) (Scheme 263) [294].

The best yields are obtained when the thiophene *S*-oxides are donor and acceptor groups are substituents in the alkynes. As far as the mechanism of this reaction is concerned, it has not been explained. The authors claim that SO bridge extrusion takes place, which results in cycloadduct aromatization.

**Scheme 264:**
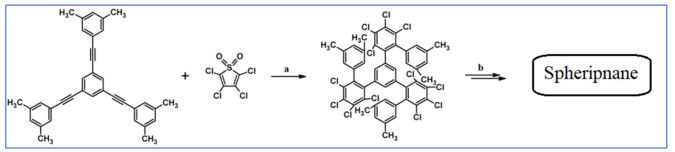
DA cycloaddition (with SO_2_ extrusion) between “tri-acetylene” and dioxo-tetrachlorothiophene for a spheriphane syntheses [318]. Reagents and conditions: a = xylene, 150 °C, 46 h; Y = 59%, mixture of stereoisomers was obtained, b = next steps leading to spheriphane.

According to Nierle and Kuck, DA cycloaddition with SO_2_ extrusion was one of the crucial steps in the synthetic route to the globular cyclophane (a C_48_H_36_-spheriphane) (Scheme 264) [318].

It should be added that only using dioxotiophene enables the smooth introduction of four chlorine (or bromine) atoms to the desired molecule. It results from the easy synthesis and thermal stability of tetrahalogenothiophenes. Importantly, analogous derivatives of 2-pyranone and cyclopentadienone do not exist or have limited stability. According to the authors, compounds shown in Scheme 264 can play the role of fullerene precursors.

**Scheme 265:**
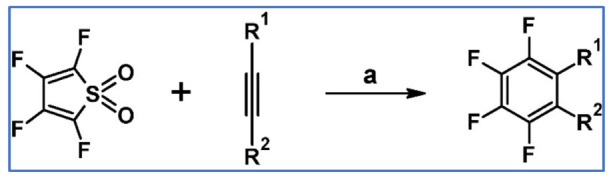
Syntheses of substituted arenes via DA cycloaddition with formally SO_2_ extrusion [319]. Reagents and conditions: a = up to 110 °C; up to 28 h; appropriated solvent; R^1^, R^2^ = Ph, Ph (Y = 34%); COOMe, COOMe (Y = 53%); Me, Et (Y = ~35%); Me_3_Si, Me_3_Si (Y = 67%); Ph, H (Y = overall 96%, [2 + 2]/[4 + 2] = 2, 8/1); cyclohexen-1-yl, H (Y = overall 95%, [2 + 2]/[4 + 2] = 3, 2/1); n-Bu, H (Y = overall 80%, [2 + 2]/[4 + 2] = 1/1.2).

In the article by Lemal devoted to cycloaddition of tetrafluorothiophene 1,1-dioxide seven examples of DA cycloaddition with SO_2_ extrusion, involving the above-mentioned diene and di- (four examples) and monosubstituted (three reactions) acetylenes were presented (Scheme 265) [319].

Interestingly, in the case of terminal alkynes the orbital-forbidden [2 + 2] cycloaddition is strongly present or even dominant.

**Scheme 266:**
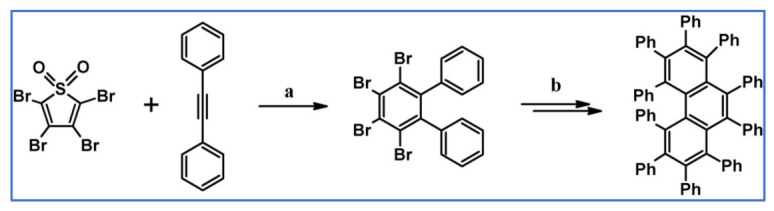
DA of diphenylacetylene and tetrabromothiophene as an initial step in the syntheses of DPhA [320]. Reagents and conditions: a = xylene, rfx, Y = 35%; b = next two steps.

In 2020, Xiao’s research group reported DA cycloaddition between 1,1-dioxo-2,3,4,5-tertrabromothiophene and diphenylacetylene (with SO_2_ extrusion) employed in the syntheses of decaphenylphenanthrene (DPhA) (Scheme 266) [320].

X-ray analysis and theoretical calculations showed that the phenanthrene core of DPhA is strongly twisted, and the whole molecule is not configurationally stable at room temperature.

## 5. 1,2-. Diazines and 1,2,4,5-Tetrazines for the Benzene Ring Formation

The number of such reactions (with N_2_ extrusion) described in the scientific literature is very limited. Importantly, as in the case of cycloaddition with 1,1-dioxothiophenes, no development of this type of reaction has been observed in the last 15–20 years. This is, of course, due to difficulties in synthesizing corresponding substituted 1,2-diazines and 1,2,4,5-tetrazines and the low atom economy of the latter.

**Scheme 267:**
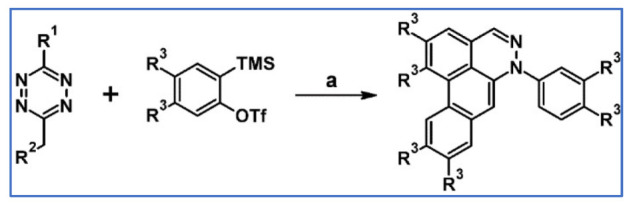
Syntheses of polyaromatic heterocycles starting from DA cycloaddition with N_2_ extrusion [321,322]. Reagents and conditions: R^1^, R^2^, R^3^ = Me, H, H; *n*-C_9_H_19_, H, H; *p*-BrPh, H, H; 2-thienyl, H, H; Et, Me, H; Ph, H, Me; Et, Et, Me and others, up to 52% yield; a = TBAF, CH_2_Cl_2_, 24 °C, 5 min (overall cascade time).

Suh et al., presented intriguingly effective access to a new class of polyaromatic heterocycles, starting from DA cycloaddition with N_2_ extrusion, involving tetrazines and benzyne as only substrates (Scheme 267) [321,322].

DFT theoretical calculations showed that the first step of this multistep cascade involved benzyne cycloaddition with CO extrusion, followed by several steps involving the two consecutive benzyne molecules [322]. Interestingly, emission spectra of the neutral and protonated form of the obtained heterocycles can be tuned, thanks to diverse substituents, in the wide range of visible spectrum. Therefore, they are interesting, i.e., as fluorophores for bioimaging [321].

**Scheme 268:**
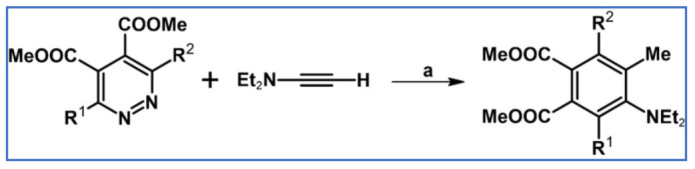
Derivatives of 1,2-diazine and ynamine for the synthesis of amino-1,2-benzenedi- or tetracarboxylates via DA cycloaddition with N_2_ extrusion [305]. Reagents and conditions: a = MeCN, rfx; R^1^, R^2^ = H, H; COOMe, COOMe and others.

As reported in 2017 by Duret et al., dialkylaminobenzenedi- or even tetracarboxylates can be smoothly obtained via DA cycloaddition with CO_2_ extrusion using electron-rich ynamines and electron-poor dienes, i.e., 1,2-diazines possessing up to four CO_2_Me substituents (Scheme 268) [305].

**Scheme 269:**
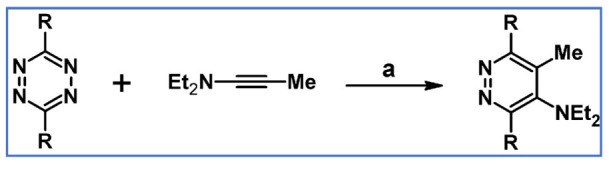
1,2,4,5-Tetrazines and *N*,*N*-diethyl-*N*-(1-propynyl)amine involving in the syntheses leading to 4-(diethylamino)-1,2-diazine derivatives [305]. Reagents and conditions: a = heating, solvent; R = Ph, COOMe, Me, SMe and others; up to 100% yield.

In the same work, synthesis of 4-(diethylamino)-1,2-diazine derivatives using 1,2,4,5-tetrazines and *N*,*N*-diethyl-*N*-(1-propynyl)amine as partners in DA cycloaddition with N_2_ was presented (Scheme 269) [305].

## 6. Conclusions

The state of the art regarding the selected variants of Diels–Alder cycloaddition reaction has been presented. The partners are cyclopentadienones, 2-pyranones, 1,1-dioxothiophenes, 1,2-diazines, and 1,2,4,5-tetrazines as dienes and acetylenes (including masked ones), diynes and arynes as dienophiles. These reactions proceed with extrusion of CO (cyclopentadienones), CO_2_ (pyranones), SO_2_ (1,1-dioxothiophenes), and N_2_ (diazines and tetrazines), respectively. Importantly, this review discusses thermal reactions without the participation of other reagents, e.g., bases (except the reagents necessary for in situ arynes generation) and the participation of catalysts. Moreover, it should be marked that in 1965 a review on the chemistry of cyclopentadienones was published, and it covered cycloaddition with CO extrusion, with acetylenes and arynes. Therefore, this review presents works on cyclopentadienones after that date and selected works quoted in this review, yet commenting on them in more detail. Generally, in the discussed area of science, cycloaddition with CO extrusion dominates, involving cyclopentadienones and acetylenes as dienes and dienophiles, respectively. Publications devoted to reactions of such type constitute more than 90% of all articles devoted to the title cycloaddition reactions, and in the last 20 years—more than 95%. This is as convenient methods for synthesising cyclopentadienones and acetylenes with virtually any structure have been developed. Secondly, the progress of Scholl cyclodehydrocondensation and the APEX strategy, in general, have resulted in DA cycloaddition with CO extrusion being (and now—almost always has been) combined with the follow-up Scholl reaction. Additionally, various functionalisation methods, particularly the coupling reactions (which development is spectacular), are involved in the synthesis of more and more π-expanded and precisely predesigned functionalised PAHs. Thus, starting from the readily available cyclopentadienones and acetylenes and engaging DA cycloaddition and other reactions, Scholl reaction especially, there is π-expansion of polyphenylenes and their functionalisation. Notably, the cyclodehydrocondensation reactions due to high regioselectivity allow obtaining PAHs and nanographenes with the expected structure. PAHs and nanographene pieces are obtained with the planned size and molecular structure, with the expected curvature, extraordinary optical, electrochemical, and other properties. The above-mentioned advantages of the DA cycloaddition with CO extrusion, Scholl reaction and various type of coupling as part of the bottom-up strategy also apply to heterodoped nanographenes, in the structure of which heteroatoms such as *N*, *B*, *S*, and others are in strictly defined, designed positions.

When it comes to DA reactions with the participation of multisubstituted 2-pyranones (tetrasubstituted are especially important), the insufficiently universal methods of their synthesis are certainly a limitation. Comparing tetrasubstituted 2-pyranones with tetrasubstituted cyclopentadienones, the synthesis of the latter substituted with very different substituents is more approachable. However, many reactions involve 2-pyranones, especially with benzyne, which lead to attractive nanomaterials, e.g., suitable for OLED technology, conducting polymers. Advances in the synthesis of substituted multisubstituted 2-pyranones, especially in all positions, including heteroaryls with solubilising groups, will undoubtedly contribute to the increased importance of DA cycloaddition with CO_2_ extrusion.

In contrast, reactions with SO_2_ extrusion are limited to tetrachloro- and tetrabromothiophenes, and no development of this variant of DA cycloaddition is observed. Obviously, further functionalisation of the obtained tetrahalogene-substituted benzene derivative via coupling and other transformation is necessary. However, obtaining the target structure is most often possible differently, i.e., using cyclopentadienones instead of dioxothiophenes. Additionally, thermal reactions with N_2_ or HCl extrusion have not been developed for many years. As in the case of the reaction with CO_2_ and SO_2_ extrusion, this is due to the undeniable advantage of DA cycloaddition using cyclopentadienones and dienophiles equipped with a triple bond, in particular acetylenes, diynes, and, less frequently, arynes.

The combination of DA cycloaddition with CO extrusion involving cyclopentadienones and acetylenes, with Scholl reaction and others transformation, e.g., Buchwald type amination, is worth emphasising Suzuki–Miaura coupling, is particularly intensively used in materials chemistry for several years now. Thanks to this combination of these two main reactions and the aforementioned reactions leading to a strictly planned functionalisation of the final structure, it is possible to obtain functionalised nanomaterials, including nanografenes and polymers with the expected properties, starting from readily available substrates. The obtained nanomaterials, including aggregated ones, polymers, thanks to their unique optical properties, including nonlinear, electrochemical, and photophysical, are attractive as biosensors, materials for OLED technology, solar cells technology, conductive polymers, nanoreactors or ligands for catalysts, semiconductors, and detectors of exploding substances.

An analysis of the progress of cycloaddition, cyclodehydrocondensation, and various types of coupling reaction shows that the importance of DA cycloaddition with small molecules extrusion, especially with CO extrusion, will continue to grow. Due to the spectacular progress of synthesis methods, it is possible to plan structures and even tune their properties, especially when it comes to bottom-up and APEX strategies. Fortunately, we understand better and better the influence of shape and size of the molecules on the properties of polyphenylenes and nanographenes; in particular, the role of different forms of deformation, heteroatom dopping, and functional and peripheral groups is being acknowledged.

An example may be attractive for OLED technology, flat nanographenes equipped with peripheral 2,6-dimethylphenyl groups obtained through bottom-up strategy, i.e., DA cycloaddition with CO extrusion followed by Scholl dehydrocondensation [271]. Both experiments and theoretical calculations have shown that the steric effect of peripheral groups has a crucial role in limiting aggregation (intermolecular distance is higher than 12 Ǻ), narrowing EL band and increasing solid-state emission. HBC (hexabenzocoronene) possessing such picket-fence-type substituents has almost the same emission properties in solid state as in solution [271]. As a second example, nanographenes obtained using bottom-up strategy (DA cycloaddition with CO extrusion followed by Scholl reaction) were characterised by negative or positive curvature, depending on the Scholl reaction conditions [323].

Interestingly, detailed X-ray, photophysical, Raman, electrochemical, and theoretical studies have shown that seemingly small changes in the geometry of the obtained nanographenes have enormous consequences in terms of their properties. However, series of synthetic challenges are faced by organic synthesis polyphenylenes and nanongraphenes via DA cycloaddition with CO extrusion–Scholl reaction. For instance, the synthesis of BN-doped polyphenylenes characterised by various shapes leading to regularly BN-doped nanographenes, e.g., via cyclodehydrocondensation, remains challenging. Nevertheless, the scope of possibilities of various materials and nanomaterials synthesis using DA cycloaddition with CO extrusion, Scholl reaction, and other advanced synthetic methods is so vast that this combination of crucial reactions is of growing importance for diverse technologies.

Suppose an attempt was made to identify the most important and prospective achievements of the discussed reactions for material chemistry and modern technologies. In that case, it is undoubtedly necessary to point out the production of functionalized nanographenes applicable in organic electronics. There is also increasing interest in unique materials and nanomaterials for catalysis, analytical devices and bioimaging. Selected relevant and spectacular applications of DA cycloaddition with CO, CO_2_, N_2_, and SO_2_ in material chemistry are presented below (our subjective selection).

**Materials for organic electronics**

- Unwrapped HBC derivative forming a stable columnar liquid crystalline mesophase and characterized by good solution processability, long-range order, possessing the great potential to be an active component in organic electronic devices (Scheme 48).
- Ar_5_Ph-L-PhAr_5_ type and star-shaped hexarylbenzene derivatives belonging to donor-acceptor-donor type nanomaterials is characterized by antenna effect, high glass transition temperature, and efficient electroluminescence (Scheme 49).
- Hexaphenylbenzene derivatives bearing diphenylamine or N-carbazole moieties have high triplet energy levels and were tested in blue organic electrophosphorescent devices, i.e., a blue OLED (Scheme 59). The device with a blue phosphorescent emitter, iridium(III) bis[(4,6-difluorophenyl)pyridinato-*N*,C^2^′]-picolinate, exhibit a high external quantum efficiency of 11% (24 cd/A) and a high power efficiency of 12 lm/W at 100 cd/m^2^.
- Sizeable colloidal graphene quantum dots with a uniform and tunable size through solution chemistry (Scheme 70). Above-mentioned quantum dots have significant extinction coefficients in a wide spectral range from UV to near-infrared and, thus, can serve as a new type of light-harvesting media for photovoltaics.
- Hyperbranched polymers containing HPB were characterized by good solubility in organic liquids, by film-forming ability and thermal stability, and by emitting pure, deep-blue light in a stable manner in air and at increased temperatures (Scheme 72). Two-layer PLED devices fabricated from the polymer mentioned above were promising as far as their electroluminescence, luminance efficiency, and brightness are concerned.
- Hybrid materials featuring the dipolar fragment 6*H*-indolo[2,3-*b*]quinoxaline were attached to the bulkier polyaromatic hydrocarbons such as fluoranthene, triphenylene, or polyphenylated benzene (Scheme 84). Optical, electrochemical, and other properties suggest that obtained compounds are promising electron-transporting and emitting materials suitable for double layer organic light-emitting diodes.
- Free-base porphyrin with two HBC moieties and its Zn^2+^ derivative was obtained (Scheme 108). Due to the substantial electron transfer between the two chromophores, HBC-porphyrin conjugate exhibits excellent absorption and emission, making it a potential building block for carbon-rich supramolecular functional architectures in the field of organic electronics.
- Attractive for OFET polymeric-PAHs possessing *t*-butyl peripheral groups were obtained (Scheme 129). The obtained polymeric PAHs (before Scholl reaction) were characterized by increasing degree of planarity and decreasing bandgaps, varied weight-average molecular weights, and intermolecular π–π-stacking. As far as polymers after Scholl dehydrocondensation are concerned, the differences between bandgaps and oxidation onset increase.
- The APEX-bottom-up strategy application in the synthetic route leads/led to a carbon nanoring-shaped-nanographene containing four HBC moieties equipped with mesityl-peripheral groups (Scheme 130). The obtained large π-expanded [4]HBC was characterized by unique photophysical properties, especially by forming a 1:1 host-guest complex with fullerene C_70_.
- Luminescent HBC derivatives equipped with donor and acceptor motifs were synthesized (Scheme 134). The obtained functionalized nanographene is characterized by significant dipole moment, remarkable solvatochromic emission and high fluorescence quantum yield, atypical as far as other HBCs is concerned. Due to the properties mentioned above, the obtained compound can be attractive for optoelectronics.
- Attractive optoelectronics HBCs bearing alkyl chain-DNA fragment were reported (Scheme 135). The presence of both hydrophobic and amphiphilic fragments results in powerful self-assembly ability and finally leads to 2D micrometre-size sheets. Amphiphilic properties of DNA fragment mainly drive sheet creation, but π-stacking of HBC fragments and van der Waals interaction between alkyl chains are also important for photophysical properties.
- The synthesis of a distorted carbonyl group and *t*-butyl end-groups containing ribbon-shaped nanographene was reported (Scheme 138). This nanomaterial is characterized by extraordinary properties, namely: two-photon absorption-based up-conversion, circularly polarized luminescence, and long emission lifetime.
- Indeno[2,1-*c*]fluorene-based with a narrow full width strong blue fluorescent oligomers and polymers were synthesized for UV light-emitting devices (Scheme 141).
- Syntheses of porphyrin bearing two Ph_5_Ph or two HBC motifs and their Zn-complexes (Scheme 155). The obtained nanomaterials are characterized by intense and long-distant electronic communication and a small HOMO–LUMO gap. Due to the above-mentioned advantages, such porphyrins are attractive for light-harvesting and charge transport in optoelectronic devices.
- The family of distorted ribbon-shaped nanographenes is characterized by non-linear optical properties (Scheme 188). The idea of introducing distortions on nanographenes in a well-defined manner is a viable molecular engineering strategy to tune their properties for optoelectronic devices.
- Three-dimensional non-planar perylene bisimide derivative (PBI-derivative) with fluorinated shell was obtained (Scheme 196). Thanks to their properties, namely high luminescent quantum yield, excellent amorphous and thermal stability, and electrochemical features, the obtained compound can be qualified as a potential multifunctional material, i.e., *n*-type red emitter for non-doped OLEDs and electron acceptor for solar cells.
- A thieno-[3,4-*b*]-pyrazine derivative bearing four –C_6_H_4_–C_6_H(2,3,4,5–C_6_F_5_)_4_ groups Scheme 199. These groups perform several important functions: ensure good thermal stability, significant Stokes shift, prevent emission quenching, and facilitate electron injection. Due to the above-mentioned advantages, the commented compound is an attractive OLED material characterized by red electroluminescence and good brightness and luminance.
- Atomically precise bottom-up synthesized graphene nanoribbons (GNRs) are considered promising candidates for next-generation electronic materials (Scheme 207). Cutting-edge synthetic tools applied in this procedure allowed to fully control the implementation of the obtained semiconductors into devices characterized by complex architectures. Controlling or tuning the size, sequence of structural elements, types of end-groups, and hierarchical assemblies turned out to be essential. Moreover, using a copolymer templating strategy will enable integration leading to well-defined heterojunctions and single-graphene nanoribbons transistors.
- Application of bottom-up strategy for syntheses of attractive for OLED technology, flat nanographenes equipped with peripheral groups thanks to such picket-fence-type substituents exhibit almost the same emissive properties both in solid state and solution (Scheme 220). Importantly, the steric effect of peripheral groups plays a crucial role in reducing aggregation, narrowing EL band, increasing emission in the solid state.

**Materials for catalysis**

- An *N*-doped oligophenylene derivative aggregates can act as reactors and stabilizers for the generation of palladium nanoparticles in the Sonogashira coupling under aerial conditions, at room temperature (Scheme 117).
- HBCs bearing DNA fragment attached through C_4_-alkyl chain were reported (Scheme 135). The presence of both hydrophobic and amphiphilic fragments results in powerful self-assembly ability and finally leads to 2D micrometre-size sheets. Amphiphilic properties of DNA fragments mainly drive sheet creation, but π-stacking of HBC fragments are also important for applaying in biocatalysis.
- Preparation of Au-Fe_3_O_4_ containing nanocomposites encapsulated inside AIE-active, supramolecular, porous hetero-oligophenylene nanomaterial (Scheme 150). This precursor was utilized to develop photo-active, easily separated, and reusable catalysts for aniline reactions with propiolate, leading to quinoline or aminoacrylate derivatives in mild and eco-friendly conditions. AIE activity of supporting material (for Au-Fe_3_O_4_) plays a crucial role in the significant increase in the efficiency of the photocatalytic system.
- Reusable, highly active in mild, aerial conditions and visible light irradiation supramolecular catalytic system (HP-T@Au-Fe_3_O_4_) for Kumada cross-coupling and Heck reaction, containing magnetic Fe_3_O_4_ (Scheme 159).
- AIEE active hexarylbenzene derivative forms fluorescent aggregates in aqueous media (Scheme 176). The obtained aggregates are applied as reactors and stabilizers to prepare in situ generated copper nanoparticles (CuNPs). The CuNPs served as effective catalysts for the alkyl–azide ‘click’ reactions to synthesize 1,2,3-triazoles could be recycled and reused five times without substantial loss of their catalytic activity.

**Materials for biochemistry (bioimaging, molecular recognition)**

- A new class of polyaromatic heterocycles is characterized by tunable emission from the neutral and protonated form in the wide visible spectrum range. Therefore, they are interesting, i.e., as fluorophores for bioimaging.
- Promisingly for molecular recognition, HBCs bearing DNA fragment attached through C_4_-alkyl chain were reported (Scheme 135). The presence of both hydrophobic and amphiphilic fragments results in powerful self-assembly ability and finally leads to 2D micrometre-size sheets. Amphiphilic properties of DNA fragment mainly drive sheet creation, but π-stacking of HBC fragments and van der Waals interaction between alkyl chains are also important and decisive as far as application for molecular recognition.

**Materials for analytical chemistry/analytical devices**

- Imidazolium-based amphiphilic hexa-peri-hexabenzocoronenes are characterized by ordered columnar self-assembly in solid and solution, leading to well-defined nanofibers (Scheme 61). Due to the above-mentioned phenomena, one should expect that those nanofibers will exhibit satisfying ion conductivity and charge mobility.
- Penta- and hexaarylbenzenes equipped with specific chromophores exhibit AIE (Scheme 88). Both compounds were envisioned as perfect selective sensors for TNT in solution, solid, and vapour state (at the picogram level).
- *N*-doped, hetero-oligophenylene easily create aggregates, which in H_2_O + THF solution exhibits the AIEE phenomenon (Scheme 93). These aggregates can be used as a selective biological probe for particular proteins and the selective indication of some metal ions.
- A tripticene derivative possessing three HBC motifs constitute an example of 3D nanographene (Scheme 96). After appropriate functionalization, it can be used as an in vivo and in vitro bioimaging agent characterized by low toxicity and good anti-photobleaching properties.
- Sterically encumbered sulfonated, poly(arylene ether) copolymers were prepared for membranes manufacturing (Scheme 111). The membranes parameters are as follows: (a) ion exchange capacities: 1.9 to 2.7 mmol/g; (b) proton conductivities: 0.24 to 16 mS/cm at 80 °C/60% RH, and 3 to 167 mS/cm at 80 °C/95% RH; excellent mechanical strength.
- HPB-based polymers is characterized by intrinsic microporosity with methyl, bromine, and nitrile substituents (Scheme 112). Modifying polymer structures by introducing the polar groups allows effective tuning of membrane properties. In particular, it increases the selectivity towards specific gases, especially carbon dioxide.
- *N*-doped heterooligophenylene possessing carbazole and 2-pirydyl moieties can act as the super-effective threonine detectors in aqueous solution and the presence of Zn^2+^ ions (Scheme 115).
- The derivative of HPB bearing two imine-quinoline motifs can selectively complex Zn^2+^ ions (Scheme 127). This ligand exhibits aggregation-induced emission enhancement (AIEE) characteristics and undergoes fluorescence enhancement in the presence of only Zn^2+^ ions in aqueous media. Significantly, the in situ synthesized Ligand–Zn^2+^ combination was employed as a valuable tool for selective detection of pyrophosphate ions in aqueous media.
- The syntheses of HBC with two permethylated-β-cyclodextrin moieties were executed (Scheme 131). Self-assembly of this molecule is characterized by a specific fluorescence response as far as exploders are concerned. Detection of 2,4,6-trinitrophenol can be reached at 306 and 2 ppb in solution and on the test paper, respectively, what seems to meet practical requirements of exploder detectors.
- Hexabenzoperylenes possessing special peripheral groups exhibit semiconductivity (Scheme 137). This nanomaterial is charecterized by the ability to π-stacking and are attractive as a supramolecular platform for sensing applications. Such HBPs used in devices integrating an OFET channel and a microfluidic channel enable the detection of diverse chemical and biological objects.
- Methyl acetate functionalized HBP (hexabenzoperylene) self-assembles into a π-stacked supramolecular nanosheet (Scheme 152). Such active ester-functionalized HBP could be used for manufacturing analytical devices for effective differentiation between primary, secondary, and tertiary amines in water solutions.

## 7. Remaining Challenges

In our opinion, when it comes to unresolved problems, i.e., the challenges facing cycloaddition with CO, SO_2_, CO_2_, and N_2_ extrusion, there are at least three significant issues to point out. The first issue to be addressed is to find efficient catalytic systems that enable reactions between cyclopentadienones and acetylenes and diynes or polyynes to be carried out at lower temperatures. This would allow the application of less thermally stable functionalized dienophiles, especially conjugated diynes. The second is a problem of significantly extending the possibility of obtaining multisubstituted 2-pyranones, the synthesis of which is complex (or even fails), especially when they contain long alkyl chains. Thirdly, the regioselective reactions of cyclopentadienones and 2-pyranones with di- or poly-arynes, e.g., benzodines leading to more extensive aryne precursors, remain a challenge. Pd-mediated cyclotrimerization of the latter would open up new possibilities in synthesizing star-shaped nanographenes. Equally important is the further development of the Scholl reaction, which is often the last stage of multistep procedures leading to complex structures, including nanographenes.

The goal is finding solutions, particularly oxidizing systems that limit intermolecular reactions. Perhaps the development of an electrochemical variant of cyclodehydrocondensation will allow the implementation of many reactions in a strictly regioselective manner, which is an advantage of electrochemical oxidation. The reaction mechanism is also underexplored. Although the first stage, i.e., DA cycloaddition between dienes and triple bond-contained dienophiles (including FMO interactions), is well known, the critical stage mechanism, i.e., extrusion CO (and also CO_2_, N_2_, and SO_2_) has not been explained yet. Extrusion can proceed in one stage (which is unlikely) or in two stages through diradical structures. Importantly, extrusion is by far the slowest stage in this multistep transformation starting from DA-adduct and leading to benzene or naphthalene rings. After extrusion, the electron system must be reorganized (probably swiftly) with the formation of an aromatic system. Undoubtedly, theoretical calculations of possible reaction pathways are necessary.

## Abbreviations

A–D–A	Acceptor-Donor-Acceptor
AIE	aggregation-induced emission
AIEE	aggregation-induced emission enhancement
APEX	annulative π-extension
Bpin	bis(pinacolato)diboron
Bt	2,2′ -bithiophene-5-yl group
CPME	cyclopentyl methyl ether
CuNPs	copper nanoparticles
CV	cyclic voltammetry
DA	Diels–Alder
DAC	Diels–Alder cycloaddition
D–A–D	donor-acceptor-donor
DAT	2,4-diamino-1,3,5-triazin-6-yl
DBTF	dibromoterfluorene
DDQ	2,3-dichloro-5,6-dicyano-1,4-benzoquinone
DFT	density functional theory
DMC	dimethyl carbonate
DNA	deoxyribonucleic acid
DPhA	decaphenylphenanthrene
DSSC	dye-sensitized solar cell
EA	electron acceptor
ED	electron donor
EDG	electron-donating group
EL	electroluminescence
EQE	external quantum efficiency
EWG	electron-withdrawing group
FHBC	fluorenylhexabenzocoronene
FMO	frontier molecular orbital
FWHM	full width at half maximum
GNR	graphene nanoribbon
HAB	hexaarylbenzene
HBC	hexabenzocoronene
HBP	hexabenzoperylene
HEHBC	heptagon-embedded hexabenzocoronene
HOMO	highest occupied molecular orbital
HPB	hexaphenylbenzene
HPLC	high-performance liquid chromatography
L	ligand
LUMO	lowest unoccupied molecular orbital
*m*-CPBA	*meta*-chloroperoxybenzoic acid
MDM	molecular dipole moment
MOF	metal-organic framework
MW	microwaves
NBS	*n*-bromosuccinimide
NG	nanographene
NHC	*N*-heterocyclic
NLO	nonlinear optical
NMR	nuclear magnetic resonance
NP	nanoparticle
OFET	organic field-effect transistor
OLED	organic light-emitting diode
PAH	polycyclic aromatic hydrocarbon
PBI	perylene bisimide
Pc	phthalocyanine
PEG	poly(ethylene glycol)
PLED	polymer organic light-emitting diode
PMCD	permethylated-β-cyclodextrin
RH	relative humidity
ROMP	ring-opening metathesis polymerization
SBQ	superbenzoquinone
SubPc	boron subphthalocyanine
TADF	thermally activated delayed fluorescence
TBTQ	tribenzotriquinacene
TFT	thin-film transistor
THF	tetrahydrofuran
TIPS	triisopropylsilyl group
TMS	trimethylosilyl group
TNT	trinitrotoluene
tpy	2,2′:6′,2′′-terpyridine
TSCT	through-space charge transfer
UV	ultraviolet
